# Photochemical Reactivity of Naphthol-Naphthalimide Conjugates and Their Biological Activity

**DOI:** 10.3390/molecules26113355

**Published:** 2021-06-02

**Authors:** Matija Sambol, Patricia Benčić, Antonija Erben, Marija Matković, Branka Mihaljević, Ivo Piantanida, Marijeta Kralj, Nikola Basarić

**Affiliations:** 1 Department of Organic Chemistry and Biochemistry, Ruđer Bošković Institute, Bijenička cesta 54, 10000 Zagreb, Croatia; m.sambol01@gmail.com (M.S.); Antonija.Erben@irb.hr (A.E.); Marija.Matkovic@irb.hr (M.M.); Ivo.Piantanida@irb.hr (I.P.); 2 Fidelta Ltd., Prilaz Baruna Filipovića 29, 10000 Zagreb, Croatia; 3 Division of Molecular Medicine, Ruđer Bošković Institute, Bijenička cesta 54, 10000 Zagreb, Croatia; patricia.bencic@pharmazie.uni-freiburg.de (P.B.); marijeta.kralj@irb.hr (M.K.); 4 Institute of Pharmaceutical Sciences, Albert-Ludwigs-Universität Freiburg, Albertstr. 25, 79104 Freiburg, Germany; 5 Division of Material Chemistry, Ruđer Bošković Institute, Bijenička cesta 54, 10000 Zagreb, Croatia; branka.mihaljevic@irb.hr

**Keywords:** naphthols, naphthalimides, FRET, PET, quinone methides, antiproliferative activity

## Abstract

Quinone methide precursors **1a**–**e**, with different alkyl linkers between the naphthol and the naphthalimide chromophore, were synthesized. Their photophysical properties and photochemical reactivity were investigated and connected with biological activity. Upon excitation of the naphthol, Förster resonance energy transfer (FRET) to the naphthalimide takes place and the quantum yields of fluorescence are low (Φ_F_ ≈ 10^−2^). Due to FRET, photodehydration of naphthols to QMs takes place inefficiently (Φ_R_ ≈ 10^−5^). However, the formation of QMs can also be initiated upon excitation of naphthalimide, the lower energy chromophore, in a process that involves photoinduced electron transfer (PET) from the naphthol to the naphthalimide. Fluorescence titrations revealed that **1a** and **1e** form complexes with ct-DNA with moderate association constants *K*_a_ ≈ 10^5^–10^6^ M^−1^, as well as with bovine serum albumin (BSA) *K*_a_ ≈ 10^5^ M^−1^ (1:1 complex). The irradiation of the complex **1e@BSA** resulted in the alkylation of the protein, probably via QM. The antiproliferative activity of **1a**–**e** against two human cancer cell lines (H460 and MCF 7) was investigated with the cells kept in the dark or irradiated at 350 nm, whereupon cytotoxicity increased, particularly for **1e** (>100 times). Although the enhancement of this activity upon UV irradiation has no imminent therapeutic application, the results presented have importance in the rational design of new generations of anticancer phototherapeutics that absorb visible light.

## 1. Introduction

Reagents that can alkylate and cross-link DNA molecules are among the most cytotoxic molecules, often employed in anticancer treatment [1]. Among the molecules that have been intensively studied as DNA cross-linking agents are quinone methides (QMs), reactive intermediates in phenol chemistry and photochemistry [2]. The biological activity of QMs [3,4] has been connected to their reactivity with proteins [5,6,7], nucleobases [8], DNA [9,10,11,12] and G-quadruplexes [13,14,15,16]. Moreover, the antiproliferative action of the anticancer antibiotic mitomycin [17,18,19] is rationalized by the intracellular formation of QMs, followed by reversible DNA cross-linking [20,21,22]. An important advantage in the use of QMs in biological systems stems from the fact that QMs can be generated in photochemical reactions under mild conditions [23,24], which allows for the spatial and temporal control of their formation. However, QMs react efficiently with H_2_O molecules [25,26,27], which competes with the reactivity with biological targets such as DNA. Therefore, for efficient DNA alkylation and cross-linking, it is important to attach to the QM precursor units to groups that can bind to DNA through noncovalent interactions [10].

We have investigated the photoinduced antiproliferative effect of several classes of QM-precursors, consisting of hydroxybiphenyls [28,29], naphthols [30,31], and anthrols [32,33]. Some anthrol derivatives were particularly interesting since they did not exhibit cytotoxicity in the dark, but upon exposure to near-visible light the cytotoxicity was enhanced 100 times and showed selectivity against cancer stem cells [33]. However, for the ultimate application of QM precursors in biological systems, it is important to develop molecules that can generate QMs upon excitation with visible light [34], as well as to increase their reaction selectivity with biological targets, which can be facilitated via the substitution of QM precursors to DNA binding units [10]. In that context, we have explored the application of QM precursors attached to phenanthridine [35]. Further modifications are required, since in these examples QMs were generated by UV light, precluding their application in living cells.

**Scheme 1:**
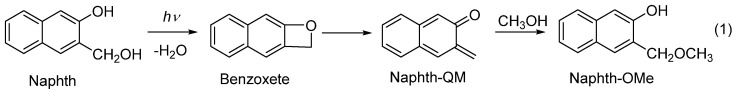
Photodehydration of Naphth to quinone methide.

Herein we present an investigation of the photochemical reactivity and noncovalent and covalent binding of a series of naphthol-naphthalimide conjugates, **1**. Naphthol QM precursors are known to undergo efficient photodehydration and deliver QMs (Scheme 1) [36], which can be applied in biological systems and labelling [6,37,38,39,40]. Our new QM precursors are connected by an alkyl linker of different length to 1,8-naphthalimide, which is a known pharmacophore, exhibiting antiviral [41,42,43], analgesic [44], local anesthetic [45], antitrypanosomal [46] and antagonist activity against 5-HT_3_ and 5-HT_4_ receptors [47,48,49]. Of particular interest is the ability of 1,8-naphthalimide derivatives to intercalate into DNA [50,51], which was exploited in the development of anticancer agents [52,53], initiating further clinical studies [54,55,56,57,58]. Furthermore, 1,8-naphthalimides undergo photoinduced electron transfer with guanine-rich polynucleotides [59,60] and induce cleavage of the chains [61,62]. Consequently, newly designed molecules contain two warheads for their anticancer activity. We have investigated the photochemical reactivity of **1** and showed that compounds undergo dehydration, delivering QMs, albeit inefficiently. Furthermore, QMs can be formed upon the excitation of low-energy chromophore naphthalimide, in a process that involves photoinduced electron transfer (PET), as has been demonstrated in a similar system by Freccero et al. [63]. The photophysical properties of **1** were investigated with steady-state and time-resolved fluorescence, whereas reactive intermediates in the photochemistry of **1** were detected through laser flash photolysis (LFP). The biological applicability of **1** was demonstrated by the binding study of **1** to calf thymus DNA (ct-DNA) and bovine serum albumin (BSA). The antiproliferative activity for **1** was investigated on human cancer carcinoma cells, with or without exposure to irradiation, showing an enhancement of the effect upon irradiation.

## 2. Results and Discussion

### 2.1. Synthesis

**Scheme 2:**
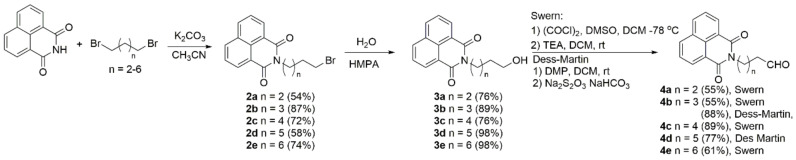
Synthesis of naphthalimide precursors **4**.

The synthesis of naphthol-naphthalimide conjugates is based on a Grignard reaction as the key step in connecting two molecular fragments. The key intermediates, 1,8-naphthalimide derivatives **4**, for the coupling, were prepared in three steps (Scheme 2) in good to excellent yields. These steps involved the alkylation of the imide by dibromides to afford **2** [64], followed by hydrolysis by HMPA and H_2_O to alcohols **3** [65], and oxidation to furnish **4**. We tried two oxidation protocols, those of Swern [66] and Dess-Martin [67], both of which produced **4** in good yields. However, the procedure of Dess-Martin is a more elegant procedure, allowing for easier isolation of the products.

**Scheme 3:**
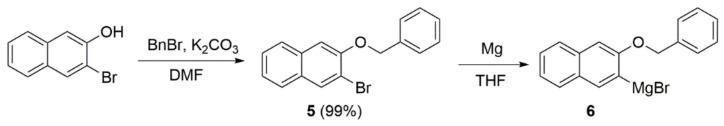
Preparation of naphthol Grignard reagent **6 [30]**.

Grignard reagent **6** for the coupling was prepared in two steps (Scheme 3), according to the known procedure from 3-bromo-2-naphthol, which was protected by a benzyl group and then transformed to the organometallic reagent via treatment with Mg [30].

**Scheme 4:**
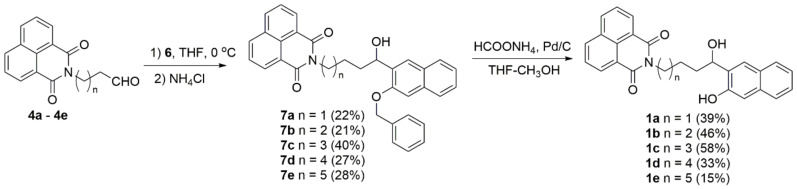
Synthesis of target naphthol-naphthalimides **1a**–**1e**.

Naphthalimide aldehydes **4** were treated with Grignard reagent **6**, and after hydrolysis, coupled products **7** were isolated in moderate yields. We tried several deprotection protocols, such as the usual hydrogenolysis, over Pd/C [30] or treatment with triethylsilane and Pd/C [68], or cyclohexene and Pd/C [69], which were unsuccessful due to a competing elimination of the benzylic alcohol. However, we encountered a hydrogen transfer procedure with ammonium formate on Pd/C [70] that gave the desired target molecules **1** in moderate yields (Scheme 4).

### 2.2. Photophysical Properties

**Figure 1:**
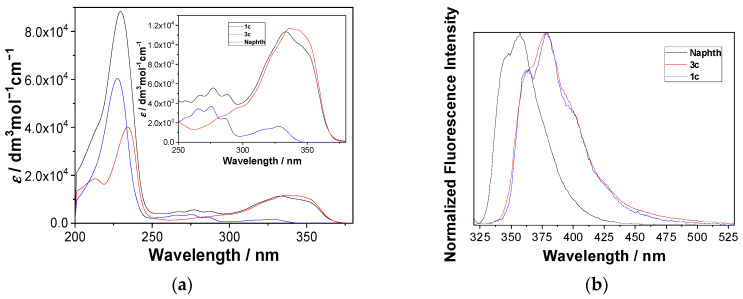
(**a**) Absorption spectra of Naphth, **3c** and **1c** in CH_3_OH-H_2_O (4:1 *v*/*v*), (**b**) normalized fluorescence spectra (*λ*_ex_ = 310 nm) of Naphth, **3c** and **1c** in CH_3_CN.

UV-vis and steady-state fluorescence measurements for **1** were conducted in CH_3_CN and CH_3_CN-H_2_O (4:1, *v/v*) solutions, where the difference was expected due to excited state proton transfer (ESPT) pathways taking place in the aqueous solution only [71,72,73,74], and because β-naphthols are known to undergo ESPT [75]. However, molecule **1** contains two chromophores, naphthol and naphthalimide. Figure 1 shows the absorption spectra of **1c**, together with the spectra of the corresponding naphthol (Naphth, Scheme 1) and naphthalimide alcohol **3c** (for UV-vis spectra of **1** see Figure S1). The absorption spectrum of **1c** corresponds to the sum of the absorption spectra of the two chromophores (Figure S2 in the ESI), indicating that the two chromophores do not interact in the ground state. On the contrary, the normalized emission spectra of Naphth, **3c** and **1c** (Figure 1, right), show that the emission from **1c** originates from the naphthalimide chromophore and not from the naphthol. The same behavior was observed for all derivatives, **1** (Figure S3). Since the emission spectrum of the naphthol overlaps with the absorption spectrum of the naphthalimide (Figure S4 in the ESI), upon excitation of the naphthol, an efficient Förster resonance energy transfer (FRET) to the naphthalimide takes place (vide infra).

Quantum yields of fluorescence (Φ_f_) were measured in CH_3_CN and CH_3_CN-H_2_O (4:1 *v/v*) with the use of quinine sulfate in 0.5 M H_2_SO_4_ as a reference (Φ_f_ = 0.546) [76] (Table 1). The Φ_f_ values are generally low, in accordance with the emission properties of 1,8-naphthalimides, which are known to undergo efficient intersystem crossing (Φ_ISC_ > 0.9) and populate triplet excited states [77]. The higher Φ_f_ measured in the aqueous solvent than in the neat CH_3_CN is also in accord with the photophysical properties of naphthalimides. Protic solvents form H-bonds with the imide chromophore, which induces bathochromic shifts in the fluorescence spectra. Furthermore, protic solvents perturb the energy levels of the n,π* and π,π* singlet and triplet excited states, and therefore affect the efficiency of the ISC [74]. One generally sees an increase in the Φ_f_ by increasing the alkyl linker length between the two chromophores. A mechanism that can involve depopulation of the imide singlet or triplet excited state is photoinduced electron transfer (PET) from the naphthol to the imide, since 1,8-naphthlimides are good oxidizing agents in the excited state [78,79,80].

Singlet excited state lifetimes for **1a**–**1e** in CH_3_CN were measured by means of time-correlated single photon counting (TC-SPC; for all data see Figures S5–S10 and Table S4 in the ESI). The samples were excited at 340 nm where almost only naphthalimide absorbs light, and the emission was detected at 390 nm. Fluorescence decays were fit to single exponential functions, giving lifetimes in the range 80–130 ps, at the limits of detection of the setup used. For some derivatives, fluorescence decay was best described as sum of two exponentials, with a small contribution of a longer decay component ascribed to the emission from the naphthol.

Normalized fluorescence spectra of **1a**–**1e** in CH_3_CN show the strong bands between 350 and 425 nm corresponding to the emission from the naphthalimide, and very weak shoulders at ≈340 nm corresponding to the emission from naphthol, quenched by FRET to the naphthalimide (Figure S4 in the ESI). To measure the efficiency of FRET, we performed TC-SPC measurements by exciting samples at 280 nm, where both naphthol and naphthalimide absorb light, and by collecting decays at 340 nm, where only naphthol emits. These decays were compared to that of Naphth in CH_3_CN. Fluorescence decays of **1a**–**1e** at 340 nm were all fit to a sum of three exponents, and the average decay time was significantly shorter compared to that of Naphth (τ = 9.07 ns), revealing the efficiency of FRET to be between 69% and 82% (Table 1). The three-exponential decays were explained by the conformational mobility of molecules **1a**–**1e**, resulting in a distribution of distances between the donor and the acceptor and different rates of FRET.

### 2.3. Photochemistry

Irradiation of Naphth in aqueous CH_3_OH leads to the dehydration and formation of QM, which reacts with nucleophiles, delivering the photomethanolysis product, methyl ether (Naphth-OMe Scheme 1) [36]. To probe for the methanolysis reaction with naphthol-naphthalimide conjugates, **1**, we performed irradiations in CH_3_OH-H_2_O (4:1 *v/v*), where the anticipated methyl ethers would indicate the formation of QMs as intermediates. Irradiations were performed at 254 nm, exciting mostly naphthol, or at 350 nm, where the naphthalimide was only excited. In both cases, photolyses were not efficient, 1 h irradiation gave photoproducts in 1–2% yield, detected using UPLC-MS. In addition to the anticipated ethers, some unidentified products were also formed. To characterize the anticipated ethers, they were prepared from **1** by means of H_2_SO_4_-catalyzed thermal methanolysis. By comparing UPLC-MS chromatograms of the synthesized ethers and irradiated mixtures, we have shown that the same ethers were formed in the photolyses. Preparative irradiations of **1a**, **1c** and **1e** were performed at 350 nm, since photolyses were more efficient than those conducted at 254 nm. After the irradiations, the mixtures were separated by means of UPLC-MS, and enriched fractions in photoproducts were analyzed using NMR and UPLC-MS. In addition to the anticipated ethers, we detected 1,8-naphthalimide, formed via the fragmentation of **1**.

The quantum yields of the photomethanolysis reaction were estimated upon excitation at 254 nm in CH_3_OH-H_2_O (4:1 *v/v*), which is a monochromatic irradiation source, and KI/KIO_3_ was used as an actinometer (Φ_R_ = 0.74) [81]. The results are compiled in Table 2. One generally sees very low values for Φ_R_, which are due to FRET from the naphthol to the naphthalimide. Such a result was not anticipated in the first instance, since it is known that photodehydration takes place in an ultrafast photochemical reaction [82], which in principle could be in competition with FRET.

### 2.4. Laser Flash Photolysis (LFP)

To detect intermediates in the photochemistry of naphthol-naphthalimides **1**, LFP was used. The samples were excited with a Nd:YAG laser at 266 or 355 nm. The measurements were performed in Ar- and O_2_-purged CH_3_CN solution, where O_2_ was expected to quench triplets and radicals, but not QMs (for all transient absorption spectra and the associated decay kinetics, see Figures S11–S16).

Upon excitation at 266 nm, in both Ar- and O_2_-purged solution, a strong transient was detected, with a maximum at 350 nm, with weaker absorption extending over the visible region. Thus, the transient absorption at 400 nm was fit to a sum of two exponents with lifetimes of *τ* = 2.6 ± 0.1 ns and *τ* = 23.1 ± 0.8 ns. The transients were tentatively assigned to the naphthol radical cation (*λ*_max_ = 360 and 460 nm) [83] and the naphthalimide radical anion (*λ*_max_ = 410 nm) [84], based on comparison with the published spectra in the preceding literature, and the known photochemistry of the phenol-imide conjugates [63]. The short lifetime was tentatively associated to the naphthol radical cation, prone to fast decay via deprotonation to a naphthoxyl radical. The species with longer lifetime probably corresponds to the naphthalimide radical anion, for which the main decay pathway is probably back electron transfer, taking place more slowly than protonation.

**Figure 2:**
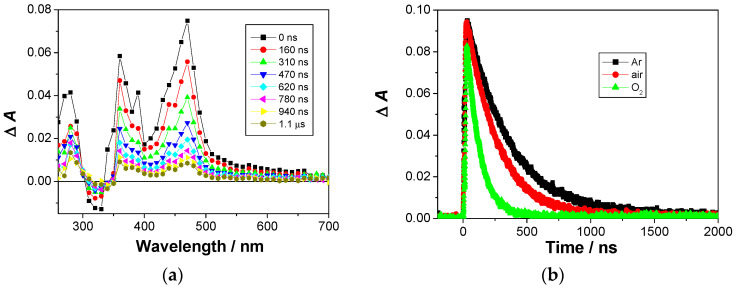
(**a**) Transient absorption spectra of N_2_-purged solution of **1a** in CH_3_CN (*c* = 7.23 × 10^−5^ M) The samples were excited at 355 nm, laser power ≈ 20 mJ/pulse. (**b**) Decay of transient absorbance at 470 nm in Ar-purged, not purged and O_2_-purged CH_3_CN solution.

LFP measurements allowed for the detection of additional weak transient absorption with a maximum at 470 nm. It decayed with unimolecular kinetics, with the following lifetimes: *τ*_Ar_ = 460 ± 20 ns; *τ*_air_ = 290 ± 20 ns and *τ*_O2_ = 100 ± 3 ns. Since the transient was quenched by O_2_, *k*_q_ = (8.7 ± 0.7) × 10^8^ M^−1^ s^−1^, and based on the comparison with literature [63,79], it was assigned to the naphthalimide triplet excited state. The naphthalimide triplet was formed using FRET from the naphthol, followed by ISC. Upon excitation at 355 nm, only one strong transient was detected, absorbing with a maximum at 470 nm (Figure 2), decaying with unimolecular kinetics with the lifetimes; *τ*_Ar_ = 340 ± 2 ns; *τ*_air_ = 245 ± 3 ns and *τ*_O2_ = 105 ± 2 ns. Based on the quenching by O_2_, *k*_q_ = (7.3 ± 0.3) × 10^8^ M^−1^ s^−1^; and its maximum of absorption [63,79], it was assigned to the naphthalimide triplet excited state. Note the much shorter decay time of the naphthalimide triplet in Ar-purged solution than the published value (*τ* = 0.25 ± 0.12 ms) [79], which can be explained by the intramolecular PET between the naphthol and the naphthalimide, although characterized with a slow process and the rate constant of *k =* 2.9 × 10^6^ s^−1^. Based on the proposed PET between the naphthol and the naphthalimide, the corresponding radical cation and the radical anion should have been detected. However, their fast decay and low absorptivity precluded their detection. Thus, after the decay of the triplet, due to the low intensity of the transient absorption signal, additional transients were not detected.

### 2.5. Photochemical Reaction Mechanism

**Scheme 5:**
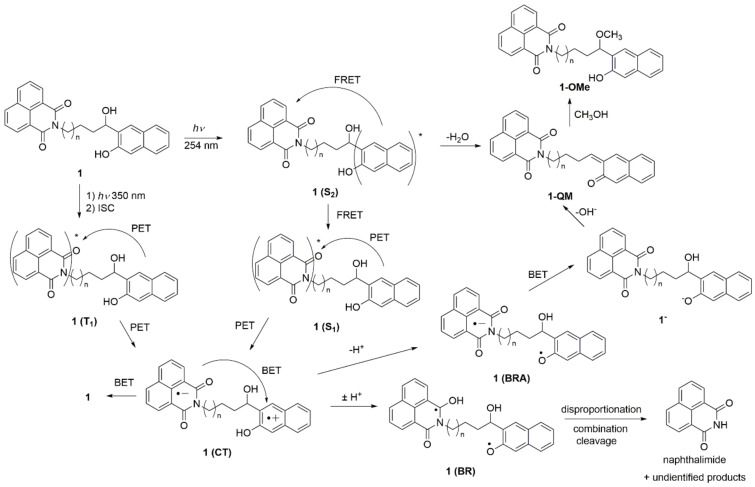
Plausible photochemical processes upon excitation at 254 nm or 350 nm.

A plausible mechanism for the formation of ethers, as well as for the cleavage photoproducts upon excitation at 350 nm, is shown in Scheme 5. The mechanism involves PET from the naphthol to the naphthalimide as the key step, which was indicated from the LFP measurements, and is in accordance with the mechanism proposed by Freccero et al. [63]. Thus, the formation of the QM and methanolysis product upon excitation at 254 nm may proceed via the dehydration mechanism proposed by Popik et al. [36]. However, due to efficient FRET from the naphthol to the naphthalimide, the methanolysis upon excitation at 254 nm is not efficient. On the other hand, excitation at 350 nm leads to naphthalimide S_1_, which, after an efficient ISC, leads to a naphthalimide triplet excited state. The naphthalimide triplet may undergo PET, whereupon the naphthol radical cation and the naphthalimide radical anion are formed (**1**(CT) in Scheme 5). Efficient back electron transfer (BET) gives **1** in the ground state, and it is probably the main deactivation pathway resulting in the low efficiency of the photochemical processes. In a protic solvent, the acidic radical cation is deprotonated to the phenoxyl radical **1**(BRA), whereas the naphthalimide radical anion is protonated to a ketyl radical **1**(BR). The formation of radicals most probably leads to combinations of disproportionation and cleavage reactions, whereupon naphthalimide is formed as a detectable product. Note that the formation of biradicals such as **1**(BR) may occur via photoinduced H-transfer reactions. Although such processes are not ubiquitous in the naphthalimide photochemistry [77], photochemical hydrogen abstractions are well documented for imide derivatives such as phthalimides [85]. Furthermore, as suggested by Freccero [63], the naphthalimide radical anion may be protonated before the BET, whereupon naphtholate **1** is formed. A subsequent loss of OH^−^ leads to the formation of QM. Note that the QM formation, in this case, was initiated by excitation of the lower-energy chromophore naphthalimide, at 350 nm. Furthermore, the formation of QMs takes place more efficiently upon excitation at 350 nm, although excitation of the naphthol at 254 nm is followed by FRET to the naphthalimide. However, the FRET is not 100% efficient.

The irradiation experiments and mechanistic investigations indicated the formation of QMs from **1**, which can induce biological effects, but the reaction is inefficient. However, photolysis of **1** gives rise to a plethora of other reactive intermediates, such as free radicals that can engage in reactions with biomacromolecules and induce cell damage. Most importantly, upon excitation at 350 nm, the naphthalimide triplet excited state is populated, which is quenched by O_2_, giving rise to singlet O_2_ and increased ROS, which is probably the main species responsible for the cytotoxic effect.

### 2.6. Noncovalent Binding to ct-DNA

Since naphthalimide derivatives are known to bind to polynucleotides [50,51], we investigated the non-covalent binding of **1** with naturally occurring calf thymus DNA (ct-DNA), characterized by the typical B-helical secondary structure and the almost equimolar ratio of AT- and GC-basepairs. The effect of binding was investigated via thermal denaturation experiments, whereas binding constants were determined by fluorescence titration. To probe for the binding mode (intercalation vs groove binding), we conducted CD measurements. Due to low solubility in H_2_O, stock solutions of **1** were prepared in DMSO and diluted with cacodylate buffer solutions.

#### 2.6.1. Thermal Denaturation

The binding of small molecules to double stranded (ds) polynucleotides affects the stability of the ds-helix, usually by stabilizing its structure. Thus, thermal denaturation and unwinding of the ds-helix into the single stranded (ss) form takes place at a higher or lower temperature, known as the melting temperature (*T*_m_) [86]. The difference between the *T*_m_ value of a free ds-polynucleotide and a complex with a small molecule (Δ*T*_m_ value) provides information about the interaction between the small molecule and the ds-polynucleotides. Moderate to strong stabilization (Δ*T*_m_ > 5 °C) supports the intercalative or minor groove binding interaction [87], whereas weak stabilization (Δ*T*_m_ = 0–5 °C) suggests a binding process driven by hydrophobic effects, accompanied by weak H-bonding or electrostatic interactions.

The thermal denaturation experiments were conducted with **1a** and **1f** and ct-DNA (Tables S5 and S6 and Figures S17 and S18 in the ESI). However, the compounds in the concentration corresponding to the ratio *r =* (**1**)/(ct-DNA) = 0.3 did not affect the thermal stability of the helices, suggesting non-specific binding along the DNA double helix.

#### 2.6.2. UV-Vis and Fluorescence Titrations

**Figure 3:**
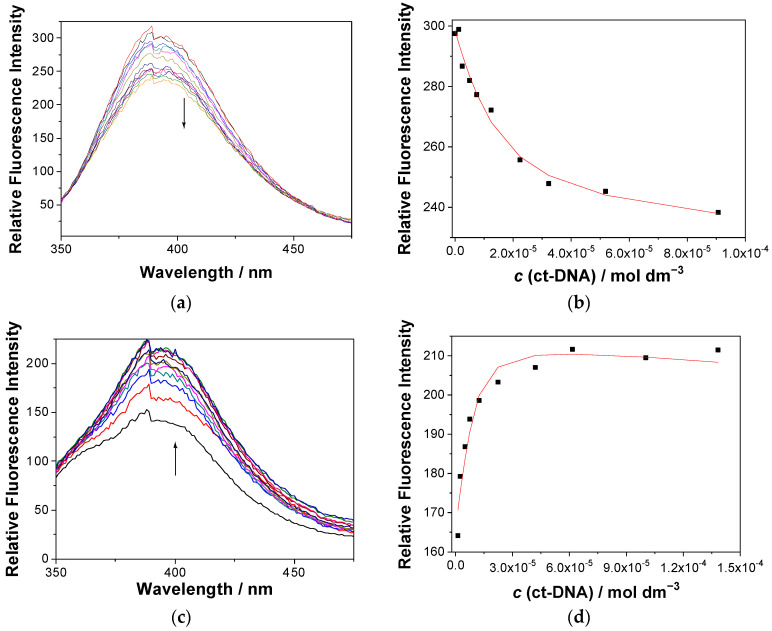
(**a**,**c**) Fluorescence spectra (*λ*_exc_ = 320 nm) of **1a** (**top**) and **1e** (**bottom**) (*c* = 2.0 × 10^−6^ M) at different concentrations of ct-DNA. (**b**,**d**) Dependence of the fluorescence intensity at 390 nm for the solution of **1a** (**a**,**b**) and **1e** (**c**,**d**); the black dots are experimental values, whereas the red line is a fitted value according to the Scatchard model [88]. The measurements were performed in Na cacodylate buffer (pH 7.0, 50 mM at 25 °C, containing <1% DMSO).

The binding of **1** to ct-DNA was assayed via UV-vis and fluorescence titrations in cacodylate buffer at pH 7. However, UV-vis titrations did not show applicability in determining the association constants due to the low solubility of molecules **1** in the aqueous cacodylate buffer (containing less than 1% DMSO). In the measurement conducted with the use of a 5 cm pathlength probe, allowing titrations at lower concentrations of **1** and DNA, **1a** precipitated immediately, whereas **1e** precipitated after the addition of ct-DNA at the ratio *r =* (**1**)/(ct-DNA) = 0.5, precluding estimation of the constant. However, even three additions of DNA at *r* = 2–0.5 revealed a weak hypochromic effect at λ < 370 nm, and a hyperchromic effect at λ > 370 nm (Figure S19 in the ESI), suggesting the formation of a complex. Fortunately, fluorescence of **1** allowed fluorescence titrations at much lower concentrations. The addition of polynucleotide to the solution generally induced weak emission changes in **1**, allowing the estimation of the binding constants (*K*_a_) only. The samples were excited at 320 nm, where the hypochromic effect in UV spectrum was negligible (<7%). The titration with ct-DNA induced weak emission quenching of **1a** (Figure 3, top) but an increase in the emission of **1e** (Figure 3, bottom).

The quenching of fluorescence by ct-DNA may be rationalized by PET between the naphthalimide and guanine, which is the nucleobase with the lowest oxidation potential [59,60,89], and in principle, it can be static (due to the formation of a nonfluorescent complex) or dynamic (due to diffusion). To check if the quenching is static, we performed TC-SPC measurement for **1a** and a mixture of **1a** and ct-DNA, in the same concentrations as in the fluorescence titration experiments (see Figures S20–S21 and Table S7 in the ESI). The same decay time in the presence of ct-DNA would indicate the static quenching. However, the short decay times at the limits of the detection of the setup used precluded their precise determination. Thus, although shortening of the main decay component from 210 to 170 ps was observed, these values are within experimental error. Consequently, we cannot completely rule out a dynamic quenching mechanism, but the short decay times and quenching in the concentration of DNA at 10^−4^ M is in favor of the static quenching mechanism. Namely, the Stern–Volmer analysis of the quenching by DNA would provide the quenching constant *k*_q_ in the order of 10^12^ M^−1^ s^−1^, which is higher than the diffusion limit.

Non-linear fitting of the fluorescence dependence on the ct-DNA concentration using the Scatchard model [88] gave a binding constant log*K*_a_ = 5.8, for the fixed value of the Scatchard ratio n[**1a**]/[ct-DNA] = 0.2. On the contrary, the addition of polynucleotide to the solution of **1e** resulted in an increase in the fluorescence (Figure 3, bottom), further supporting the possibility that the changes in fluorescence are not due to dynamic quenching. A tentative explanation for the fluorescence enhancement is the aggregation of the larger molecule **1e** in the aqueous medium, yielding a lower intensity in respect to **1a** (Figure 3, see starting intensities of free dyes). Thus, upon the addition of the DNA, de-aggregation of **1e** takes place, resulting in the fluorescence increase. The association constant log*K*_a_ = 6.3 (fixed value n = 0.2) was estimated through the non-linear fitting of data to the Scatchard model [88].

#### 2.6.3. CD Experiments

Although the thermal denaturation experiments indicated that derivatives **1** do not intercalate or bind to grooves of ct-DNA, binding was further investigated by means of circular dichroism (CD) spectroscopy. Namely, CD spectroscopy is a powerful analytical tool for the binding studies of small molecules to chiral macromolecules such as DNA [90], giving rise to the distinctive spectral differences for intercalators and groove binding derivatives [91,92]. Compounds **1** are chiral, but they are present in a racemic mixture, and therefore they do not have measurable CD spectra. However, upon their addition to the solution of ct-DNA, they can affect the helical chirality of DNA, or if they form complexes with the characteristic organization of the chromophores along the chiral axes of DNA, an induced CD signal can show up in the region where molecules **1** absorb light [91,92].

The addition of **1a** or **1e** to the solution of ct-DNA induced only small changes in the DNA CD signal, decreasing the intensity of the positive band at 275 nm, and that of the negative band at 245 nm (Figures S22 and S23 in the ESI). The lack of measurable ICD bands >300 nm suggests a non-uniform orientation of the chromophores of **1e** in respect to the DNA chiral axis, which is in agreement with the non-specific binding of the small molecule along DNA double helix.

The binding studies with ct-DNA have shown that compounds **1a** and **1e** form complexes with polynucleotides with moderate binding constants. However, the negligible effect of **1a** and **1e** in the thermal denaturation of ct-DNA, as well as the absence of ICD bands in the CD experiments, suggest that the studied naphthol-naphthalimides bind to ds-DNA in a nonspecific way, most probably by agglomeration along the double helix, caused by hydrophobic effects, accompanied potentially by weak H-bonding interactions.

### 2.7. Noncovalent and Covalent Binding to BSA Protein

Naphthol naphthalimide derivatives are non-charged species and not very polar, being good substrates for noncovalent binding to serum albumin proteins. We took advantage of the intrinsic fluorescence of **1a** and **1e** to study their binding to BSA, presuming that the insertion of small molecules into a BSA binding site would change the hydration of chromophores, consequently yielding a measurable change in their emission. The dynamic quenching mechanism was again dismissed due to the short singlet excited state lifetimes of **1a**–**1e** and the low concentration range of protein, which affected the fluorescence.

**Figure 4:**
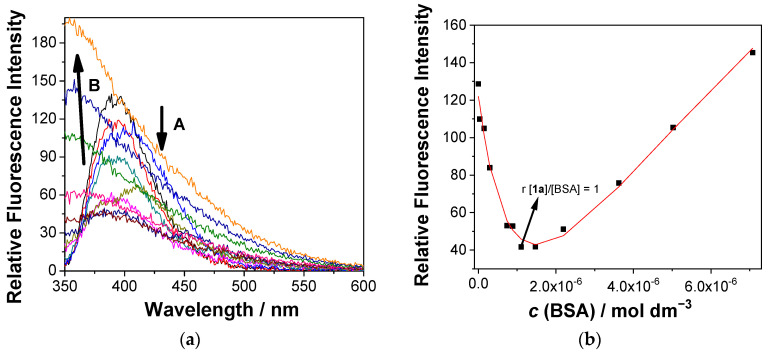
(**a**) Fluorescence spectra (*λ*_exc_ = 320 nm) of **1a** (*c* = 1.0 × 10^−6^ M) in the presence of different concentrations of BSA. (**b**) Dependence of the fluorescence intensity at 390 nm on the BSA concentration. The black dots are experimental values and the red line is the fit to the model, involving **1a@BSA** in the stoichiometric ratios of 1:1 and 2:1. The measurement was conducted in aqueous solution, containing 0.05 M sodium cacodylate buffer at pH = 7.0, at 25 °C (containing <1% DMSO).

Accordingly, upon the addition of BSA, the fluorescence of **1a** was quenched until a ratio of 1:1 between **1a** and BSA was reached (Figure 4, arrow A). Further additions of BSA induced a fluorescence increase in **1a** and a considerable broadening of the emission spectrum, accompanied by a significant hypsochromic shift of the emission maximum (Figure 4, arrow B). Such opposite changes clearly indicate the formation of two types of complexes. The fluorescence quenching upon the addition of BSA to **1a** is assigned to the aggregation of two molecules within the BSA binding site, whereas further additions of BSA allowed each molecule of **1a** to bind to an independent binding site, thus resulting in the formation of a 1:1 complex (denoted as **1a@BSA**) and a consequent fluorescence increase. Multicomponent nonlinear regression analysis of the fluorescence data using HypSpec2014 software [93] gave the best fit to the formation of **1a@BSA** complexes in the stoichiometries 2:1 and 1:1, with the association constants logβ (**1a**_2_**@BSA**) = 11.38 ± 0.04 and logβ (**1a@BSA**) = 5.24 ± 0.05, respectively.

Contrary to **1a**, the addition of BSA to the solution of **1e** led to a fluorescence increase only (Figure S23). Nonlinear regression analysis using HypSpec2014 software revealed the formation of one complex with the stoichiometric ratio of 1:1 and the association constant log*K*_a_ (**1e@BSA**) = 5.05 ± 0.05. It should be noted that **1a** and **1e** form 1:1 complexes with BSA with similar binding constants, but only the smallest **1a** forms a 2:1 complex within a BSA binding site.

Our preliminary binding experiments with BSA undoubtedly indicate the formation of complexes **1a**@BSA and **1e@BSA**. However, the exact binding site of **1** in the protein remained undetermined and is not in the scope of this report. To demonstrate that the irradiation of complexes of **1** with BSA leads to the alkylation of the protein, we performed irradiations of the **1a@BSA** and **1e@BSA** complexes. The aqueous buffered solutions of the complexes were irradiated at 350 nm, followed by desalting of the protein and analysis by means of MALDI-MS. Naphthol-naphthalimide with the shortest linker, **1a**, did not alkylate the protein, whereas the one with the longest linker, **1e**, covalently attached to BSA. The molecular weight of 67.1–67.2 kDa pointed to the addition of **1e** to the protein (see Figures S25–S27 and Table S8 in the ESI). It is plausible to assume that the photoinduced mechanism of protein alkylation involves QM intermediates, as seen in the prior literature [6,7], although the photodehydration to QMs from **1** is inefficient.

### 2.8. Antiproliferative Activity

We have demonstrated that compounds **1a**–**1e** bind to a model DNA and protein by means of noncovalent interactions, where, after photochemical excitation, they may induce damage. Therefore, we investigated the antiproliferative activity of compounds **1a**–**1e** on two human cancer cell lines, H-460 (lung) and MCF-7 (breast), with the cells kept in the dark, or irradiated at 350 nm (for all data, see Table S9 in the ESI). Control experiments were performed with a psoralene derivative (trioxsalen), which is known to induce photoactivable cross-linking [12,94] and Naphth (Scheme 1). The analysis of results in Table S9 showed decreasing cytotoxicity under dark conditions (expressed by IC_50_ values) proportional to the linker length between the two chromophores, with **1e** bearing the longest linker, being non-toxic for the H-460 cell line even at the highest tested concentration. However, the enhancement of the antiproliferative effect upon UV irradiation (Table S9, 350 nm) shows the opposite trend—the strongest effects were observed for the compounds with longer linkers (**1d**, **1e**). This result suggests that UV irradiation of **1e** inside the cell leads to the production of a higher amount of cytotoxic agent (possibly QMs, as well as singlet oxygen/reactive oxygen species (ROS)) in comparison to the smallest, **1a** (showing an order of magnitude weaker effect). Furthermore, compounds **1a**–**e** exhibit higher cytotoxicity than Naphth, indicating that the naphthalimide moiety is important for this activity. Although we do not have clear evidence as to which of the reactive species formed in the photochemical reactions led to the observed photoinduced antiproliferative effect, it is plausible that singlet O_2_ and subsequent ROS formed by the quenching of the naphthalimide triplet played an important role, as well as QMs and reactive radicals formed in the photoinduced processes. It should be noted that the investigated molecules cannot have therapeutic value, since irradiation with UV light was used. However, this proof of principle is important for the future rational design of photoactivable compounds with antiproliferative activity.

## 3. Conclusions

Naphthol-naphthalimide conjugates, QM precursors **1** undergo photodehydration to the corresponding QMs very inefficiently due to FRET from the naphthol to the naphthalimide. In addition to the elimination of H_2_O, QMs can also be formed upon excitation of the naphthalimide in a process that involves PET from the naphthol to the naphthalimide. The linker length variation between the naphthol and the naphthalimide in the **1a**–**1e** series mildly affects their photophysical properties and the photochemical reactivity, but had a much more obvious impact on bio-relevant properties, DNA-fluorimetric response, protein-binding stoichiometry and cytotoxicity on cell lines. The naphthol-naphthalimide conjugates showed moderate affinity toward ds-DNA (*K*_a_ ≈ 10^5^–10^6^ M^−1^), due to their non-specific agglomeration along the DNA helix. Comparison of the shortest (**1a**) and the longest (**1e**) derivative revealed opposite fluorimetric responses, attributed to **1e** aggregation in a free state and de-aggregation upon DNA binding. Furthermore, the studied compounds also efficiently bind to the BSA protein, whereby the shortest analogue, **1a**, can form dimers inside a BSA binding site, at variance to the longest, **1e**, which binds only as a monomer. Upon irradiation of the complex **1e@BSA**, photoinduced alkylation of the protein takes place, probably via the QM. Derivatives **1a**–**e** exhibit cytotoxic effects against cancer cell lines H-460 and MCF-7 that is reversely proportional to the linker length and correlated to the aggregation propensity of the longest derivative, **1e**. All compounds showed enhanced cytotoxicity upon exposure to irradiation at 350 nm, and this was particularly pronounced for the longest analogue, **1e**, which not only increased by two orders of magnitude in H-460 cells but also was an order of a magnitude stronger than the shortest analogue, **1a**. The enhancement of this activity probably stems from the formation of singlet oxygen (or ROS-related species) but the effect of QMs and reactive radicals formed in the photoinduced processes cannot be disregarded. The particularly promising theragnostic properties of **1e**, unifying photoinduced-bioactivity and fluorescence enhancement upon binding to biorelevant targets, encourage the further study of its close analogues.

## 4. Materials and Methods

General: ^1^H and ^13^C NMR spectra were recorded at 300, 400, 500 or 600 MHz at rt, using TMS as a reference, and chemical shifts were reported in ppm. Melting points were determined using a Mikroheiztisch apparatus and were not corrected. IR spectra were recorded on an ATR spectrometer and the characteristic peak values were given in cm^−1^. HRMS was carried out on a MALDI TOF/TOF instrument. Details on semipreparative MPLC and UPLC separations and analyses are reported in the ESI. Chemicals were purchased from the usual commercial sources and were used as received. Solvents for conducting moisture-sensitive reactions were further dried over sodium metal. Solvents for chromatographic separations were used as delivered from the supplier (p.a. or HPLC-grade). Preparations of known compounds **2**, **3**, **5** and **6** are described in the ESI. ct-DNA was dissolved in Na-cacodylate buffer, *I* = 0.05 mol dm^−3^, pH 7.0, and it was additionally sonicated and filtered through a 0.45-μm filter. The polynucleotide concentration was determined spectroscopically as the concentration per nucleotide/phosphate, using molar absorption coefficient for calf thymus DNA of 6600 M^−1^ cm^−1^ at 260 nm.

- **General procedure for the preparation of *N*-(ω-formylalkyl)-1,8-naphthalimide 4—Swern oxidation**

A dry two-neck round-bottom flask, equipped with a dropping funnel and a septum, under Ar inert atmosphere, was charged with oxalyl chloride (5 mmol) and dry CH_2_Cl_2_ (1 mL/mmol oxalyl chloride). The reaction mixture was cooled down to −78 °C, and a solution of DMSO (10 mmol) in dry CH_2_Cl_2_ (0.2 mL/mmol DMSO) was added dropwise. After 1 h of stirring at −78 °C to the reaction mixture, a solution of **3** (1 mmol) in dry CH_2_Cl_2_ (1 mL/mmol **3**) was added dropwise. The reaction mixture was stirred at −78 °C for 2 h. Dry triethylamine (15 mmol) was then added dropwise, and the reaction was stirred at −78 °C for 1 h and then was left to spontaneously reach rt over 17 h. The reaction mixture was transferred to a separation funnel, diluted with H_2_O and extracted with CH_2_Cl_2_ (3 × 20 mL). The combined organic layers were washed with saturated aqueous NH_4_Cl (3 × 20 mL) and brine (2 × 20 mL). The solution was dried over anhydrous Na_2_SO_4_, filtered and the solvent was removed on a rotary evaporator. The residue was purified on a silica gel column using EtOAc (0 to 50%)/cyclohexane as eluent to afford the pure product.

- **General procedure for the preparation of *N*-(ω-formylalkyl)-1,8-naphthalimide—Dess–Martin oxidation** [95]

A round-bottom flask, equipped with a dropping funnel, was charged with Dess–Martin periodinane (DMP, 1.2 mmol) and CH_2_Cl_2_ (2.5 mL/mmol DMP). A solution of **3** (1 mmol) in CH_2_Cl_2_ (2.5 mL/mmol **3**) was added dropwise at rt. The reaction mixture was stirred at rt for 1.5 h, and then a solution of Na_2_S_2_O_3_ (9.5 mmol) in a saturated aqueous solution of NaHCO_3_ (1 mL/mmol Na_2_S_2_O_3_) was added. The resulting suspension was stirred for 20 min after it was transferred to a separation funnel. The layers were separated and the aqueous layer was extracted with CH_2_Cl_2_ (3 × 20 mL). The combined organic layers were washed with saturated aqueous NaHCO_3_ (3 × 10 mL) and brine (2 × 10 mL). The solution was dried over anhydrous Na_2_SO_4_, filtered and the solvent was removed on a rotary evaporator. The residue was purified on a silica gel column using EtOAc (0 to 50%)/cyclohexane as eluent to afford the pure product.

*N*-(3-formylprop-1-yl)-1,8-naphthalimide (**4a**) was prepared according to the general procedure for Swern oxidation from alcohol **3a** (2.15 g, 7.9 mmol). The reaction after work-up and chromatography furnished 1.17 g (55%) of the pure product in the form of a colorless solid. The NMR characterization was in accordance with the data from the preceding literature [96].

mp 78–80 °C; IR (ATR) $\tilde{\nu }$/cm^−1^: 3063 (Ar C‒H), 2952, 2729 (C‒H), 2830 (C‒H ald.), 1698 (C=O, ald.), 1660 (C=O), 1586 (C–N amide), 1341 (C=C); ^1^H NMR (CDCl_3_, 400 MHz) *δ*/ppm: 9.81 (t, 1H, *J* = 1.4 Hz), 8.59 (dd, 2H, *J* = 7.3 Hz, *J* = 1.1 Hz), 8.22 (dd, 2H, *J* = 8.3 Hz, *J* = 1.0 Hz), 7.76 (dd, 2H, *J* = 8.2 Hz, *J* = 7.2 Hz), 4.25 (t, 2H, *J* = 7.0 Hz), 2.59 (td, 2H, *J* = 7.3 Hz, *J* = 1.4 Hz), 2.11 (quin, 2H, *J* = 7.2 Hz); ^13^C NMR (CDCl_3_, 100 MHz) *δ*/ppm: 201.4 (d), 164.2 (s, 2C), 134.0 (d, 2C), 131.5 (s), 131.3 (d, 2C), 128.1 (s), 126.9 (d, 2C), 122.5 (s, 2C), 41.3 (t), 39.4 (t), 20.7 (t); UPLC-MS/UV: method ➁, *t*_R_ = 0.96 min, *m*/*z* = 268.10 [M + H]^+^, found 268.14; HRMS (MALDI TOF/TOF): calculated for C_16_H_13_NO_3_ [M + H]^+^ 268.0974; found 268.0984.

*N*-(4-formylbut-1-yl)-1,8-naphthalimide (**4b**) was prepared according to the general procedure for Swern oxidation from alcohol **3b** (1.93 g, 6.8 mmol). The reaction after work-up and chromatography furnished 1.22 g (55%) of the pure product in the form of a colorless solid.

The Dess–Martin oxidation method with alcohol **3b** (4.86 g, 17.2 mmol) with DMP (8.73 g, 20.6 mmol) after the work-up and chromatography gave 4.25 g (88%) of the pure product in the form of a colorless solid.

mp 94–95 °C; IR (ATR) $\tilde{\nu }$/cm^−1^: 2942, 2732 (C‒H), 2831 (C‒H ald.), 1690 (C=O, ald.), 1656 (C=O), 1586 (C–N amide), 1339 (C=C); ^1^H NMR (CDCl_3_, 300 MHz) *δ*/ppm: 9.79 (t, 1H, *J* = 1.6 Hz), 8.60 (dd, 2H, *J* = 7.3 Hz, *J* = 1.0 Hz), 8.22 (dd, 2H, *J* = 8.3 Hz, *J* = 0.9 Hz), 7.76 (dd, 2H, *J* = 8.2 Hz, *J* = 7.3 Hz), 4.22 (t, 2H, *J* = 6.9 Hz), 2.59–2.48 (m, 2H), 1.88–1.70 (m, 4H); ^13^C NMR (CDCl_3_, 100 MHz) *δ*/ppm: 202.2 (d), 164.2 (s, 2C), 133.9 (d, 2C), 131.5 (s), 131.2 (d, 2C), 128.1 (s), 126.9 (d, 2C), 122.5 (s, 2C), 43.4 (t), 39.7 (t), 27.5 (t), 19.5 (t); UPLC-MS/UV: method ➁, *t*_R_ = 1.03 min, *m*/*z* = 282.11 [M + H]^+^, found 282.10; HRMS (MALDI TOF/TOF): calculated for C_17_H_15_NO_3_ [M + H]^+^ 282.1130; found 282.1140.

*N*-(5-formylpent-1-yl)-1,8-naphthalimide (**4c**) was prepared according to the general procedure for Swern oxidation from alcohol **3c** (5.24 g, 17.6 mmol). The reaction after work-up and chromatography furnished 4.65 g (89%) of the pure product in the form of a colorless solid.

mp 76–77 °C; IR (ATR) $\tilde{\nu }$/cm^−1^: 3060 (Ar C‒H), 2927, 2851 (C‒H), 2699 (C‒H ald.), 1690 (C=O, ald.), 1649 (C=O), 1584 (C–N amide), 1343 (C=C); ^1^H NMR (CDCl_3_, 400 MHz) *δ*/ppm: 9.76 (t, 1H, *J* = 1.8 Hz), 8.59 (dd, 2H, *J* = 7.3 Hz, *J* =1.0 Hz), 8.20 (dd, 2H, *J* = 8.3 Hz, *J* = 1.0 Hz), 7.74 (dd, 2H, *J* = 8.2 Hz, *J* = 7.3 Hz), 4.18 (t, 2H, *J* = 7.5 Hz), 2.45 (td, 2H, *J* = 7.3 Hz, *J* = 1.7 Hz), 1.82–1.66 (m, 4H), 1.52–1.42 (m, 2H); ^13^C NMR (CDCl_3_, 100 MHz) *δ*/ppm: 202.5 (d), 164.1 (s, 2C), 133.9 (d, 2C), 131.5 (s), 131.2 (d, 2C), 128.1 (s), 126.9 (d, 2C), 122.6 (s, 2C), 43.7 (t), 40.0 (t), 27.7 (t), 26.5 (t), 21.5 (t); UPLC-MS/UV: method ➁, *t*_R_ = 1.11 min, *m*/*z* = 296.13 [M + H]^+^, found 296.16; HRMS (MALDI TOF/TOF): calculated for C_18_H_17_NO_3_ [M + H]^+^ 296.1287; found 296.1291.

*N*-(6-formylhex-1-yl)-1,8-naphthalimide (**4d**) was prepared according to the general procedure for Dess–Martin oxidation from alcohol **3d** (3.68 g, 11.8 mmol) and DMP (6.02 g, 14.2 mmol). The reaction after work-up and chromatography furnished 2.82 g (77%) of the pure product in the form of a colorless solid.

mp 83–84 °C; IR (ATR) $\tilde{\nu }$/cm^−1^: 2931, 2857 (C‒H), 2710 (C‒H ald.), 1693 (C=O, ald.), 1657 (C=O), 1586 (C–N amide), 1343 (C=C); ^1^H NMR (CDCl_3_, 400 MHz) *δ*/ppm: 9.75 (t, 1H, *J* = 1.9 Hz), 8.59 (dd, 2H, *J* = 7.4 Hz, *J* = 0.9 Hz), 8.20 (dd, 2H, *J* = 8.2 Hz, *J* = 0.9 Hz), 7.74 (dd, 2H, *J* = 8.2 Hz, *J* = 7.3 Hz), 4.17 (t, 2H, *J* = 7.4 Hz), 2.43 (td, 2H, *J* = 7.3 Hz, *J* = 1.8 Hz), 1.75 (quin, 2H, *J* = 7.4 Hz), 1.65 (quin, 2H, *J* = 7.3 Hz), 1.50–1.36 (m, 4H); ^13^C NMR (CDCl_3_, 100 MHz) *δ*/ppm: 202.7 (d), 164.1 (s, 2C), 133.8 (d, 2C), 131.5 (s), 131.1 (d, 2C), 128.1 (s), 126.9 (d, 2C), 122.6 (s, 2C), 43.7 (t), 40.2 (t), 28.8 (t), 27.8 (t), 26.8 (t), 21.9 (t); UPLC-MS/UV: method ➁, *t*_R_ = 1.19 min, *m*/*z* = 310.14 [M + H]^+^, found 310.18; HRMS (MALDI TOF/TOF): calculated for C_19_H_19_NO_3_ [M + H]^+^ 310.1443; found 310.1438.

*N*-(7-formylhept-1-yl)-1,8-naphthalimide (**4e**) was prepared according to the general procedure for Swern oxidation from alcohol **3e** (5.85 g, 17.9 mmol). The reaction after work-up and chromatography furnished 3.57 g (61%) of the pure product in the form of a colorless solid.

mp 71–73 °C; IR (ATR) $\tilde{\nu }$/cm^−1^: 2928, 2727 (C‒H), 2850 (C‒H ald.), 1695 (C=O, ald.), 1655 (C=O), 1590 (C–N amide), 1342 (C=C); ^1^H NMR (CDCl_3_, 400 MHz) *δ*/ppm: 9.75 (t, 1H, *J* = 1.8 Hz), 8.59 (dd, 2H, *J* = 7.2 Hz, *J* = 0.8 Hz), 8.20 (dd, 2H, *J* = 8.2 Hz, *J* = 0.7 Hz), 7.74 (t, 2H, *J* = 7.8 Hz), 4.17 (t, 2H, *J* = 7.5 Hz), 2.41 (td, 2H, *J* = 7.3 Hz, *J* = 1.7 Hz), 1.73 (quin, 2H, *J* = 7.4 Hz), 1.62 (quin, 2H, *J* = 7.3Hz), 1.49–1.28 (m, 6H); ^13^C NMR (CDCl_3_, 100 MHz) *δ*/ppm: 202.8 (d), 164.1 (s, 2C), 133.8 (d, 2C), 131.5 (s), 131.1 (d, 2C), 128.1 (s), 126.9 (d, 2C), 122.7 (s, 2C), 43.8 (t), 40.3 (t), 29.0 (t, 2C), 27.9 (t), 26.8 (t), 22.0 (t); UPLC-MS/UV: method ➁, *t*_R_ = 1.26 min, *m*/*z* = 324.16 [M + H]^+^, found 324.20; HRMS (MALDI TOF/TOF): calculated for C_20_H_21_NO_3_ [M + H]^+^ 324.1600; found 324.1598.

- **Grignard reaction of *N*-(ω-formylalkyl)-1,8-naphthalimide 4 and 6—general procedure**

A well-dried round-bottom flask equipped with a septum under Ar inert atmosphere was charged with *N*-(ω-formylalkyl)-1,8-naphthalimide (**4**, 1.0 mmol) and anhydrous THF (7 mL). The solution was cooled to 0 °C, and a solution of Grignard reagent **6** (1.1 mmol) was added dropwise via a syringe. The reaction mixture was stirred at 0 °C for 30 min, and then it was allowed to reach rt over 5 h. To the reaction mixture, a saturated aqueous solution of NH_4_Cl (10 mL) was added, and after 15 min of stirring, the mixture was transferred to a separation funnel. Extraction with EtOAc (3 × 20 mL) was carried out. The combined organic layers were dried over anhydrous MgSO_4_ and filtered and the solvent was removed on a rotary evaporator. The residue was purified on a silica gel column using EtOAc (0 to 30%)/cyclohexane as eluent to afford the pure product.

*N*-{4-[3-(benzyloxy)naphthalen-2-yl]-4-hydroxybut-1-yl}-1,8-naphthalimide (**7a**). Prepared according to the general procedure from aldehyde **4a** (920 mg, 3.44 mmol) and the Grignard reagent, **6**. After chromatography, the reaction furnished 385 mg (22%) of the pure product in the form of a pale yellow solid.

mp 156–157 °C; IR (ATR) $\tilde{\nu }$/cm^−1^: 3031 (Ar C‒H), 2954, 2923 (C‒H), 1655 (C=O), 1591 (C–N amide), 1341 (C=C), 1254 (C–O), 1061 (C–O–C); ^1^H NMR (CDCl_3_, 400 MHz) *δ*/ppm: 8.57 (d, 2H, *J* = 7.3 Hz), 8.19 (d, 2H, *J* = 8.2 Hz), 7.81 (s, 1H), 7.78–7.69 (m, 3H), 7.67 (dd, 1H, *J* = 8.2 Hz, *J* = 0.3 Hz), 7.49–7.42 (m, 2H), 7.42–7.35 (m, 3H), 7.35–7.27 (m, 2H), 7.17 (s, 1H), 5.19 (s, 2H), 5.15 (q, 1H, *J* = 6.0 Hz), 4.25 (t, 2H, *J* = 7.2 Hz), 2.85 (d, 1H, *J* = 6.7 Hz), 2.14–1.78 (m, 4H); ^13^C NMR (CDCl_3_, 100 MHz) *δ*/ppm: 164.2 (s, 2C), 154.3 (s), 136.5 (s), 133.8 (d, 2C), 133.6 (s), 131.5 (s), 131.2 (d, 2C), 128.79 (s), 128.73 (d, 2C), 128.1 (s), 128.0 (d), 127.7 (d), 127.3 (d, 2C), 126.9 (d, 2C), 126.3 (d), 126.2 (d), 126.1 (d), 123.9 (d), 122.6 (s, 2C), 106.6 (d), 71.2 (d), 70.0 (t), 40.1 (t), 34.6 (t), 24.8 (t), a signal of one quaternary C-atom was not observed; UPLC-MS/UV: method ➁, *t*_R_ = 1.42 min, *m*/*z* = 484.19 [M − OH]^+^, found 484.15; HRMS (MALDI TOF/TOF): calculated for C_33_H_27_NO_4_ [M + Na]^+^ 524.1838; found 524.1852.

*N*-{4-[3-(benzyloxy)naphthalen-2-yl]-4-hydroxypent-1-yl}-1,8-naphthalimide (**7b**) was prepared according to the general procedure from aldehyde **4b** (968 mg, 3.44 mmol) and Grignard reagent **6**. After chromatography, the reaction furnished 381 mg (21%) of the pure product in the form of a pale yellow solid.

mp 155–157 °C; IR (ATR) $\tilde{\nu }$/cm^−1^: 3047 (Ar C‒H), 2955, 2925, 2854 (C‒H), 1649 (C=O), 1591 (C–N amide), 1340 (C=C), 1237 (C–O), 1091 (C–O–C); ^1^H NMR (CDCl_3_, 400 MHz) *δ*/ppm: 8.58 (dd, 2H, *J* = 7.3 Hz, *J* = 0.9 Hz), 8.20 (dd, 2H, *J* = 8.3 Hz, *J* = 0.8 Hz), 7.81 (s, 1H), 7.79–7.66 (m, 4H), 7.49–7.43 (m, 2H), 7.43–7.30 (m, 4H), 7.25–7.18 (m, 2H), 5.21 (s, 2H), 5.10 (q, 1H, *J* = 6.2 Hz), 4.16 (t, 2H, *J* = 7.5 Hz), 2.60 (d, 1H, *J* = 6.2 Hz), 2.06–1.88 (m, 2H), 1.84–1.70 (m, 2H), 1.69–1.58 (m, 1H), 1.56–1.44 (m, 1H); ^13^C NMR (CDCl_3_, 100 MHz) *δ*/ppm: 164.1 (s, 2C), 154.4 (s), 136.5 (s), 134.2 (s), 133.8 (d, 2C), 133.5 (s), 131.5 (s), 131.2 (d, 2C), 128.8 (s), 128.6 (d, 2C), 128.1 (s), 128.0 (d), 127.7 (d), 127.4 (d, 2C), 126.9 (d, 2C), 126.3 (d), 126.1 (d), 126.0 (d), 123.9 (d), 122.7 (s, 2C), 106.6 (d), 70.9 (d), 70.1 (t), 40.2 (t), 37.1 (t), 27.8 (t), 23.5 (t); UPLC-MS/UV: method ➁, *t*_R_ = 1.45 min, *m*/*z* = 498.21 [M − OH]^+^, found 498.25; HRMS (MALDI TOF/TOF): calculated for C_34_H_29_NO_4_ [M + Na]^+^ 538.1994; found 538.1970.

*N*-{4-[3-(benzyloxy)naphthalen-2-yl]-4-hydroxyhex-1-yl}-1,8-naphthalimide (**7c**) was prepared according to the general procedure from aldehyde **4c** (930 mg, 3.15 mmol) and Grignard reagent **6**. After chromatography the reaction furnished 662 mg (40%) of the pure product in the form of a pale yellow solid.

mp 146–149 °C; IR (ATR) $\tilde{\nu }$/cm^−1^: 3053, 3027 (Ar C‒H), 2926, 2858 (C‒H), 1648 (C=O), 1590 (C–N amid), 1345 (C=C), 1239 (C–O), 1074 (C–O–C); ^1^H NMR (CDCl_3_, 400 MHz) *δ*/ppm: 8.58 (dd, 2H, *J* = 7.2 Hz, *J* = 0.7 Hz), 8.19 (dd, 2H, *J* = 8.3 Hz, *J* = 0.6 Hz), 7.82–7.66 (m, 5H), 7.48–7.27 (m, 7H), 7.19 (s, 1H), 5.20 (s, 2H), 5.11–5.02 (m, 1H), 4.20–4.10 (m, 2H), 2.00–1.82 (m, 2H), 1.79–1.67 (m, 2H), 1.65–1.43 (m, 4H); ^13^C NMR (CDCl_3_, 100 MHz) *δ*/ppm: 164.1 (s, 2C), 154.4 (s), 136.5 (s), 134.2 (s), 133.8 (d, 2C), 133.5 (s), 131.5 (s), 131.2 (d, 2C), 128.8 (s), 128.6 (d, 2C), 128.1 (s), 128.0 (d), 127.7 (d), 127.3 (d, 2C), 126.9 (d, 2C), 126.3 (d), 126.1 (d), 126.0 (d), 123.9 (d), 122.7 (s, 2C), 106.5 (d), 70.9 (d), 70.0 (t), 40.3 (t), 37.3 (t), 28.0 (t), 26.9 (t), 25.7 (t); UPLC-MS/UV: method ➁, *t*_R_ = 1.50 min, *m*/*z* = 512.22 [M − OH]^+^, found 512.69; HRMS (MALDI TOF/TOF): calculated for C_35_H_31_NO_4_ [M + Na]^+^ 552.2151 found 552.2131.

*N*-{4-[3-(benzyloxy)naphthalen-2-yl]-4-hydroxyhept-1-yl}-1,8-naphthalimide (**7d**) was prepared according to the general procedure from aldehyde **4d** (1064 mg, 3.44 mmol) and Grignard reagent **6**. After chromatography the reaction furnished 508 mg (27%) of the pure product in the form of a pale yellow solid.

mp 122–123 °C; IR (ATR) $\tilde{\nu }$/cm^−1^: 3048, 3023 (Ar C‒H), 2938, 2925, 2851 (C‒H), 1645 (C=O), 1588 (C–N amide), 1347 (C=C), 1237 (C–O), 1054 (C–O–C); ^1^H NMR (CDCl_3_, 400 MHz) *δ*/ppm: 8.58 (dd, 2H, *J* = 7.2 Hz, *J* = 0.9 Hz), 8.19 (dd, 2H, *J* = 8.3 Hz, *J* = 0.7 Hz), 7.81–7.66 (m, 5H), 7.49–7.36 (m, 5H), 7.36–7.29 (m, 2H) 7.20 (s, 1H), 5.21 (s, 2H), 5.10–5.00 (m, 1H), 4.15 (t, 2H, *J* = 7.6 Hz), 2.59 (d, 1H, *J* = 6.1 Hz), 1.97–1.79 (m, 2H), 1.77–1.62 (m, 2H), 1.56–1.33 (m, 6H); ^13^C NMR (CDCl_3_, 100 MHz) *δ*/ppm: 164.1 (s, 2C), 154.4 (s), 136.5 (s), 134.3 (s), 133.8 (d, 2C), 133.5 (s), 131.5 (s), 131.2 (d, 2C), 128.8 (s), 128.6 (d, 2C), 128.1 (s), 128.0 (d), 127.7 (d), 127.4 (d, 2C), 126.9 (d, 2C), 126.3 (d), 126.1 (d), 126.0 (d), 123.9 (d), 122.7 (s, 2C), 106.6 (d), 71.1 (d), 70.1 (t), 40.3 (t), 37.4 (t), 29.1 (t), 28.0 (t), 27.0 (t), 25.9 (t); UPLC-MS/UV: method ➁, *t*_R_ = 1.55 min, *m*/*z* = 526.24 [M − OH]^+^, found 526.16; HRMS (MALDI TOF/TOF): calculated for C_36_H_33_NO_4_ [M + Na]^+^ 566.2307; found 566.2295.

*N*-{4-[3-(benzyloxy)naphthalen-2-yl]-4-hydroxyoct-1-yl}-1,8-naphthalimide (**7e**) was prepared according to the general procedure from aldehyde **4e** (1113 mg, 3.44 mmol) and Grignard reagent **6**. After chromatography the reaction furnished 542 mg (28%) of the pure product in the form of a pale yellow solid.

mp 111–112 °C; IR (ATR) $\tilde{\nu }$/cm^−1^: 3060, 3032 (Ar C‒H), 2931, 2925 (C‒H), 1663 (C=O), 1590 (C–N amide), 1341 (C=C), 1232 (C–O), 1072 (C–O–C); ^1^H NMR (CDCl_3_, 400 MHz) *δ*/ppm: 8.59 (dd, 2H, *J* = 7.2 Hz, *J* = 0.9 Hz), 8.19 (dd, 2H, *J* = 8.3 Hz, *J* = 1.0 Hz), 7.82–7.66 (m, 5H), 7.49–7.36 (m, 5H), 7.36–7.28 (m, 2H), 7.21 (s, 1H), 5.21 (s, 2H), 5.10–4.97 (m, 1H), 4.15 (t, 2H, *J* = 7.5 Hz), 2.55 (d, 1H, *J* = 5.9 Hz), 1.97–1.79 (m, 2H), 1.77–1.63 (m, 2H), 1.56–1.27 (m, 8H); ^13^C NMR (CDCl_3_, 100 MHz) *δ*/ppm: 164.1 (s, 2C), 154.4 (s), 136.5 (s), 134.3 (s), 133.8 (d, 2C), 133.5 (s), 131.5 (s), 131.1 (d, 2C), 128.8 (s), 128.7 (d, 2C), 128.16 (s), 128.13 (d), 127.7 (d), 127.4 (d, 2C), 126.9 (d, 2C), 126.3 (d), 126.1 (d), 126.0 (d), 123.9 (d), 122.7 (s, 2C), 106.6 (d), 71.2 (d), 70.1 (t), 40.4 (t), 37.4 (t), 29.4 (t), 29.2 (t), 28.0 (t), 27.0 (t), 26.0 (t); UPLC-MS/UV: method ➁, *t*_R_ = 1.59 min, *m*/*z* = 540.25 [M − OH]^+^, found 540.17; HRMS (MALDI TOF/TOF): calculated for C_37_H_35_NO_4_ [M + Na]^+^ 580.2464; found 580.2460.

- **Removal of the benzyl group—general procedure**

A round-bottom flask, equipped with a septum, was charged with benzyl ether **7** (1 mmol), THF (5 mL/mmol **7**), methanol (5 mL/mmol **7**), Pd/C (10%, 0.1 mmol) and ammonium formate (5.0 mmol). The reaction mixture was heated at 65 °C for 2–6 h and the progress of the reaction was monitored by means of UPLC-MS/UV (method ➁, see the ESI). After all the benzyl ether was consumed, the reaction mixture was cooled down to rt, filtered through a celite plug, and the solvent was removed on a rotary evaporator. The residue was purified by means of preparative HPLC-MS/UV or on a column of silica gel using EtOAc (0 to 30%)/cyclohexane as eluent to afford the pure product.

*N*-{4-[3-hydroxynaphthalen-2-yl]-4-hydroxybut-1-yl}-1,8-naphthalimide (**1a**) was prepared according to the general procedure from ether **7a** (350 mg, 0.70 mmol), Pd/C (10%, 75 mg, 70 μmol) and ammonium formate (220 mg, 3.49 mmol). After chromatography, the reaction furnished 113 mg (39%) of the pure product in the form of a pale yellow solid.

mp 205–207 °C; IR (ATR) $\tilde{\nu }$/cm^−1^: 3425 (O–H alcohol), 3207 (Ar O–H), 3045 (Ar C‒H), 2953, 2916 (C‒H), 1652 (C=O), 1585 (C–N amide), 1347 (C=C), 1239 (C–O); ^1^H NMR (DMSO-*d*_6_, 400 MHz) *δ*/ppm: 9.78 (br. s, 1H), 8.46 (dd, 2H, *J* = 7.2 Hz, *J* = 1.1 Hz), 8.44 (dd, 2H, *J* = 8.4 Hz, *J* = 1.0 Hz), 7.90–7.78 (m, 3H), 7.72 (dd, 1H, *J* = 7.9 Hz, *J* = 0.5 Hz), 7.62 (dd, 1H, *J* = 8.4 Hz, *J* = 0.5 Hz), 7.36–7.28 (m, 1H), 7.26–7.18 (m, 1H), 7.08 (s, 1H), 5.21 (br. s, 1H), 5.04–4.91 (m, 1H), 4.16–3.99 (m, 2H), 1.91–1.56 (m, 4H); ^13^C NMR (DMSO-*d*_6_, 100 MHz) *δ*/ppm: 163.3 (s, 2C), 152.6 (s), 135.3 (s), 134.2 (d, 2C), 133.2 (s), 131.2 (s), 130.7 (d, 2C), 127.6 (s), 127.4 (d), 127.3 (s), 127.2 (d, 2C), 125.4 (d), 125.3 (d), 125.0 (d), 122.6 (d), 122.0 (s, 2C), 108.3 (d), 67.0 (d), 35.2 (t), 24.2 (t), one CH_2_ signal is covered by the DMSO-signal; UPLC-MS/UV: method ➂, *t*_R_ = 1.34 min, *m*/*z* = 410.14 [M − H]^−^, found 410.48; *m*/*z* = 394.14 [M − OH]^+^, found 394.66; HRMS (MALDI TOF/TOF): calculated for C_26_H_21_NO_4_ [M + H]^+^ 412.1549; found 412.1542.

*N*-{4-[3-hydroxynaphthalen-2-yl]-4-hydroxypent-1-yl}-1,8-naphthalimide (**1b**) was prepared according to the general procedure from ether **7b** (330 mg, 0.64 mmol), Pd/C (10%, 68 mg, 64 μmol) and ammonium formate (214 mg, 3.39 mmol). After chromatography, the reaction furnished 121 mg (45%) of the pure product in the form of a pale yellow solid.

mp 167–168 °C; IR (ATR) $\tilde{\nu }$/cm^−1^: 3423 (O–H alcohol), 3231 (Ar O–H), 3042 (Ar C‒H), 2948, 2866 (C‒H), 1663 (C=O), 1591 (C–N amide), 1342 (C=C), 1242 (C–O); ^1^H NMR (DMSO-*d*_6_, 600 MHz) *δ*/ppm: 9.76 (s, 1H), 8.45 (dd, 2H, *J* = 7.2 Hz, *J* = 1.0 Hz), 8.42 (dd, 2H, *J* = 8.3 Hz, *J* = 1.0 Hz), 7.86–7.79 (m, 3H), 7.72 (d, 1H, *J* = 8.1 Hz), 7.61 (d, 1H, *J* = 7.9 Hz), 7.33–7.29 (m, 1H), 7.23–7.19 (m, 1H), 7.08 (s, 1H), 5.16 (d, 1H, *J* = 4.6 Hz), 4.95 (quin, 1H, *J* = 3.9 Hz), 4.02 (t, 2H, *J* = 7.4 Hz), 1.83–1.76 (m, 1H), 1.72–1.54 (m, 3H), 1.53–1.41 (m, 2H); ^13^C NMR (DMSO-*d*_6_, 150 MHz) *δ*/ppm: 163.3 (s, 2C), 152.8 (s), 135.6 (s), 134.2 (d, 2C), 133.2 (s), 131.2 (s), 130.6 (d, 2C), 127.6 (s), 127.4 (d), 127.3 (s), 127.2 (d, 2C), 125.4 (d), 125.3 (d), 124.9 (d), 122.5 (d), 122.0 (s, 2C), 108.3 (d), 67.1 (d), 37.5 (t), 27.6 (t), 23.1 (t), one CH_2_ signal is covered by the DMSO-signal; UPLC-MS/UV: method ➂, *t*_R_ = 1.41 min, *m*/*z* = 424.16 [M − H]^−^, found 424.09; *m*/*z* = 408.16 [M − OH]^+^, found 408.17; HRMS (MALDI TOF/TOF): calculated for C_27_H_23_NO_4_ [M + H]^+^ 426.1705; found 426.1709.

*N*-{4-[3-hydroxynaphthalen-2-yl]-4-hydroxyhex-1-yl}-1,8-naphthalimide (**1c**) was prepared according to the general procedure from ether **7c** (350 mg, 0.66 mmol), Pd/C (10%, 70 mg, 66 μmol) and ammonium formate (208 mg, 3.30 mmol). After chromatography the reaction furnished 169 mg (58%) of the pure product in the form of a pale yellow solid.

mp 145–146 °C; IR (ATR) $\tilde{\nu }$/cm^−1^: 3521 (O–H alcohol), 3264 (Ar O–H), 3052 (Ar C‒H), 2939, 2928 (C‒H), 1645 (C=O), 1589 (C–N amide), 1342 (C=C), 1238 (C–O); ^1^H NMR (DMSO-*d*_6_, 400 MHz) *δ*/ppm: 9.75 (br. s, 1H), 8.54–8.40 (m, 4H), 7.86 (dd (t), 2H, *J* = 7.8 Hz), 7.84 (s, 1H), 7.73 (d, 1H, *J* = 7.9 Hz), 7.62 (d, 1H, *J* = 8.1 Hz), 7.36–7.28 (m, 1H), 7.26–7.18 (m, 1H), 7.08 (s, 1H), 5.15 (br. s, 1H), 4.99–4.90 (m, 1H), 4.03 (t, 2H, *J* = 7.4 Hz), 1.82–1.27 (m, 8H); ^13^C NMR (DMSO-*d*_6_, 100 MHz) *δ*/ppm: 163.3 (s, 2C), 152.8 (s), 135.7 (s), 134.2 (d, 2C), 133.2 (s), 131.2 (s), 130.7 (d, 2C), 127.7 (s), 127.4 (d), 127.3 (s), 127.1 (d, 2C), 125.4 (d), 125.3 (d), 124.9 (d), 122.5 (d), 122.0 (s, 2C), 108.2 (d), 67.0 (d), 37.6 (t), 27.6 (t), 26.6 (t), 25.2 (t), one CH_2_ signal is covered by the DMSO-signal; UPLC-MS/UV: method ➂, *t*_R_ = 1.49 min, *m*/*z* = 438.17 [M − H]^−^, found 438.76; *m*/*z* = 422.18 [M − OH]^+^, found 422.71; HRMS (MALDI TOF/TOF): calculated for C_28_H_25_NO_4_ [M + Na]^+^ 462.1681; found 462.1678.

*N*-{4-[3-hydroxynaphthalen-2-yl]-4-hydroxyhept-1-yl}-1,8-naphthalimide (**1d**) was prepared according to the general procedure from ether **7d** (350 mg, 0.64 mmol), Pd/C (10%, 69 mg, 65 μmol) and ammonium formate (203 mg, 3.22 mmol). After chromatrography the reaction furnished 95 mg (33%) of the pure product in the form of a pale yellow solid.

mp 75–76 °C; IR (ATR) $\tilde{\nu }$/cm^−1^: 3314 (O–H), 3056 (Ar C‒H), 2929, 2855 (C‒H), 1648 (C=O), 1589 (C–N amide), 1343 (C=C), 1234 (C–O); ^1^H NMR (DMSO-*d*_6_, 600 MHz) *δ*/ppm: 9.74 (s, 1H), 8.46 (dd, 2H, *J* = 7.3 Hz, *J* = 1.1 Hz), 8.42 (dd, 2H, *J* = 8.3 Hz, *J* = 1.0 Hz), 7.84 (dd (t), 2H, *J* = 7.6 Hz), 7.80 (s, 1H), 7.73 (d, 1H, *J* = 7.9 Hz), 7.61 (d, 1H, *J* = 7.9 Hz), 7.33–7.28 (m, 1H), 7.23–7.18 (m, 1H), 7.07 (s, 1H), 5.12 (d, 1H, *J* = 4.6 Hz), 4.94 (quin, 1H, *J* = 3.9 Hz), 4.01 (t, 2H, *J* = 7.5 Hz), 1.74–1.68 (m, 1H), 1.64–1.57 (m, 2H), 1.56–1.49 (m, 1H), 1.45–1.32 (m, 6H); ^13^C NMR (DMSO-*d*_6_, 150 MHz) *δ*/ppm: 163.3 (s, 2C), 152.8 (s), 135.7 (s), 134.2 (d, 2C), 133.2 (s), 131.2 (s), 130.6 (d, 2C), 127.7 (s), 127.4 (d), 127.3 (s), 127.1 (d, 2C), 125.4 (d), 125.3 (d), 125.0 (d), 122.5 (d), 122.0 (s, 2C), 108.3 (d), 67.1 (d), 37.7 (t), 28.8 (t), 27.5 (t), 26.6 (t), 25.3 (t), one CH_2_ signal is covered by the DMSO-signal; UPLC-MS/UV: method ➂, *t*_R_ = 1.56 min, *m*/*z* = 452.19 [M − H]^−^, found 452.14; *m*/*z* = 436.19 [M − OH]^+^, found 436.22; HRMS (MALDI TOF/TOF): calculated for C_29_H_27_NO_4_ [M+K]^+^ 492.1577; found 492.1592.

*N*-{4-[3-hydroxynaphthalen-2-yl]-4-hydroxyoct-1-yl}-1,8-naphthalimide (**1e**) was prepared according to the general procedure from ether **7e** (350 mg, 0.63 mmol), Pd/C (10%, 67 mg, 63 μmol) and ammonium formate (198 mg, 3.14 mmol). After chromatography the reaction furnished 45 mg (15%) of the pure product in the form of a pale yellow solid.

mp 69–70 °C; IR (ATR) $\tilde{\nu }$/cm^−1^: 3423 (O–H alcohol), 3301 (Ar O–H), 3053 (Ar C‒H), 2930, 2855 (C‒H), 1657 (C=O), 1590 (C–N amide), 1346 (C=C), 1236 (C–O); ^1^H NMR (DMSO-*d*_6_, 600 MHz) *δ*/ppm: 9.74 (s, 1H), 8.46 (dd, 2H, *J* = 7.2 Hz, *J* = 1.0 Hz), 8.41 (dd, 2H, *J* = 8.2 Hz, *J* = 0.7 Hz), 7.84 (dd (t), 2H, *J* = 7.6 Hz), 7.80 (s, 1H), 7.73 (d, 1H, *J* = 8.0 Hz), 7.61 (d, 1H, *J* = 8.1 Hz), 7.32–7.28 (m, 1H), 7.23–7.18 (m, 1H), 7.07 (s, 1H), 5.11 (d, 1H, *J* = 4.6 Hz), 4.94 (quin, 1H, *J* = 3.9 Hz), 4.00 (t, 2H, *J* = 7.5 Hz), 1.76–1.67 (m, 1H), 1.64–1.48 (m, 3H), 1.45–1.16 (m, 8H); ^13^C NMR (CDCl_3_, 150 MHz) *δ*/ppm: 163.3 (s, 2C), 152.8 (s), 135.7 (s), 134.2 (d, 2C), 133.2 (s), 131.2 (s), 130.6 (d, 2C), 127.7 (s), 127.4 (d), 127.3 (s), 127.1 (d, 2C), 125.4 (d), 125.3 (d), 125.0 (d), 122.5 (d), 122.0 (s, 2C), 108.3 (d), 67.2 (d), 37.7 (t), 28.9 (t), 28.8 (t), 27.4 (t), 26.8 (t), 25.3 (t), one CH_2_ signal is covered by the DMSO-signal; UPLC-MS/UV: method ➂, *t*_R_ = 1.64 min, *m*/*z* = 466.20 [M − H]^−^, found 466.09; *m*/*z* = 450.21 [M − OH]^+^, found 450.22; HRMS (MALDI TOF/TOF): calculated for C_30_H_29_NO_4_ [M+K]^+^ 506.1734; found 506.1751.

- **General procedure for the preparation of 1-OMe ethers using the acid-catalyzed thermal method**

A round-bottom flask (10 mL) was charged with a derivative **1** (≈10 mg), methanol (3.0 mL) and concentrated sulfuric acid (50 μL). The reaction mixture was stirred at rt for 15 h and then diluted with H_2_O (10 mL). The reaction mixture was neutralized with a saturated aqueous solution of NaHCO_3_ (≈2 mL), transferred to a separation funnel and extracted with diethyl ether (3 × 5 mL). The combined organic layers were dried over anhydrous MgSO_4_ and filtered and the solvent was removed on a rotary evaporator. The residue was chromatographed on a TLC using cyclohexane-EtOAc (7:3 *v/v*) as eluent to afford the pure product.

*N*-{4-[3-hydroxynaphthalen-2-yl]-4-methoxybut-1-yl}-1,8-naphthalimide (**1a-OMe**) was prepared according to the general procedure from alcohol **1a** (10 mg, 24 μmol). After chromatrographic separation the reaction afforded 5 mg (44%) of the pure product in the form of a pale yellow solid.

mp 64–65 °C; IR (ATR) $\tilde{\nu }$/cm^−1^: 3229 (Ar O–H), 3054 (Ar C‒H), 2948, 2918 (C‒H), 1646 (C=O), 1588 (C–N amide), 1344 (C=C), 1233 (C–O), 1105 (C–O–C); ^1^H NMR (CDCl_3_, 600 MHz) *δ*/ppm: 8.58 (dd, 2H, *J* = 7.2 Hz, *J* = 1.0 Hz), 8.19 (dd, 2H, *J* = 8.2 Hz, *J* = 1.0 Hz), 8.02 (s, 1H), 7.74 (dd (t), 2H, *J* = 7.7 Hz), 7.69 (dd, 1H, *J* = 8.1 Hz, *J* = 0.6 Hz), 7.66 (dd, 1H, *J* = 8.2 Hz, *J* = 0.4 Hz), 7.52 (s, 1H), 7.39–7.35 (m, 1H), 7.29–7.26 (m, 1H), 7.20 (s, 1H), 4.58–4.53 (m, 1H), 4.31–4.19 (m, 2H), 3.42 (s, 3H), 2.17–2.10 (m, 1H), 2.00–1.86 (m, 2H), 1.83–1.75 (m, 1H); ^13^C NMR (CDCl_3_, 150 MHz) *δ*/ppm: 164.2 (s, 2C), 153.4 (s), 134.4 (s), 133.9 (d, 2C), 131.5 (s), 131.2 (d, 2C), 128.15 (s), 128.13 (s), 127.9 (d), 127.4 (d), 127.2 (s), 126.9 (d, 2C), 126.2 (d, 2C), 123.4 (d), 122.6 (s, 2C), 111.4 (d), 85.5 (d), 57.4 (q), 39.7 (t), 33.3 (t), 24.6 (t); UPLC-MS/UV: method ➁, *t*_R_ = 1.30 min, *m*/*z* = 424.16 [M − H]^−^, found 424.08; *m*/*z* = 394.14 [M − OCH_3_]^+^, found 394.16; HRMS (MALDI TOF/TOF): calculated for C_27_H_23_NO_4_ [M + Na]^+^ 448.1525; found 448.1520.

*N*-{4-[3-hydroxynaphthalen-2-yl]-4-methoxypent-1-yl}-1,8-naphthalimide (**1b-OMe**) was prepared according to the general procedure from alcohol **1b** (10 mg, 24 μmol). After chromatographic separation the reaction afforded 6 mg (60%) of the pure product in the form of a pale yellow solid.

mp 58–60 °C; IR (ATR) $\tilde{\nu }$/cm^−1^: 3320 (Ar O–H), 3056 (Ar C‒H), 2931, 2863 (C‒H), 1657 (C=O), 1589 (C–N amide), 1342 (C=C), 1234 (C–O), 1169 (C–O–C); ^1^H NMR (CDCl_3_, 600 MHz) *δ*/ppm: 8.58 (dd, 2H, *J* = 7.3 Hz, *J* = 1.0 Hz), 8.20 (dd, 2H, *J* = 8.3 Hz, *J* = 0.9 Hz), 8.03 (s, 1H), 7.74 (dd (t), 2H, *J* = 7.7 Hz), 7.69 (t, 2H, *J* = 7.6 Hz), 7.47 (s, 1H), 7.40–7.36 (m, 1H), 7.29–7.26 (m, 1H), 7.21 (s, 1H), 4.49–4.45 (m, 1H), 4.17 (t, 2H, *J* = 7.6 Hz), 3.40 (s, 3H), 2.15–2.06 (m, 1H), 1.90–1.82 (m, 1H), 1.82–1.73 (m, 2H), 1.65–1.57 (m, 1H), 1.50–1.42 (m, 1H); ^13^C NMR (CDCl_3_, 150 MHz) *δ*/ppm: 164.1 (s, 2C), 153.5 (s), 134.4 (s), 133.8 (d, 2C), 131.5 (s), 131.2 (d, 2C), 128.15 (s), 128.11 (s), 127.7 (d), 127.4 (s), 127.3 (d), 126.9 (d, 2C), 126.2 (d, 2C), 123.4 (d), 122.6 (s, 2C), 111.3 (d), 85.8 (d), 57.3 (q), 40.1 (t), 35.5 (t), 27.6 (t), 23.4 (t); UPLC-MS/UV: method ➁, *t*_R_ = 1.35 min, *m*/*z* = 438.17 [M − H]^−^, found 438.61; *m*/*z* = 408.16 [M − OCH_3_]^+^, found 408.64; HRMS (MALDI TOF/TOF): calculated for C_28_H_25_NO_4_ [M + Na]^+^ 462.1681; found 462.1671.

*N*-{4-[3-hydroxynaphthalen-2-yl]-4-methoxyhex-1-yl}-1,8-naphthalimide (**1c-OMe**) was prepared according to the general procedure from alcohol **1c** (10 mg, 23 μmol). After chromatographic separation the reaction afforded 6 mg (53%) of the pure product in the form of pale yellow solid.

mp 55–56 °C; IR (ATR) $\tilde{\nu }$/cm^−1^: 3319 (Ar O–H), 3056 (Ar C‒H), 2933, 2858 (C‒H), 1658 (C=O), 1589 (C–N amide), 1341 (C=C), 1236 (C–O), 1106 (C–O–C); ^1^H NMR (CDCl_3_, 600 MHz) *δ*/ppm: 8.58 (dd, 2H, *J* = 7.2 Hz, *J* = 1.0 Hz), 8.19 (dd, 2H, *J* = 8.3 Hz, *J* = 1.0 Hz), 8.03 (s, 1H), 7.74 (dd (t), 2H, *J* = 7.7 Hz), 7.69 (d, 1H, *J* = 8.1 Hz), 7.67 (d, 1H, *J* = 8.2 Hz), 7.45 (s, 1H), 7.40–7.36 (m, 1H), 7.30–7.26 (m, 1H), 7.21 (s, 1H), 4.43 (t, 1H, *J* = 7.0 Hz), 4.19–4.13 (m, 2H), 3.39 (s, 3H), 2.08–1.98 (m, 1H), 1.83–1.67 (m, 3H), 1.59–1.51 (m, 1H), 1.49–1.41 (m, 2H), 1.41–1.33 (m, 1H); ^13^C NMR (CDCl_3_, 150 MHz) *δ*/ppm:164.1 (s, 2C), 153.5 (s), 134.4 (s), 133.8 (d, 2C), 131.5 (s), 131.1 (d, 2C), 128.1 (s), 128.0 (s), 127.7 (d), 127.5 (s), 127.3 (d), 126.9 (d, 2C), 126.2 (d, 2C), 123.4 (d), 122.7 (s, 2C), 111.3 (d), 86.0 (d), 57.2 (q), 40.2 (t), 35.8 (t), 27.9 (t), 26.8 (t), 25.6 (t); UPLC-MS/UV: method ➁, *t*_R_ = 1.41 min, *m*/*z* = 452.19 [M − H]^−^, found 452.23; *m*/*z* = 422.18 [M − OCH_3_]^+^, found 422.26; HRMS (MALDI TOF/TOF): calculated for C_29_H_27_NO_4_ [M + Na]^+^ 476.1838; found 476.1815.

*N*-{4-[3-hydroxynaphthalen-2-yl]-4-methoxyhept-1-yl}-1,8-naphthalimide (**1d-OMe**) was prepared according to the general procedure from alcohol **1d** (10 mg, 22 μmol). After chromatographic separation the reaction afforded 8 mg (80%) of the pure product in the form of a pale yellow solid.

mp 38–40 °C; IR (ATR) $\tilde{\nu }$/cm^−1^: 3343 (Ar O–H), 3055 (Ar C‒H), 2930, 2856 (C‒H), 1657 (C=O), 1589 (C–N amide), 1342 (C=C), 1233 (C–O), 1107 (C–O–C); ^1^H NMR (CDCl_3_, 600 MHz) *δ*/ppm: 8.59 (dd, 2H, *J* = 7.2 Hz, *J* = 1.0 Hz), 8.19 (dd, 2H, *J* = 8.2 Hz, *J* = 0.9 Hz), 8.05 (s, 1H), 7.74 (dd (t), 2H, *J* = 7.7 Hz), 7.71 (d, 1H, *J* = 8.1 Hz), 7.67 (d, 1H, *J* = 8.1 Hz), 7.46 (s, 1H), 7.40–7.36 (m, 1H), 7.30–7.23 (m, 1H), 7.22 (s, 1H), 4.42 (t, 1H, *J* = 7.0 Hz), 4.16 (t, 2H, *J* = 7.6 Hz), 3.40 (s, 3H), 2.05–1.97 (m, 1H), 1.81–1.68 (m, 3H), 1.51–1.26 (m, 6H); ^13^C NMR (CDCl_3_, 150 MHz) *δ*/ppm:164.1 (s, 2C), 153.5 (s), 134.3 (s), 133.8 (d, 2C), 131.5 (s), 131.1 (d, 2C), 128.1 (s), 128.0 (s), 127.7 (d), 127.5 (s), 127.3 (d), 126.9 (d, 2C), 126.2 (d, 2C), 123.4 (d), 122.7 (s, 2C), 111.3 (d), 86.0 (d), 57.2 (q), 40.3 (t), 35.8 (t), 29.0 (t), 28.0 (t), 26.9 (t), 25.8 (t); UPLC-MS/UV: method ➁, *t*_R_ = 1.47 min, *m*/*z* = 466.20 [M − H]^−^, found 466.19; *m*/*z* = 436.19 [M − OCH_3_]^+^, found 436.27; HRMS (MALDI TOF/TOF): calculated for C_30_H_29_NO_4_ [M + Na]^+^ 490.1994; found 490.1983.

*N*-{4-[3-hydroxynaphthalen-2-yl]-4-methoxyoct-1-yl}-1,8-naphthalimide (**1e-OMe**) was prepared according to the general procedure from alcohol **1e** (10 mg, 21 μmol). After chromatographic separation the reaction afforded 6 mg (53%) of the pure product in the form of a pale yellow solid.

mp 38–40 °C; IR (ATR) $\tilde{\nu }$/cm^−1^: 3343 (Ar O–H), 3055 (Ar C‒H), 2928, 2855 (C‒H), 1658 (C=O), 1589 (C–N amide), 1342 (C=C), 1233 (C–O), 1167 (C–O–C); ^1^H NMR (CDCl_3_, 600 MHz) *δ*/ppm: 8.59 (dd, 2H, *J* = 7.2 Hz, *J* = 1.0 Hz), 8.20 (dd, 2H, *J* = 8.2 Hz, *J* = 0.9 Hz), 8.06 (s, 1H), 7.74 (dd (t), 2H, *J* = 7.7 Hz), 7.71 (dd, 1H, *J* = 8.3 Hz, *J* = 0.6 Hz), 7.68 (dd, 1H, *J* = 8.2 Hz, *J* = 0.6 Hz), 7.46 (s, 1H), 7.40–7.36 (m, 1H), 7.30–7.27 (m, 1H), 7.22 (s, 1H), 4.26 (t, 1H, *J* = 7.0 Hz), 4.16 (t, 2H, *J* = 7.6 Hz), 3.40 (s, 3H), 2.04–1.95 (m, 1H), 1.80–1.67 (m, 3H), 1.49–1.35 (m, 4H), 1.33–1.23 (m, 4H); ^13^C NMR (CDCl_3_, 150 MHz) *δ*/ppm: 164.2 (s, 2C), 153.5 (s), 134.3 (s), 133.8 (d, 2C), 131.5 (s), 131.1 (d, 2C), 128.16 (s), 128.10 (s), 127.7 (d), 127.6 (s), 127.3 (d), 126.9 (d, 2C), 126.2 (d, 2C), 123.4 (d), 122.7 (s, 2C), 111.2 (d), 86.1 (d), 57.3 (q), 40.4 (t), 35.8 (t), 29.1 (t, 2C), 28.0 (t), 26.9 (t), 25.8 (t); UPLC-MS/UV: method ➁, *t*_R_ = 1.52 min, *m*/*z* = 480.22 [M − H]^−^, found 480.26; *m*/*z* = 450.21 [M − OCH_3_]^+^, found 450.23; HRMS (MALDI TOF/TOF): calculated for C_31_H_31_NO_4_ [M + Na]^+^ 504.2151; found 504.2133.

- **Irradiation experiments—general**

A quartz test tube (20 mL) was filled with a solution of **1** in CH_3_OH (7 mL/1 mg of **1**), and the solution was diluted with H_2_O. The final CH_3_OH-H_2_O ratio was 4:1 (*v/v*). The solution was sealed with a septum and purged with Ar for 15 min and irradiated in a reactor equipped with 8 or 14 lamps (1 lamp = 8 W) with the output at 254 nm, or ≈350 nm. CH_3_OH for the irradiations was of HPLC purity, and Milli-Q H_2_O was used. The solutions were irradiated from 60 to 180 min, and the composition of the irradiated solutions was analyzed using UPLC-MS/UV.

- **Irradiation of 1a**

According to the general procedure, **1a** (9 mg, 22 μmol) was dissolved in CH_3_OH-H_2_O (4:1 *v/v*) (20 mL) and irradiated at 350 nm (14 lamps) over 180 min. After the irradiation, **1a-OMe** and 1,8-naphthalimide were detected in a complex mixture of photoproducts determined, by analysis of the solution using UPLC/MS-UV.

UPLC-MS/UV: method ➁, *t*_R_ (**1a**) = 1.16 min, *m*/*z* = 394.14 [M − OH]^+^, found 394.26; *t*_R_ (**1a-OMe**) = 1.32 min, *m*/*z* = 394.14 [M − OCH_3_]^+^, found 394.31; *t*_R_ (1,8-naphthalimide) = 0.74 min, *m*/*z* = 198.05 [M + H]^+^, found 198.21.

- **Irradiation of 1c**

According to the general procedure, **1c** (7 mg, 13 μmol) was dissolved in CH_3_OH-H_2_O (4:1 *v/v*) (20 mL) and irradiated at 254 nm or 350 nm (8 lamps) over 60 min or at 350 nm (14 lamps) over 180 min. After the irradiation, **1c-OMe** was detected in a complex mixture of photoproducts, determined by analysis of the solution using UPLC/MS-UV.

UPLC-MS/UV: method ➁ (at 254 nm, 60 min) *t*_R_ (**1c**) = 1.27 min, *m*/*z* = 438.17 [M − H]^−^, found 438.55; *t*_R_ (**1c-OMe**) = 1.42 min, *m*/*z* = 422.18 [M − OCH_3_]^+^, found 422.66; [at 350 nm, 60 min] *t*_R_ (**1c**) = 1.25 min, *m*/*z* = 438.17 [M − H]^−^, found 438.55; *t*_R_ (**1c-OMe**) = 1.42 min, *m*/*z* = 422.18 [M − OCH_3_]^+^, found 422.60.

- **Irradiation of 1e**

According to the general procedure, **1e** (8 mg, 22 μmol) was dissolved in CH_3_OH-H_2_O (4:1 *v/v*) (20 mL) and irradiated at 350 nm (14 lamps) over 180 min. After the irradiation, **1e-OMe** and 1,8-naphthalimide were detected in a complex mixture of photoproducts, determined by analysis of the solution using UPLC/MS-UV.

UPLC-MS/UV: method ➁, *t*_R_ (**1e**) = 1.39 min, *m*/*z* = 450.21 [M − OH]^+^, found 450.36; *t*_R_ (**1e-OMe**) = 1.55 min, *m*/*z* = 450.36 [M − OCH_3_]^+^, found 450.41; *t*_R_ (1,8-naphthtalimide) = 0.74 min, *m*/*z* = 198.05 [M + H]^+^, found 198.16.

- **Quantum yield of methanolysis**

Quantum yields for the photomethanolysis reactions for **1** were determined using a KI/KIO_3_ (*Φ*_254_ = 0.74) actinometer [81], as recently described by us [97]. Solutions of **1** in CH_3_OH-H_2_O (4:1 *v/v*) and the actinometer were freshly prepared and their concentrations were adjusted to have absorbances of 0.4–0.8 at 254 nm. After the adjustment of the concentrations and measurement of the corresponding UV-vis spectra, the solutions were purged with a stream of Ar (20 min), and then, sealed with a cap. The cells were placed in a holder, which ensured an equal distance of all samples from the lamp, and were irradiated at the same time in the reactor with 1 lamp at 254 nm for 6 h (1 min for the actinometer). Before and after the irradiation, the samples were taken from the cells with the use of a syringe and were analyzed using UPLC-MS/UV to determine photochemical conversions. The conversion did not exceed 30% to avoid a change in the absorbance or filtering of the light by the product. Based on the actinometer conversion, irradiance was calculated according to Equations (S1)–(S5), reported in the ESI. The average value of three measurements was reported.

- **Absorption and fluorescence measurements**

Absorption spectra were recorded on a PG T80/T80+ or a Varian Cary 100 Bio spectrophotometer at rt. Fluorescence measurements were performed on an Agilent Cary Eclipse fluorimeter with slits corresponding to the bandpass of 10 nm for the excitation and 20 nm for the emission. The samples were dissolved in CH_3_CN or CH_3_CN-H_2_O (4:1 *v/v*) and the concentrations were adjusted to absorbances of less than 0.1 at the excitation wavelengths of 300, 310 and 320 nm. Fluorescence quantum yields were determined through the comparison of the integral of the emission bands with that of quinine sulfate in 0.05 M H_2_SO_4_ (Φ_f_ = 0.546) [76]. One fluorescence measurement was performed by exciting sample at three different wavelengths and the average value was calculated (Equation (S6) in the ESI). Prior to the measurements, the solutions were purged with Ar for 15 min. The measurement was performed at rt (25 °C). Fluorescence decays were measured using the TC-SPC method on an Edinburgh FS5 spectrometer equipped with pulsed LEDs at 280 or 340 nm. The duration of the pulse was ≈800 ps. Fluorescence signals were monitored over 1023 channels with the time increment of ≈20 ps/channel. The decays were collected until they reached 1000 counts in the peak channel. The histograms were analyzed by means of a nonlinear least-squares deconvolution method using Equation (S7) in the ESI.

- **Thermal denaturation experiments with ct-DNA** [86]

A stock solution of **1a** or **1e** was prepared in DMSO (*c* = 2.0 × 10^−2^ M), whereas the stock solution of ct-DNA was prepared in aqueous cacodylate buffer (pH = 7.0, 50 mM) *c*(ct-DNA) = 1.1 × 10^−3^ M. In the denaturation experiments, the ct-DNA solution was diluted in a quartz UV-vis cell (with the optical path of 1.0 cm) with cacodylate buffer to the concentration of *c* = 3.0 × 10^−5^ M, and the appropriate amount of the solution of **1a** or **1e** was added to reach the desired ratio *r* ((**1**)/(ct-DNA)) = 0.3 (containing <1% DMSO). The dependence of the absorbance at 260 nm as a function of temperature was measured on a Cary 100 Bio (Agilent Varian) UV-vis spectrometer. The temperature was varied from 25 °C to 98 °C in intervals of 0.5 °C. The denaturation temperature *T*_m_ values are the midpoints of the transition curves, determined from the maximum of the first derivative. ∆*T*_m_ values were calculated by subtracting the *T*_m_ of the free nucleic acid from that of the respective complex, with ∆*T*_m_ values being the average of at least two independent measurements and the error in ∆*T*_m_ being ca. ±0.5 °C.

- **Fluorescence titrations with ct-DNA**

Stock solutions of solution **1a** or **1e** were prepared in DMSO (*c* = 1.0 × 10^−3^ M). For the titrations, the stock solutions were diluted in a fluorescence cell (3 mL) with cacodylate buffer (pH 7.0, 50 mM) to reach the concentration *c* = 2.0 × 10^−6^ M (containing <1% DMSO). The fluorescence spectra were measured on a Cary Eclipse (Agilent Technologies, Santa Clara, CA, USA) at 25 °C. The samples were excited at 320 nm, and the emission was recorded in the range of 340–650 nm. The excitation slit was set to the bandpass of 10 nm, and the emission slit to 20 nm. Small aliquots of the solutions of the ct-DNA (*c* = 5.0 × 10^−3^ M) were added to the solution and after an incubation time of 2 min, fluorescence spectra were measured. Data obtained by means of fluorescence titrations were processed using nonlinear regression analysis according to the Scatchard model (McGhee, von Hippel formalism) [88].

- **Circular dichroism spectroscopy with ct-DNA**

Circular dichroism spectra were measured on a Jasco J-815 spectrometer in quartz cells with an optical path of 1 cm at 25 °C. To the polynucleotide solutions in cells (*c* = 3.0 × 10^−5^ M) in cacodylate buffer (pH = 7.0, 50 mM), aliquots of the solution of **1a** or **1e** in buffer (*c* = 1.0 × 10^−3^ M) were added to reach the concentration ratio *r*(**1**)/(polynucleotide) = 0.1–0.7 (containing <1% DMSO). The CD spectra were recorded in the wavelength range 220–400 nm with a scanning rate of 200 nm/min and with 2 accumulations.

- **Fluorescence titrations with BSA**

Stock solutions of solution **1a** or **1e** were prepared in DMSO (*c* = 1.0 × 10^−3^ M). For the titrations, the stock solutions were diluted in a fluorescence cell (3 mL) with cacodylate buffer (pH 7.0, 50 mM) to reach the concentration *c* = 1.0 × 10^−6^ M (containing <1% DMSO). A stock solution of BSA in cacodylate buffer was prepared *c* = 1.0 × 10^−3^ M. The fluorescence spectra were measured on a Cary Eclipse (Agilent Technologies) at 25 °C. The samples were excited at 320 nm, and the emission was recorded in the range 340–700 nm. The excitation slit was set to the bandpass of 10 nm, and the emission slit to 20 nm. Small aliquots of the solutions of BSA were added to the solution and, after an incubation time of 2 min, fluorescence spectra were measured. Data obtained via fluorescence titrations were processed by means of multicomponent nonlinear regression analysis in HypSpec2014 software [93].

- **Photochemical alkylation of BSA**

A fluorescence cell containing an aqueous solution of BSA (*c* = 1.4 × 10^−5^ M) in cacodylate buffer (2 mL, 50 mM, pH 7.0) and **1a** or **1e** (*c* = 1.0 × 10^−6^ M) in DMSO (total amount < 1%) was irradiated in a Luzchem photoreactor with 8 lamps at 350 nm over 5 min. Prior to the MS analysis, 100 μL of the irradiated solution was purified using Aspire RP30 Desalting Tips (ThermoFisher Scientific, MA, USA). After the purification, the solvent was evaporated in Speed-Vac (Eppendorf, Switzerland) and re-constituted in 10 μL of the sinapinic acid matrix (5 mg/mL of sinapinic acid in 1:1, CH_3_CN:0.1% TFA, *v/v*). The mixture of the analyte and the matrix was deposited onto the MALDI plate (1 μL) and dried slowly in a stream of cold air. MALDI mass spectra were recorded on an AB SCIEX MALDI-TOF/TOF 4800+ (MA, USA).

- **Laser flash photolysis (LFP)**

The measurements were performed on a LP980 Edinburgh Instruments spectrometer. For the excitation, a Qsmart Q450 Quantel YAG laser was used, the fourth at 266 nm or the third harmonic at 355 nm. The energy of the laser pulse was set to 20 mJ and the pulse duration was 7 ns. Absorbances at the excitation wavelength were set to 0.3–0.4. The static cells were used and they were frequently exchanged to assure no absorption of light by photoproducts. The solutions were purged for 15 min with Ar or O_2_ prior to the measurements, which were conducted at 25 °C.

## Figures and Tables

**Table 1:** Quantum yields of fluorescence Φ_f_^a^ and efficiencies of FRET (Φ_FRET_) ^b^.

Comp.	Φ_f_ (CH_3_CN) × 10^3^	Φ_FRET_ (CH_3_CN) ^b^	Φ_f_ (CH_3_CN-H_2_O) × 10^3 c^
**1a**	12 ± 1	0.70	14.3 ± 0.7
**1b**	15 ± 2	0.79	32.6 ± 0.5
**1c**	16 ± 2	0.82	37.4 ± 0.7
**1d**	17 ± 2	0.71	40 ± 1
**1e**	18 ± 2	0.69	48.2 ± 0.9

^a^ Quantum yield of fluorescence was measured using quinine sulfate in 0.5 M H_2_SO_4_ (Φ_f_ = 0.546) [76]. The fluorescence spectra were measured by exciting at 300, 310 and 320 nm, and the average values of the quantum yields (Equation (S1) in the ESI) were calculated. The measurement was performed three times and the average values are reported. The associated errors correspond to the maximum absolute deviation. ^b^ The efficiency of the Förster resonance energy transfer was determined by shortening of the average decay time of the donor (naphthol) detected at 340 nm, compared to the decay time of Naphth, according to Equations (S8) and (S9) in the ESI. ^c^ CH_3_CN-H_2_O (4:1, *v*/*v*).

**Table 2:** Quantum yields (Φ_R_) of the photomethanolyses of **1** in CH_3_OH-H_2_O (4:1 *v*/*v*) ^a^.

Comp.	Φ_R_ × 10^5^
**1a**	3.0 ± 0.3
**1b**	2.6 ± 0.8
**1c**	2.8 ± 0.5
**1d**	3.5 ± 0.9
**1e**	3.6 ± 0.8

^a^ KI/KIO_3_ (Φ_R_ = 0.74) [81] was used as an actinometer and the samples were excited at 254 nm and analyzed using UPLC-MS. The measurement was performed in triplicate and the average value is reported. The quoted error corresponds to the maximum standard deviation.

## Data Availability

Electronic supporting information (ESI) of this article contains synthetic procedures for the preparation of known compounds, UV-vis and fluorescence data, noncovalent binding to ct-DNA, noncovalent and covalent binding to BSA, data on antiproliferatve activity and copes of ^1^H and ^13^C NMR spectra of all new compounds.

## Supplementary Materials

The following are available online. Synthetic procedures for the preparation of known precursors, fluorescence spectra, LFP data and non-covalent binding to ct-DNA and BSA, as well as copies of ^1^H and ^13^C NMR spectra.
