# Supplementary material for: Photochemical Reactivity of Naphthol-Naphthalimide Conjugates and Their Biological Activity
Source: Molecules. 2021 Jun 2;26(11):3355. doi: 10.3390/molecules26113355 (PMC8199699; doi:10.3390/molecules26113355)
Supplement: Supplementary file 1 [file molecules-26-03355-s001.zip › molecules-1229835-supplementary.pdf]

**Electronic supporting information**

**for**

# Photochemical reactivity of naphthol-naphthalimide conjugates and their biological activity


Matija Sambol,[a, b] Patricia Benčić,[c, d] Antonija Erben,[a] Marija Matković,[a] Branka Mihaljević,[e] Ivo Piantanida,[a] Marijeta Kralj,[c] Nikola Basarić[a]*

[a] Department of Organic Chemistry and Biochemistry, Ruđer Bošković Institute, Bijenička cesta 54, 10 000 Zagreb, Croatia. Fax: + 385 1 4680 195; Tel: +385 1 4680 196

[b] Current address: Fidelta Ltd., Prilaz baruna Filipovića 29, 10000 Zagreb, Croatia

[c] Division of Material Chemistry, Ruđer Bošković Institute, Bijenička cesta 54, 10 000 Zagreb

[d] Current address: Albert-Ludwigs-Universität Freiburg, Institute of Pharmaceutical Sciences, Albertstr. 25, 79104 Freiburg, Germany

[e] Division of Material Chemistry, Ruđer Bošković Institute, Bijenička cesta 54, 10 000 Zagreb

Corresponding author's E-mail address: nbasaric@irb.hr


Content:


1. Synthetic procedures for the preparation of known compounds (Eqs S1-S5, Tables S1-S3) S2

2. UV-vis and fluorescence data (Figs S1-S10, Table S4 Eqs. S6-S9) S11

3. Laser flash photolysis (Figs S11-S16) S18

4. Noncovalent binding to ct-DNA (Figs S17-S22, Table S5-S7) S21

5. Noncovalent and covalent binding to BSA (Fig S23-S26 and Table S8) S26

6. Antiproliferative activity (Table S9) S29

7. NMR spectra S31

8. References S94

## 1. Synthetic procedure for the preparation of known compounds

**General procedure for the preparation of *N*-(ω-bromoalkyl)-1,8-naphthalimide (2)**[1]

A two neck round bottom flask, equipped with a condenser and a dropping funnel was charged with dibromoalkane (2.5 mmol), $K_2CO_3$ (4 mmol) and $CH_3CN$ (25 mL/1 mmol naphthalimide). The resulting suspension was heated to reflux temperature and stirred, while a suspension of 1,8-naphthalimide (1 mmol) in $CH_3CN$ (6 mL/mmol naphthalimide) was added dropwise. After complete addition, the reaction mixture was stirred at reflux temperature over additional 18 h, cooled to rt and filtered. The filtrate was concentrated on a rotary evaporator. Evaporation residue was dissolved in $CH_2Cl_2$ (15 mL) and silica gel was added (3×weight of the crude product). The solvent was removed on a rotary evaporator and the residue was transferred to a sinter funnel containing a silica gel plug (4 cm). The funnel content was flushed with cyclohexane (3×15 mL) and then with $CH_2Cl_2$ (3×20 mL). The $CH_2Cl_2$ solution was concentrated on rotary evaporator yielding product that was used without further purification in the next synthetic step.

***N*-(4-bromobut-1-yl)-1,8-naphthalimide (2a)**

Prepared according to the general procedure from 1,4-dibromobutane (6.54 mL, 54.77 mmol) and 1,8-naphthalimide (4.32 g, 21.9 mmol) in the presence of $K_2CO_3$ (12.11. g, 87.6 mmol). The reaction gave after the purification 3.90 g (54%) of the product in the form of colorless solid. NMR characterization is in accordance with the data from literature precedent.[1]

mp 105-106 °C (lit. 100-103 °C);[1] IR (ATR) $\tilde{\nu}$/cm$^{-1}$: 3078 (Ar C–H), 2968, 2870 (C–H), 1698 (Ar C=C), 1652 (C=O), 1592, 1586 (C–N amide), 775 (C–Br); $^1$H NMR ($CDCl_3$, 500 MHz) $\delta$/ppm: 8.59 (dd, 2H, $J$ = 7.3 Hz, $J$ = 0.7 Hz), 8.21 (dd, 2H, $J$ = 7.3 Hz, $J$ = 0.4 Hz), 7.75 (t, 2H, $J$ = 7.5 Hz), 4,23 (t, 2H, $J$ = 7.1 Hz), 3.48 (t, 2H, $J$ = 6,5 Hz), 2.03-1.95 (m, 2H), 1.95-1.87 (m, 2H); $^{13}$C NMR ($CDCl_3$, 125 MHz) $\delta$/ppm: 164.1 (s, 2C), 133.9 (d, 2C), 131.5 (s), 131.2 (d, 2C), 128.1 (s), 126.9 (d, 2C), 122.5 (s, 2C), 39.3 (t), 33.1 (t), 30.2 (t), 26.8 (t); UPLC-MS/UV: method ①, $t_R$ = 1.25 min, $m/z$ = 332.03 [M+H]$^+$, found 332.07, 334.06.

***N*-(5-bromopent-1-yl)-1,8-naphthalimide (2b)**

Prepared according to the general procedure from 1,5-dibromopentane (7.46 mL, 54.77 mmol) and 1,8-naphthalimide (4.32 g, 21.9 mmol) in the presence of $K_2CO_3$ (12.11. g, 87.6 mmol). The reaction gave after the purification 6.61 g (87%) of the product t in the form of colorless solid. NMR characterization is in accordance with the data from literature precedent.[1]

mp 110-112 °C (lit. 118-120 °C);[1] IR (ATR) $\tilde{\nu}$/cm$^{-1}$: 3061, 3018 (Ar C–H), 2954, 2969, 2860 (C–H), 1694 (Ar C=C), 1656 (C=O), 1586 (C–N amide), 775 (C–Br); $^1$H NMR ($CDCl_3$, 400

MHz) $\delta$/ppm: 8.59 (dd, 2H, $J$ = 7.3 Hz, $J$ = 1.1 Hz), 8.21 (dd, 2H, $J$ = 8.4 Hz, $J$ = 1.0 Hz), 7.78-7.72 (m, 2H), 4.20 (t, 2H, $J$ = 7.5 Hz), 3.43 (t, 2H, $J$ = 6.7 Hz), 1.96 (quin, 2H, $J$ = 7.0 Hz), 1.78 (quin, 2H, $J$ = 7.5 Hz), 1.61-1.53 (m, 2H); $^{13}$C NMR ($CDCl_3$, 100 MHz) $\delta$/ppm: 164.2 (s, 2C), 133.9 (d, 2C), 131.6 (s), 131.2 (d, 2C), 128.1 (s), 126.9 (d, 2C), 122.6 (s, 2C), 40.0 (t), 35.5 (t), 32.4 (t), 27.2 (t), 25.6 (t); UPLC-MS/UV: method ②, $t_R$ = 1.32 min, $m/z$ = 346.04 $[M+H]^+$, found 346.08, 348.09.

***N*-(6-bromohex-1-yl)-1,8-naphthalimide (2c)**

Prepared according to the general procedure from 1,6-dibromohexane (9.45 mL, 62.38 mmol) and 1,8-naphthalimide (4.92 g, 24.5 mmol) in the presence of $K_2CO_3$ (13.79 g, 99.8 mmol). The reaction gave after the purification 6.45 g (72%) of the product in the form of colorless solid. NMR characterization is in accordance with the data from literature precedent.[1]

mp 99-100 °C (lit. 90-94 °C);[1] IR (ATR) $\tilde{\nu}$/cm$^{-1}$: 3062(Ar C–H), 2933, 2856 (C–H), 1693(Ar C=C), 1660 (C=O), 1588 (C–N amide), 780 (C–Br); $^1$H NMR ($CDCl_3$, 600 MHz) $\delta$/ppm: 8.59 (d, 2H, $J$ = 7.2 Hz), 8.20 (d, 2H, $J$ = 8.2 Hz), 7.74 (t, 2H, $J$ = 7.5 Hz), 4.19 (t, 2H, $J$ = 7.5 Hz), 3.40 (t, 2H, $J$ = 6.9 Hz), 1.88 (quin, 2H, $J$ = 7.1 Hz), 1.76 (quin, 2H, $J$ = 7.6 Hz), 1.55-1.49 (m, 2H), 1.49-1.43 (m, 2H); $^{13}$C NMR ($CDCl_3$, 100 MHz) $\delta$/ppm: 164.2 (s, 2C), 133.8 (d, 2C), 131.5 (s), 131.2 (d, 2C), 128.1 (s), 126.9 (d, 2C), 122.7 (s, 2C), 40.2 (t), 33.8 (t), 32.6 (t), 27.8 (t, 2C), 26.2 (t); UPLC-MS/UV: method ②, $t_R$ = 1.40 min, $m/z$ = 360.06 $[M+H]^+$, found 360.12, 362.09.

***N*-(7-bromohept-1-yl)-1,8-naphthalimide (2d)**

Prepared according to the general procedure from 1,7-dibromoheptane (9.75 mL, 57.05 mmol) and 1,8-naphthalimide (4.50 g, 22.8 mmol) in the presence of $K_2CO_3$ (12.62 g, 91.3 mmol). The reaction gave after the purification 4.94 g (58%) of the product in the form of colorless solid.

mp 80-83 °C; IR (ATR) $\tilde{\nu}$/cm$^{-1}$: 3063 (Ar C–H), 2930, 2853 (C–H), 1692 (Ar C=C), 1657 (C=O), 1588 (C–N amide), 779 (C–Br); $^1$H NMR ($CDCl_3$, 400 MHz) $\delta$/ppm: 8.59 (dd, 2H, $J$ = 7.2 Hz, $J$ = 1.1 Hz), 8.20 (dd, 2H, $J$ = 8.3 Hz, $J$ = 1.0 Hz), 7.78-7.71 (m, 2H), 4.18 (t, 2H, $J$ = 7.6 Hz), 3.39 (t, 2H, $J$ = 6.8 Hz), 1.85 (quin, 2H, $J$ = 7.5 Hz), 1.74 (quin, 2H, $J$ = 7.5 Hz), 1.50-1.32 (m, 6H); $^{13}$C NMR ($CDCl_3$, 100 MHz) $\delta$/ppm: 164.1 (s, 2C), 133.8 (d, 2C), 131.5 (s), 131.1 (d, 2C), 128.1 (s), 126.9 (d, 2C), 122.7 (s, 2C), 40.3 (t), 33.9 (t), 32.7 (t), 28.4 (t), 28.0 (t), 27.9 (t), 26.9 (t); UPLC-MS/UV: method ②, $t_R$ = 1.47 min, $m/z$ = 374.08 $[M+H]^+$, found 374.17, 376.11.

***N*-(8-bromooct-1-yl)-1,8-naphthalimide (2e)**

Prepared according to the general procedure from 1,8-dibromooctane (11.49 mL, 62.38 mmol) and 1,8-naphthalimide (4.92 g, 24.9 mmol) in the presence of $K_2CO_3$ (13.79 g, 99.8 mmol). The

reaction gave after the purification 7.19 g (74%) of the product in the form of colorless solid. NMR characterization is in accordance with the data from literature precedent.[1]

mp 80-81 °C (lit. 70-75 °C);[1] IR (ATR) $\tilde{\nu}$/cm$^{-1}$: 3063 (Ar C–H), 2935, 2919, 2852 (C–H), 1695 (Ar C=C), 1658 (C=O), 1586 (C–N amide), 777 (C–Br); $^1$H NMR ($CDCl_3$, 400 MHz) $\delta$/ppm: 8.59 (dd, 2H, $J$ = 7.2 Hz, $J$ = 1.1 Hz), 8.20 (dd, 2H, $J$ = 8.3 Hz, $J$ = 1.0 Hz), 7.78-7.72 (m, 2H), 4.18 (t, 2H, $J$ = 7.6 Hz), 3.39 (t, 2H, $J$ = 6.9 Hz), 1.84 (quin, 2H, $J$ = 7.5 Hz), 1.74 (quin, 2H, $J$ = 7.5 Hz), 1.48-1.29 (m, 8H); $^{13}$C NMR ($CDCl_3$, 100 MHz) $\delta$/ppm: 164.1 (s, 2C), 133.8 (d, 2C), 131.5 (s), 131.1 (d, 2C), 128.1 (s), 126.9 (d, 2C), 122.7 (s, 2C), 40.3 (t), 33.9 (t), 32.7 (t), 29.1 (t), 28.6 (t), 28.1 (t), 28.0 (t), 26.9 (t); UPLC-MS/UV: method ②, $t_R$ = 1.53 min, $m/z$ = 388.09 $[M+H]^+$, found 388.15, 390.15.

**General procedure for the preparation of *N*-(ω-hydroxyalkyl)-1,8-naphthalimide (3)**

*Caution should be exercised in handling HMPA at all stages of this procedure.*

In a round bottom flask was placed bromoalkylnaphthalimide **2** (1 mmol), hexamethylphosphoramide (HMPA, 4 mL/mmol **2**) and $H_2O$ (45 mmol). The resulting solution was heated to 100 °C and stirred over 17 h. The reaction mixture was cooled to rt, transferred to a separation funnel and diluted with water (15 mL). The resulting suspension was extracted with diethyl ether (3×10 mL). The combined ethereal extracts were washed sequentially with aqueous HCl (1M, 2×20 mL), saturated aqueous solution of $NaHCO_3$ (1×30 mL), aqueous solution of LiCl (5 %, 1×20 mL) and brine (1×15 mL). The solution was dried over anhydrous $Na_2SO_4$, filtered and the solvent was removed on a rotary evaporator. The crude product was used without further purification in the next synthetic step.

***N*-(4-hydroxybut-1-yl)-1,8-naphthalimide (3a)**

Prepared according to the general procedure from bromide **2a** (3.63 g, 10.9 mmol) and $H_2O$ (8.9 mL) in HMPA. The reaction after work up furnished 2.23 g (76%) of the product in the form of colorless solid. NMR characterization is in accordance with the data from literature precedent.[2]

mp 89-90 °C (lit. 111-113 °C);[2] IR (ATR) $\tilde{\nu}$/cm$^{-1}$: 3509 (Ar C–H), 3314 (O–H), 2937, 2872 (C–H), 1692 (Ar C=C), 1652 (C=O), 1587 (C–N amide); $^1$H NMR ($CDCl_3$, 400 MHz) $\delta$/ppm: 8.58 (dd, 2H, $J$ = 7.3 Hz, $J$ = 1.1 Hz), 8.20 (dd, 2H, $J$ = 8.3 Hz, $J$ = 1.0 Hz), 7.77-7.72 (m, 2H), 4.23 (t, 2H, $J$ = 7.3 Hz), 3.75 (t, 2H, $J$ = 6.2 Hz), 1.88 (s, 1H), 1.87-1.80 (m, 2H), 1.70 (quin, 2H, J = 7.3 Hz); $^{13}$C NMR ($CDCl_3$, 100 MHz) $\delta$/ppm: 164.2 (s, 2C), 133.9 (d, 2C), 131.5 (s), 131.2 (d, 2C), 128.0 (s), 126.9 (d, 2C), 122.5 (s, 2C), 62.4 (t), 39.9 (t), 29.9 (t), 24.4 (t); UPLC-MS/UV: method ②, $t_R$ = 0.87 min, $m/z$ = 270.11 $[M+H]^+$, found 270.14.

**N-(5-hydroxypent-1-yl)-1,8-naphthalimide (3b)**

Prepared according to the general procedure from bromide **2b** (3.93 g, 11.4 mmol) and $H_2O$ (9.2 mL) in HMPA. The reaction after work up furnished 2.85 g (89%) of the product in the form of colorless solid. NMR characterization is in accordance with the data from literature precedent.[2]

mp 92-93 °C (lit. 94-96 °C);[2] IR (ATR) $\tilde{\nu}$/cm$^{-1}$: 3397 (O–H), 3062 (Ar C–H), 2938, 2858 (C–H), 1698 (Ar C=C), 1661 (C=O), 1587 (C–N amide); $^1$H NMR ($CDCl_3$, 300 MHz) $\delta$/ppm: 8.60 (dd, 2H, $J$ = 7.3 Hz, $J$ = 1.1 Hz), 8.21 (dd, 2H, $J$ = 8.4 Hz, $J$ = 1.0 Hz), 7.79-7.72 (m, 2H), 4.20 (t, 2H, $J$ = 7.5 Hz), 3.67 (t, 2H, $J$ = 6.4 Hz), 1.79 (quin, 2H, $J$ = 7.4 Hz), 1.73-1.61 (m, 2H), 1.57-1.46 (m, 2H), 1.35 (br. s, 1H); $^{13}$C NMR ($CDCl_3$, 100 MHz) $\delta$/ppm: 164.2 (s, 2C), 133.8 (d, 2C), 131.5 (s), 131.2 (d, 2C), 128.1 (s), 126.9 (d, 2C), 122.6 (s, 2C), 62.7 (t), 40.2 (t), 32.3 (t), 27.8 (t), 23.2 (t); UPLC-MS/UV: method ②, $t_R$ = 0.94 min, $m/z$ = 284.13 [M+H]$^+$, found 284.16.

**N-(6-hydroxyhex-1-yl)-1,8-naphthalimide (3c)**

Prepared according to the general procedure from bromide **2c** (6.25 g, 17.4 mmol) and $H_2O$ (14.1 mL) in HMPA. The reaction after work up furnished 3.92 g (76%) of the product in the form of colorless solid. NMR characterization is in accordance with the data from literature precedent.[3]

mp 70-71 °C; IR (ATR) $\tilde{\nu}$/cm$^{-1}$: 3358 (O–H), 3285 (Ar C–H), 2929, 2858 (C–H), 1697 (Ar C=C), 1659 (C=O), 1590 (C–N amide); $^1$H NMR ($CDCl_3$, 400 MHz) $\delta$/ppm: 8.59 (dd, 2H, $J$ = 7.3 Hz, $J$ = 1.1 Hz), 8.20 (dd, 2H, $J$ = 8.4 Hz, $J$ = 1.0 Hz), 7.77-7.72 (m, 2H), 4.18 (t, 2H, $J$ = 7.5 Hz), 3.65 (t, 2H, $J$ = 6.5 Hz), 1.77-1.72 (m, 2H), 1.65-1.54 (m, 2H), 1.49-1.43 (m, 4H), 1.42 (br. s, 1H); $^{13}$C NMR ($CDCl_3$, 100 MHz) $\delta$/ppm: 164.2 (s, 2C), 133.8 (d, 2C), 131.5 (s), 131.1 (d, 2C), 128.1 (s), 126.9 (d, 2C), 122.6 (s, 2C), 62.7 (t), 40.2 (t), 32.5 (t), 27.9 (t), 26.6 (t), 25.2 (t); UPLC-MS/UV: method ②, $t_R$ = 1.01 min, $m/z$ = 298.14 [M+H]$^+$, found 298.17.

**N-(7-hydroxyhept-1-yl)-1,8-naphthalimide (3d)**

Prepared according to the general procedure from bromide **2d** (4.78 g, 12.8 mmol) and $H_2O$ (10.4 mL) in HMPA. The reaction after work up furnished 3.88 g (98%) of the product in the form of colorless solid.

mp 70-71 °C; IR (ATR) $\tilde{\nu}$/cm$^{-1}$: 3526 (O–H), 2929, 2853 (C–H), 1689 (Ar C=C), 1650 (C=O), 1589 (C–N amide); $^1$H NMR ($CDCl_3$, 400 MHz) $\delta$/ppm: 8.59 (dd, 2H, $J$ = 7.3 Hz, $J$ = 1.1 Hz), 8.20 (dd, 2H, $J$ = 8.4 Hz, $J$ = 1.0 Hz), 7.78-7.69 (m, 2H), 4.17 (t, 2H, $J$ = 7.6 Hz), 3.64 (t, 2H, $J$ = 6.6 Hz), 1.73 (quin, 2H, $J$ = 7.4 Hz), 1.57 (quin, 2H, $J$ = 6.9 Hz), 1.49-1.30 (m, 6H); $^{13}$C NMR ($CDCl_3$, 100 MHz) $\delta$/ppm: 164.2 (s, 2C), 133.8 (d, 2C), 131.5 (s), 131.1 (d, 2C), 128.1

(s), 126.9 (d, 2C), 122.7 (s, 2C), 62.9 (t), 40.3 (t), 32.6 (t), 28.9 (t), 27.9 (t), 26.9 (t), 25.5 (t); UPLC-MS/UV: method ②, $t_R$ = 1.09 min, $m/z$ = 312.16 [M+H]$^+$, found 312.19.

**$N$-(7-hydroxyoct-1-yl)-1,8-naphthalimide (3e)**

Prepared according to the general procedure from bromide **2e** (6.88 g, 17.7 mmol) and $H_2O$ (14.4 mL) in HMPA. The reaction after work up furnished 5.65 g (98%) of the product in the form of colorless solid.

mp 66-67 °C; IR (ATR) $\tilde{\nu}$/cm$^{-1}$: 3331 (O–H), 3252, 3074 (Ar C–H), 2926, 2858 (C–H), 1699 (Ar C=C), 1662 (C=O), 1588 (C–N amide); $^1$H NMR ($CDCl_3$, 400 MHz) $\delta$/ppm: 8.59 (dd, 2H, $J$ = 7.3 Hz, $J$ = 1.1 Hz), 8.20 (dd, 2H, $J$ = 8.4 Hz, $J$ = 1.0 Hz), 7.77-7.72 (m, 2H), 4.17 (t, 2H, $J$ = 7.6 Hz), 3.63 (t, 2H, $J$ = 6.6 Hz), 1.73 (quin, 2H, $J$ = 7.6 Hz), 1.57 (quin, 2H, $J$ = 6.7 Hz), 1.47-1.36 (m, 8H); $^{13}$C NMR ($CDCl_3$, 100 MHz) $\delta$/ppm: 164.1 (s, 2C), 133.8 (d, 2C), 131.5 (s), 131.1 (d, 2C), 128.1 (s), 126.8 (d, 2C), 122.7 (s, 2C), 62.9 (t), 40.4 (t), 32.7 (t), 29.2 (t, 2C), 28.0 (t), 26.9 (t), 25.6 (t); UPLC-MS/UV: method ②, $t_R$ = 1.17 min, $m/z$ = 326.18 [M+H]$^+$, found 326.20.

**Preparation of 2-benzyloxy-3-bromonaphthalene (5)[4]**

A round bottom flask (250 mL) was charged with 2-bromo-3-hydroxynaphthalene (8.88 g, 39.8 mmol), $K_2CO_3$, (11.04 g, 79.9 mmol), DMF (120 mL) and benzyl bromide (7.20 mL, 60.62 mmol). The resulting suspension was heated to 60 °C for 2.5 h (the course of the reaction was monitored by TLC). The reaction mixture was cooled to rt, transferred to a separation funnel and diluted with water (500 mL). The resulting suspension was extracted with $CH_2Cl_2$ (3×100 mL). The combined organic layers were washed sequentially with aqueous solution of LiCl (5 %, 3×200 mL) and brine (3×200 mL). The solution was dried over anhydrous $MgSO_4$, filtered and the solvent was removed on a rotary evaporator. The residue was purified on a silica gel column using DCM (0 to 33%)/cyclohexane as eluent to afford the pure product 12.35 g (99%) in the form of colorless solid. NMR characterization is in accordance with the data from literature precedent.[4]

mp 83-84 °C (lit. 83-84 °C);[4] IR (ATR) $\tilde{\nu}$/cm$^{-1}$: 3031 (Ar C–H), 2872 (C–H), 1379 (C=C), 1245 (C–O), 1019 (C–O–C), 753 (C–Br); $^1$H NMR ($CDCl_3$, 500 MHz) $\delta$/ppm: 8.06 (s, 1H), 7.69 (d, 2H, $J$ = 9.0 Hz), 7,54 (d, 2H, $J$ = 7.5 Hz), 7.46-7.38 (m, 3H), 7.38-7.31 (m, 2H), 7.22 (s, 1H), 5.27 (s, 2H); $^{13}$C NMR ($CDCl_3$, 125 MHz) $\delta$/ppm: 152.6 (s), 136.4 (s), 133.4 (s), 132.4 (d), 129.5 (s), 128.6 (d, 2C), 127.9 (d), 127.0 (d, 2C), 126.7 (d), 126.6 (d, 2C), 124.5 (d), 113.9 (s), 108.3 (d), 70.6 (t).

**Preparation of the Grignard reagent 6**

A well-dried three neck round bottom flask (250 mL), equipped with a condenser, a dropping funnel and septum, under Ar atmosphere was charged with freshly activated Mg (0.53 g, 21.8 mmol), dry THF (5.0 mL) and a few crystals of $I_2$. A solution, prepared by dissolving **5** (4.50 g, 14.4. mmol) in dry THF (80 mL), was placed in to the dropping funnel. A few drops of the solution from the dropping funnel were added to the suspension in the flask, and the mixture was heated to the reflux temperature to initiate the reaction. After disappearance of the brown color of iodine, the remaining solution from the dropping funnel was added to the mixture at rt over 1 h. After the addition was completed, the reaction mixture was heated to reflux temperature for 3-4 h and the course of the reaction was monitored by UPLC-MS/UV (method ③). The reaction mixture was cooled down to rt and used as is in the next step.

**Methods for UPLC-MS/UV analysis and preparative HPLC-MS/UV separations**

**UPLC-MS/UV analyses** were conducted on a Waters Acquity UPLC coupled with a SQD mass spectrometer. The following analysis conditions were used

<u>LC conditions</u>

Methods ① and ②: Waters Acquity UPLC BEH C18 50×2.1 mm (1.7 μm)

Method ③: Waters Acquity UPLC CSH C18 50×2.1 mm (1.7 μm)

Column temperature: 40 °C

Mobile phase:

Method ①: A: 0.1% HCOOH (aq);
B: 0.1% HCOOH in $CH_3CN$

Methods ② and ③ : A: 10 mmol dm$^{-3}$ $NH_4HCO_3$(aq) adjusted to pH = 10 with $NH_3$ (aq);
B: $CH_3CN$

Injected volume: 1.0 μL

Temperature of the sample tray: 15 °C

Table S1. Gradient for the UPLC methods ① and ②:

| Time/ min | Flow/mLmin$^{-1}$ | % A | % B |
|---|---|---|---|
| 0 | 0.9 | 97 | 3 |
| 1.5 | 0.9 | 0 | 100 |
| 1.9 | 0.9 | 0 | 100 |
| 2.0 | 0.05 | 97 | 3 |

Table S2. Gradient for the UPLC method ③:

| Time/ min | Flow/mLmin$^{-1}$ | % A | % B |
|---|---|---|---|
| 0 | 0.9 | 95 | 5 |
| 2 | 0.9 | 0 | 100 |
| 3.9 | 0.9 | 0 | 100 |
| 3.99 | 0.9 | 95 | 5 |
| 4 | 0.3 | 95 | 5 |

The duration of the analyses: 2.0 min (① i ②); 4,0 min (③)

Diluent: $CH_3CN$-$H_2O$ (1:1), $CH_3OH$ or DMSO

Detector conditions:

UV detector:

- PDA: 210-340 nm (UV-TIC spectrum is a sum of signals 210-340 nm)

MS detector:

- ionization mode: positive and negative alternating electrospray ($ESI^+$/$ESI^-$)
- Mr: 90-1000 AMU (① i ②) and 100-1500 AMU (③)
- source temperature: 150 °C; desolvation temperature: 350 °C.

**Preparative HPLC-MS/UV separations** were conducted on a Waters MDAP system equipped with a ZQ mass spectrometer. The instrument setup was the following:

LC conditions:

Column: Waters XBridge Prep. MS C18 OBD 150×30 mm (5 µm) at rt

Mobile phase: A: 10 mmol dm$^{-3}$ $NH_4HCO_3$(aq) adjusted to pH=10 with $NH_3$ (aq);

B: $CH_3CN$

Injected volume: max 950 µL

Table S3. Gradient for the preparative HPLC

| Time/ min | Flow/mLmin$^{-1}$ | % A | % B |
|---|---|---|---|
| 0 | 50 | 70 | 30 |
| 1 | 50 | 35 | 65 |
| 10 | 50 | 0 | 100 |
| 15 | 50 | 0 | 100 |

Duration of the separation 15 min

Diluent: $CH_3CN$-$H_2O$ (1:1), $CH_3OH$ or DMSO

<u>Detector conditions:</u>

UV detector

- PDA scan: 210-600 nm (UV-TIC spectrum is a sum of signals 210-600 nm)

MS detector:

- ionization mode: positive and negative electrospray ($ESI^+$/$ESI^-$)
- voltage scan: 100-1000 AMU

**Determination of the quantum yield of photomethanolysis**

The **number of absorbed photons for the $KIO_3$/KI** was calculated from:

$$n(\text{absorbed photons}) = \frac{\Delta A_{352} \times V_{irr}}{\varepsilon_{352} \times \ell \times \Phi_{lit.}} \quad \text{(S1)}$$

where:

| | |
|---|---|
| $\Delta A_{352}$ | absorbance difference at 352 nm for the irradiated and non-irradiated sample |
| $V_{irr}$ | volume of the solution which was irradiated |
| $\varepsilon_{352}$ | molar absorption coefficient for $I_3^-$ in solution which contains iodides and iodates, 27600 $M^{-1}$ $cm^{-1}$ |
| $l$ | length of the optical path (1 cm in all experiments) |
| $\Phi_{lit.}$ | quantum yield ($\Phi_{254} \approx 0.74$), the precise value was calculated from S2 and S3 (depending on the iodine concentration and temperature) |

$$c(I^-) = A_{300}/1.061 \quad [M] \quad \text{(S2)}$$

$$\Phi_{lit} = 0.75 \times [1 + 0.02(T - 20.7)] \times [1+0.23(c(I^-) - 0.577)] \quad \text{(S3)}$$

**For the absorbances in the range 0.4-0.8 the number of absorbed photons was calculated according to:**

$$n(\text{absorbed photons}) = n(\text{total photons}) \times (1\text{-}T) \quad \text{(S4)}$$

**The quantum yield for the photohydrolysis was calculated according to**:

$$\Phi = \frac{A_{254} \cdot V_{irr} \cdot x(\text{photoproduct})_{HPLC}}{\varepsilon_{254} \cdot \ell \cdot n(\text{total photons}) \cdot (1 - T_{254})} \quad \text{(S5)}$$

where:

| | |
|---|---|
| $x(\text{photoproduct})_{HPLC}$ | conversion of reactant to photoproduct determined by HPLC |
| $T_{254}$ | transmittance of light at 254 nm |

## 2. UV-vis and fluorescence data

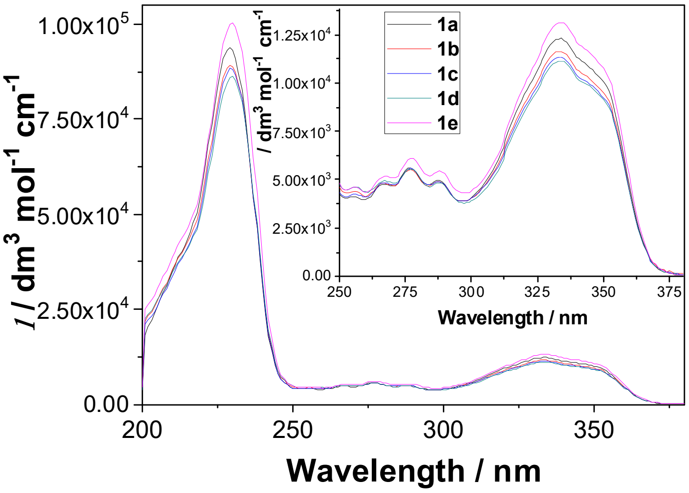


Fig S1. Absorption spectra of **1** in $CH_3OH$-$H_2O$ (4:1).

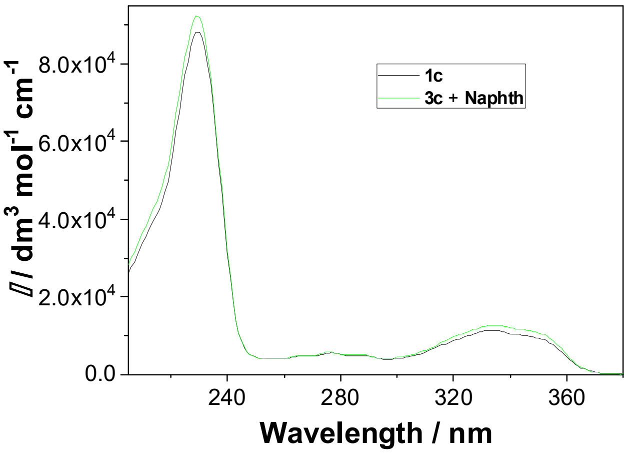


Fig S2. Absorption spectra of **1c** in $CH_3OH$-$H_2O$ (4:1), and a sum of absorption spectra of **3c** and Naphth (Eq. 1 in the manuscript).

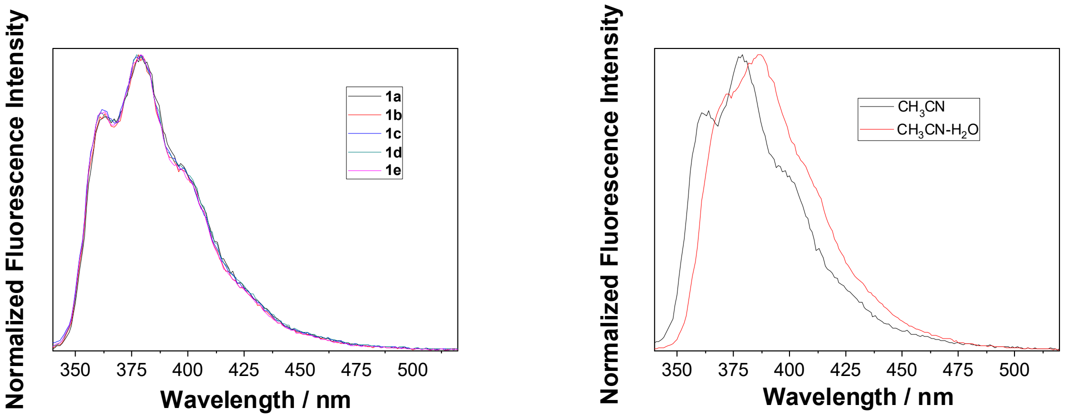


Fig S3. Left: Normalized fluorescence spectra ($\lambda_{ex}$ = 320 nm) of **1** in $CH_3CN$; Right: Normalized fluorescence spectra of **1c** in $CH_3CN$ and $CH_3CN$-$H_2O$.

<u>Fluorescence quantum yield</u> was calculated according to:

$$\Phi_f = \Phi_{Ref}\left(\frac{n}{n_R}\right)^2 \frac{I}{I_R}\frac{1-10^{-A_R}}{1-10^{-A}} \qquad \text{(S6)}$$

$\Phi_f$ and $\Phi_{Ref}$ – fluorescence quantum yield of compound and the reference;

$n$ and $n_R$ – refractive index of the solvent in which compound or the reference was dissolved;

$A$ and $A_R$ – absorbance of the compound and the reference at the excitation wavelength;

$I$ and $I_R$ – area under emission curve of the compound and the reference.

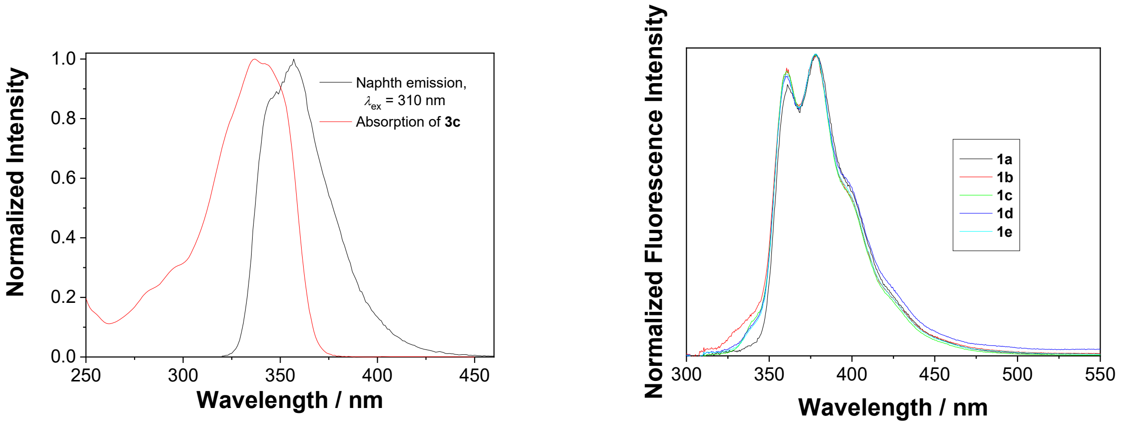


Fig S4. Left: Normalized emission spectrum of Naphth and absorption spectrum of **3c** in $CH_3CN$. A significant spectral overlap enables FRET between two chromophores. Right: Normalized fluorescence spectra of **1** in $CH_3CN$ ($\lambda_{exc}$ = 280 nm). A shoulder in the emission spectra between 300 and 350 nm corresponds to the emission from naphthol part of the molecule.

Fluorescence decays were obtained with time-correlated single-photon counting method on a FS5 Edinburgh Instruments spectrometer equipped for time correlated single photon counting method (TC-SPC). Pulsed LEDs at 280 nm or 340 nm were used for the excitation (pulse duration ≈ 800 ps). Fluorescence signals were monitored over 1023 channels with the time increment of ≈ 20 ps/channel. The decays were collected until they reached 1000 counts in the peak channel. The histograms were analyzed by a nonlinear least-squares deconvolution method using. The quality of the fit was judged by the reduced $\chi^2$ being close to unity and the random distribution of the weighted residuals. Fluorescence decays were fit to a sum of exponentials using the following expression:

$$F\,(t) = \alpha_1 \exp\left(-\frac{t}{\tau_1}\right) + \alpha_2 \exp\left(-\frac{t}{\tau_2}\right) + \alpha_3 \exp\left(-\frac{t}{\tau_3}\right) + \ \ldots \qquad \text{(S7)}$$

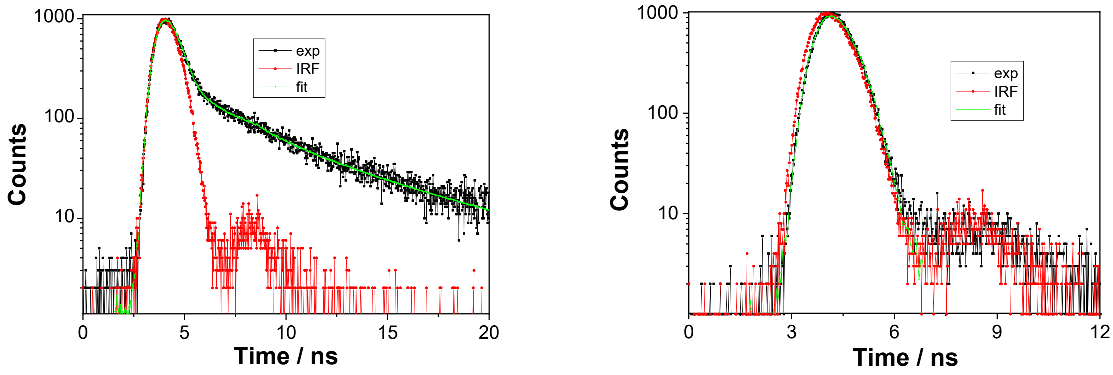


Fig S5. Decay of fluorescence for **1a** in $CH_3CN$ at 340 nm upon excitation at 280 nm (left); and decay of fluorescence at 390 nm upon excitation at 280 nm (right).

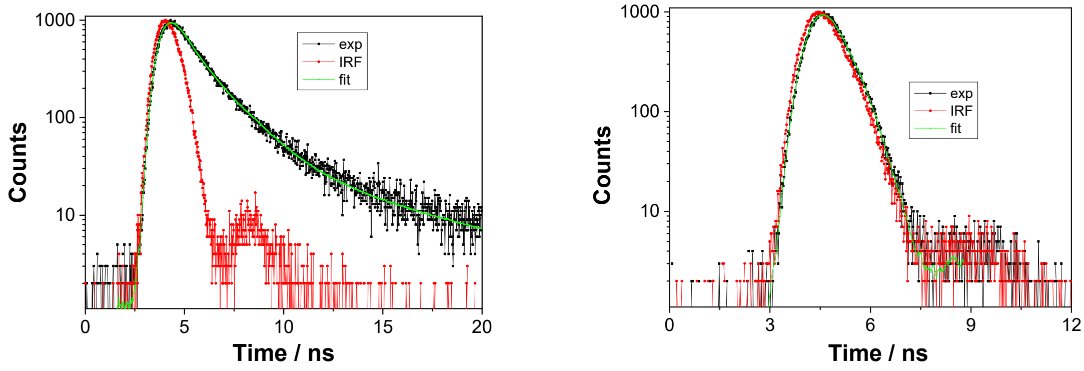


Fig S6. Decay of fluorescence for **1b** in $CH_3CN$ at 340 nm upon excitation at 280 nm (left); and decay of fluorescence at 390 nm upon excitation at 340 nm (right).

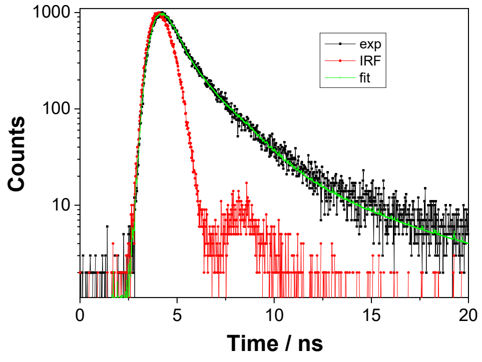


Fig S7. Decay of fluorescence for **1c** in $CH_3CN$ at 340 nm upon excitation at 280 nm.

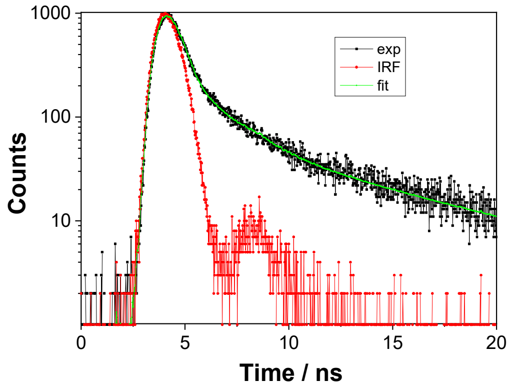


Fig S8. Decay of fluorescence for **1d** in $CH_3CN$ at 340 nm upon excitation at 280 nm.

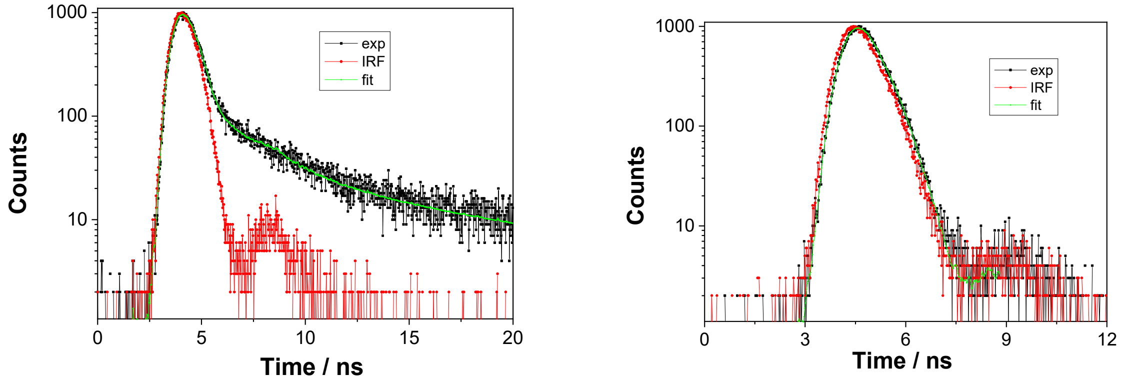


Fig S9. Decay of fluorescence for **1e** in $CH_3CN$ at 340 nm upon excitation at 280 nm (left); and decay of fluorescence at 390 nm upon excitation at 340 nm (right).

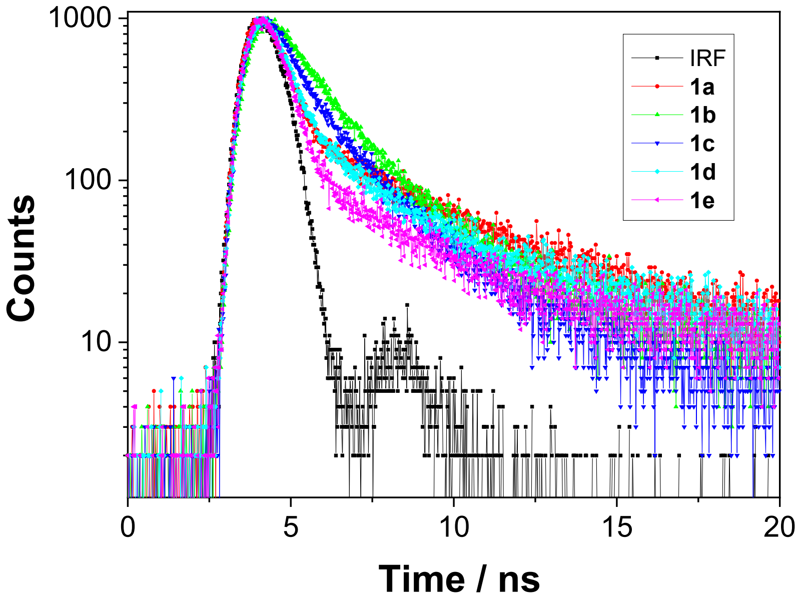


Fig S10. Decay of fluorescence for **1a**-**1e** in $CH_3CN$ at 340 nm upon excitation at 280 nm.

Table S4. Fluorescence decay times and pre-exponential factors measured by TC-SPC for **1** in $CH_3CN$. The excitation wavelength was 280 nm and the decays were collected at 340 nm, or the excitation was 340 nm and the decays were collected at 390 nm.

| Comp. | $\lambda_{exc}$ | $\tau$ / ns | Pre-exponential factors | $\chi^2$ | $\tau_{av}$ / ns |
|---|---|---|---|---|---|
| **1a** | 280 | ≈ 0.04<br>2.2 ± 0.1<br>7.50 ± 0.07 | 0.50<br>0.20<br>0.30 | 1.02 | 2.71 |
| | 280 ($\lambda_{em}$ 390) | ≈ 0.04 | 1.0 | 1.39 | - |
| | 340 | ≈ 0.08<br>1.2 ± 0.1 | 0.98<br>0.02 | 1.15 | - |
| **1b** | 280 | ≈ 0.04<br>1.51 ± 0.03<br>7.4 ± 0.5 | 0.25<br>0.50<br>0.15 | 1.02 | 1.87 |
| | 340 | 0.13 ± 0.01 | 1.0 | 0.89 | - |
| **1c** | 280 | ≈ 0.01<br>1.54 ± 0.03<br>6.5 ± 0.5 | 0.34<br>0.54<br>0.12 | 1.20 | 1.61 |
| | 340 | 0.10 ± 0.01<br>1.7 ± 0.7 | 0.98<br>0.02 | 1.15 | - |
| **1d** | 280 | ≈ 0.04<br>1.57 ± 0.07<br>8.4 ± 0.4 | 0.50<br>0.24<br>0.26 | 0.93 | 2.58 |
| | 340 | 0.12 ± 0.08<br>1.3 ± 0.3 | 0.97<br>0.03 | 1.13 | - |
| **1e** | 280 | ≈ 0.04<br>2.1 ± 0.1<br>12 ± 2 | 0.63<br>0.17<br>0.20 | 1.08 | 2.78 |
| | 340 | 0.13 ± 0.07 | 1.0 | 0.90 | - |
| **Naphth** | 280 | 9.07 ± 0.03 | 1.0 | 0.98 | - |

At 280 nm, both fluorophores are excited, the naphthol and the naphthalimide. However, at 340 nm the fluorescence is mostly detected from the naphthol. The decay of fluorescence for Naphth can be described by single exponential function with the lifetime of 9.07 ns. On the contrary, decay of fluorescence for **1a**-**1e** at 340 nm was fit to a sum of three exponents. For the sum of exponents, the average lifetime was calculated according to:

$$\tau_{av} = \Sigma\alpha_i \tau_i \quad \text{(S8)}$$

From the shortening of decay time at 340 nm for **1a**-**1e**, compared to the lifetime of Naphth, the efficiency of FRET ($\Phi_{FRET}$) from the naphthol to the naphthalimide can be calculated according to:

$$\Phi_{\mathrm{FRET}} = 1 - \frac{\tau_{av}}{\tau^0_{Naphth}} \quad \text{(S9)}$$

Upon excitation at 340 nm, almost only naphthalimide is excited and only in some cases it leads also to the excitation of the naphthol. Fluorescence decays were fit to single exponential function or a sum of two exponents with a small contribution of longer decay time. Short decay times with lifetimes 80-130 ps were assigned to the singlet state of naphthalimide, whereas the long decay time of 1.2-1.7 ns with small contribution, detected for some compounds only, corresponds to the decay of naphthol. Short decay time of the naphthol in this case is also due to FRET.

## 3. Laser Flash Photolysis

A stock solution of **1a** in $CH_3CN$, $c = 3.77 \times 10^{-4}$ M was prepared by dissolving 1.55 mg in 10 mL $CH_3CN$.

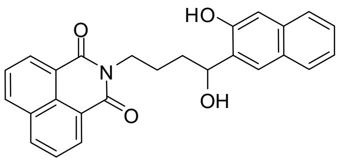

For the LFP measurement, the stock was diluted, $c = 7.23 \times 10^{-5}$ M, $A_{266} = 0.37$.

LFP measurements were performed on an Edinburgh Instruments spectrometer. For the excitation, a Quantel YAG laser fourth harmonic ($\lambda_{exc}$ = 266 nm) was used. The energy of the laser pulse was set to 20 mJ. Prior to the measurements, the solution was purged with a stream of Ar or $O_2$ for 15 min. Static cells were used for the measurements, and the solution was replaced frequently to assure that the transients are not formed from photoproducts.

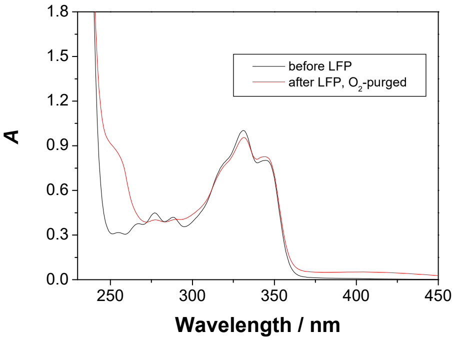


Fig S11. UV-vis spectra of **1a** in $O_2$-purged $CH_3CN$ solution before and after the LFP measurements.

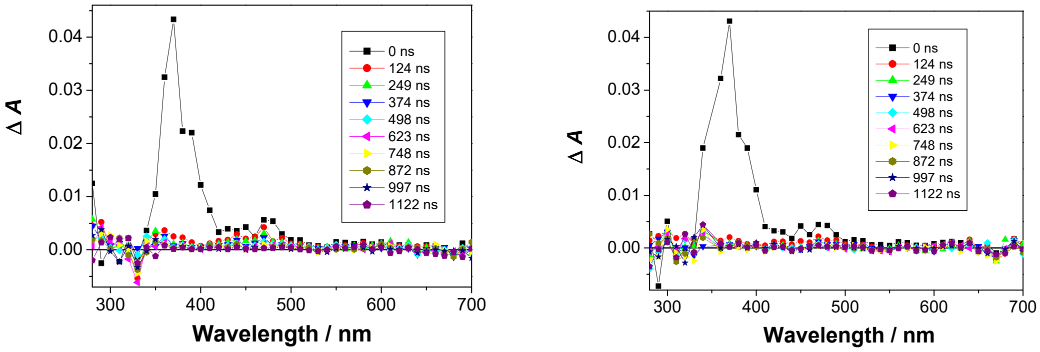


Fig S12. Transient absorption spectra of **1a** in Ar-purged (left) and $O_2$-purged (right) $CH_3CN$ solution ($\lambda_{ex}$ = 266 nm).

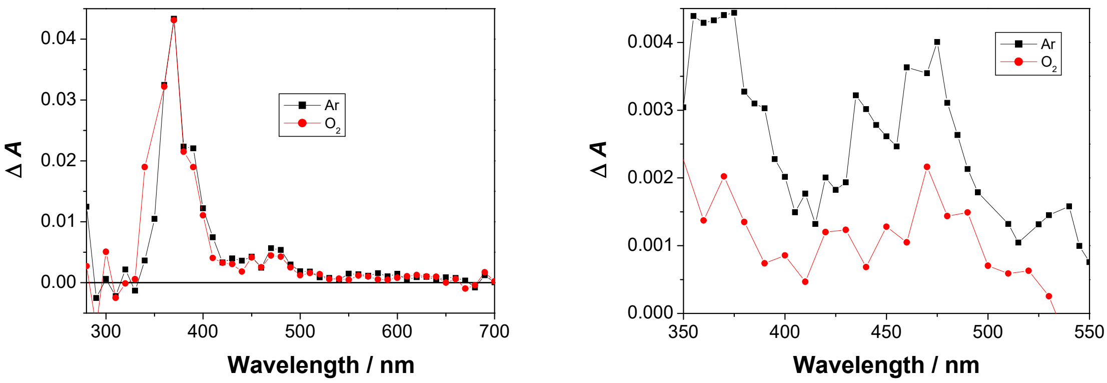


Fig S13. Transient absorption spectra of **1a** in Ar-purged and $O_2$-purged (right) $CH_3CN$ solution collected immediately after the laser pulse (left), or after a delay of 120 ns (right) ($\lambda_{ex}$ = 266 nm).

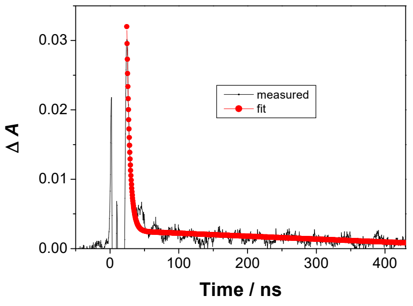


Fig S14. Decay of transient absorbance of **1a** at 400 nm in Ar-purged $CH_3CN$ solution. The fitting revealed two decay times, $\tau = 2.6 \pm 0.1$ ns and $\tau = 23.1 \pm 0.8$ ns; tentatively assigned to phenol radical-cation and naphthalimide radical-anion.

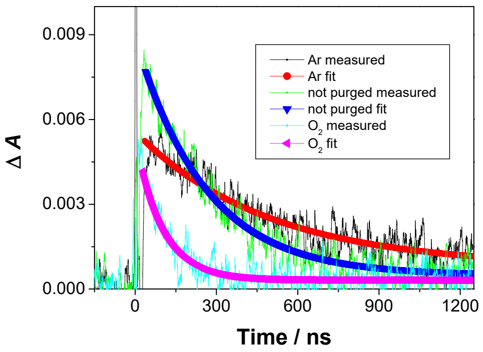


Fig S15. Decay of transient absorbance at 470 nm in Ar-purged, not purged and $O_2$-purged $CH_3CN$ solution of **1a**. The fitting revealed decay times, $\tau_{Ar} = 460 \pm 20$ ns; $\tau_{air} = 290 \pm 20$ ns and $\tau_{O2} = 100 \pm 3$ ns. The transient is quenched by $O_2$, $k_q = (8.7 \pm 0.7)\times10^8$ $M^{-1}$ $s^{-1}$; the transient was assigned to the naphthalimide triplet excited state.

LFP experiment was also performed by exciting sample at 355 nm (pulse energy 20 mJ). The same concentration was prepared as above $c = 7.23 \times 10^{-5}$ M, $A_{355} = 0.28$. Spectra were recorded for Ar- and $O_2$-purged solution.

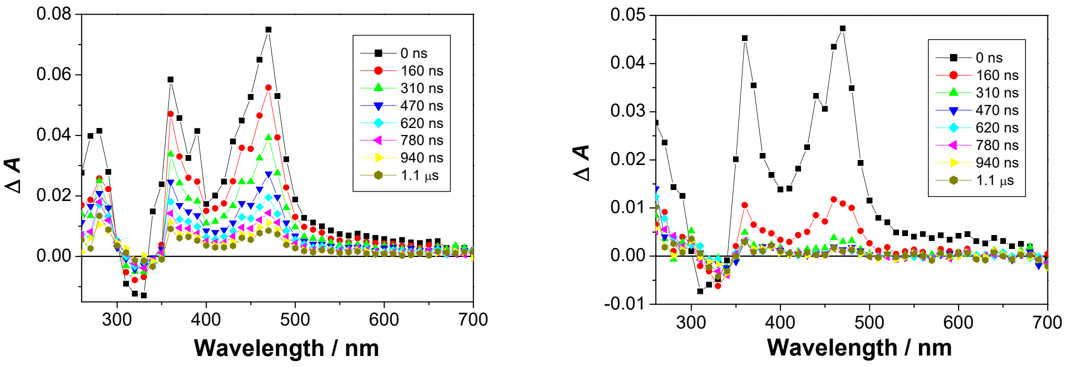


Fig S16. Transient absorption spectra of **1a** in Ar-purged (left) and $O_2$-purged (right) $CH_3CN$ solution ($\lambda_{ex}$ = 355 nm).

## 4. Noncovalent binding to ct-DNA

*Thermal denaturation experiment*

A stock solution of **1a** ($2.0\times10^{-2}$ M) was prepared by dissolving 1.67 mg in 202.9 µL DMSO. A stock solution of ct-DNA in cacodylate buffer (50 mM, pH 7.0) had concentration $1.1\times10^{-3}$ M.

Table S5. Melting temperatures ($T_m$) corresponding to the denaturation of ct-DNA and ct-DNA and mixtures of **1a** and ct-DNA.

| Sample | $r$ [b] | $T_m$ /°C | $\Delta T_m$ /°C |
|---|---|---|---|
| ct-DNA | 0 | 80.1 | - |
| ct-DNA | 0 | 80.1 | - |
| **1a** and ct-DNA | 0.3 | 80.1 | 0 |
| **1a** and ct-DNA | 0.3 | 80.1 | 0 |
| **1a** and ct-DNA + $h\nu$ [c] | 0.3 | 79.1 | -1.0 |

[a] The measurement was conducted in aqueous cacodylate buffer (50 mM, pH 7.0) by monitoring absorbance of the solution at 260 nm. The estimated error in $\Delta T_m$ is ± 0.5 °C. [b] $r$ = [**1a**]/[DNA]. [c] Irradiated at 350 nm for 5 min.

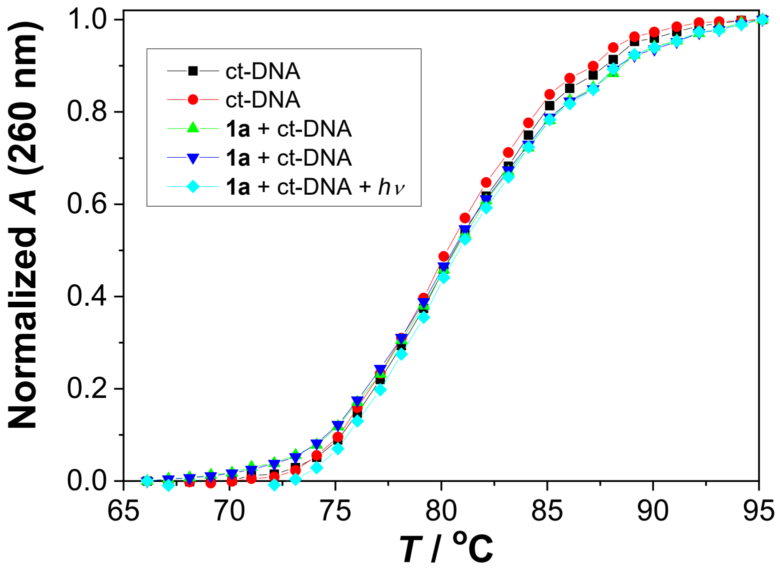


Fig S17. Normalized changes of the absorbance at 260 nm of the solution of ct-DNA and a mixture of ct-DNA and **1a** at $r$ = [**1a**]/[DNA] = 0.3, at different temperatures.

A stock solution of **1e** ($2.0\times10^{-2}$ M) was prepared by dissolving 1.96 mg in 209.6 µL DMSO. A stock solution of ct-DNA in cacodylate buffer (50 mM, pH 7.0) had concentration $1.1\times10^{-3}$ M.

Table S6. Melting temperatures ($T_m$) corresponding to the denaturation of ct-DNA and ct-DNA and mixtures of **1e** and ct-DNA.

| Sample | $r$ [b] | $T_m$ /°C | $\Delta T_m$ /°C |
|---|---|---|---|
| ct-DNA | 0 | 79.0 | - |
| ct-DNA | 0 | 79.0 | - |
| **1e** and ct-DNA | 0.3 | 79.0 | 0 |
| **1e** and ct-DNA | 0.3 | 79.0 | 0 |
| **1e** and ct-DNA + $h\nu$ [c] | 0.3 | 79.0 | 0 |

[a] The measurement was conducted in aqueous cacodylate buffer (50 mM, pH 7.0) by monitoring absorbance of the solution at 260 nm. The estimated error in $\Delta T_m$ is ± 0.5 °C. [b] $r$ = [**1a**]/[DNA]. [c] Irradiated at 350 nm for 5 min.

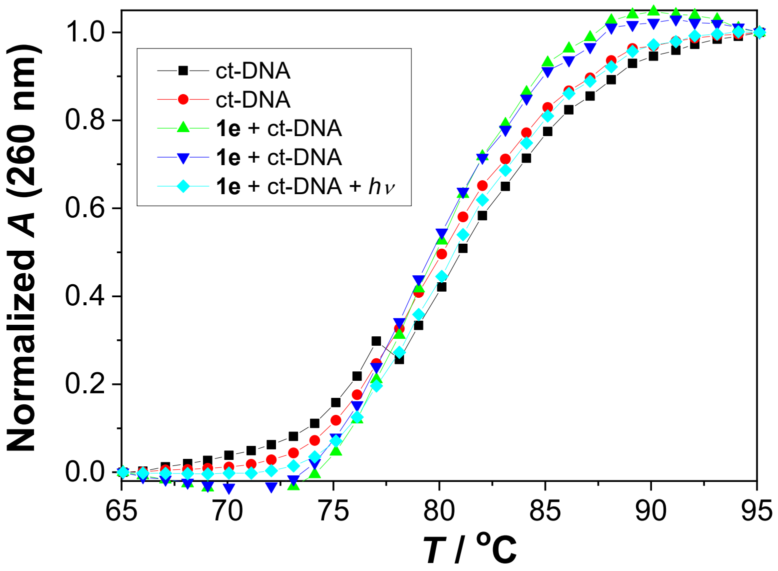


Fig S18. Normalized changes of the absorbance at 260 nm of the solution of ct-DNA and a mixture of ct-DNA and **1e** at $r$ = [**1e**]/[DNA] = 0.3, at different temperatures.

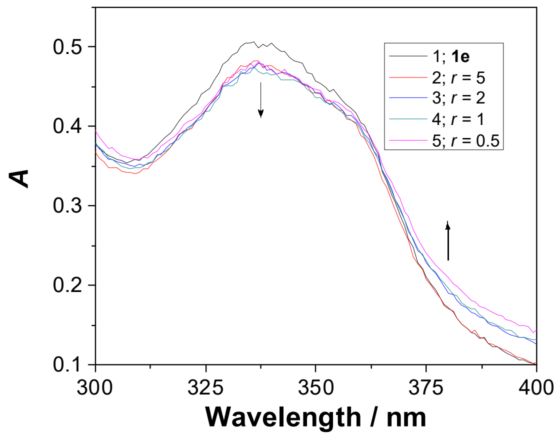


Fig S19. UV-vis spectra of **1e** ($c = 9.0\times10^{-6}$ M), in aqueous cacodylate buffer (50 mM, pH = 7.0) in the presence of ct-DNA at different ratio $r$ = [**1e**]/[DNA]. The measurement was conducted on a Cary 60 spectrometer equipped with a 5 cm pathway probe in a glass beaker (22 mL).

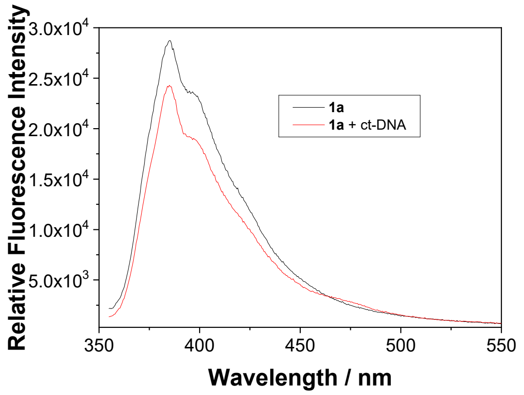


Fig S20. Fluorescence spectra ($\lambda_{exc}$ = 340 nm) of **1a** ($c = 2.0\times10^{-6}$ M), without or in the presence of ct-DNA ($c = 1.5\times10^{-4}$ M). The measurement was conducted in aqueous solution containing 0.05 M sodium cacodylate buffer pH = 7.0, at 25 °C (containing <1% DMSO). The total intensity of fluorescence was quenched 16%.

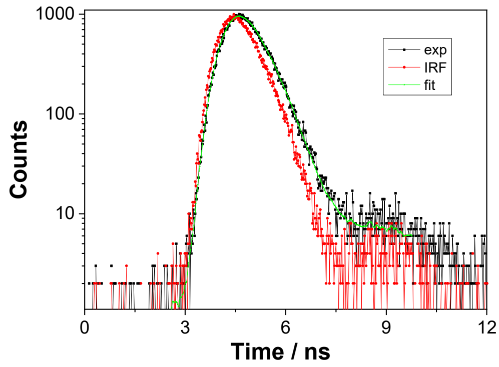


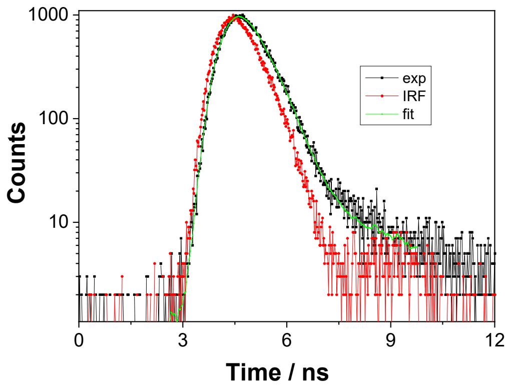


Fig S21. Left: Decay of fluorescence for **1a** ($c = 2.0\times10^{-6}$ M) at 390 nm ($\lambda_{exc}$ = 340 nm), and Right: a mixture of **1a** ($c = 2.0\times10^{-6}$ M) and ct-DNA ($c = 1.5\times10^{-4}$ M). The measurement was conducted in aqueous solution containing 0.05 M sodium cacodylate buffer pH = 7.0, at 25 °C (containing <1% DMSO).

Table S7. Decay times and pre-exponential factors obtained by TC-SPC of **1a** or **1a** and ct-DNA in aqueous solution containing 0.05 M sodium cacodylate buffer pH = 7.0, at 25 °C (containing <1% DMSO). The excitation wavelength was 280 nm and the decays were collected at 340 nm, or the excitation was 340 nm and the decays were collected at 390 nm.

| Comp. | $\lambda_{em}$ | $\tau$ / ns | Pre-exponential factors | $\chi^2$ |
|---|---|---|---|---|
| **1a** | 340 | ≈ 0.10<br>1.60 ± 0.06<br>6.9 ± 0.2 | 0.15<br>0.37<br>0.48 | 1.01 |
| | 390 | ≈ 0.21<br>2.2 ± 0.4 | 0.96<br>0.03 | 0.92 |
| **1a + ct-DNA** | 340 | ≈ 0.17<br>1.90 ± 0.08<br>6.4 ± 0.3 | 0.18<br>0.44<br>0.38 | 1.07 |
| | 390 | ≈ 0.17<br>1.3 ± 0.1 | 0.92<br>0.08 | 0.98 |

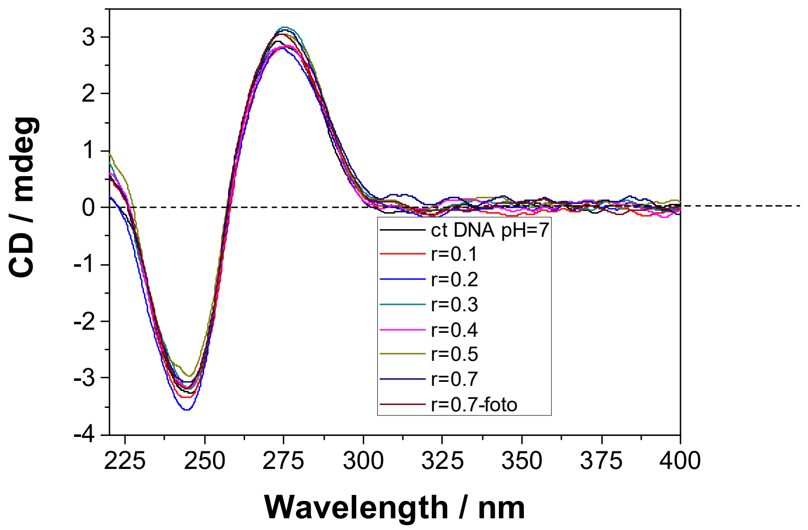


Fig S22. CD spectra of ct-DNA ($c$ = 3×10$^{-5}$ M) in the presence of different concentration of **1a** corresponding to the ratio $r$ = [**1a**]/[ct-DNA] = 0.1-0.7. The measurement was conducted in aqueous solution containing 0.05 M sodium cacodylate buffer pH = 7.0, at 25 °C (containing <1% DMSO).

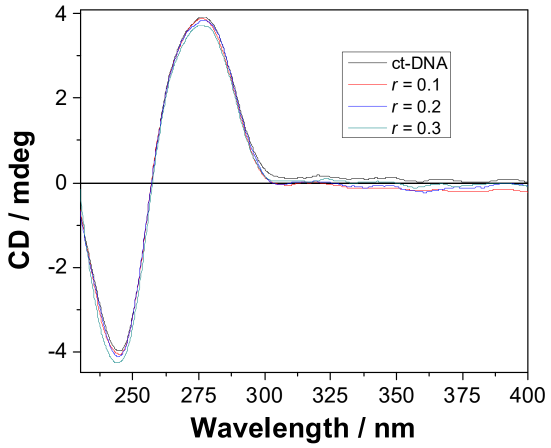


Fig S23. CD spectra of ct-DNA ($c$ = 3×10$^{-5}$ M) in the presence of different concentration of **1e** at different ratios $r$ = [**1e**]/[ct-DNA] = 0.1-0.5. The measurement was conducted in aqueous solution containing 0.05 M sodium cacodylate buffer pH = 7.0, at 25 °C (containing <1% DMSO).

## 5. Noncovalent and covalent binding to BSA

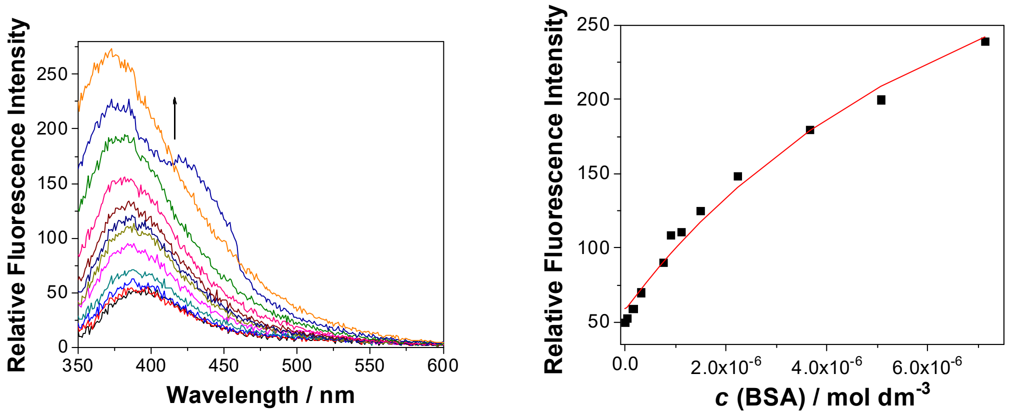


Fig S24. Left: Fluorescence spectra ($\lambda_{exc}$ = 320 nm) of **1e** ($c$ = 1.0×10$^{-6}$ M) in the presence of different concentration of BSA. The measurement was conducted in aqueous solution containing 0.05 M sodium cacodylate buffer at pH = 7.0, at 25 °C (containing <1% DMSO). Right: Dependence of the fluorescence intensity at 390 nm on the BSA concentration. The black points are experimental values and the red line is the fit to the model involving **1e@BSA** in the stoichiometry 1:1.

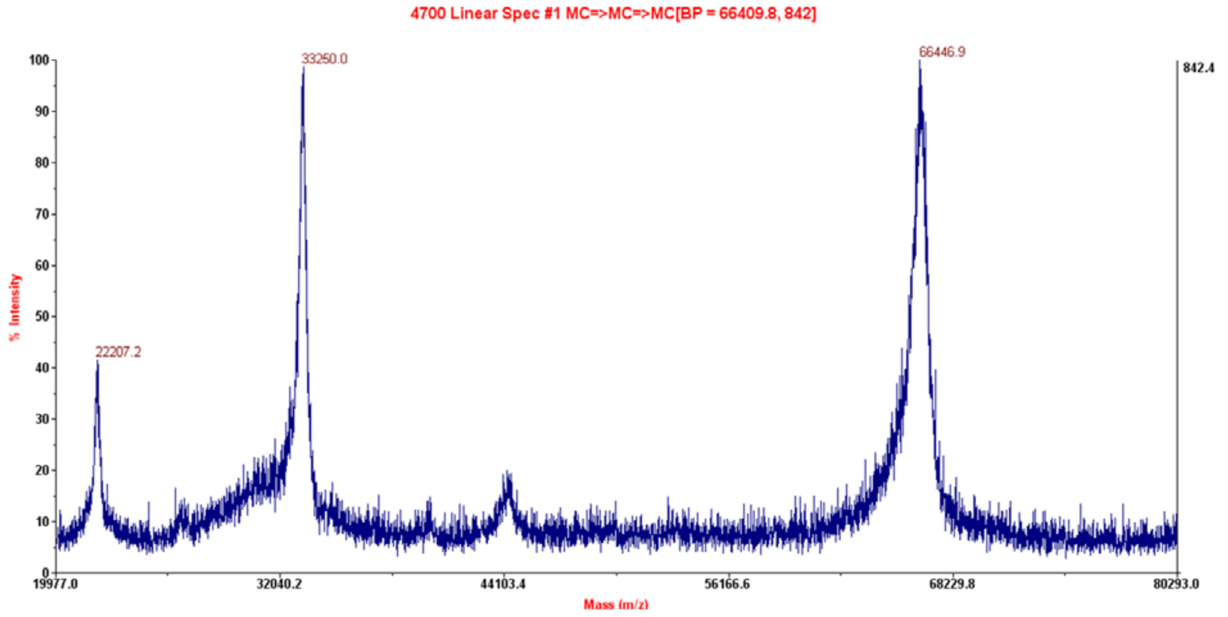


Fig S25. MALDI-MS of BSA

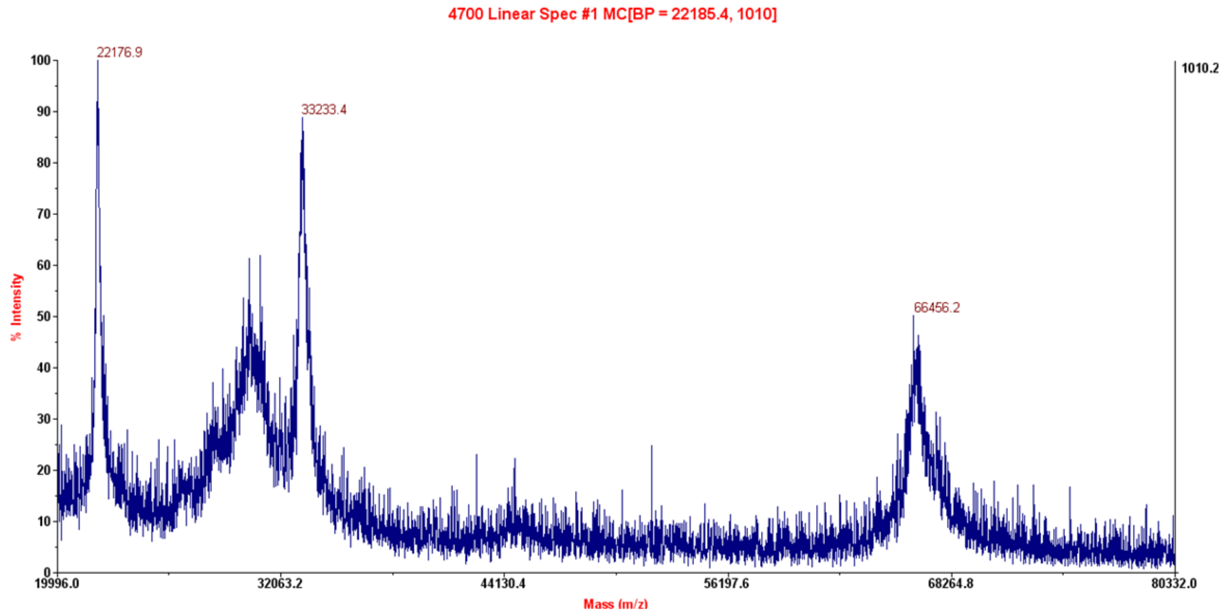


Fig S26. MALDI-MS of **1a@BSA** after the photolysis

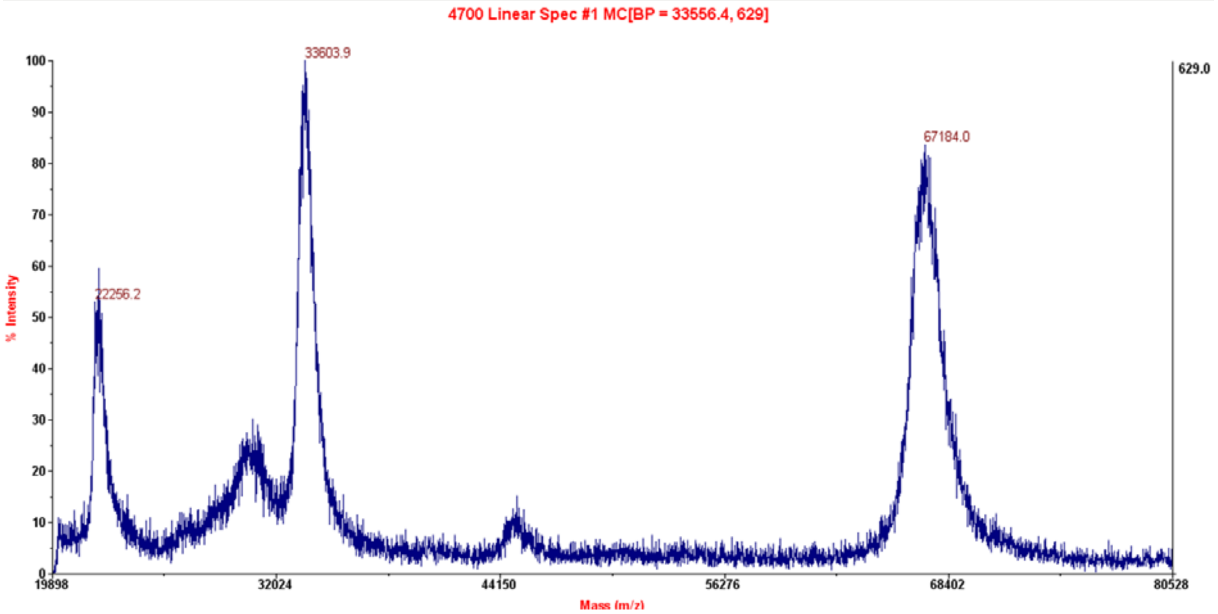


Fig S27. MALDI-MS of **1e@BSA** after the photolysis

Table S8. The detected molecular weight of singly and double charged BSA by MALDI-TOF/TOF, and the calculated average molecular weight of the singly charged species. The bottom row in bold is average molecular weight obtained from 10 measurements.

| BSA | | 1a@BSA | | 1e@BSA | |
|---|---|---|---|---|---|
| $[BSA+H^+]^{1+}$ | $[BSA+2H^+]^{2+}$ | $[BSA+H^+]^{1+}$ | $[BSA+2H^+]^{2+}$ | $[BSA+H^+]^{1+}$ | $[BSA+2H^+]^{2+}$ |
| 66633.6 | 33240.8 | 66406.1 | 33241.8 | 67233.6 | 33602.4 |
| 66607.1 | 33315.5 | 66491.7 | 33222.2 | 67346.3 | 33818.4 |
| 66606.2 | 33335.7 | 66729.1 | 33265.8 | 67421.8 | 33354.4 |
| 66446.9 | 33250.0 | 66456.2 | 33233.4 | 67242.3 | 33791.9 |
| 66610.0 | 33251.8 | 66700.3 | 33259.4 | 67168.4 | 33621.0 |
| 66609.4 | 33346.9 | 66379.5 | 33214.0 | 67469.4 | 33844.7 |
| 66610.5 | 33251.0 | 66515.0 | 33239.5 | 67296.7 | 33580.2 |
| 66182.5 | 33140.3 | 66692.4 | 33140.8 | 67022.3 | 33341.9 |
| 66610.0 | 33249.8 | 66550.9 | 33249.7 | 67184.0 | 33603.9 |
| 66607.7 | 33357.2 | 66466.7 | 33357.3 | 67008.4 | 33409.0 |
| **66551.4** | **66554.8** | **66537.8** | **66482.8** | **67238.3** | **67191.5** |

## 6. Antiproliferative activity

### MTT tests

The experiments were carried out on human carcinoma cell lines H460 (lung) and MCF-7 (breast), which were cultured as monolayers and maintained in Dulbecco's modified Eagle medium (DMEM) supplemented with 10% fetal bovine serum (FBS), 2 mM L-glutamine, 100 U/mL penicillin and 100 µg/mL streptomycin in a humidified atmosphere with 5% $CO_2$ at 37 °C. The cells were inoculated in parallel on two 96-well microtiter plates on day 0, at $3\times10^4$ cells/mL (H460), $4.5\times10^4$ cells/mL (MCF-7). Test agents were added in ten-fold dilutions ($10^{-8}$ to $10^{-4}$ M) on the next day and incubated for further 72 h. Working dilutions were freshly prepared on the day of testing. One of the plates was left in the dark, while the other was irradiated in a Luzchem reactor (6 lamps with the maximum at 350 nm for 5 min) at 4, 24, and 48 hours after the addition of the tested compounds. After 72 h of incubation, the cell growth rate was evaluated by performing the MTT assay. The absorbances were measured on a microplate reader at 570 nm. The percentage of growth (PG) of the cell lines was calculated according to the previously described.[5] Each test was performed in at least two individual experiments. Control experiments were performed with a psoralene derivative (trioxsalen), which is known to induce photoactivable cross-linking[6,7] [88,12] and Naphth (Eq 1 in the manuscript).

Table S9. Antiproliferative activity $IC_{50}$ (µM) of **1**.[a]

| Compound | H 460 dark | H 460 350 nm (3×5 min) | MCF-7 dark | MCF-7 350 nm (3×5 min) |
|---|---|---|---|---|
| **1a** | 35 ± 3 | 30 ± 1 | 12.6 ±0.7 | 15.2 ± 0.8 |
| **1b** | 47 ± 7 | 21 ± 3 | 15.1 ± 0.3 | 14.1 ± 0.6 |
| **1c** | 44 ± 7 | 13 ± 2 | 13.68 ± 0.06 | 3.1 ± 0.1 |
| **1d** | 55 ± 5 | 2.4 ± 0.5 | 18 ± 3 | 1.54 ± 0.06 |
| **1e** | > 100 | 1.8 ± 0.2 | 17 ± 9 | 1.45 ± 0.09 |
| Naphth | > 100 | 44 ± 9 | 25 ± 5 | 11.65 ± 0.08 |
| Trioxsalen[b] | > 100 | < 0.01 | 16 ± 6 | < 0.01 |

[a] $IC_{50}$, concentration that causes 50% inhibition of the cell growth. [b]Trioxsalen IUPAC name: 2,5,9- trimethyl-7*H*-furo[3,2-*g*]chromen-7-one

Detailed analysis of results in Table S9 showed decreasing cytotoxicity under dark conditions (expressed by $IC_{50}$ values) proportional to the linker length between the two chromophores, the **1e** bearing the longest linker being non-toxic for the H-460 cell line even at the highest tested concentration. This could be correlated to the significantly higher aggregation tendency of the derivative with the longest linker (**1e**) in respect to the shortest linker (**1a**), as discussed in DNA-fluorimetric titrations; whereby such aggregation could also control interactions with biorelevant intracellular targets, and consequently, the bioactivity of compounds. However, enhancement of the antiproliferative effect upon UV irradiation (Table S9, 350 nm) shows the opposite trend; the strongest effects were observed for the compounds with longer linkers (**1d**, **1e**). This result suggests that UV irradiation of aggregated **1e** inside the cell leads to the production of a higher amount of cytotoxic agent (possibly QMs as well as singlet oxygen / reactive oxygen species-ROS) in comparison to the smallest **1a** (showing an order of magnitude weaker effect). Furthermore, it was demonstrated that **1e** leads to the photoalkylation of the protein, whereas **1a** does not, which may be put in connection with a higher ability of **1e** to form QMs in the protein complex than **1a** and lead to the cytotoxic effect.

Compounds **1a-e** exhibit higher cytotoxicity than Naphth, indicating that the naphthalimide moiety is important for the activity. Although we do not have clear evidence which of reactive species formed in photochemical reactions lead to the observed photoinduced antiproliferative effect, it is plausible that singlet $O_2$ and subsequent ROS formed by the quenching of the naphthalimide triplet play an important role, as well as QMs and reactive radicals formed in the photoinduced processes.

## 6. NMR spectra

$^{1}$H NMR ($CDCl_3$, 500 MHz) of **2a** 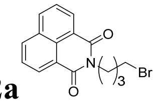


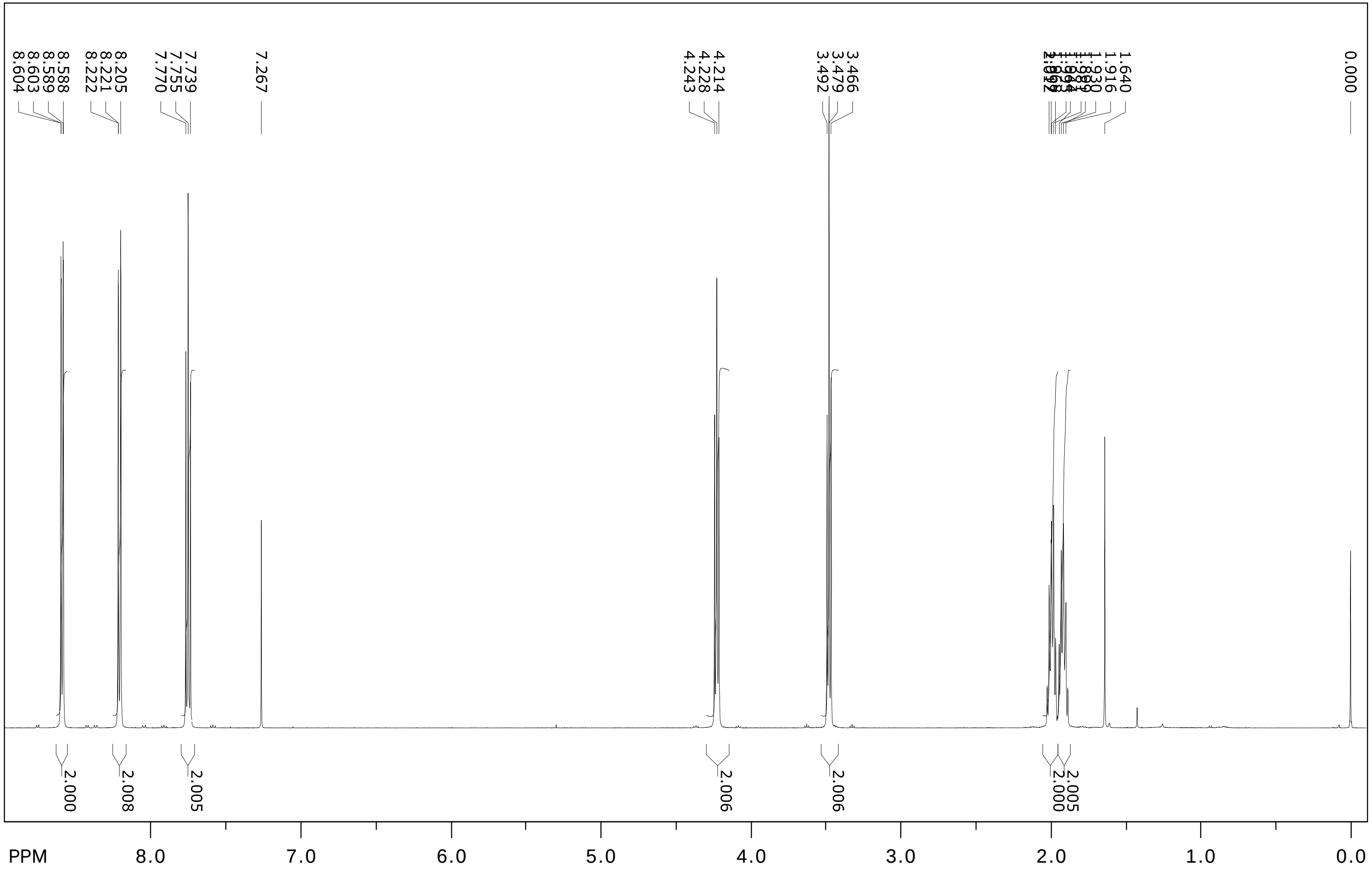

$^{13}$C NMR ($CDCl_3$, 125 MHz) of **2a** 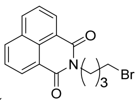



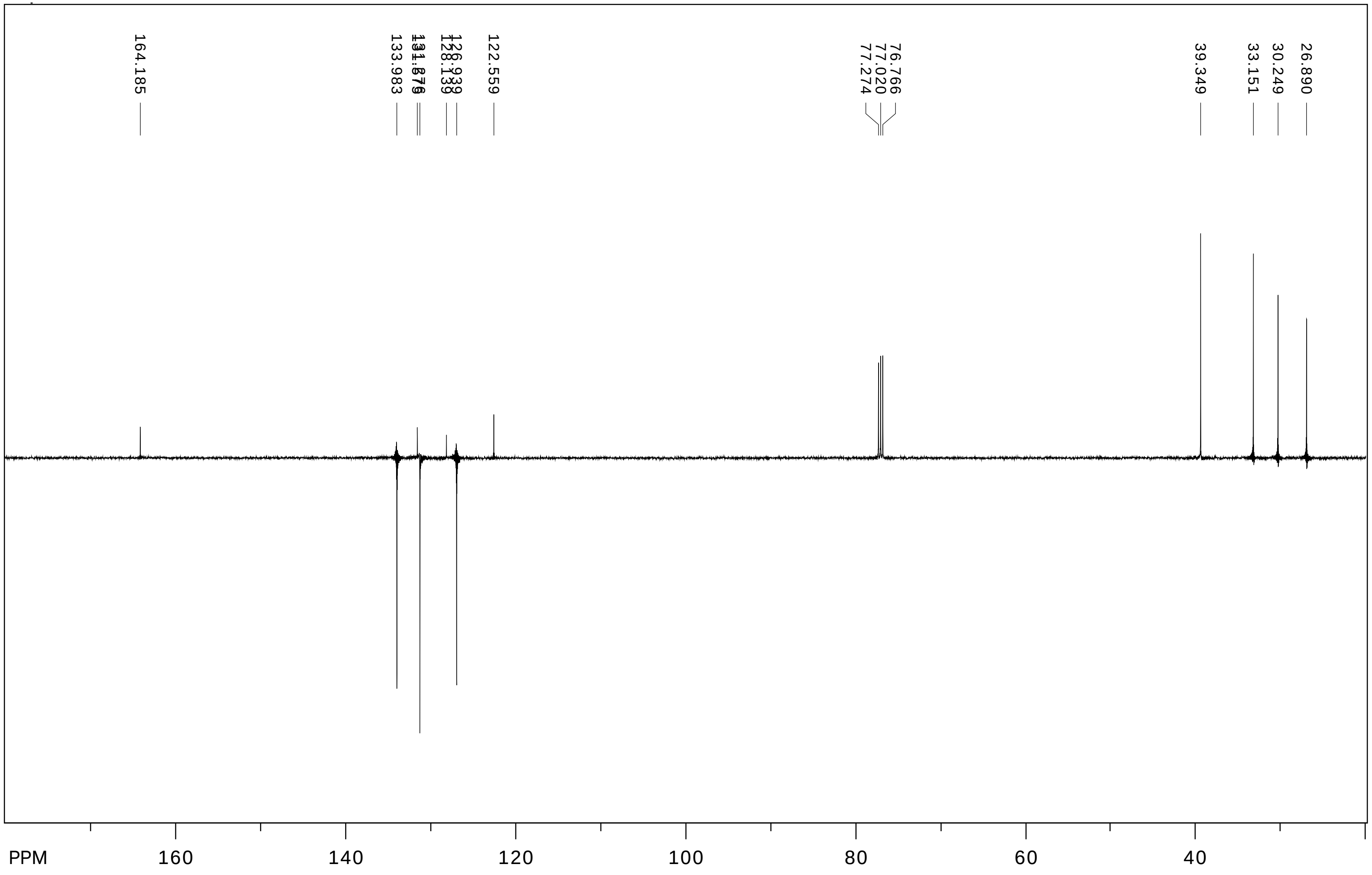

$^{1}$H NMR ($CDCl_3$, 400 MHz) of **2b** 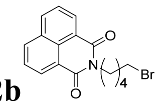


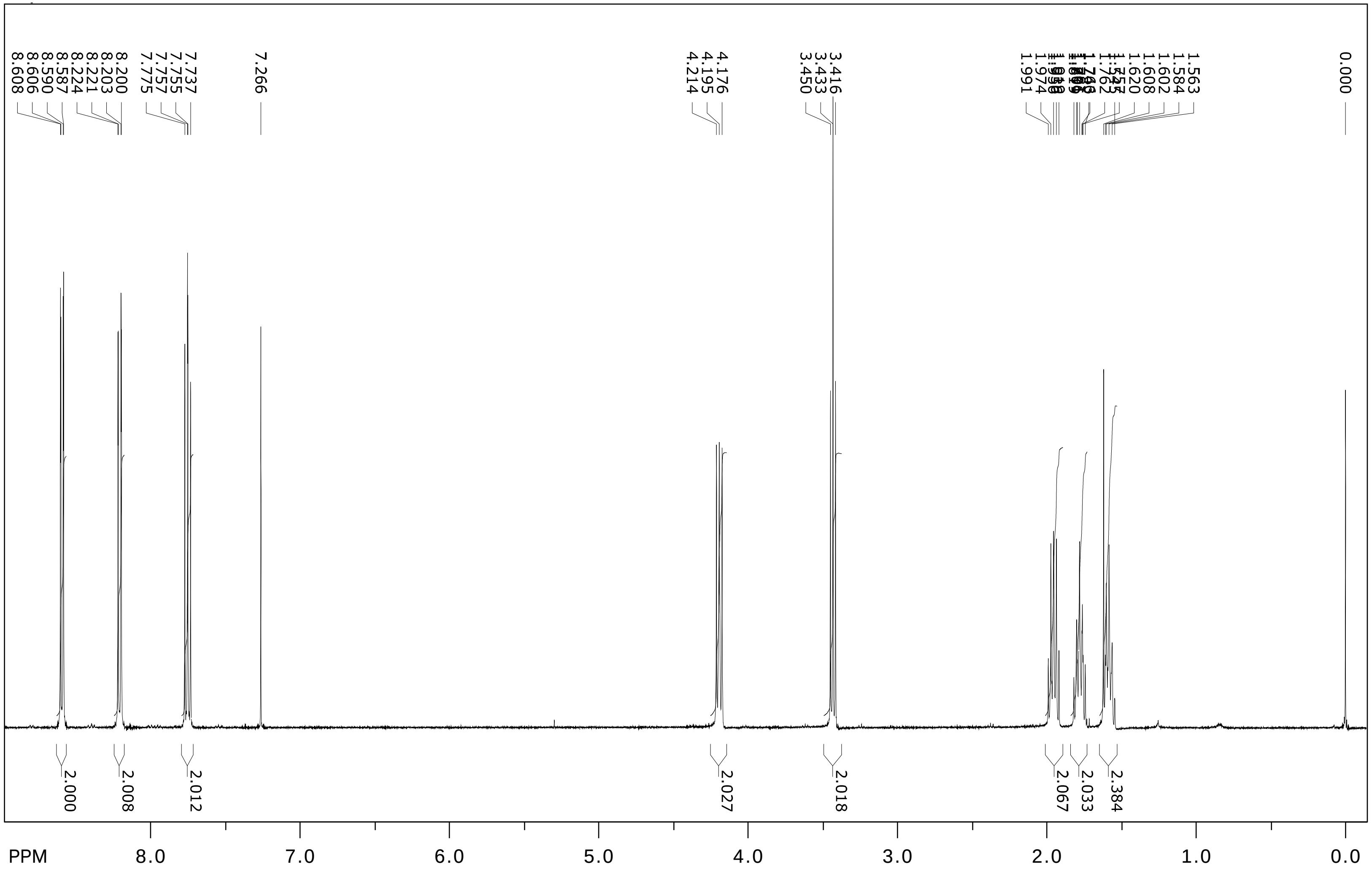

$^{13}$C NMR ($CDCl_3$, 100 MHz) of **2b**

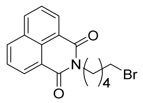


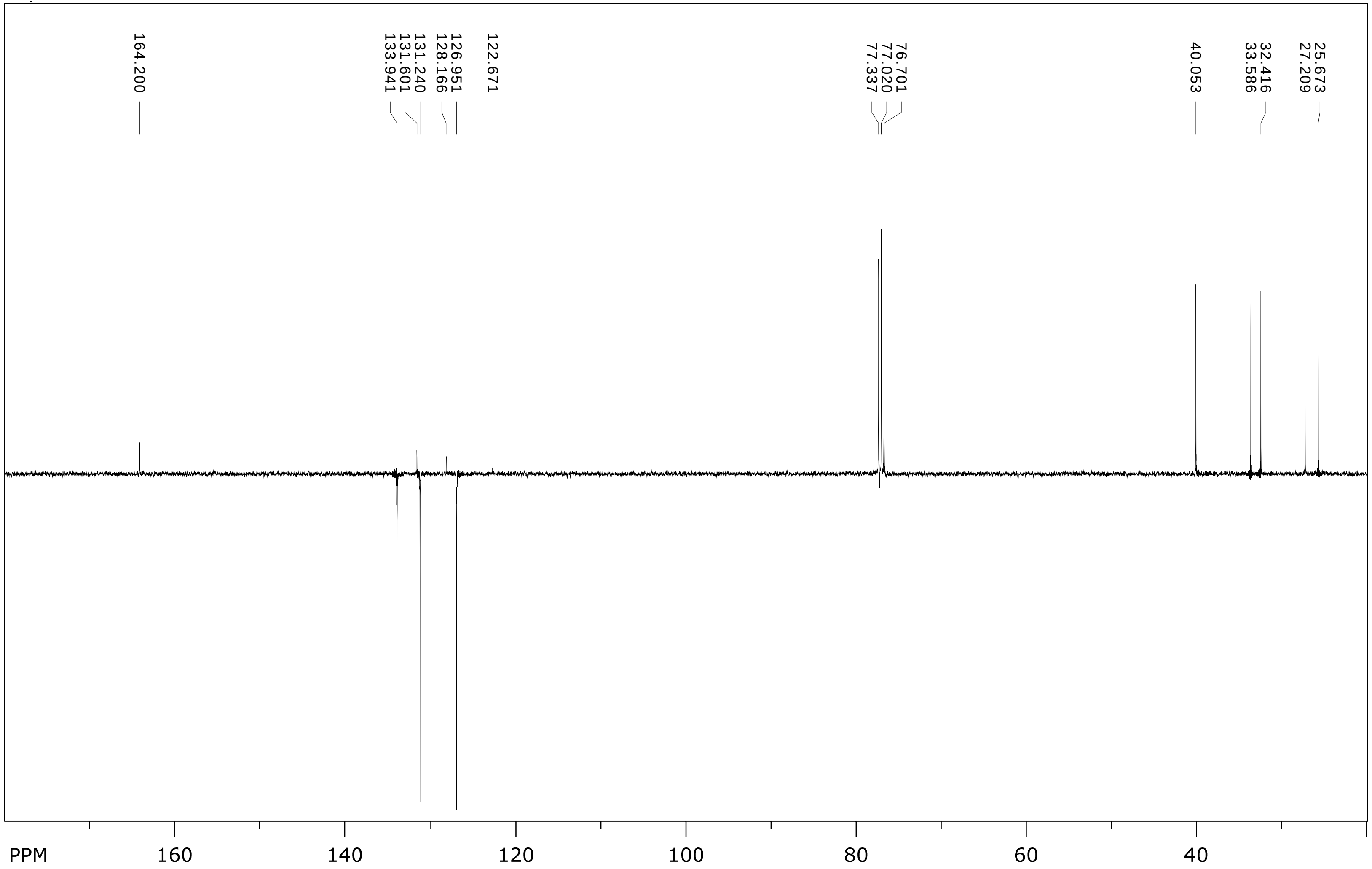

$^{1}$H NMR ($CDCl_3$, 600 MHz) of **2c** 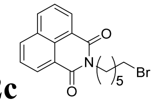


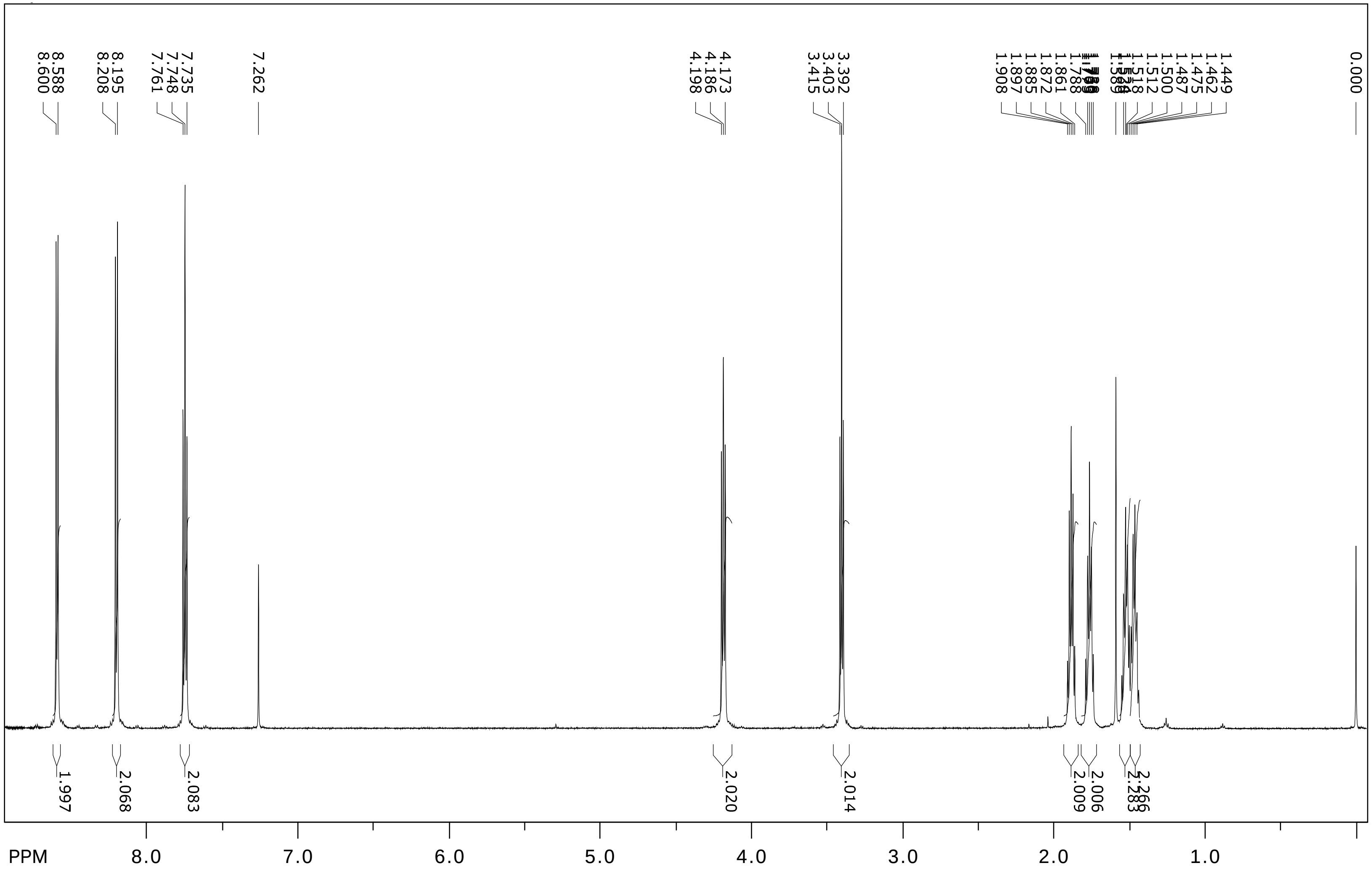

$^{13}C$ NMR ($CDCl_3$, 100 MHz) of **2c** 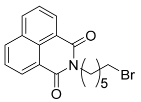



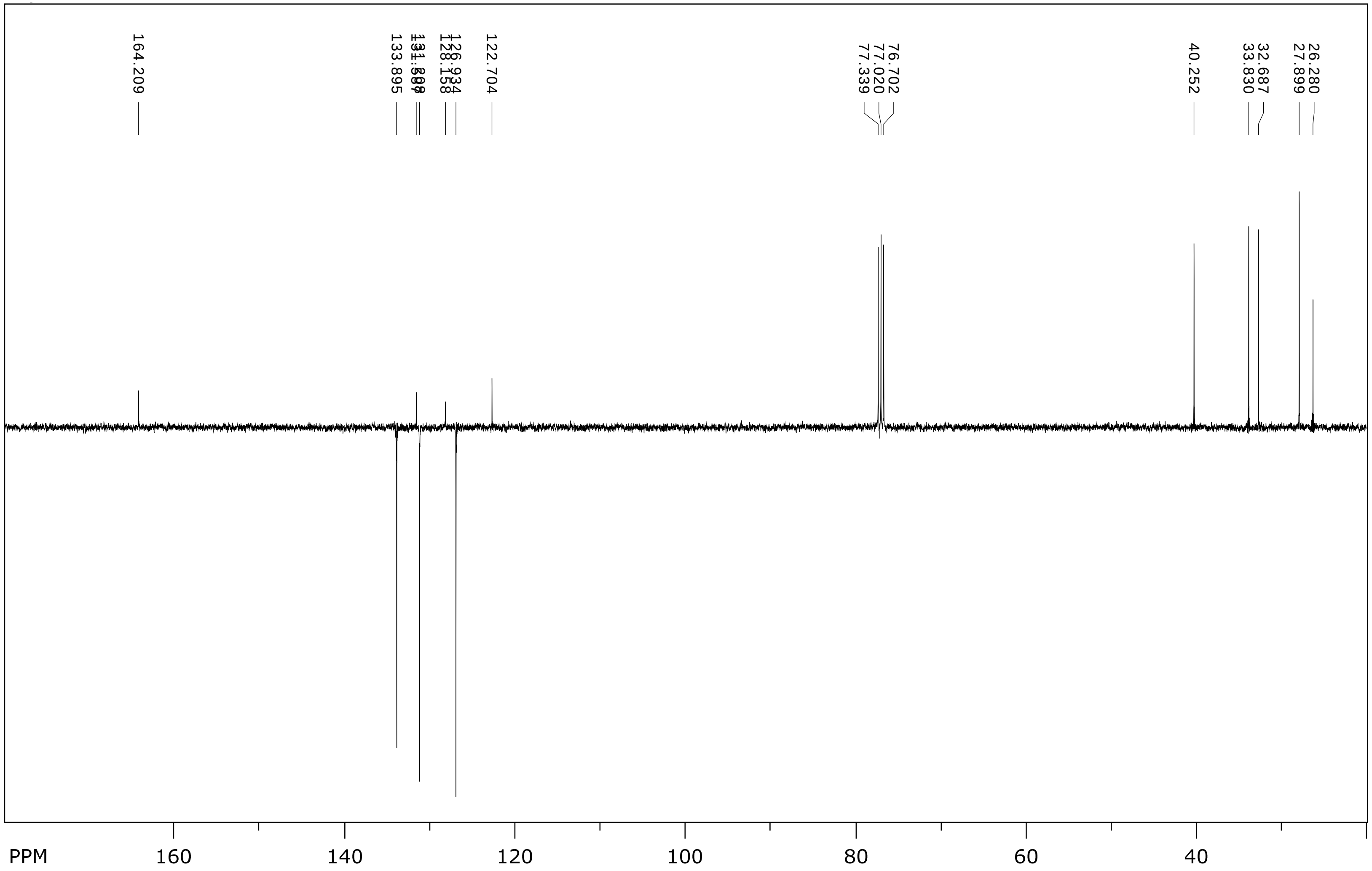

$^{1}$H NMR ($CDCl_3$, 400 MHz) of **2d** 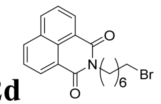


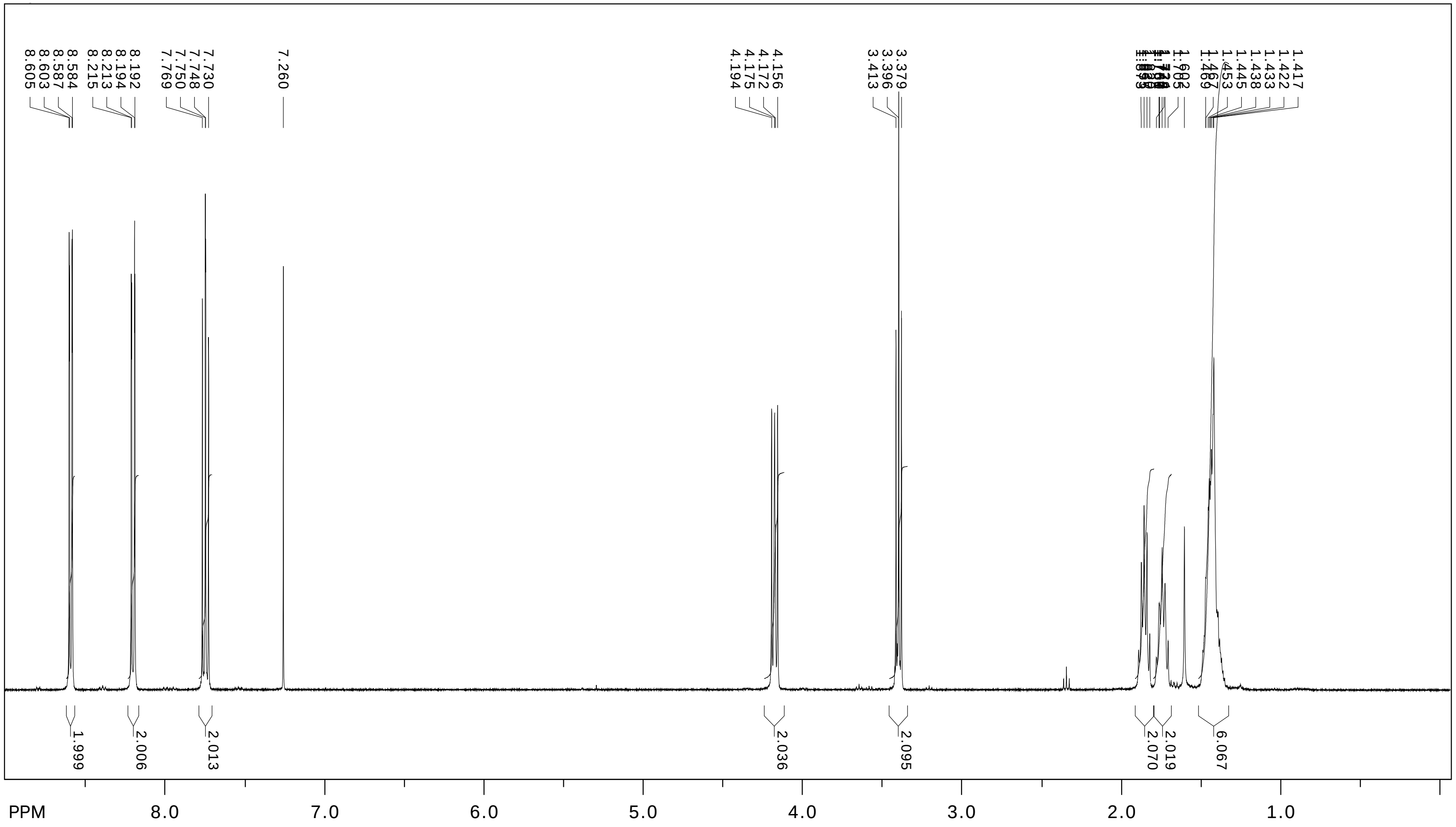

$^{13}C$ NMR ($CDCl_3$, 100 MHz) of **2d**

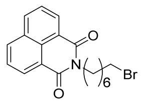


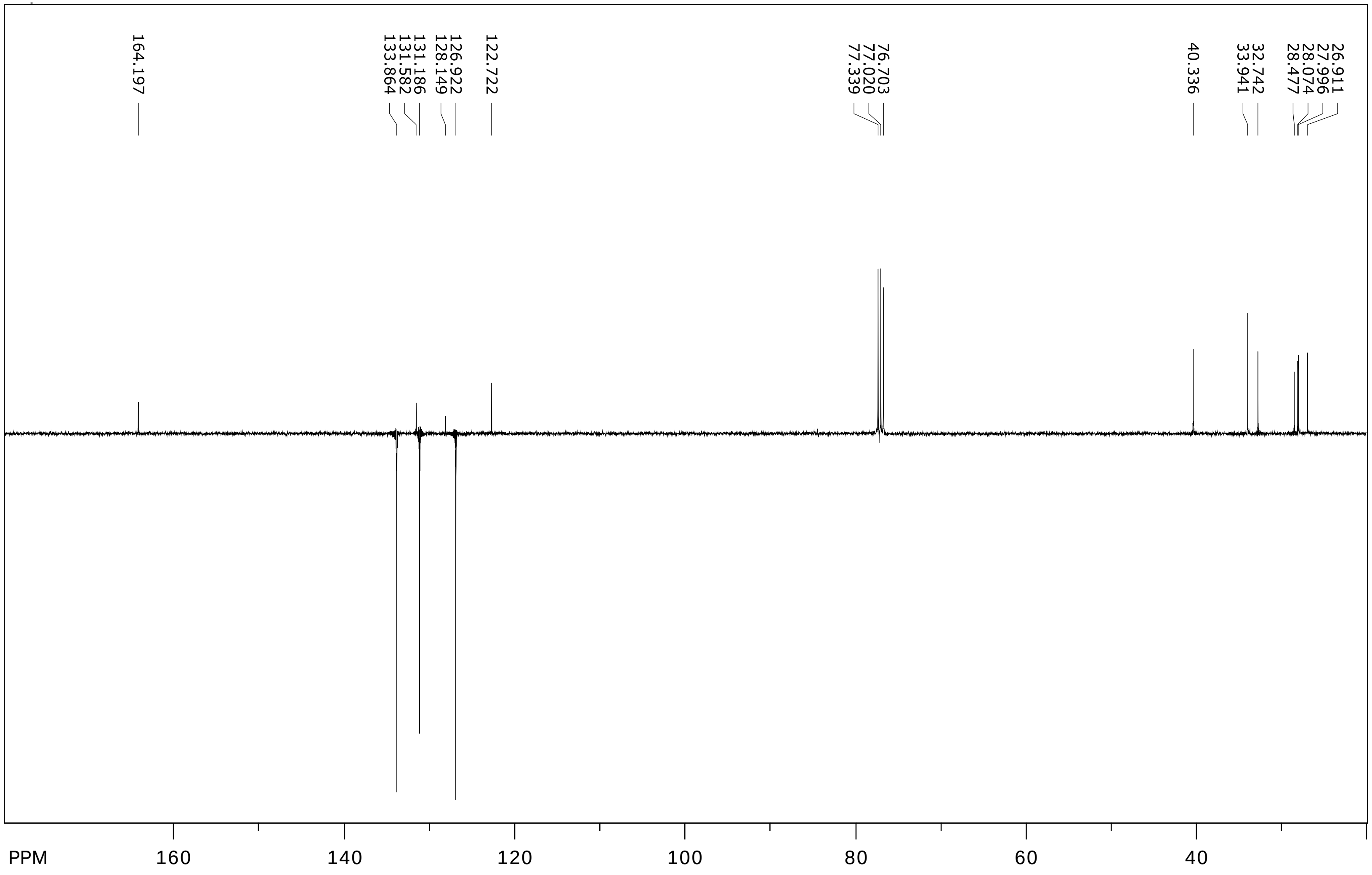

$^{1}$H NMR (CDCl3, 400 MHz) of **2e**

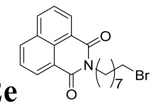

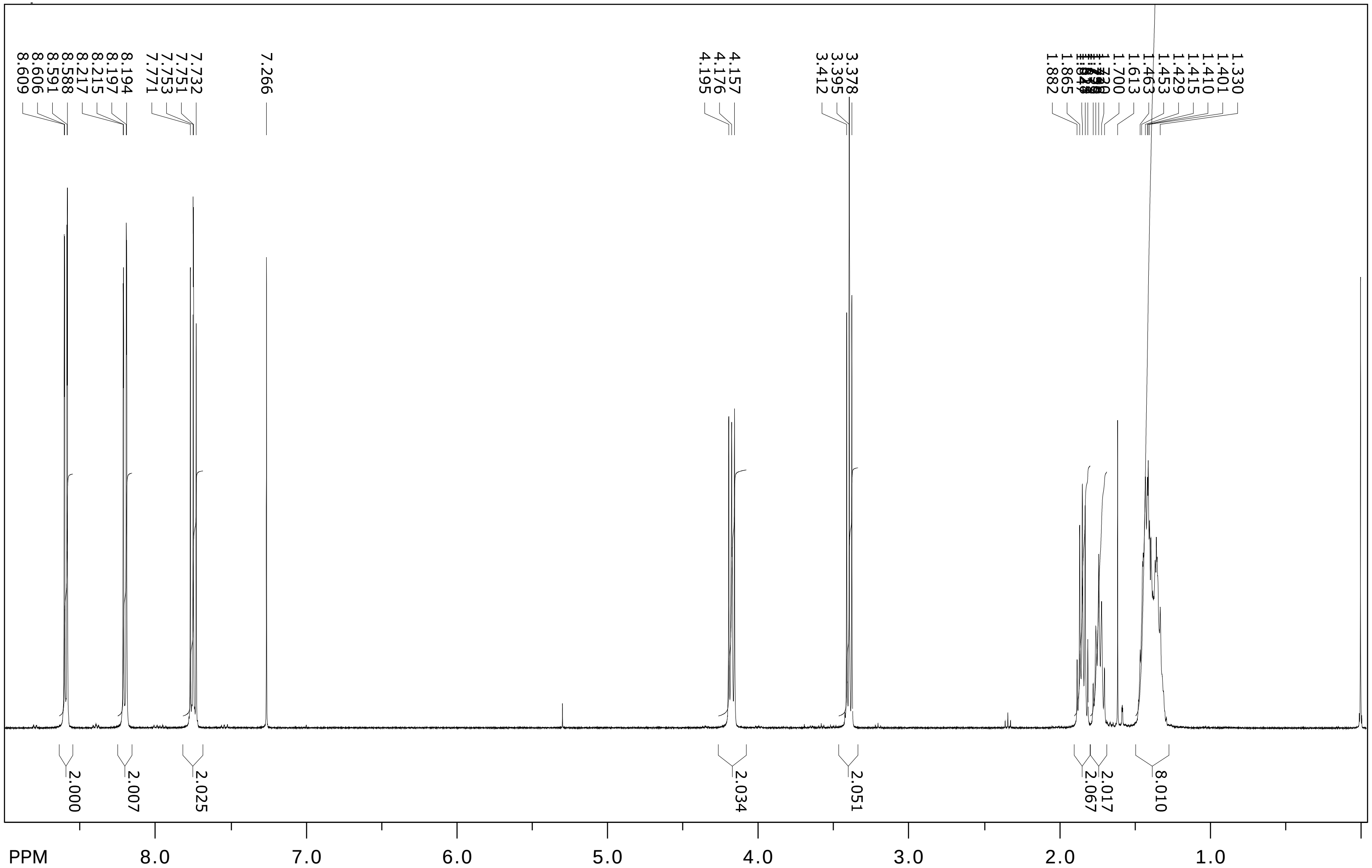

$^{13}C$ NMR ($CDCl_3$, 100 MHz) of **2e** 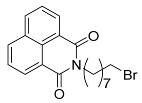


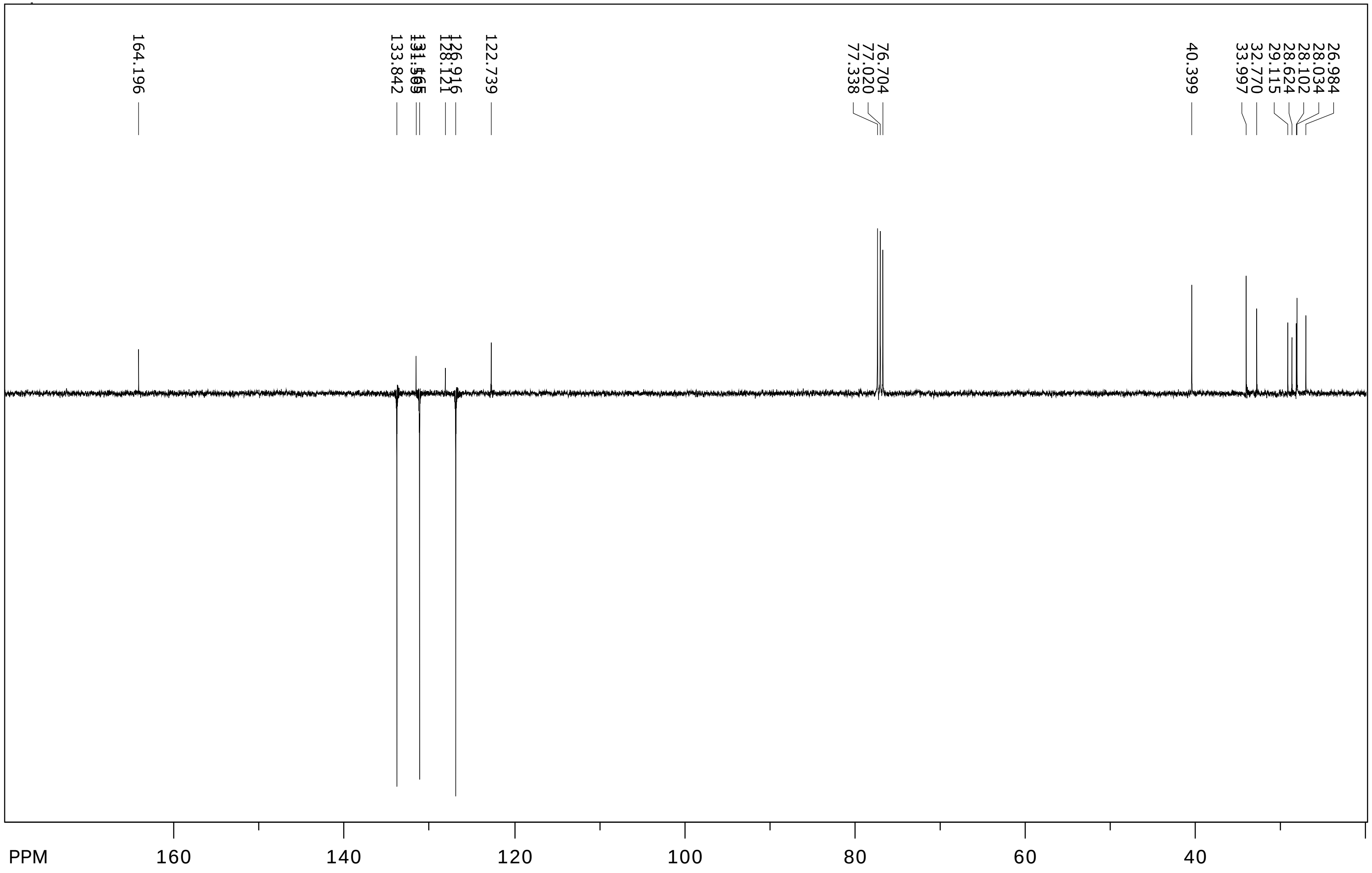

$^{1}$H NMR ($CDCl_3$, 400 MHz) of **3a** 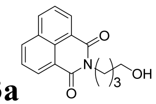

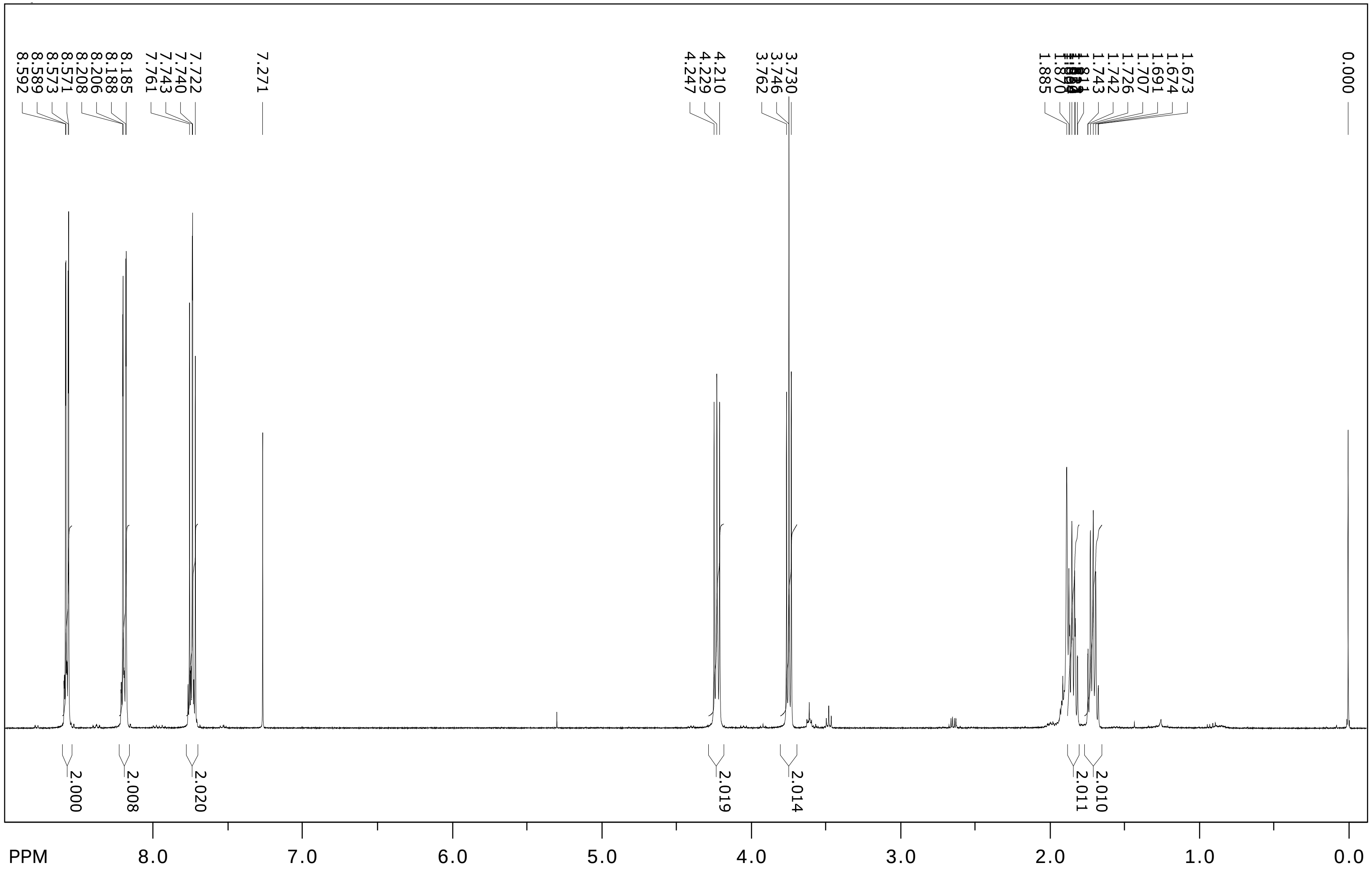

$^{13}$C NMR ($CDCl_3$, 100 MHz) of **3a** 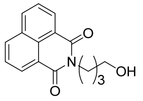

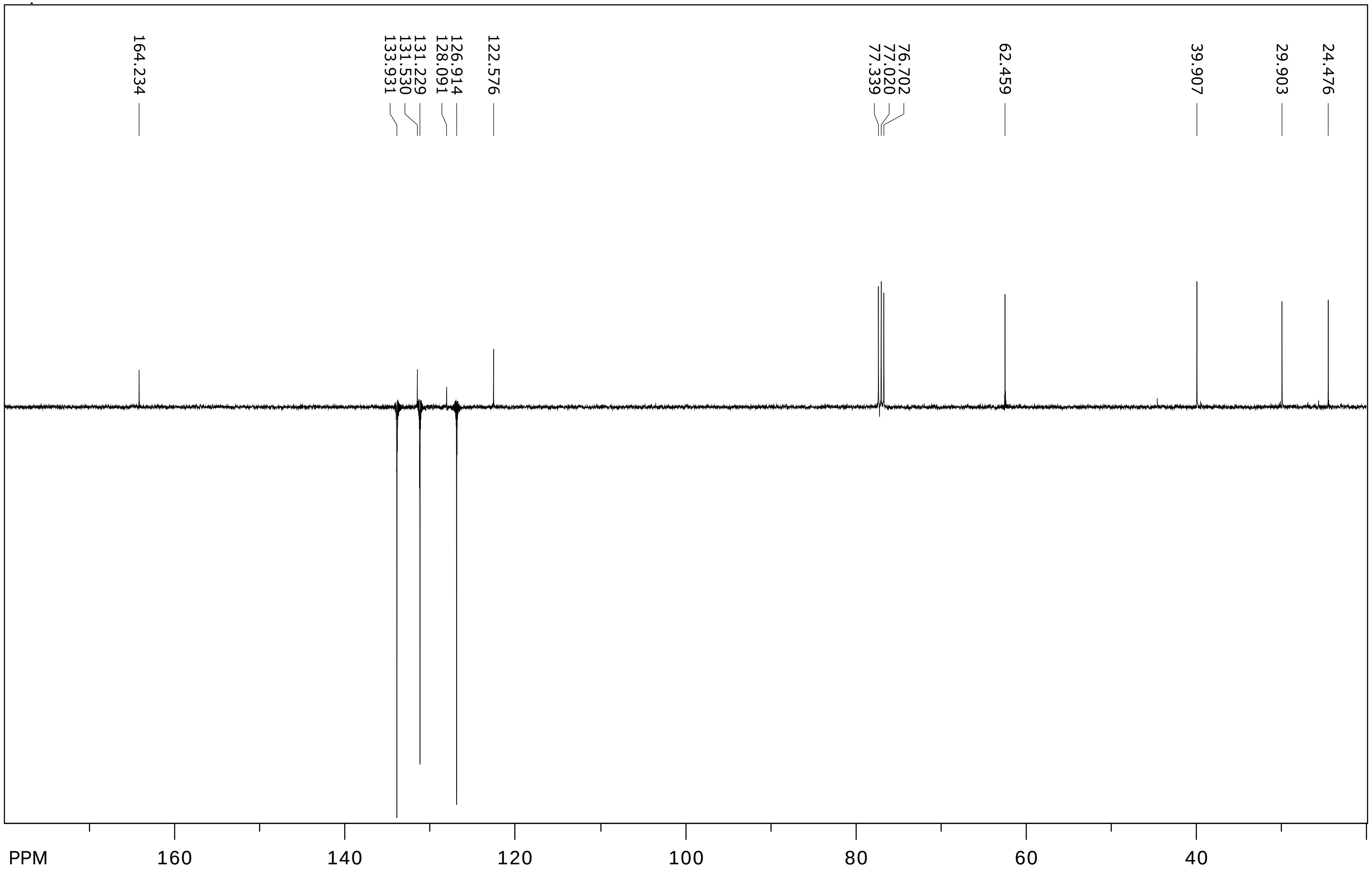

$^{1}$H NMR (CDCl3, 300 MHz) of **3b**

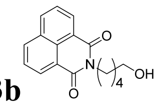

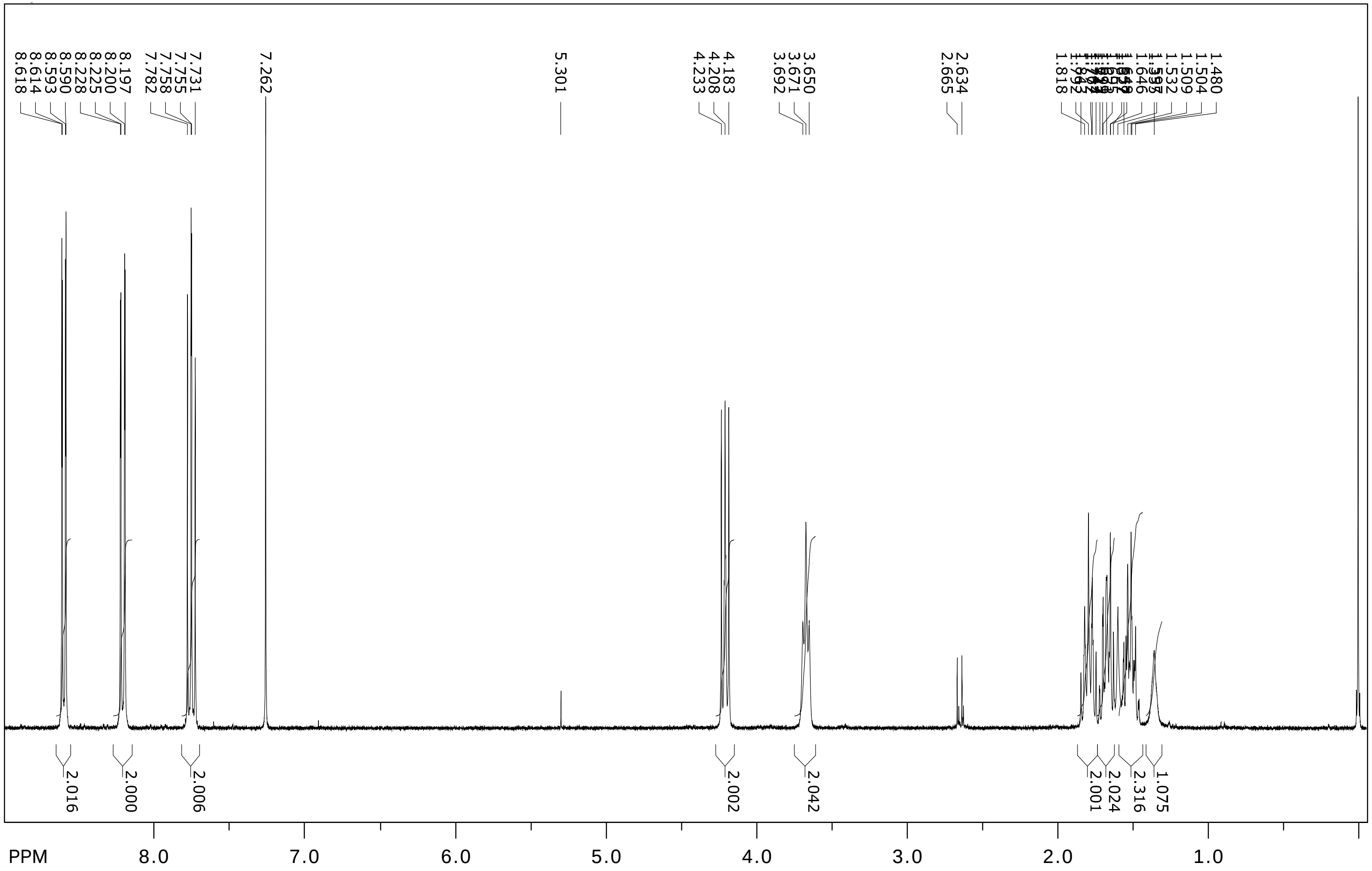

$^{13}$C NMR ($CDCl_3$, 100 MHz) of **3b** 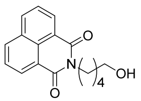


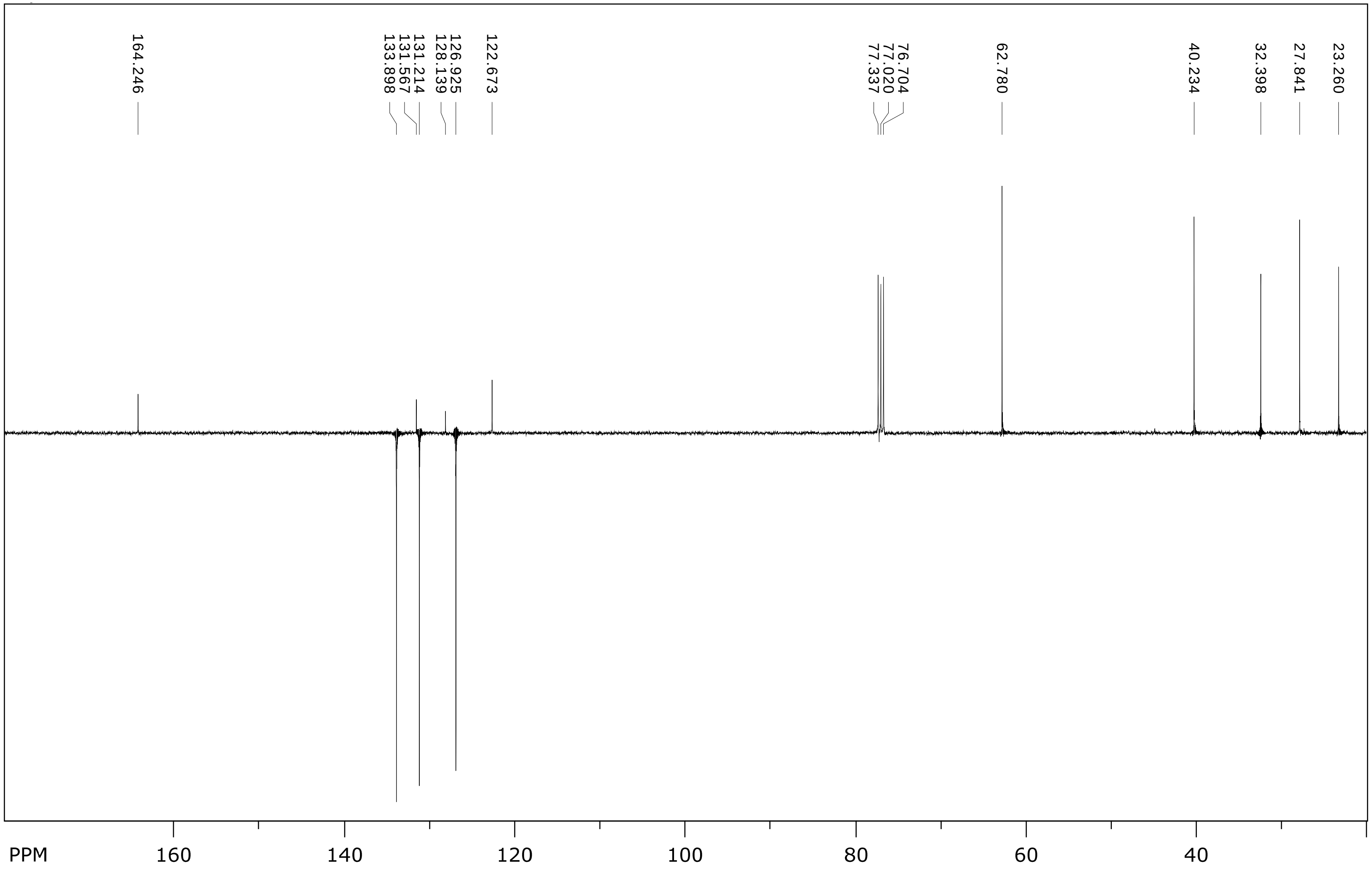

$^{1}$H NMR (CDCl3, 400 MHz) of **3c**

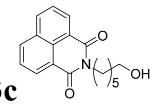

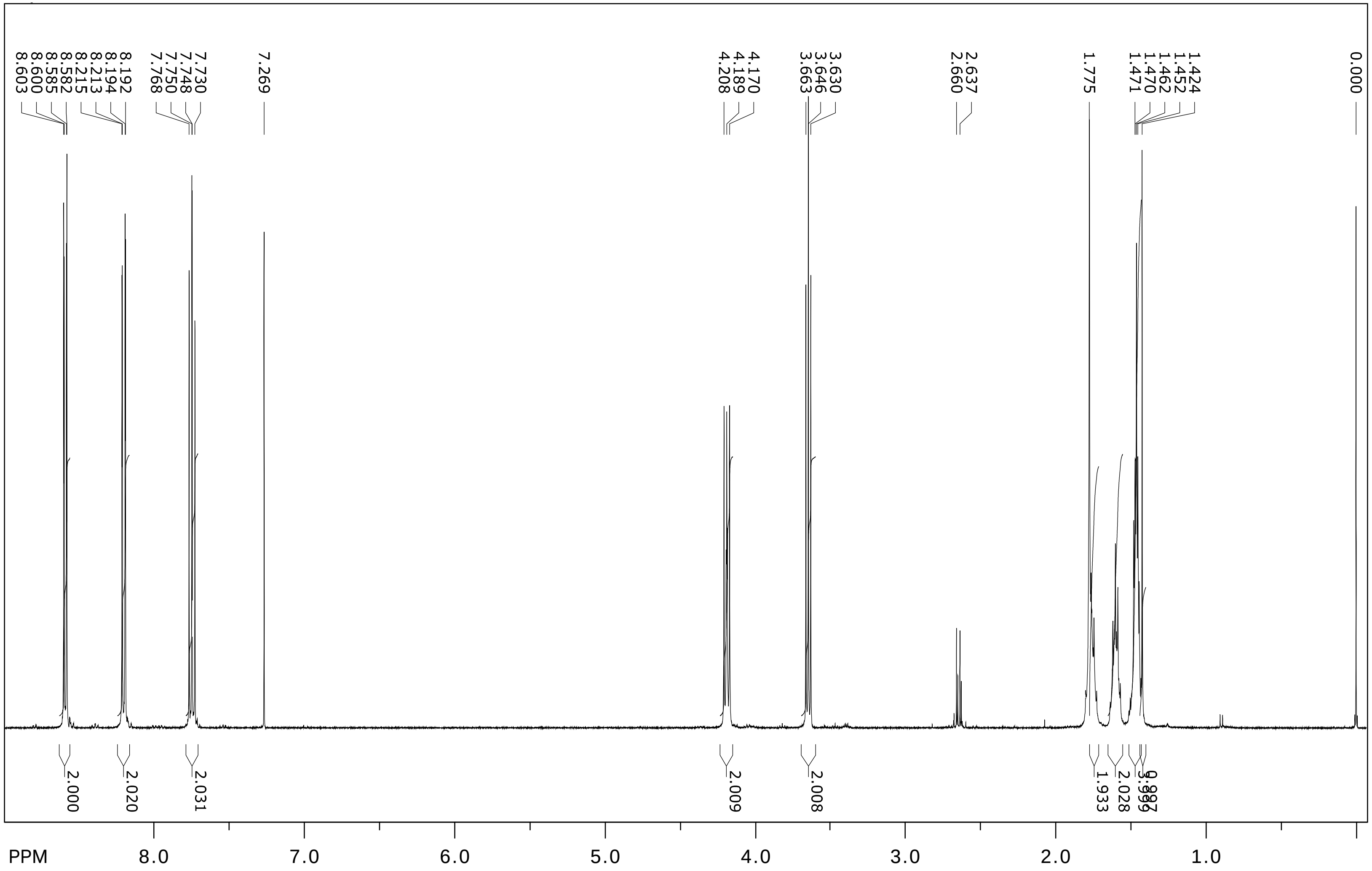

$^{13}$C NMR ($CDCl_3$, 100 MHz) of **3c** 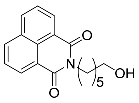

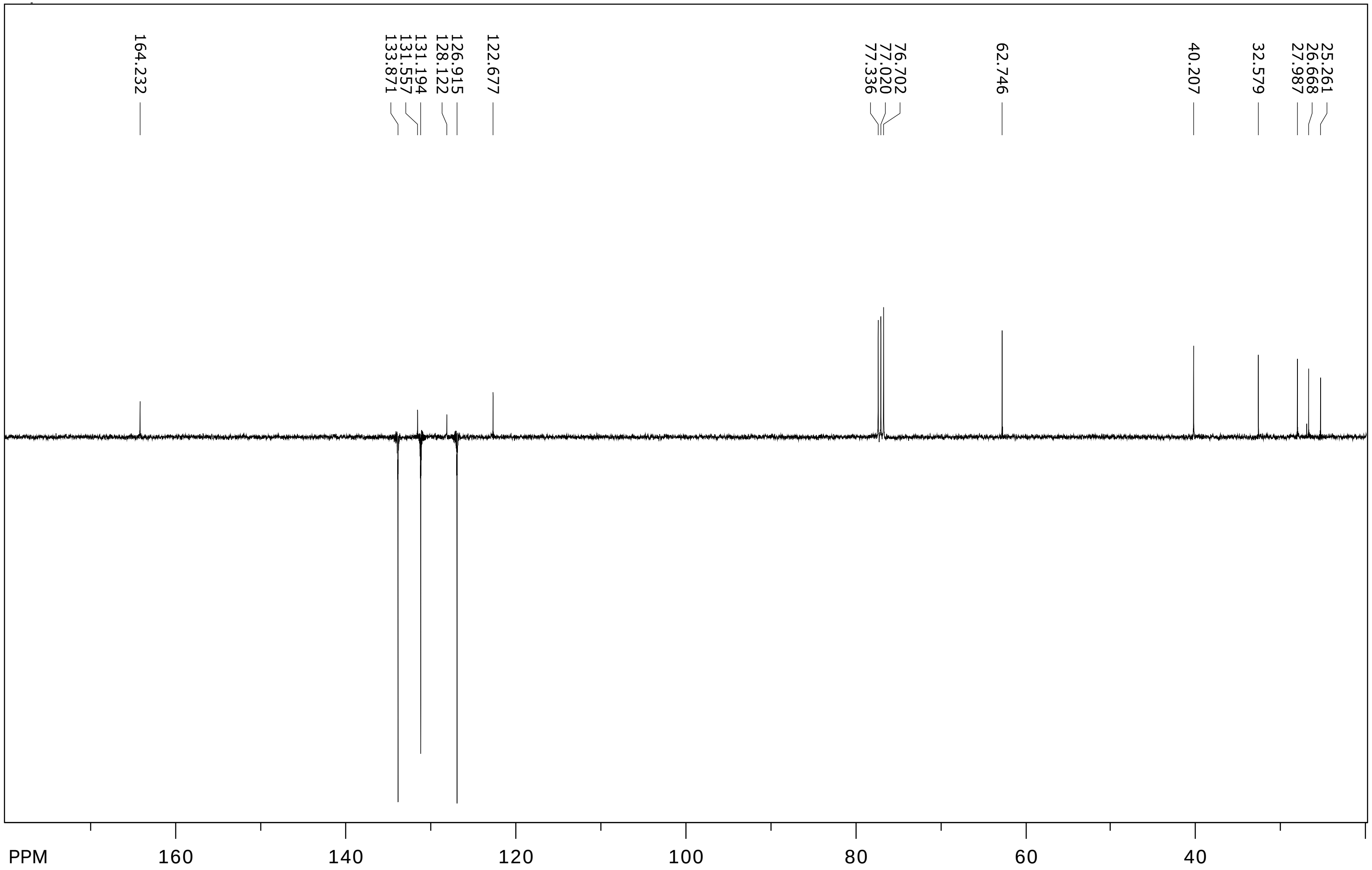

$^{1}$H NMR ($CDCl_3$, 400 MHz) of **3d** 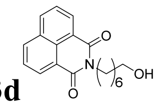

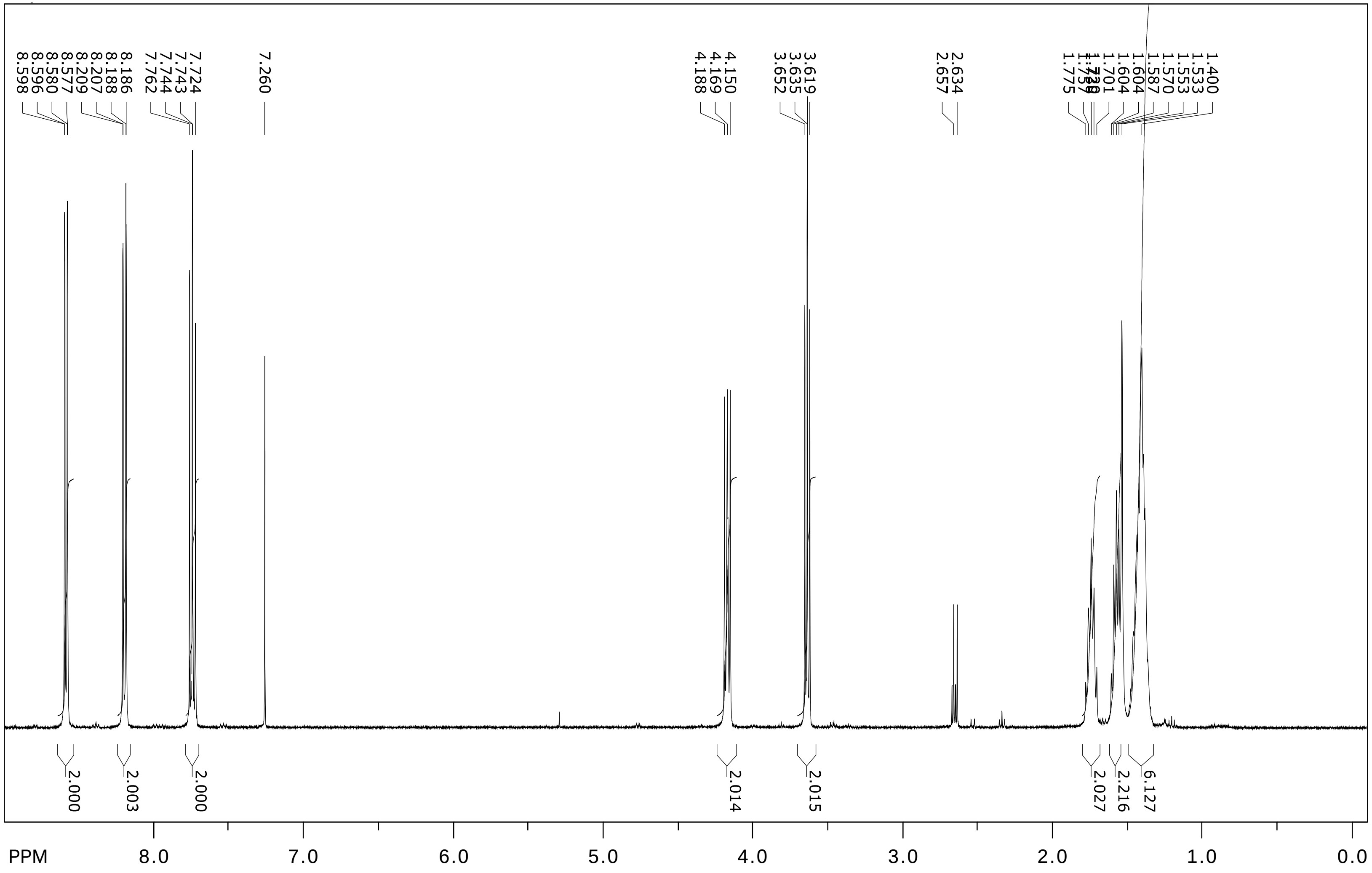

$^{13}$C NMR ($CDCl_3$, 100 MHz) of **3d** 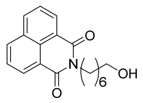


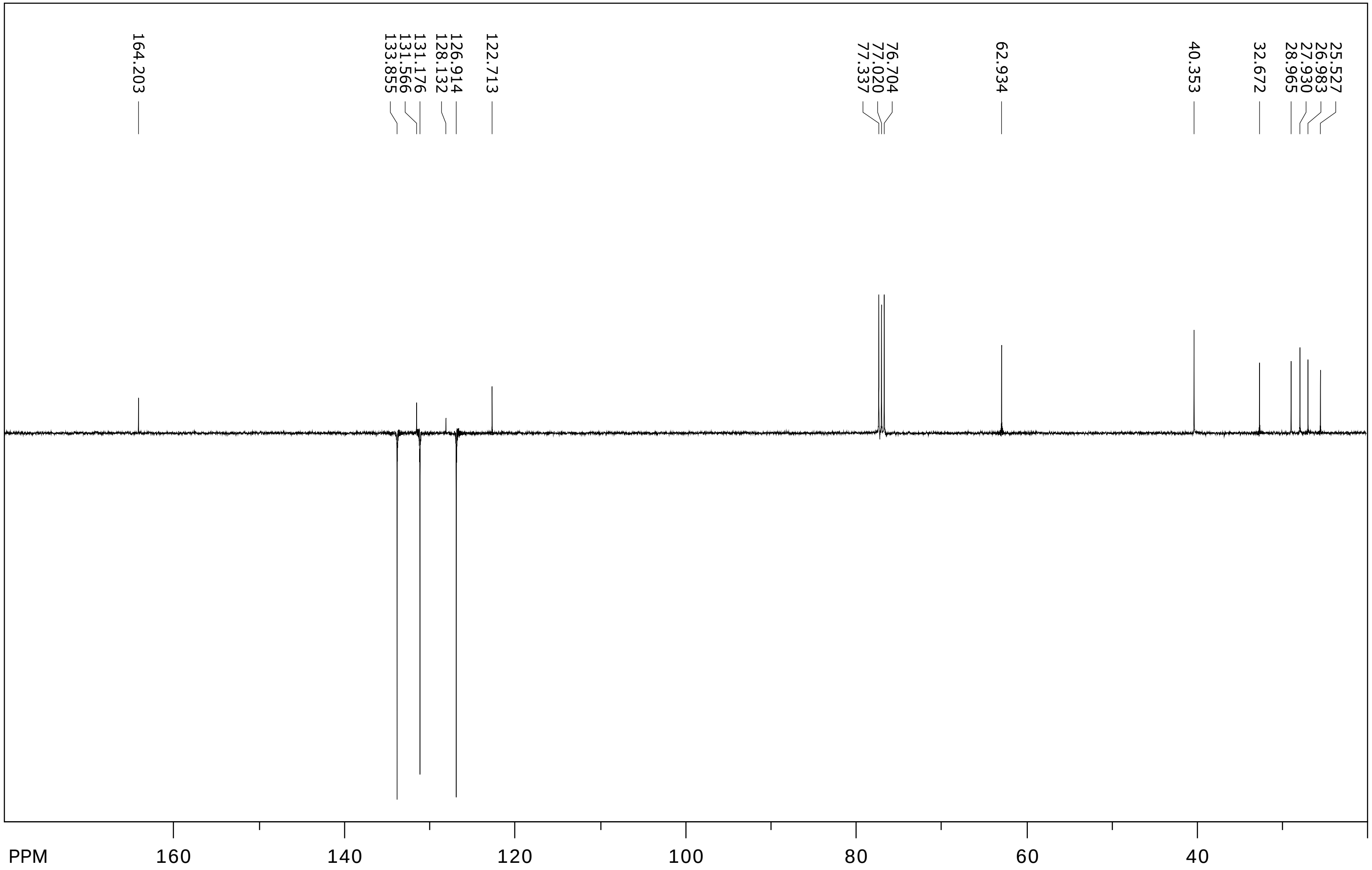

$^{1}$H NMR ($CDCl_3$, 400 MHz) of **3e**

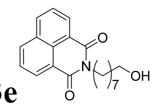

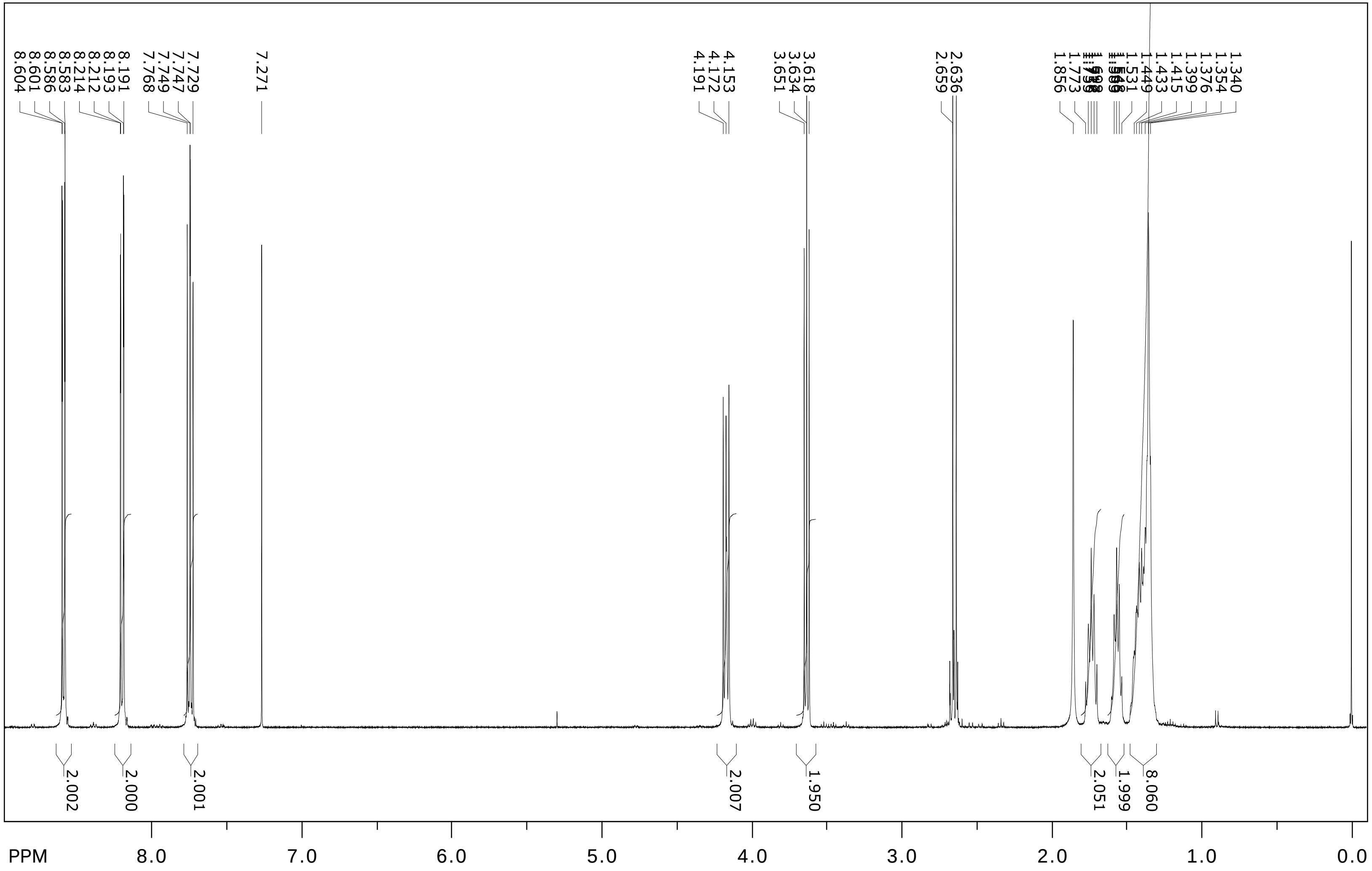

$^{13}C$ NMR ($CDCl_3$, 100 MHz) of **3e**

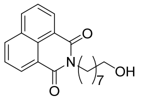

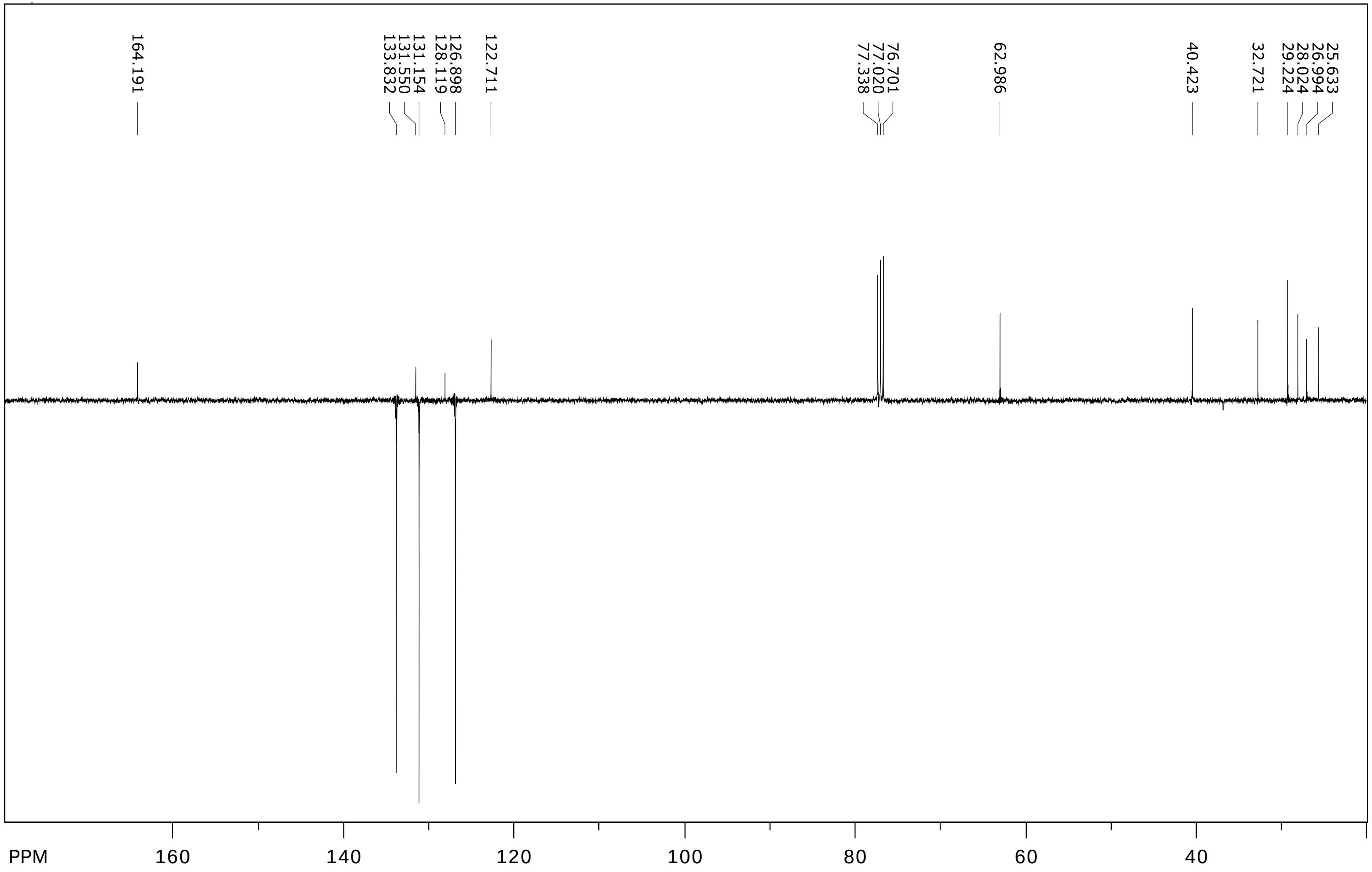

$^{1}$H NMR (CDCl3, 400 MHz) of **4a**

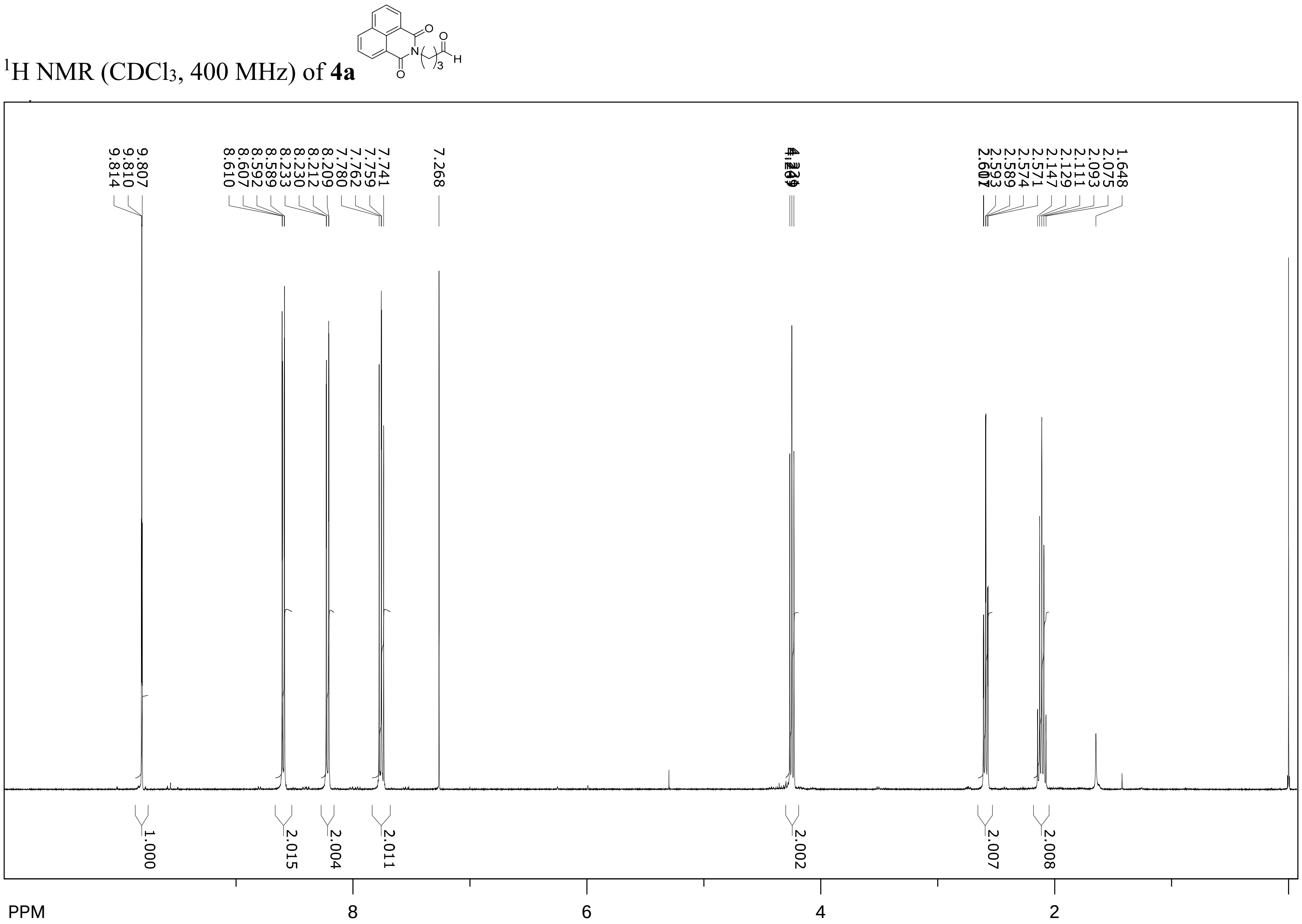

$^{13}C$ NMR ($CDCl_3$, 100 MHz) of **4a**

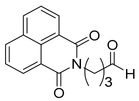

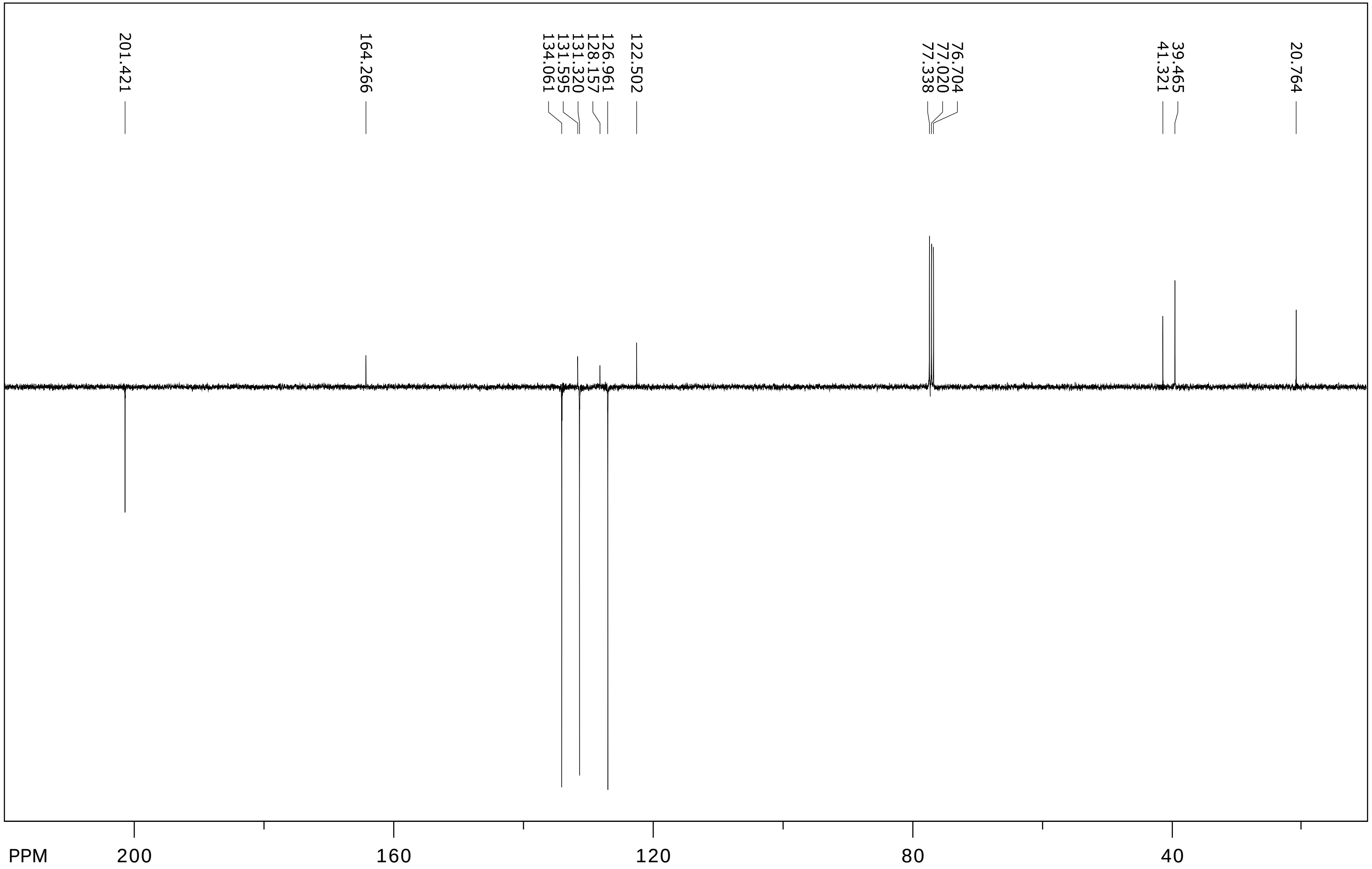

$^{1}$H NMR ($CDCl_3$, 300 MHz) of **4b** 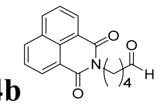

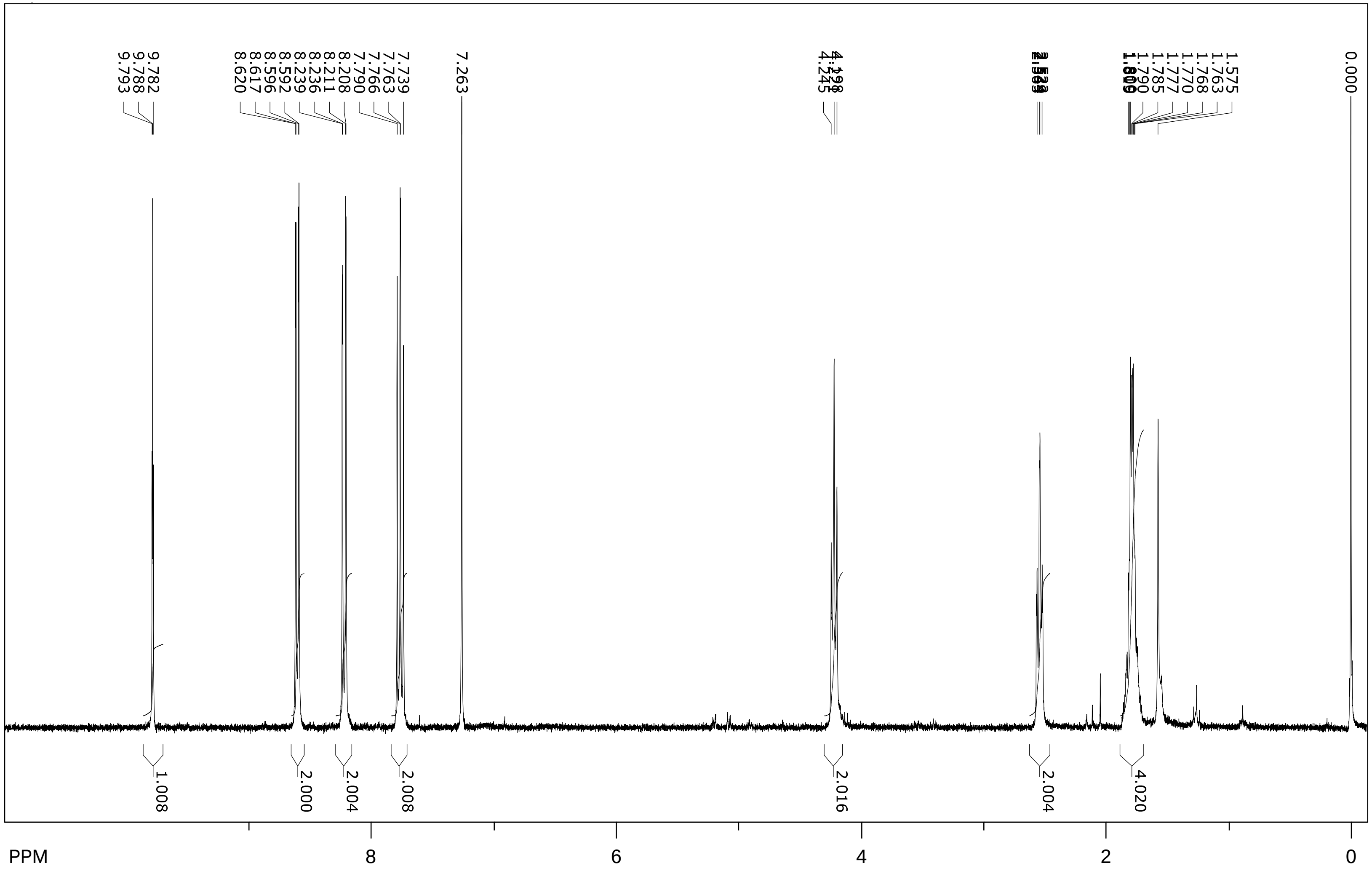

$^{13}$C NMR ($CDCl_3$, 100 MHz) of **4b** 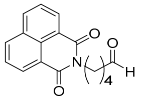

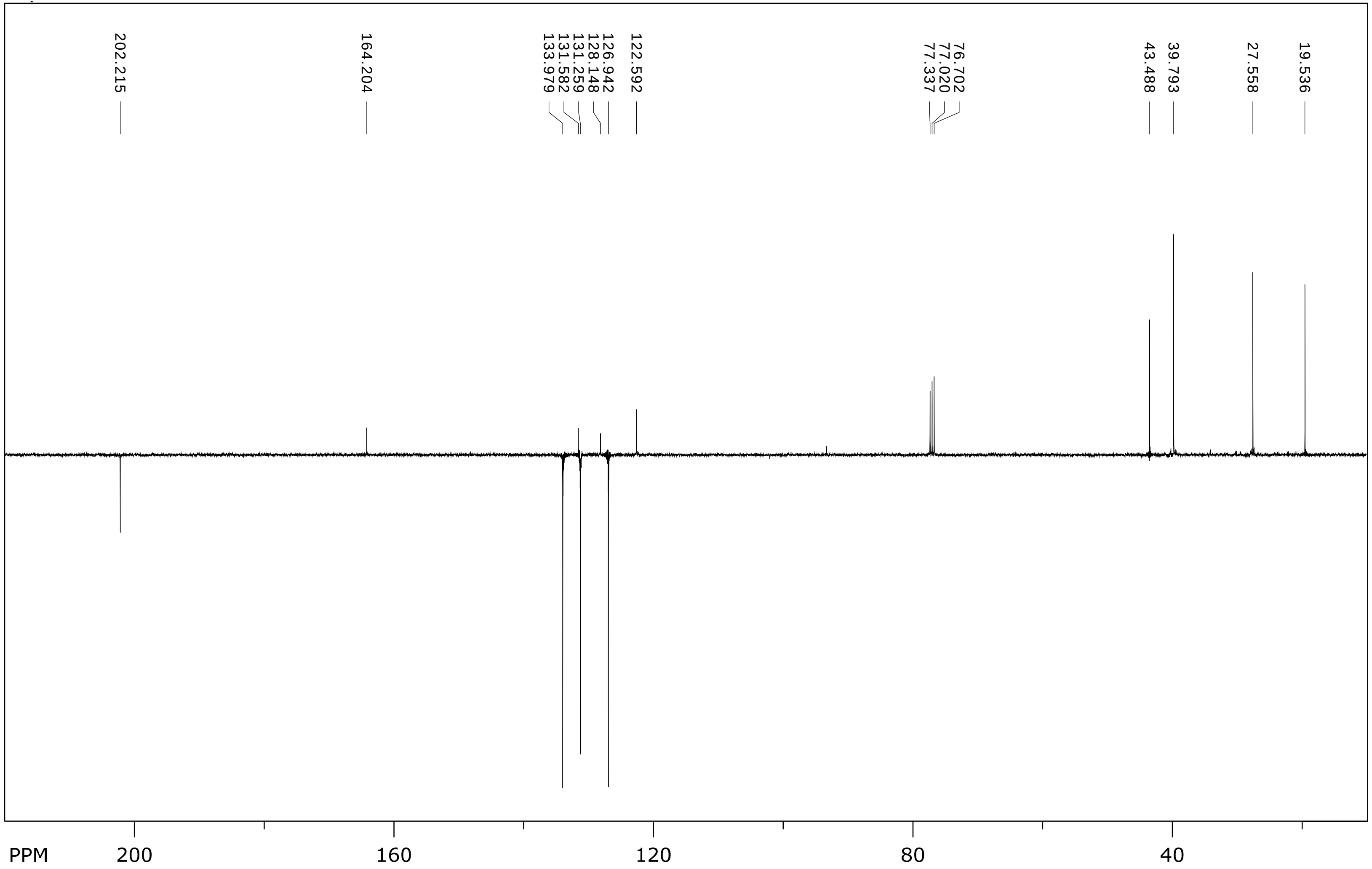

$^{1}$H NMR ($CDCl_3$, 400 MHz) of **4c** 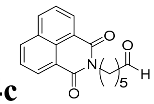

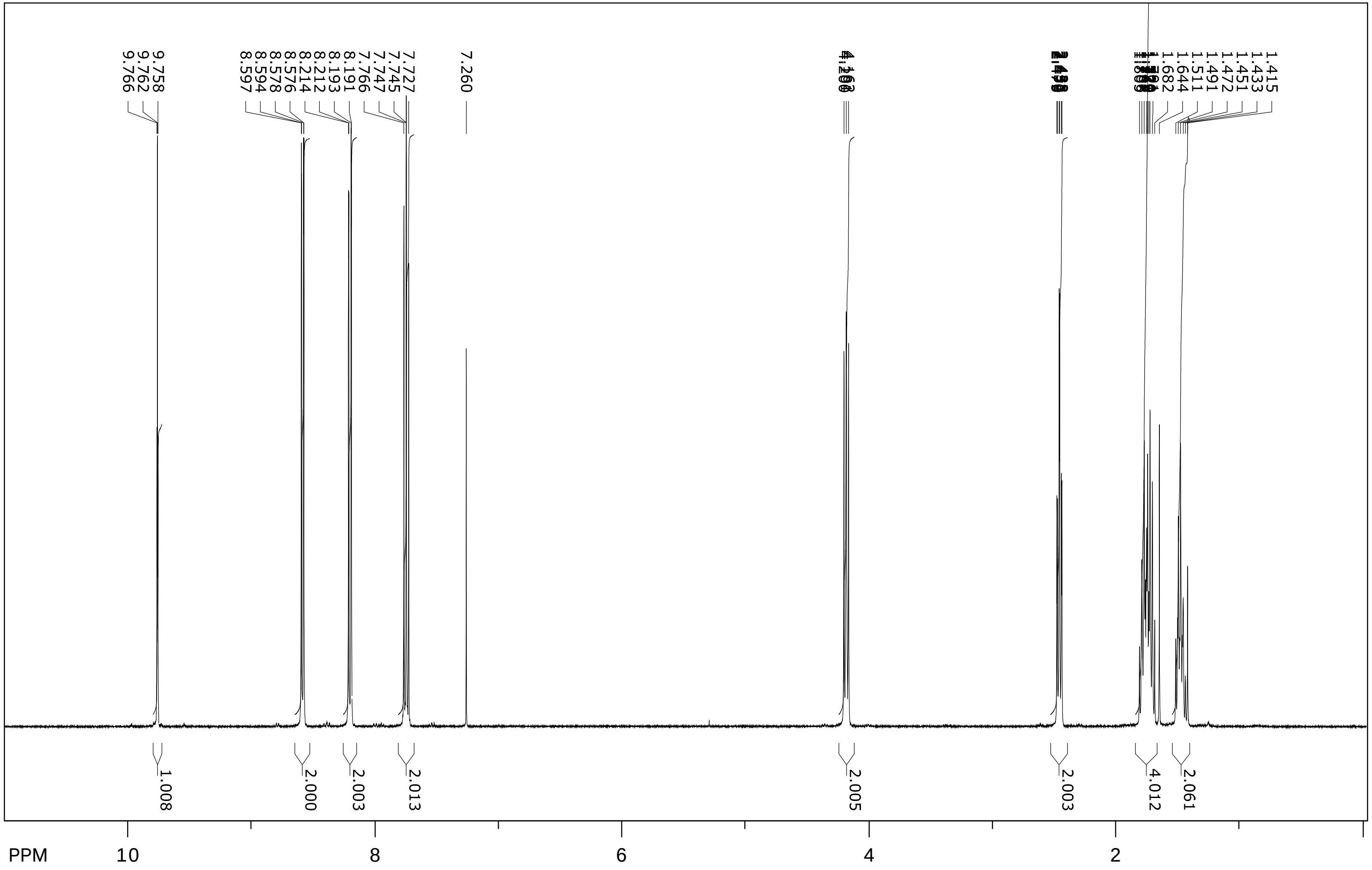

$^{13}$C NMR ($CDCl_3$, 100 MHz) of **4c**

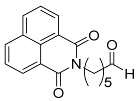

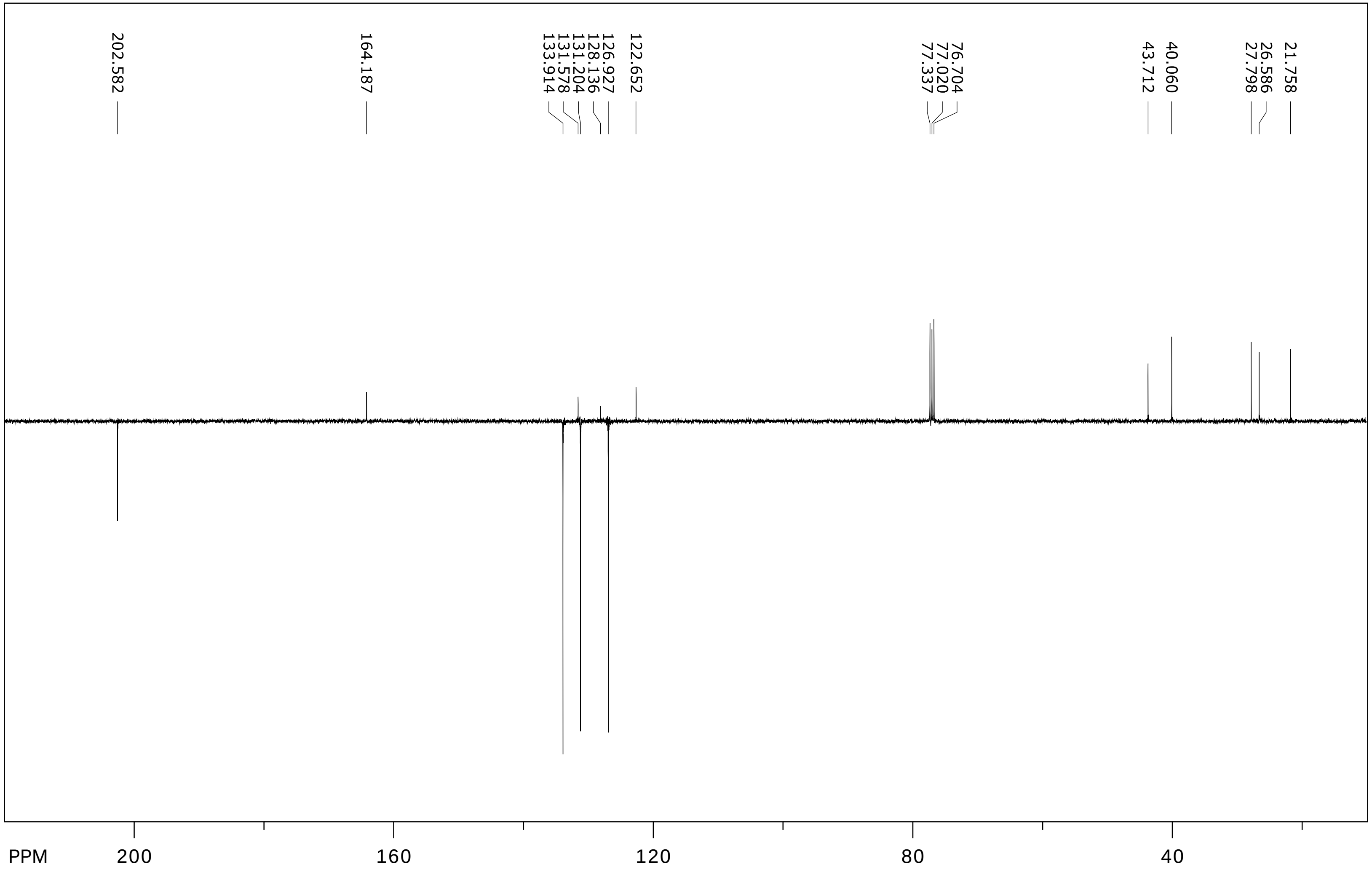

$^{1}$H NMR (CDCl3, 400 MHz) of **4d** 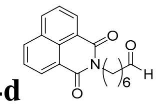

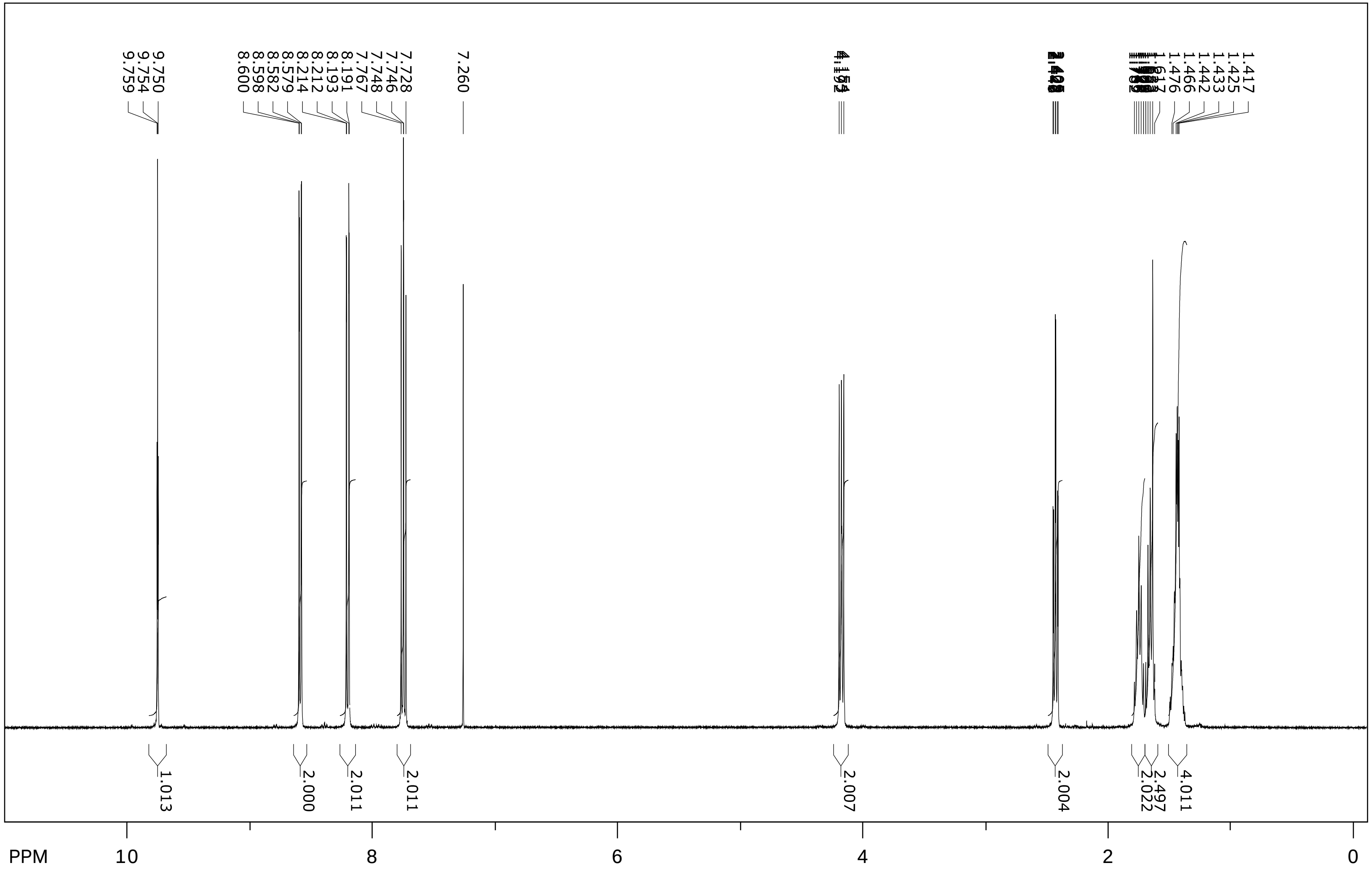

$^{13}C$ NMR ($CDCl_3$, 100 MHz) of **4d**

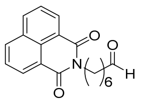

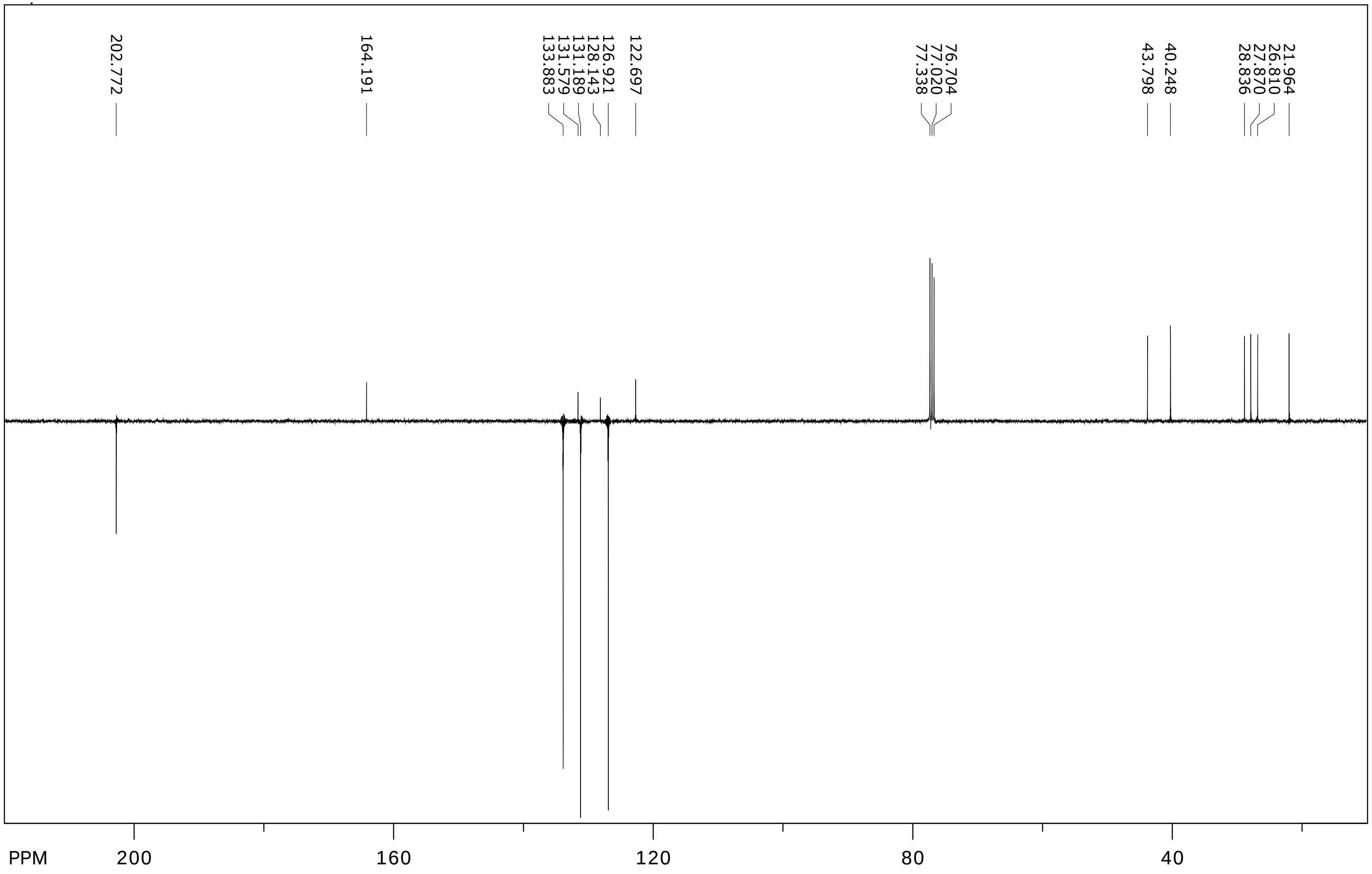

$^{1}$H NMR ($CDCl_3$, 400 MHz) of **4e** 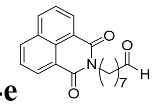


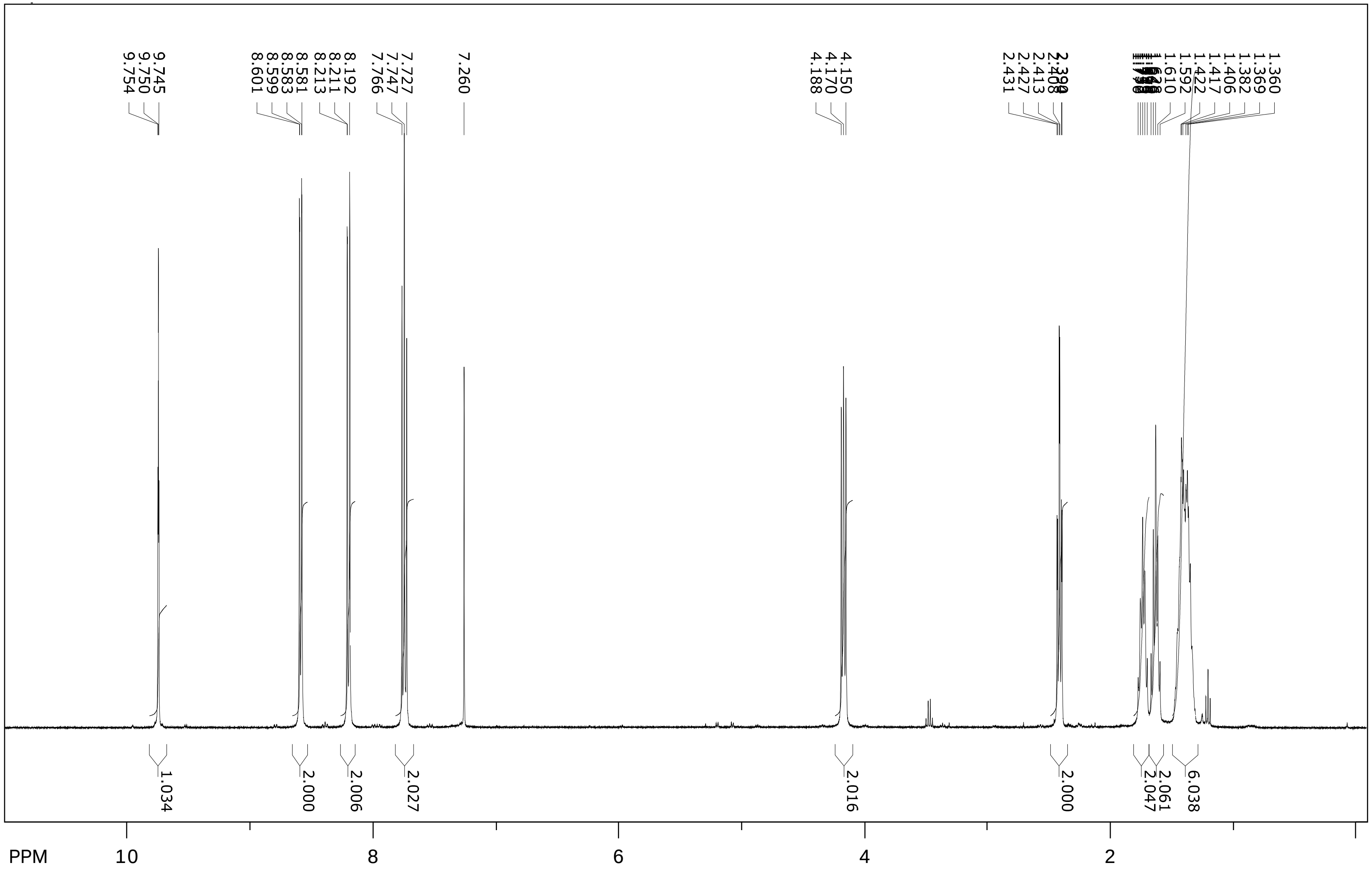

$^{13}$C NMR (CDCl$_3$, 100 MHz) of **4e** 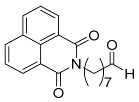

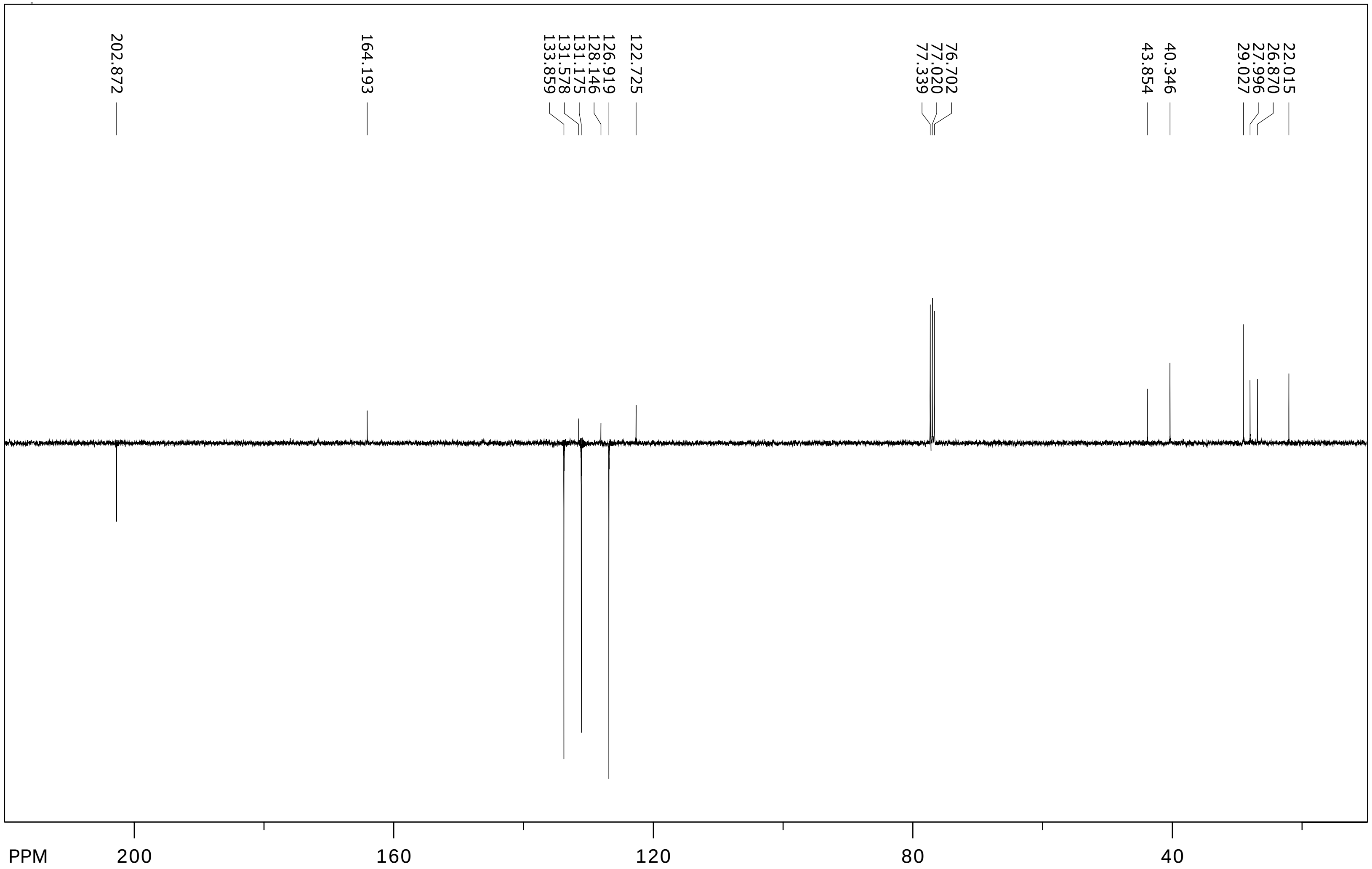

$^{1}$H NMR ($CDCl_3$, 500 MHz) of **5** 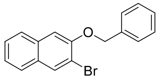


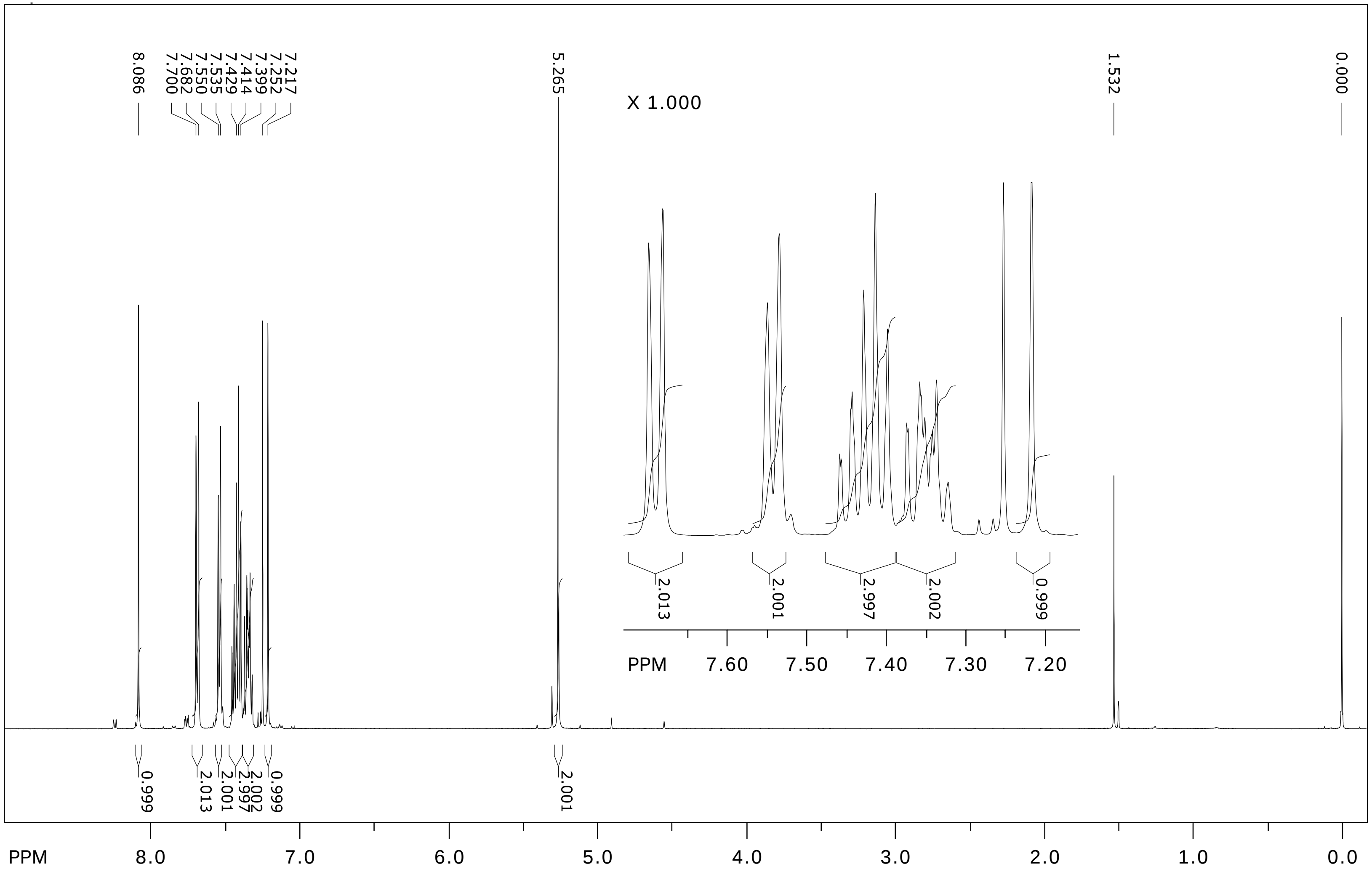

$^{13}C$ NMR ($CDCl_3$, 125 MHz) of **5** 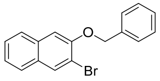


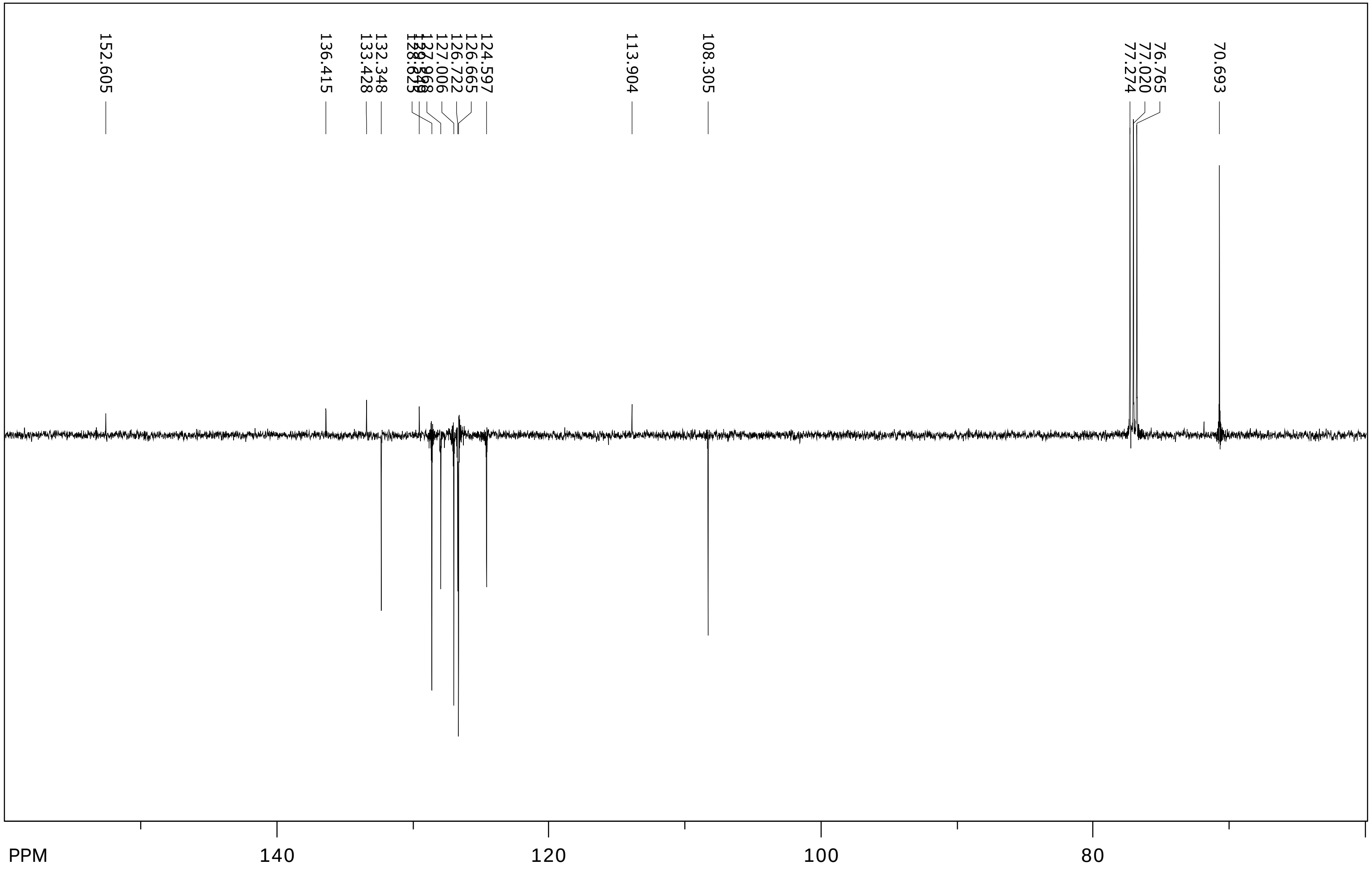

$^{1}$H NMR ($CDCl_3$, 400 MHz) of **7a**

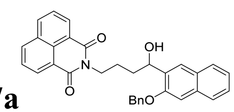


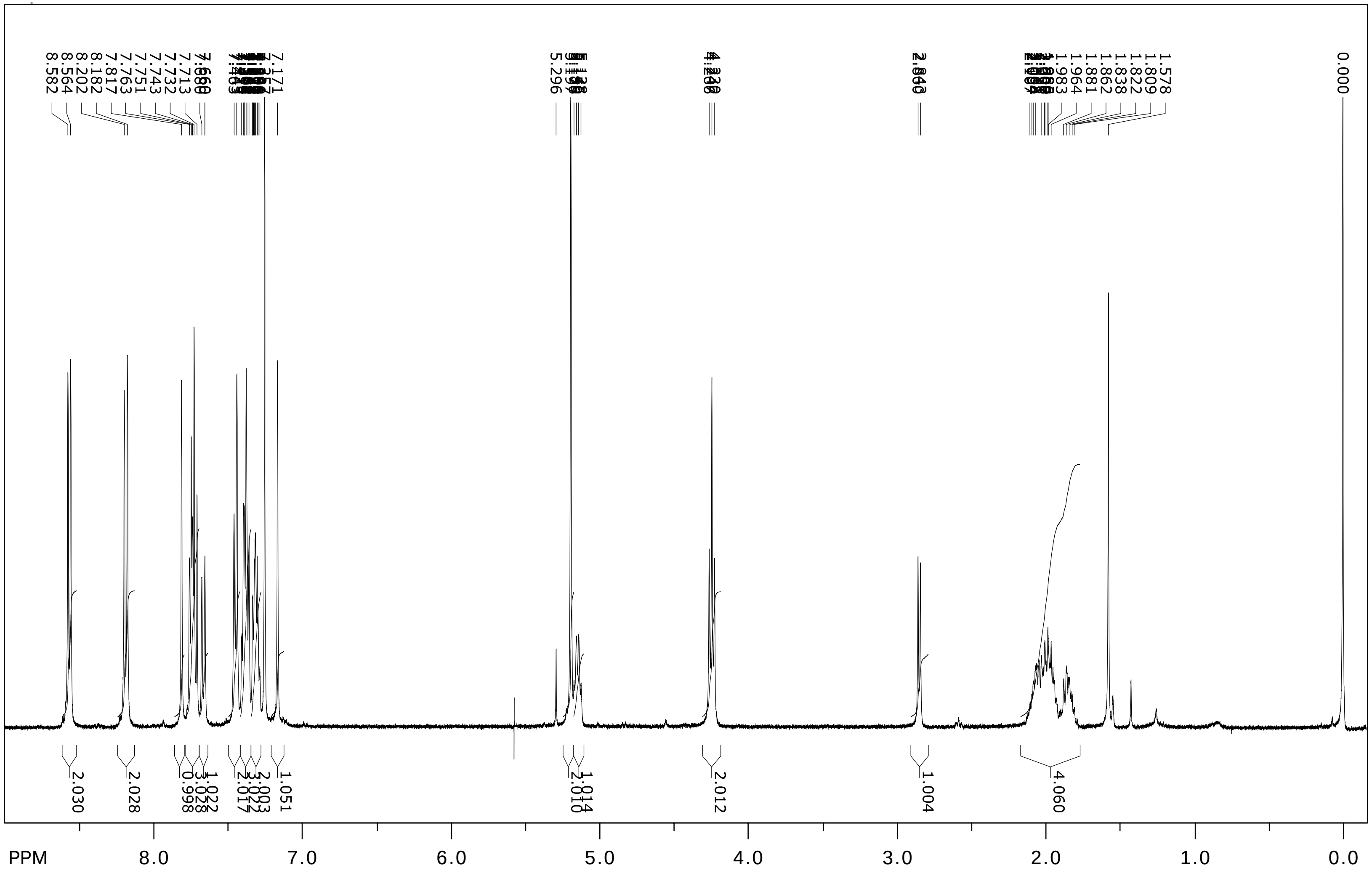

$^{13}C$ NMR ($CDCl_3$, 100 MHz) of **7a**

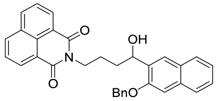


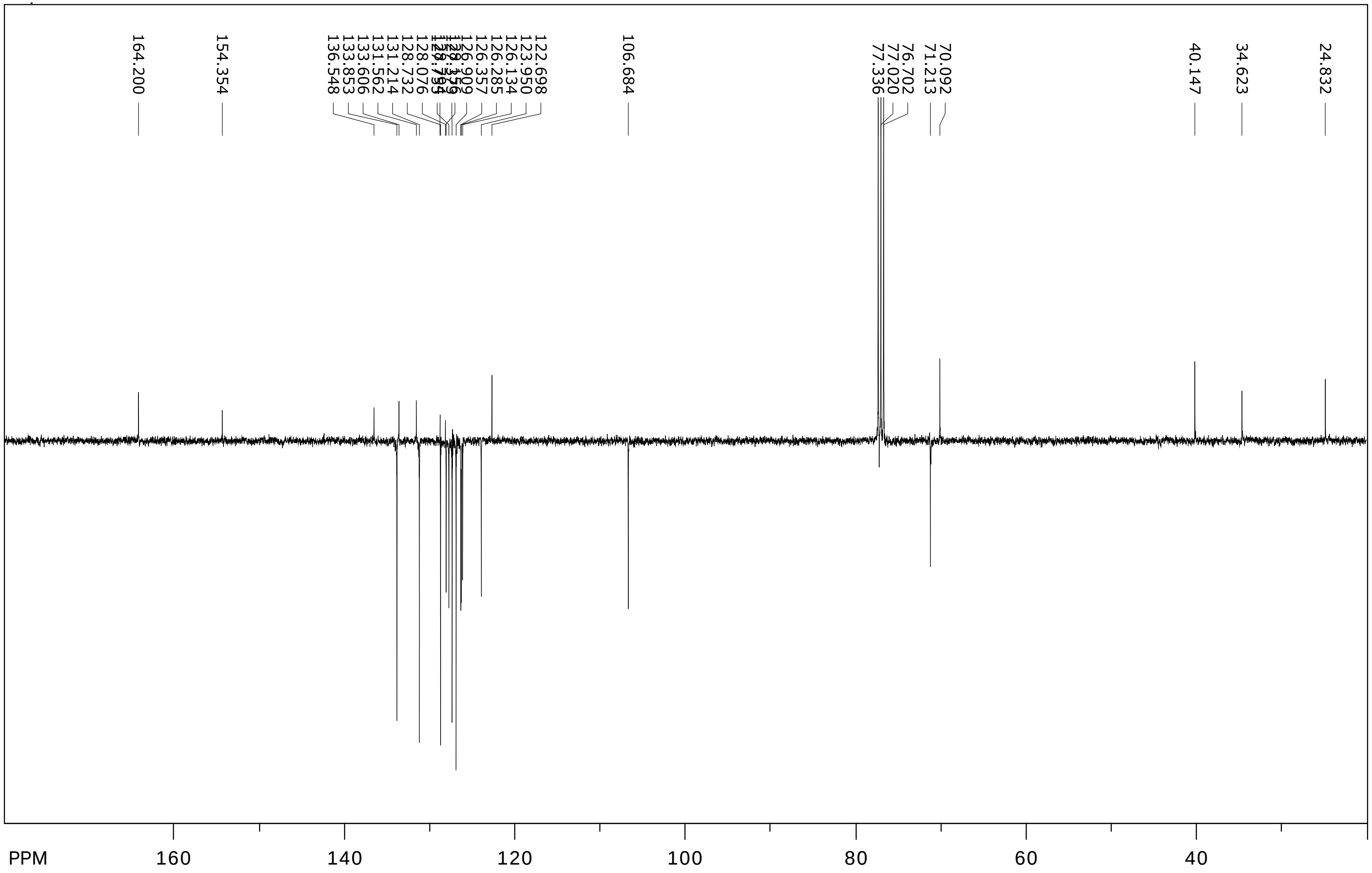

$^{1}$H NMR ($CDCl_3$, 400 MHz) of **7b**

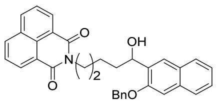


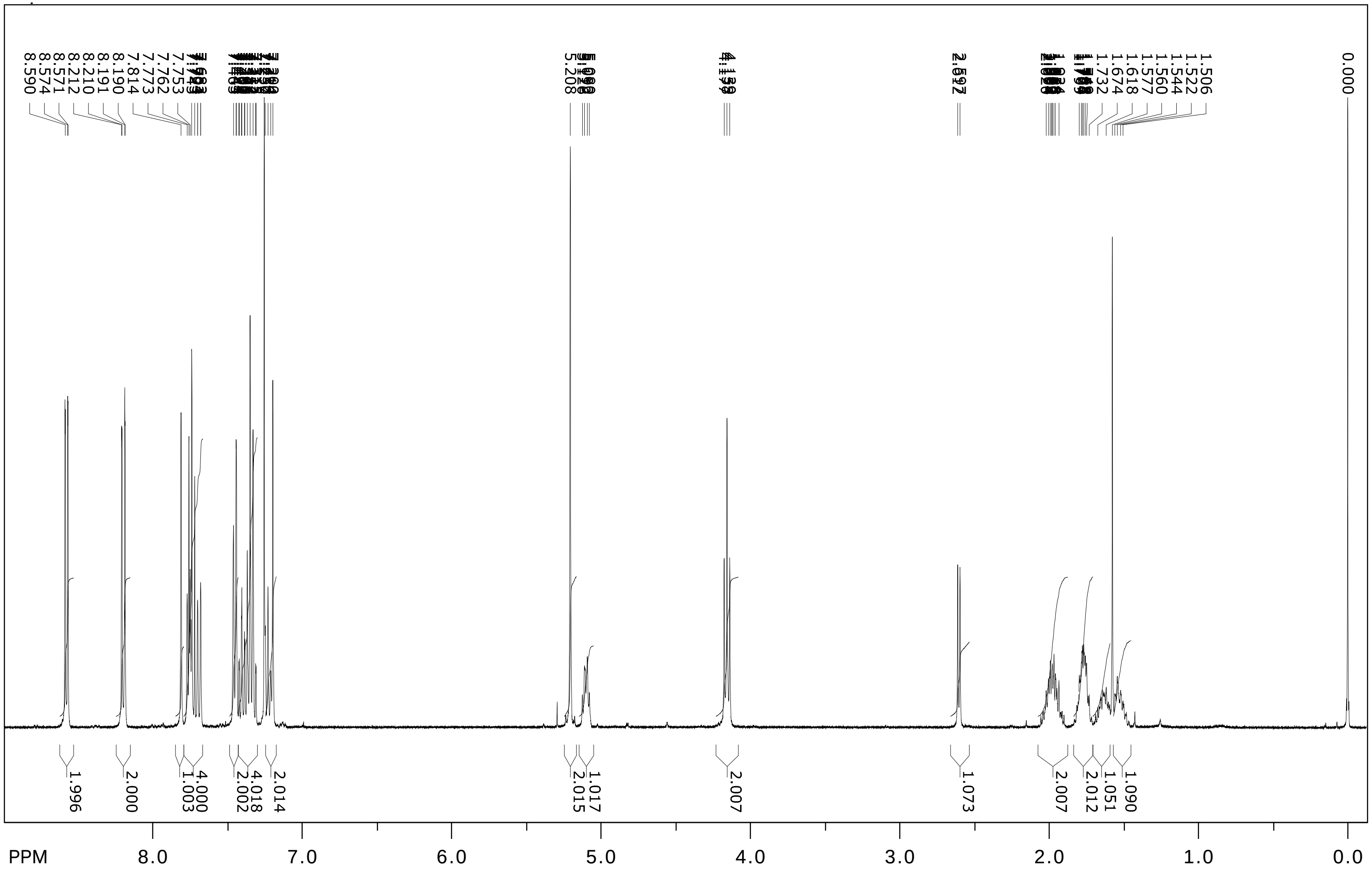

$^{13}$C NMR ($CDCl_3$, 100 MHz) of **7b** 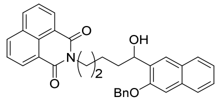

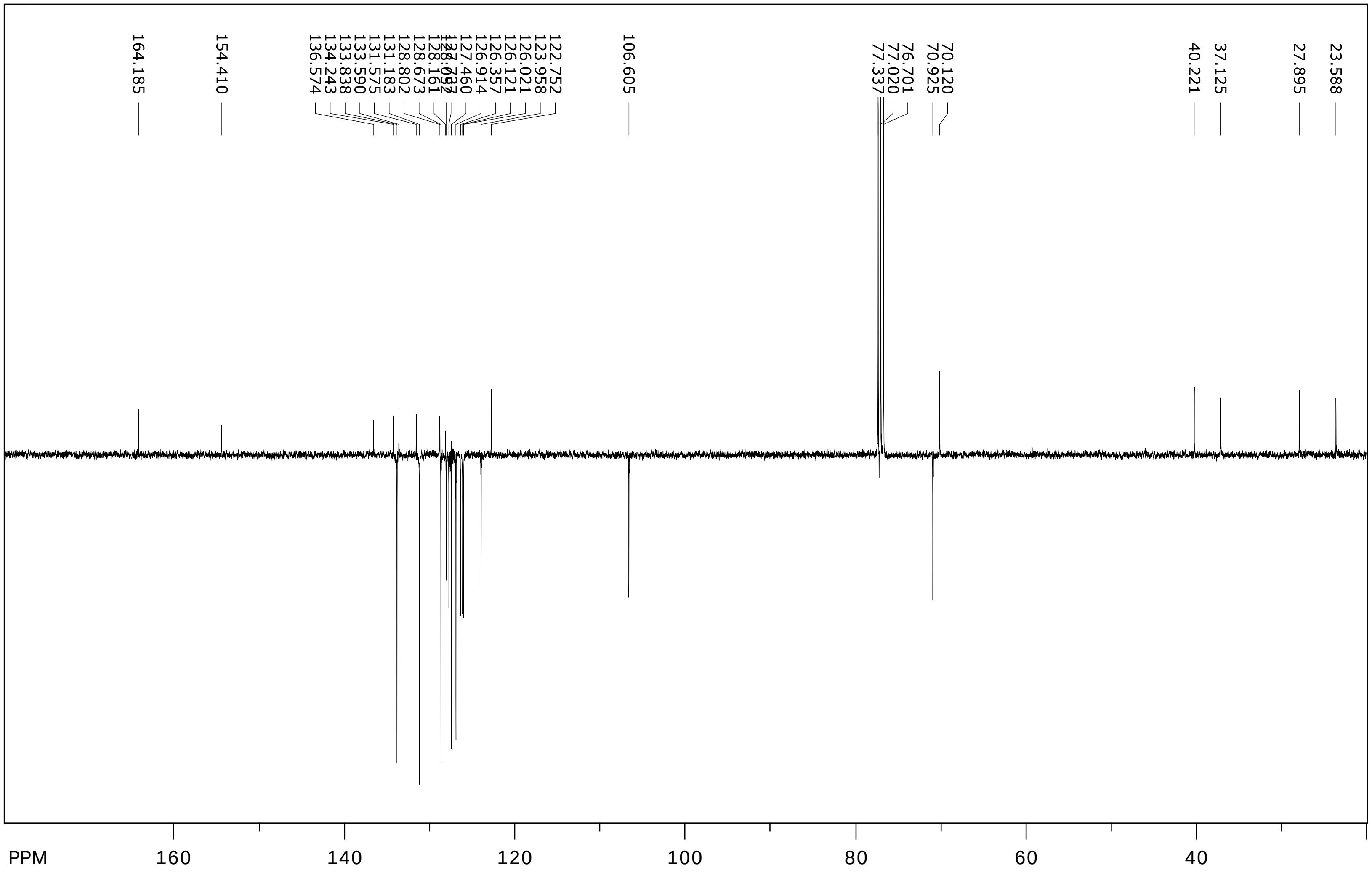

$^{1}$H NMR ($CDCl_3$, 400 MHz) of **7c**

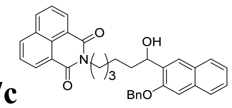


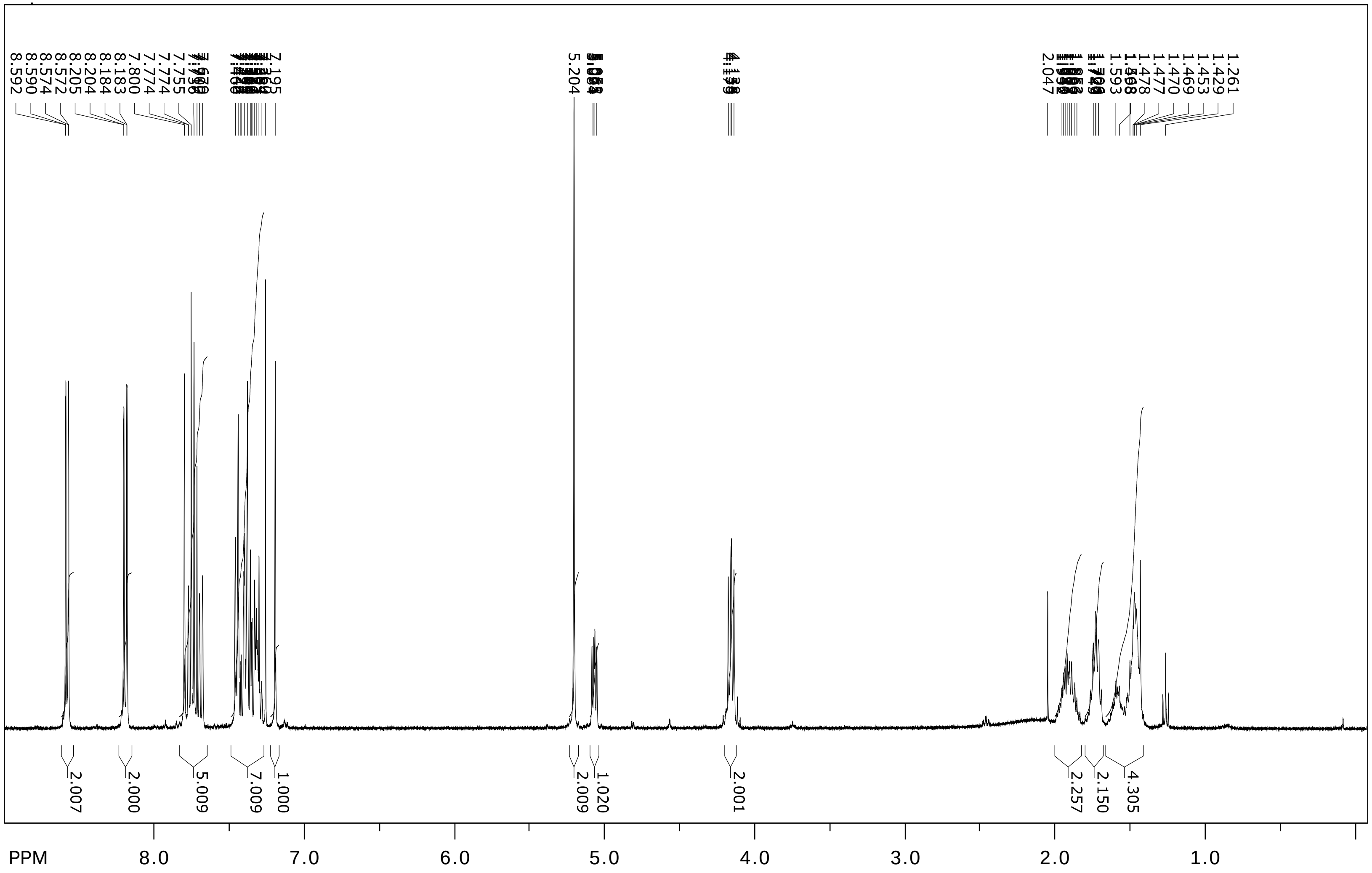

$^{13}C$ NMR ($CDCl_3$, 100 MHz) of **7c** 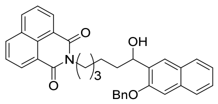


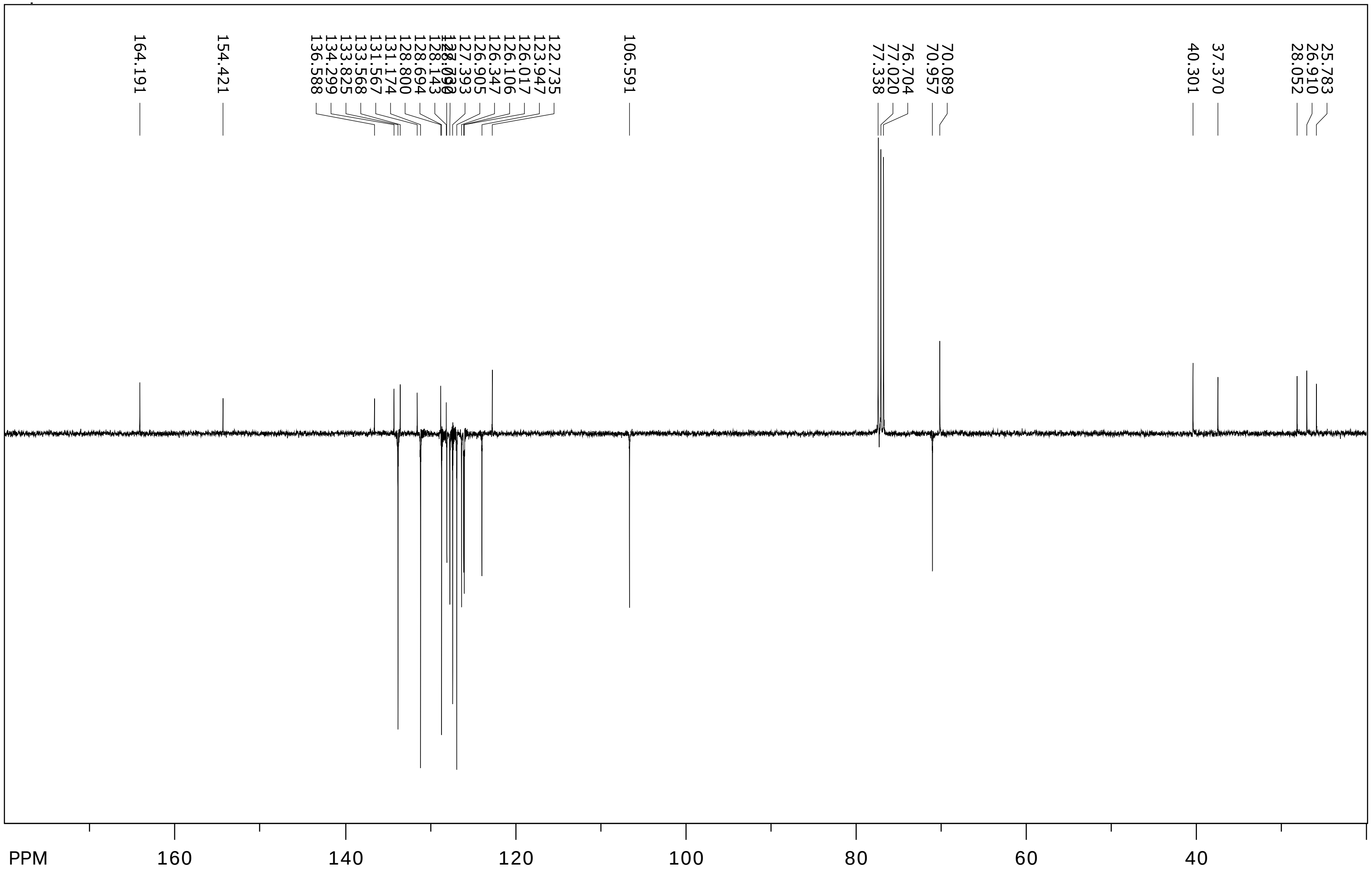

$^{1}H$ NMR ($CDCl_3$, 400 MHz) of **7d** 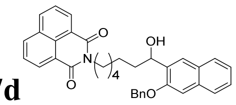


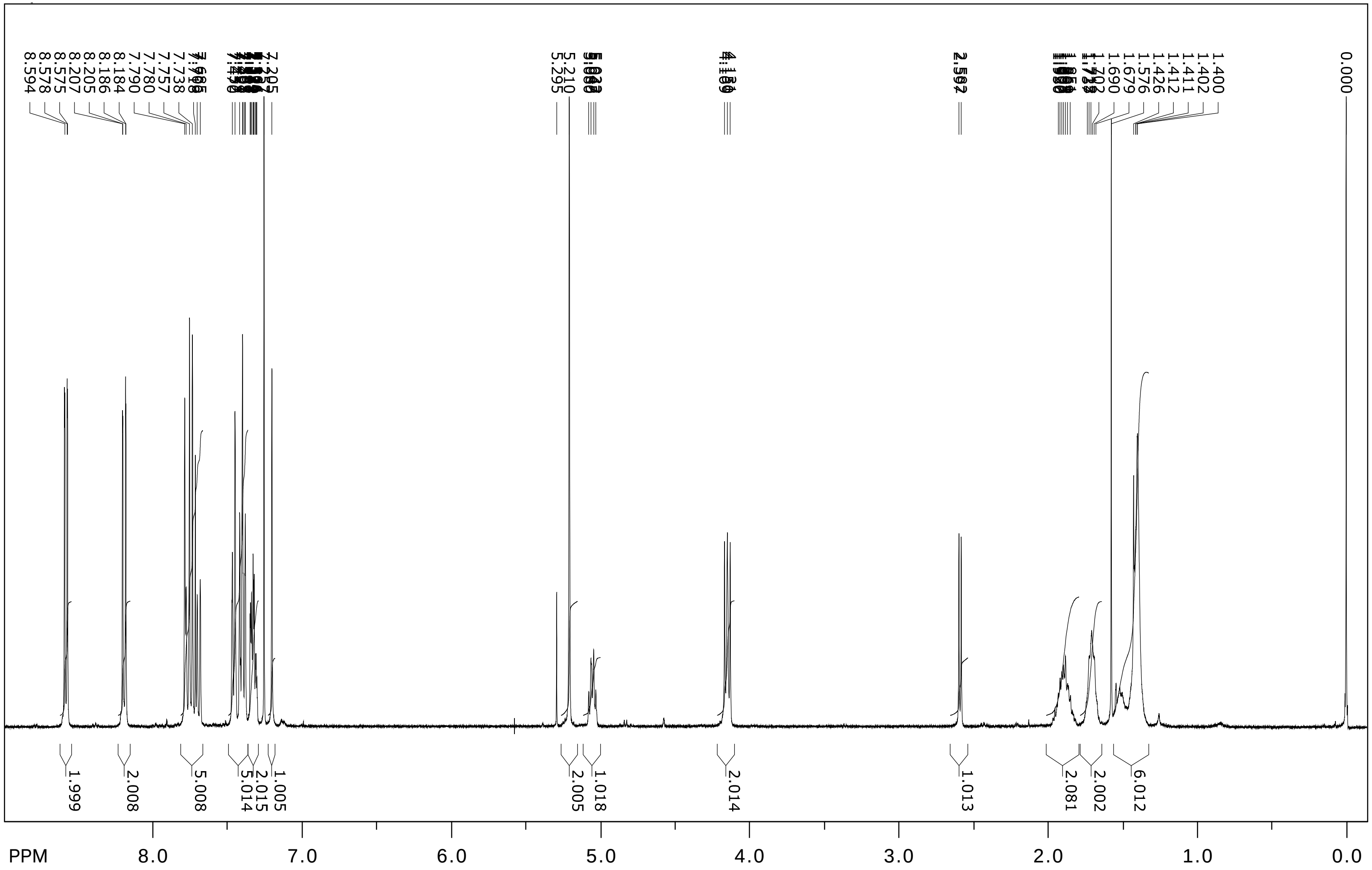

$^{13}$C NMR ($CDCl_3$, 100 MHz) of **7d** 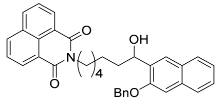


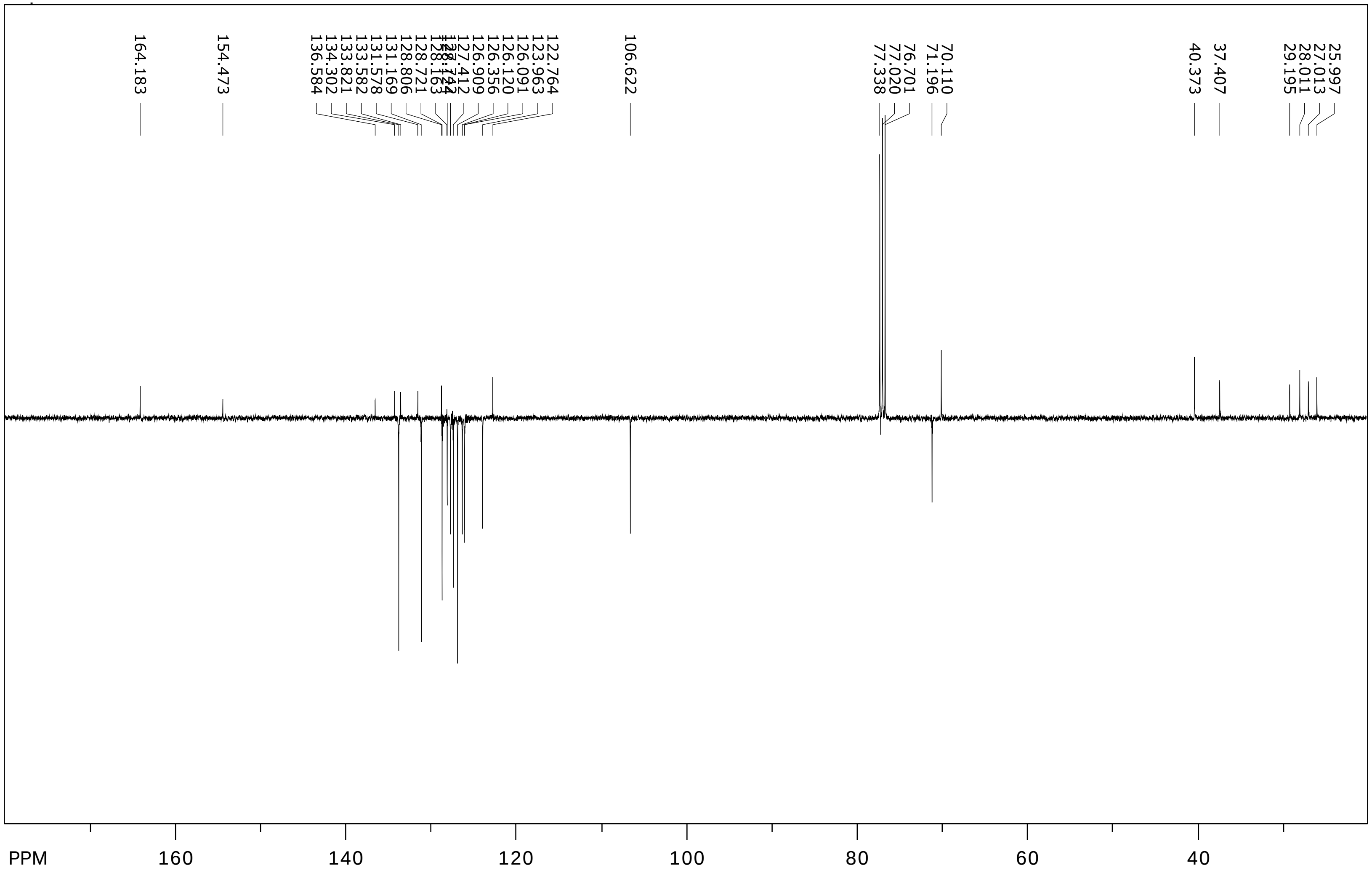

$^{1}$H NMR ($CDCl_3$, 400 MHz) of **7e** 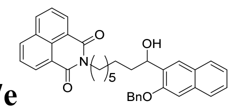


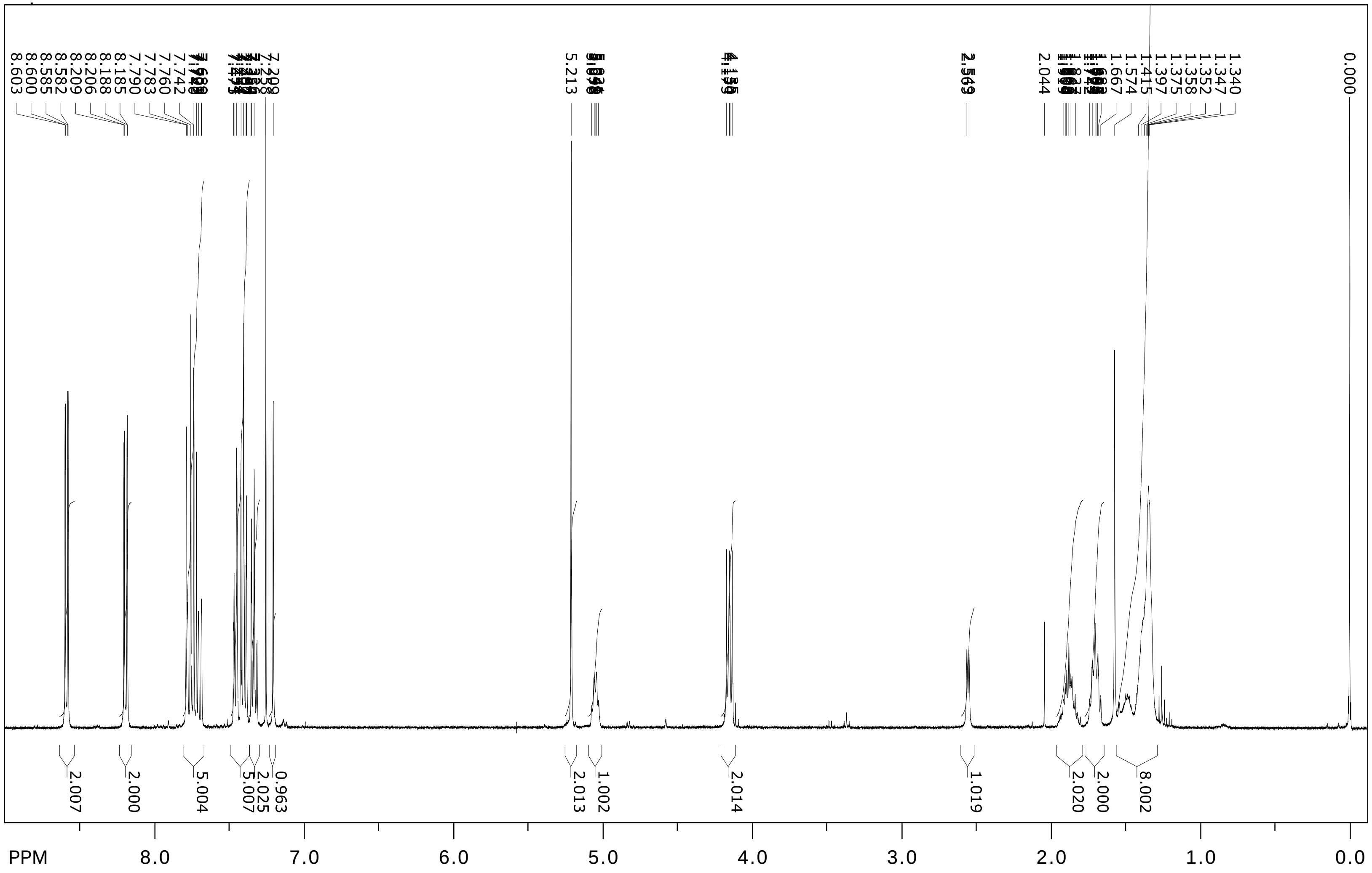

$^{13}C$ NMR ($CDCl_3$, 100 MHz) of **7e** 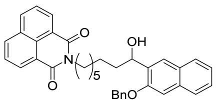


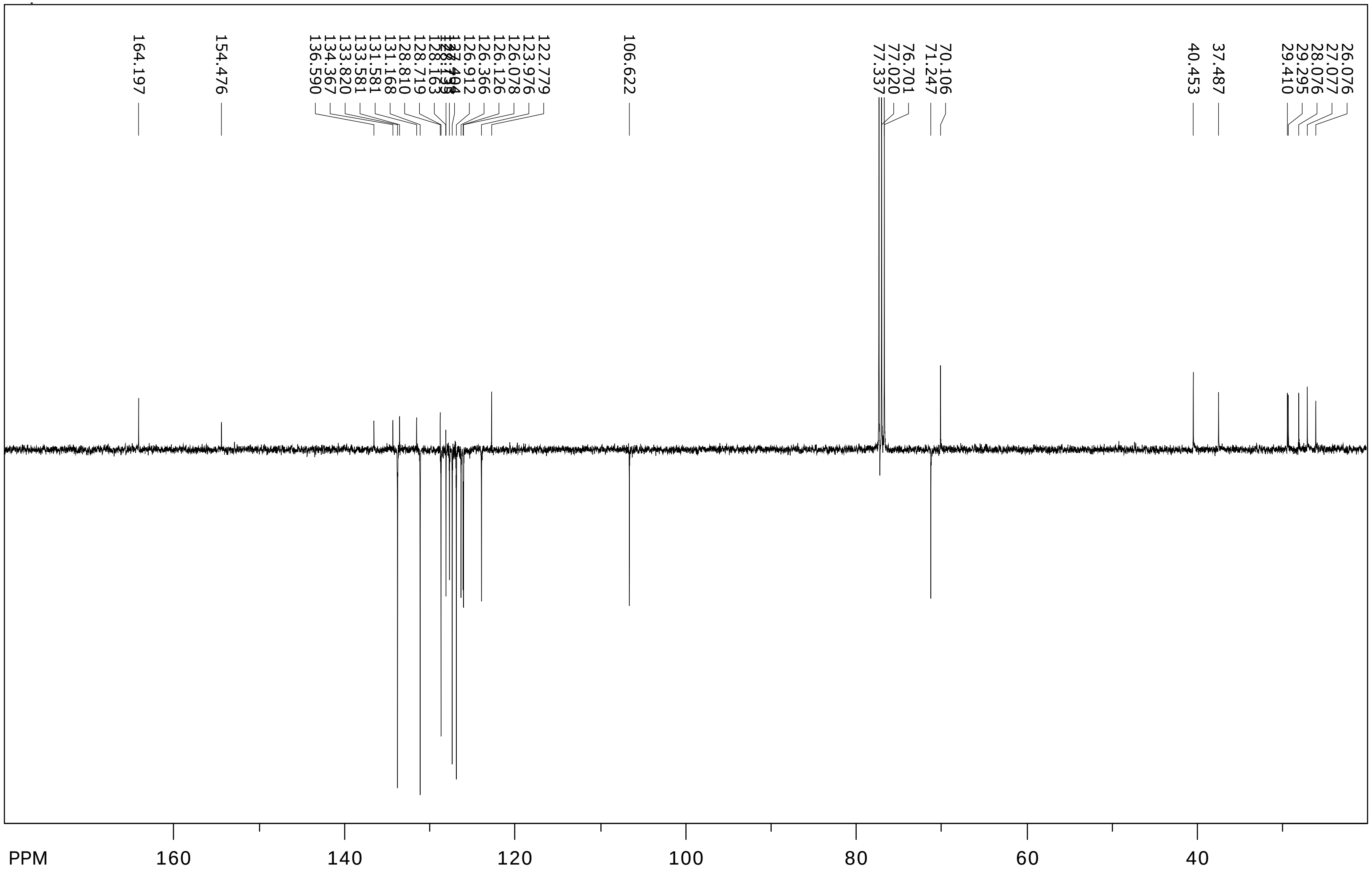

$^1$H NMR (DMSO-$d_6$, 400 MHz) of **1a**

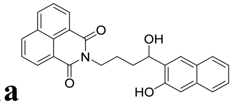

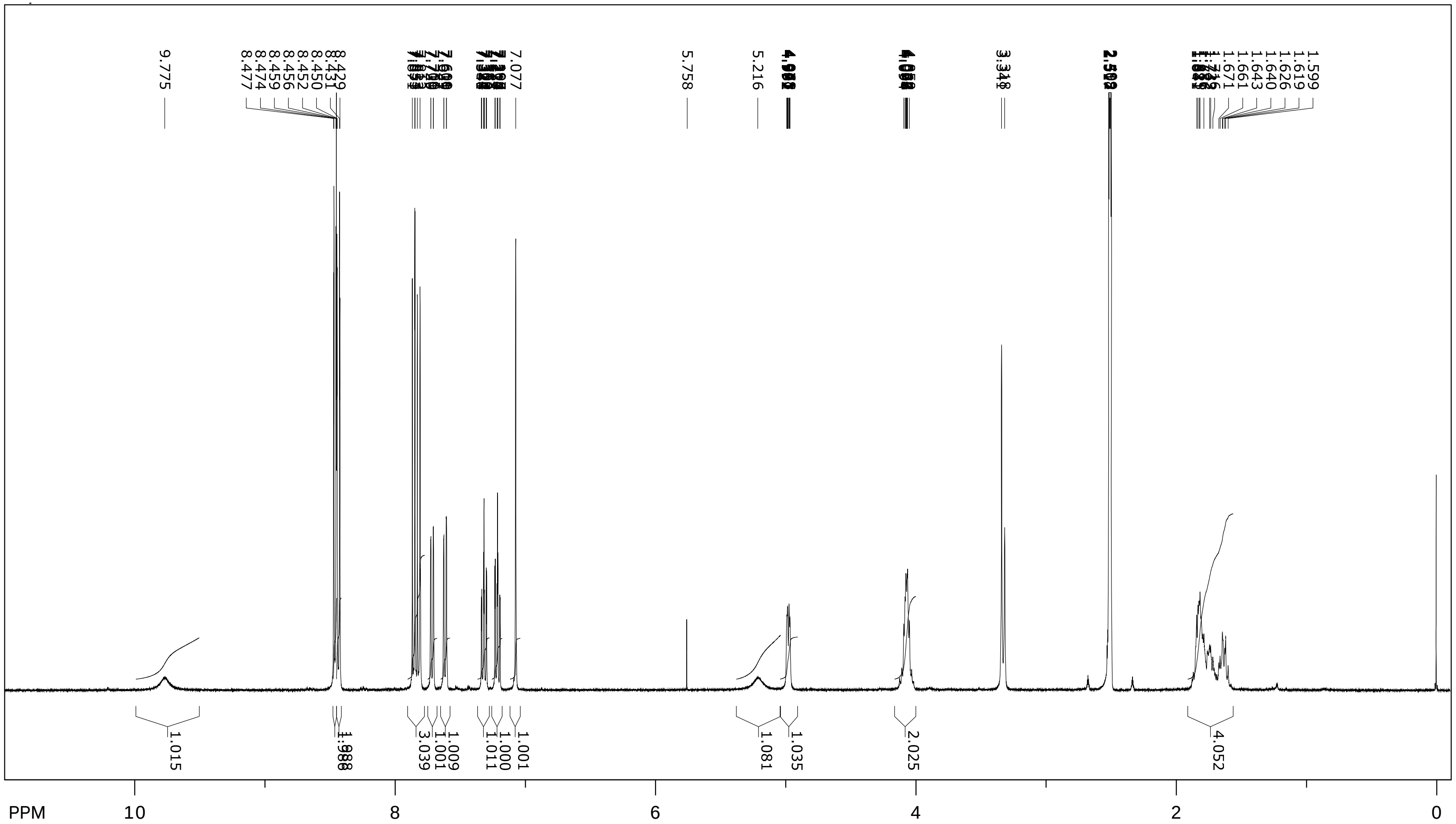

$^{13}$C NMR (DMSO-$d_6$, 100 MHz) of **1a**

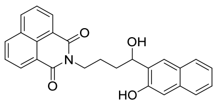

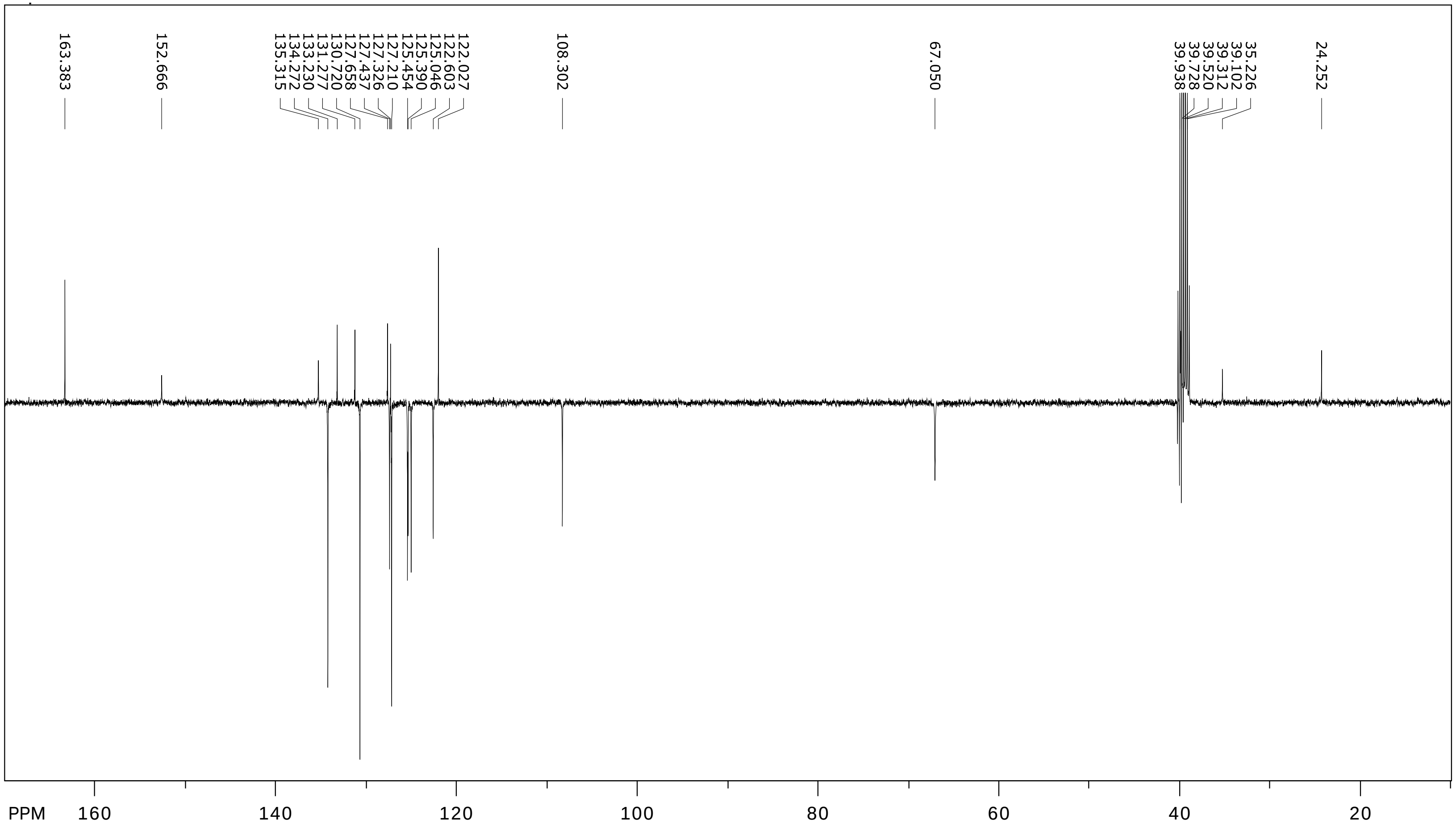

$^{1}$H NMR (DMSO-$d_6$, 600 MHz) of **1b**

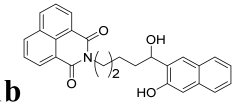

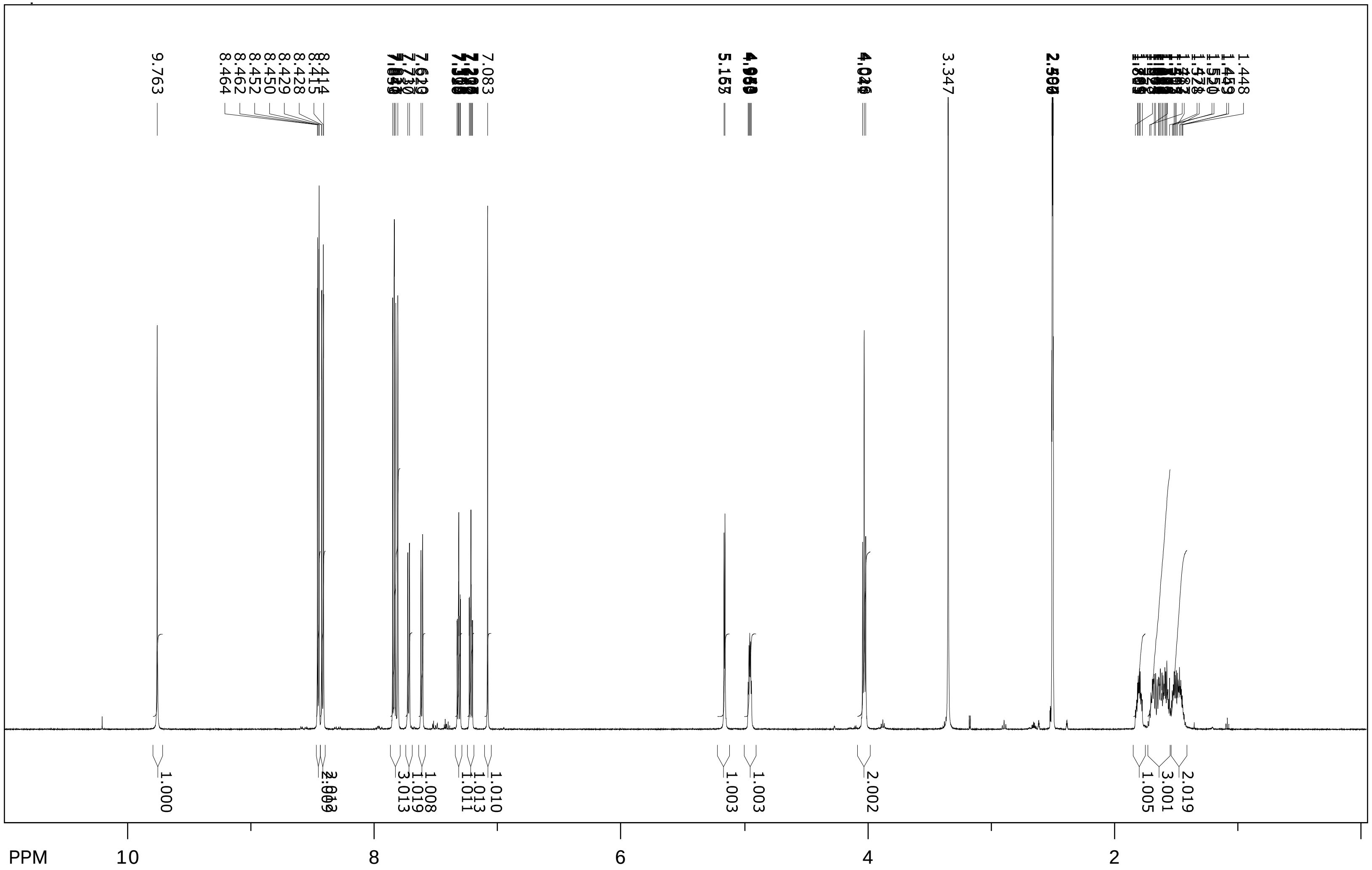

$^{13}$C NMR (DMSO-$d_6$, 150 MHz) of **1b** 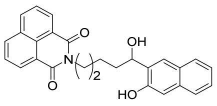


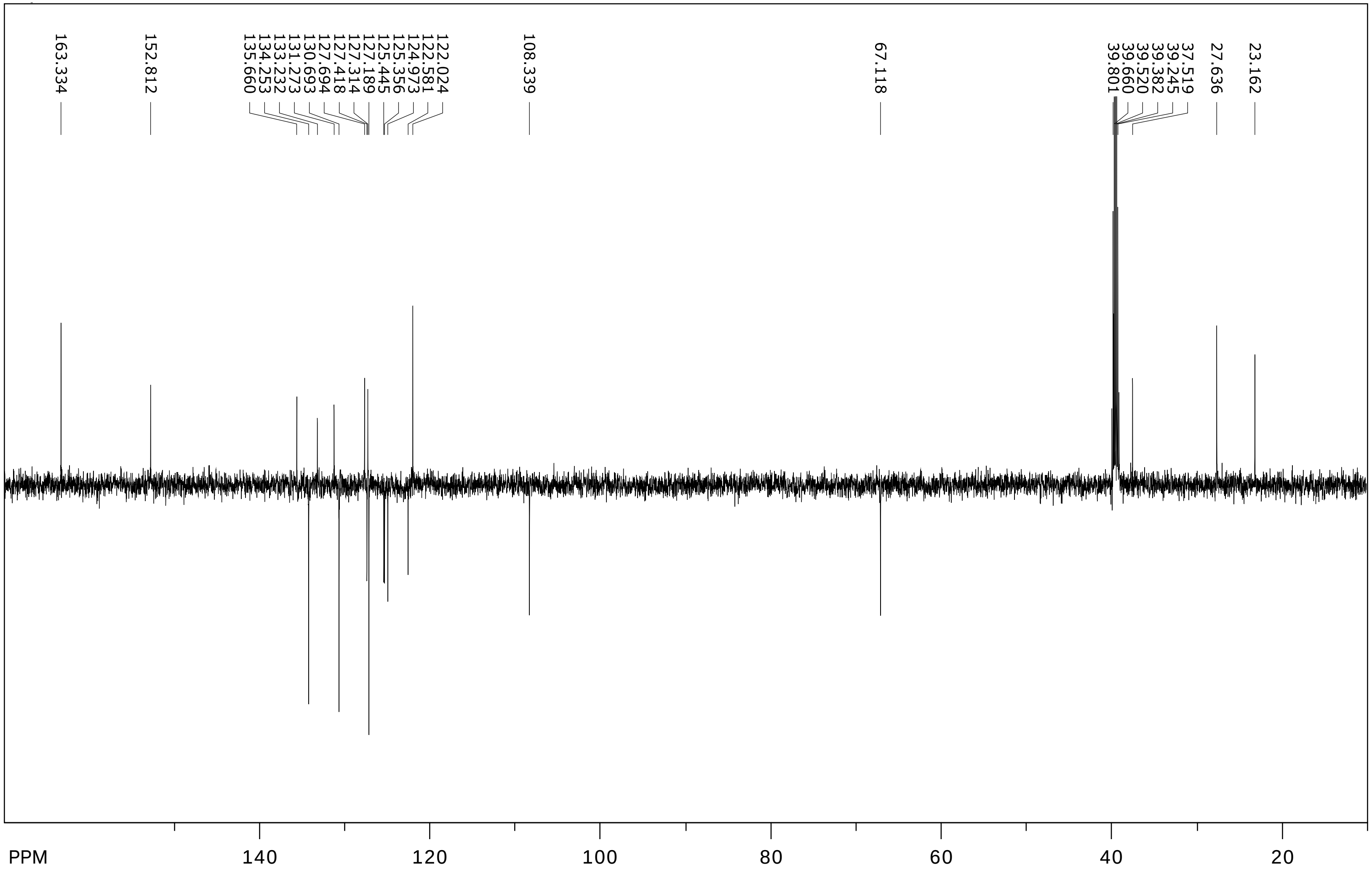

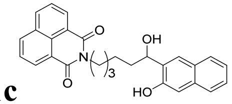


$^{1}$H NMR (DMSO-$d_6$, 400 MHz) of **1c**

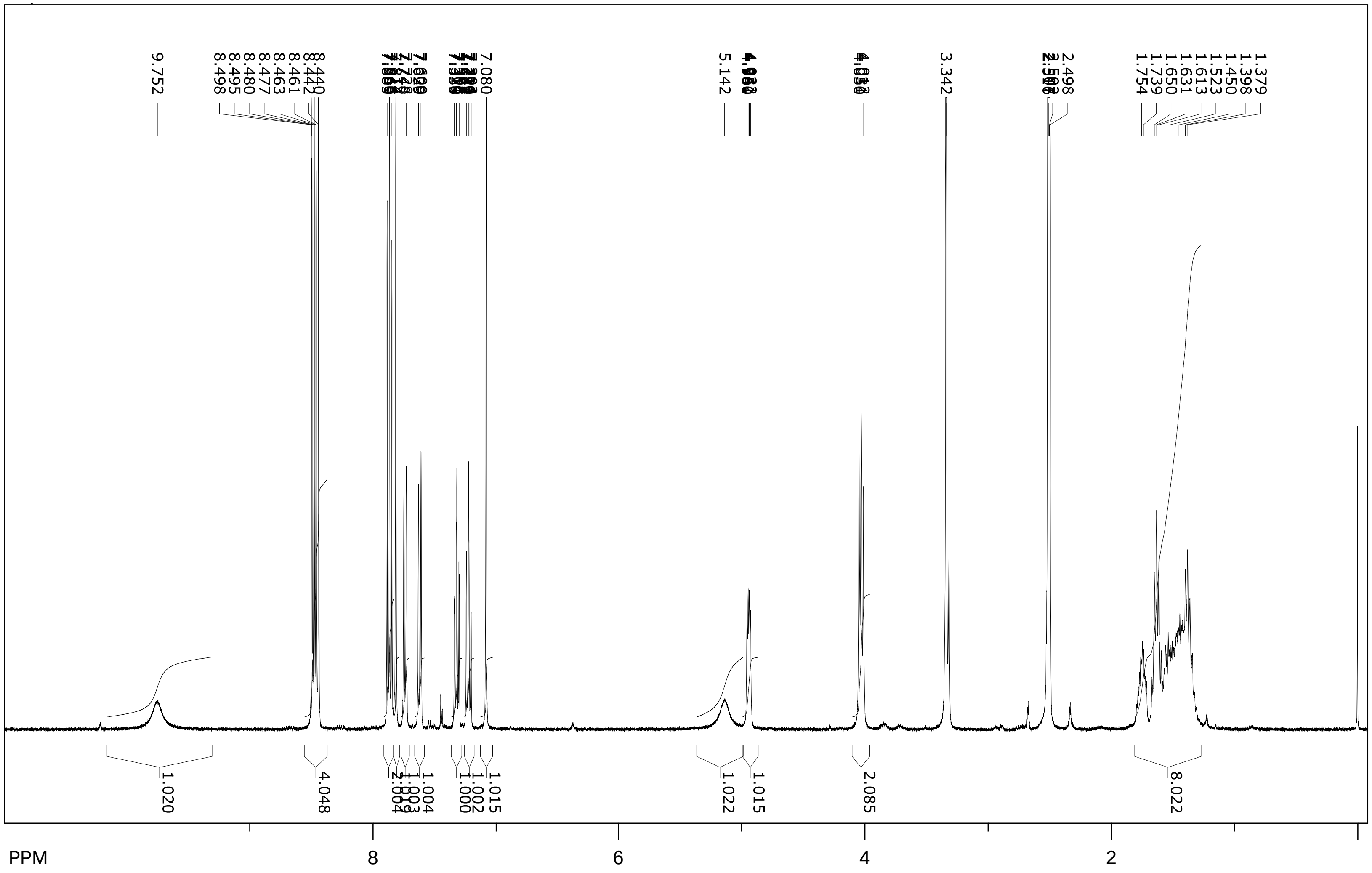

$^{13}C$ NMR (DMSO-$d_6$, 100 MHz) of **1c**

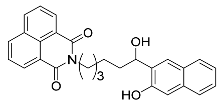


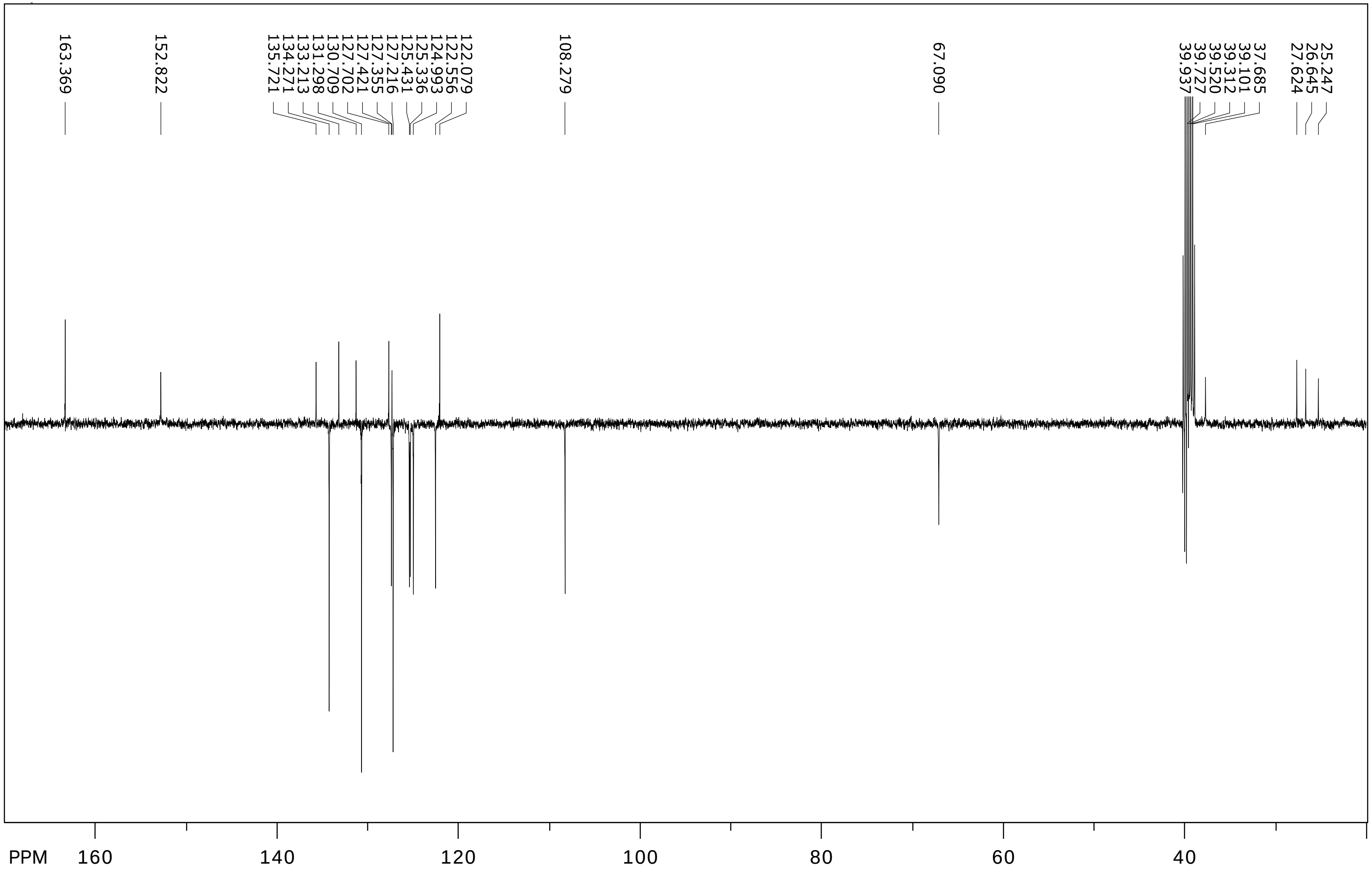

$^{1}$H NMR (DMSO-$d_6$, 600 MHz) of **1d** 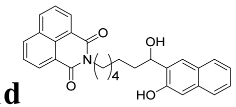



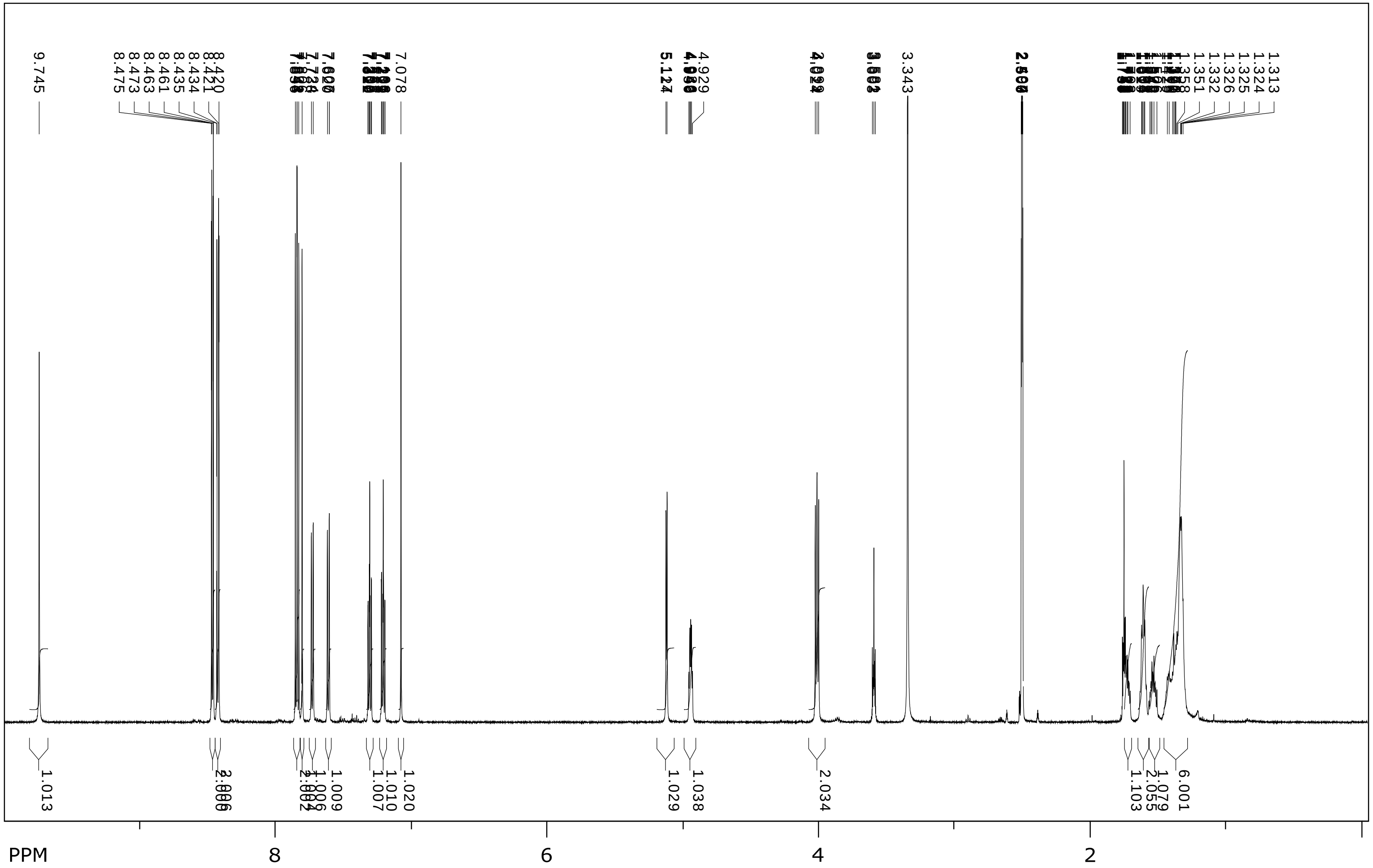

$^{13}C$ NMR (DMSO-$d_6$, 150 MHz) of **1d** 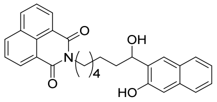

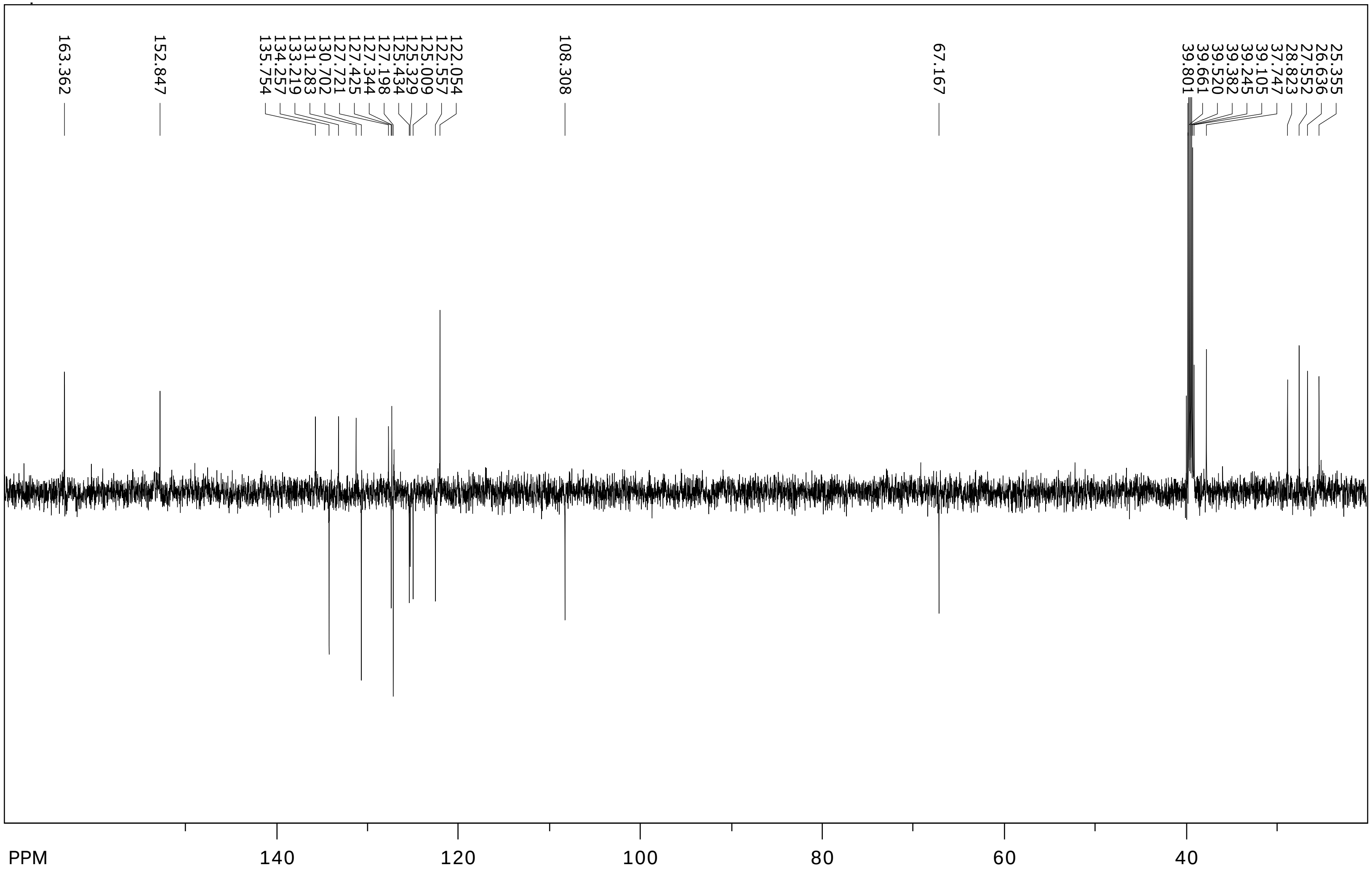

$^{1}$H NMR (DMSO-$d_6$, 600 MHz) of **1e**

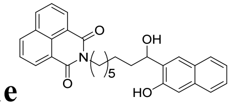

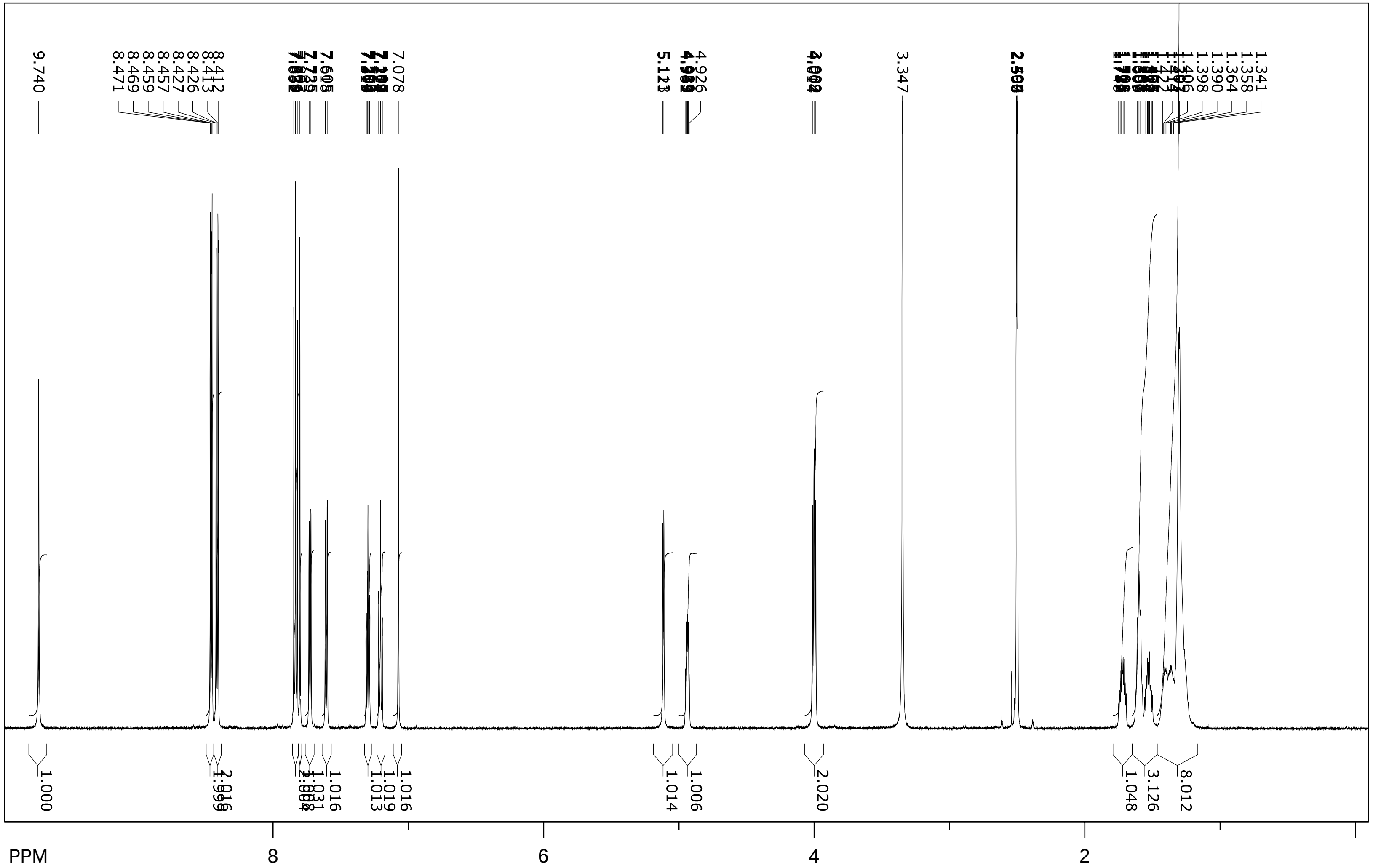

$^{13}$C NMR (DMSO-$d_6$, 150 MHz) of **1e**

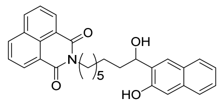

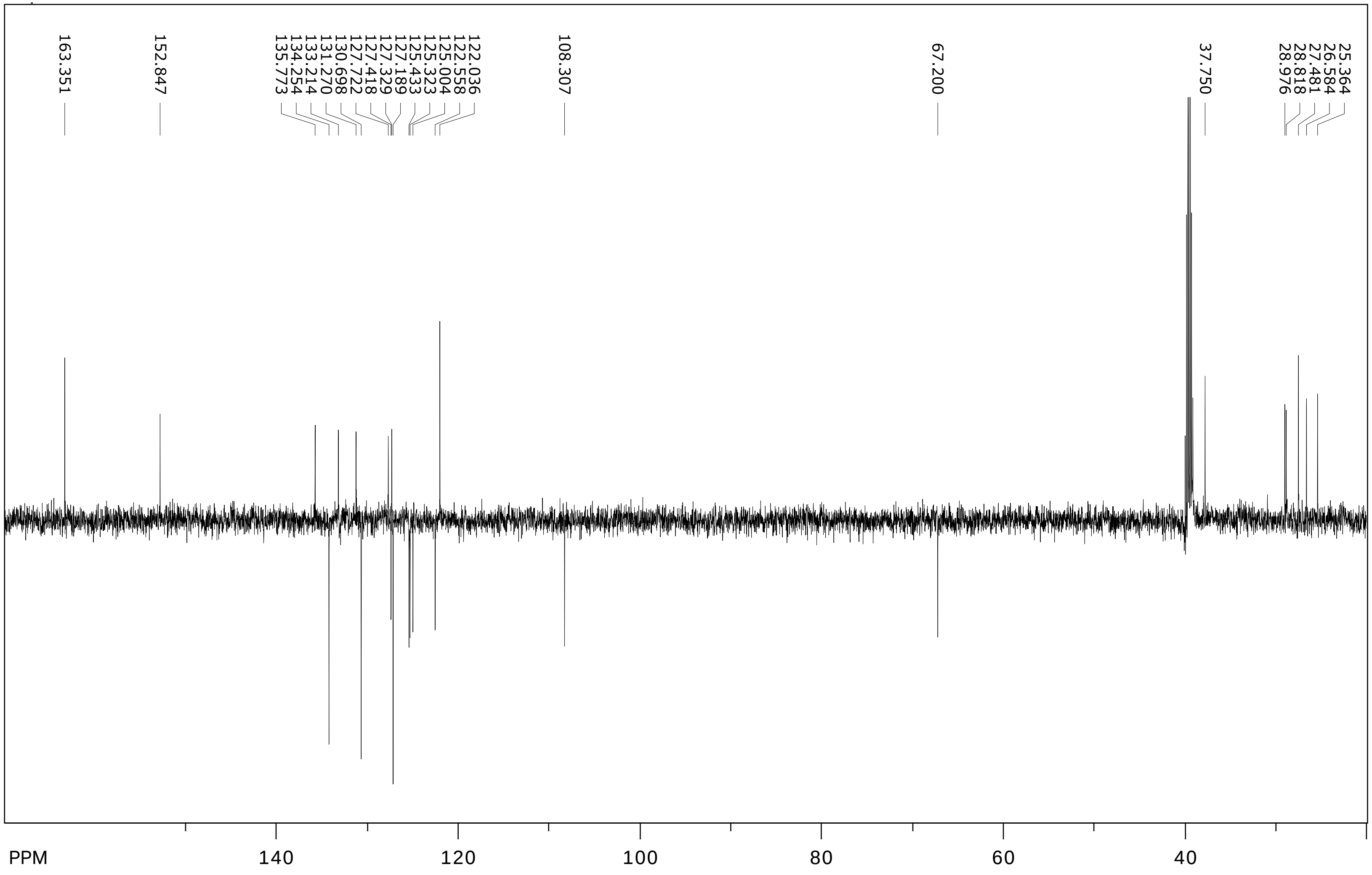

$^{1}$H NMR ($CDCl_3$, 600 MHz) of **1a-OMe** 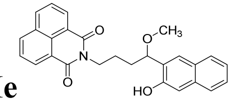

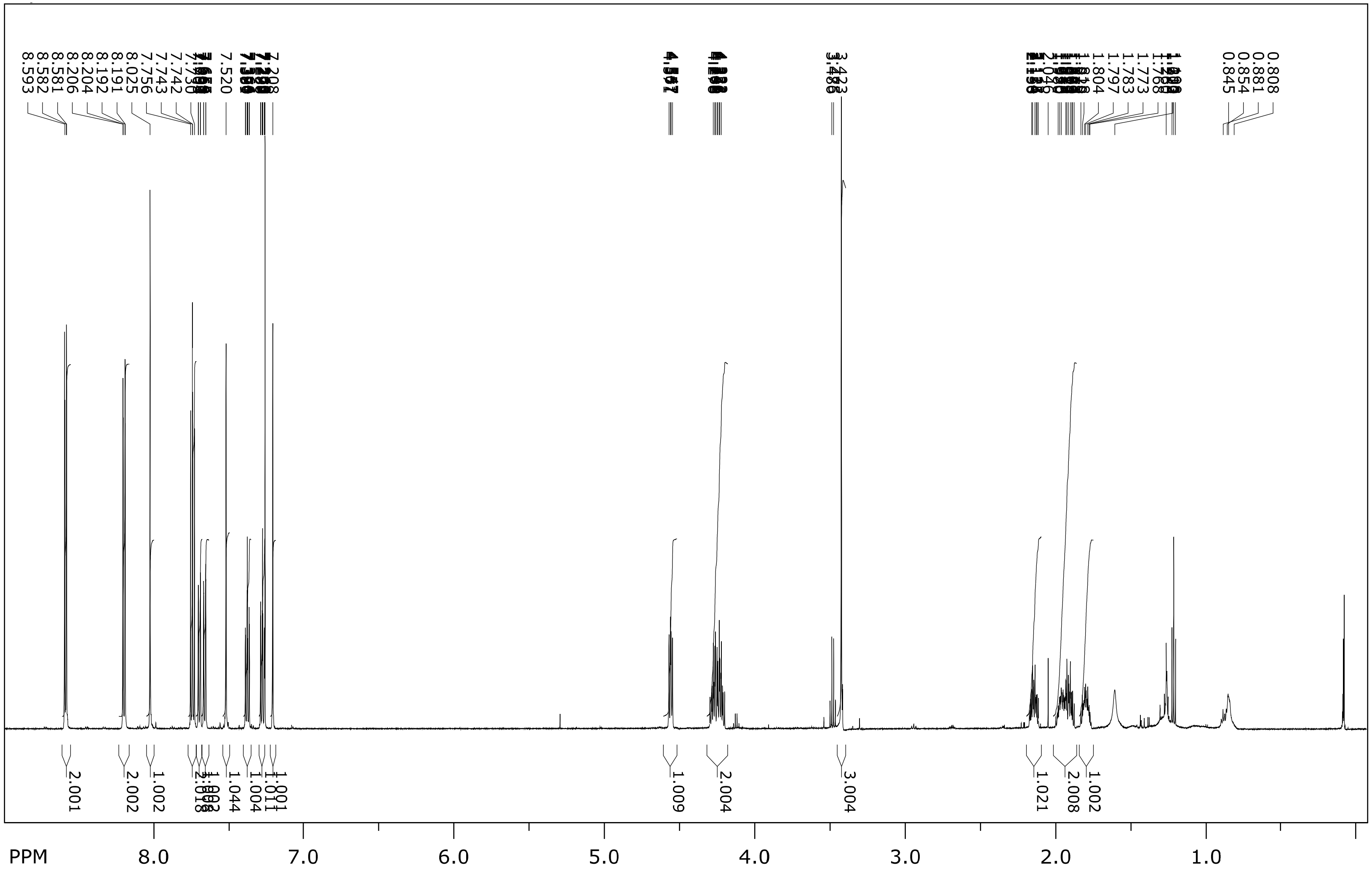

$^{13}$C NMR ($CDCl_3$, 150 MHz) of **1a-OMe** 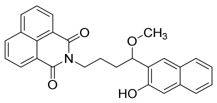

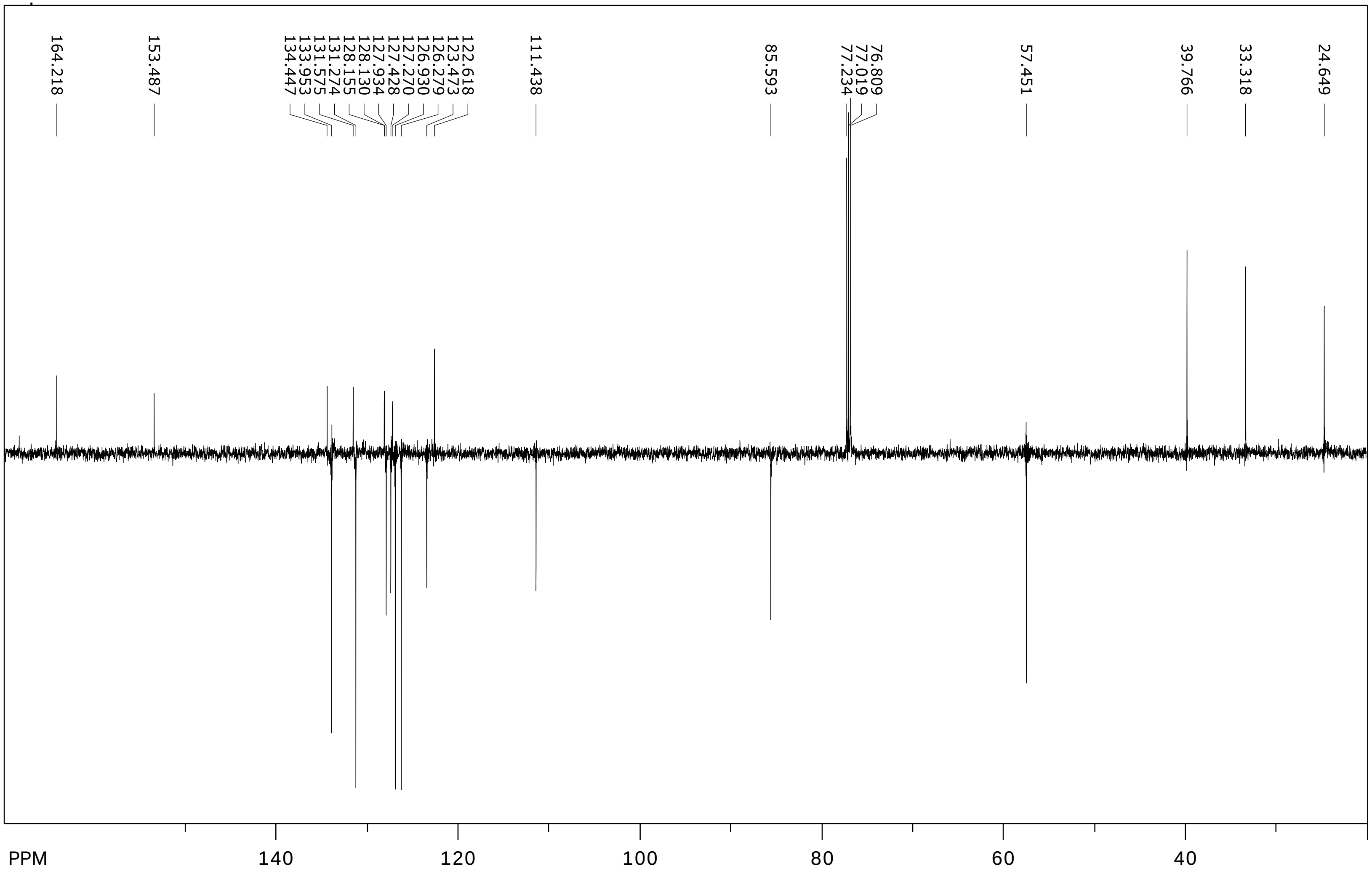

$^{1}$H NMR ($CDCl_3$, 600 MHz) of **1b-OMe**

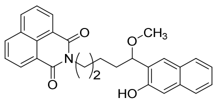

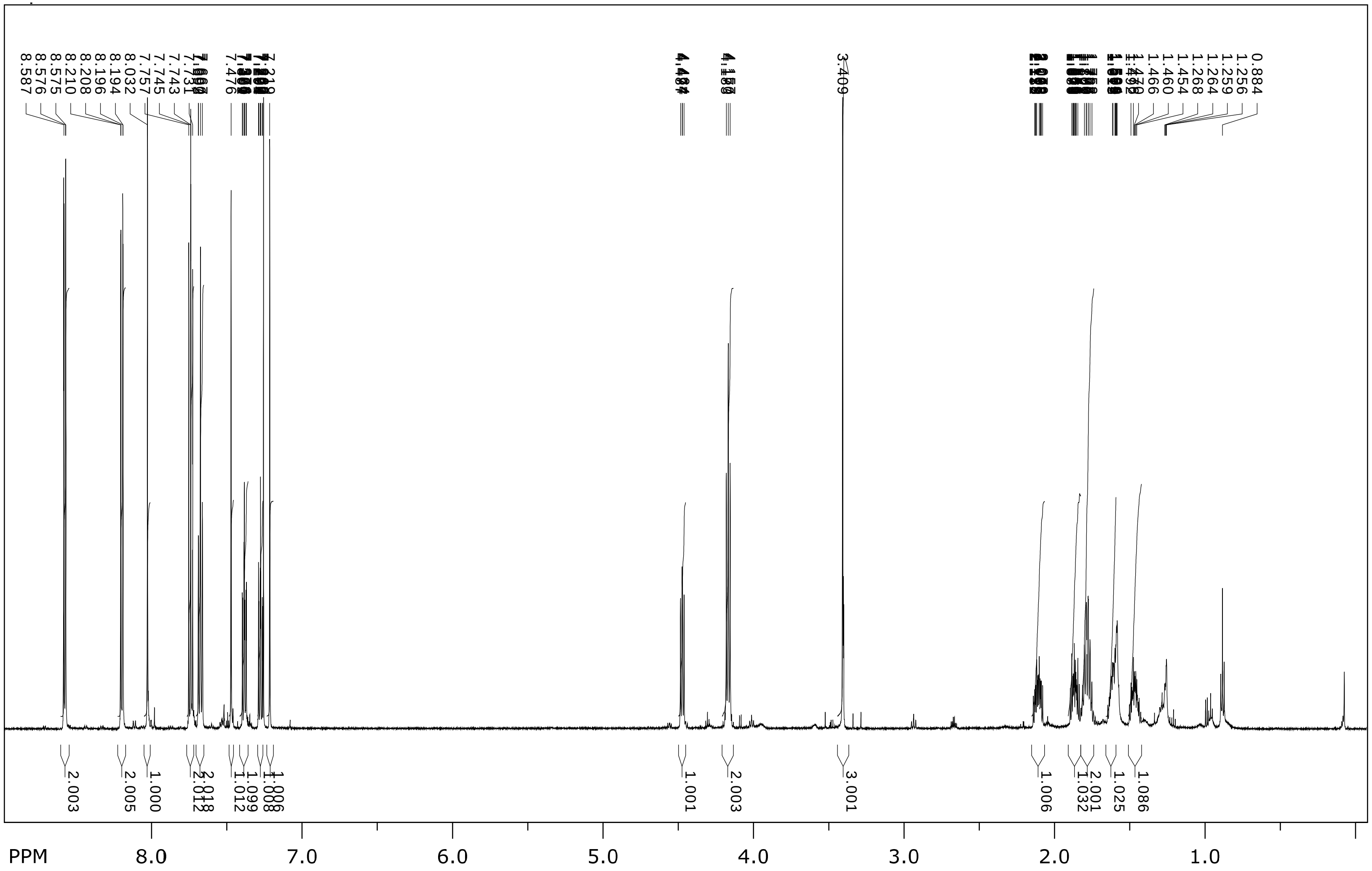

$^{13}$C NMR (CDCl$_3$, 150 MHz) of **1b-OMe**

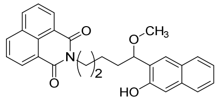


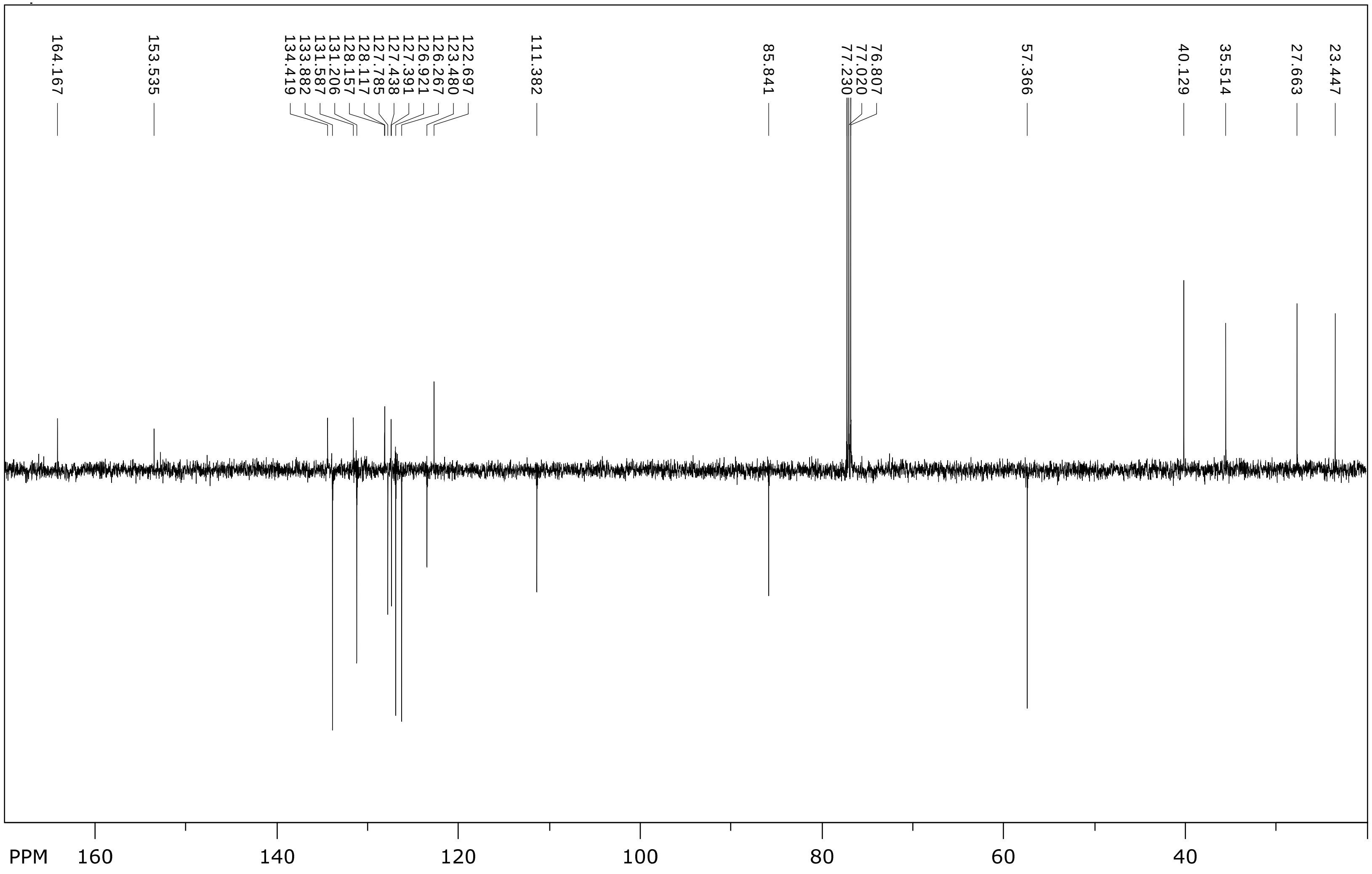

$^{1}$H NMR ($CDCl_3$, 600 MHz) of **1c-OMe**

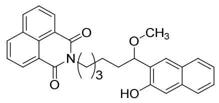


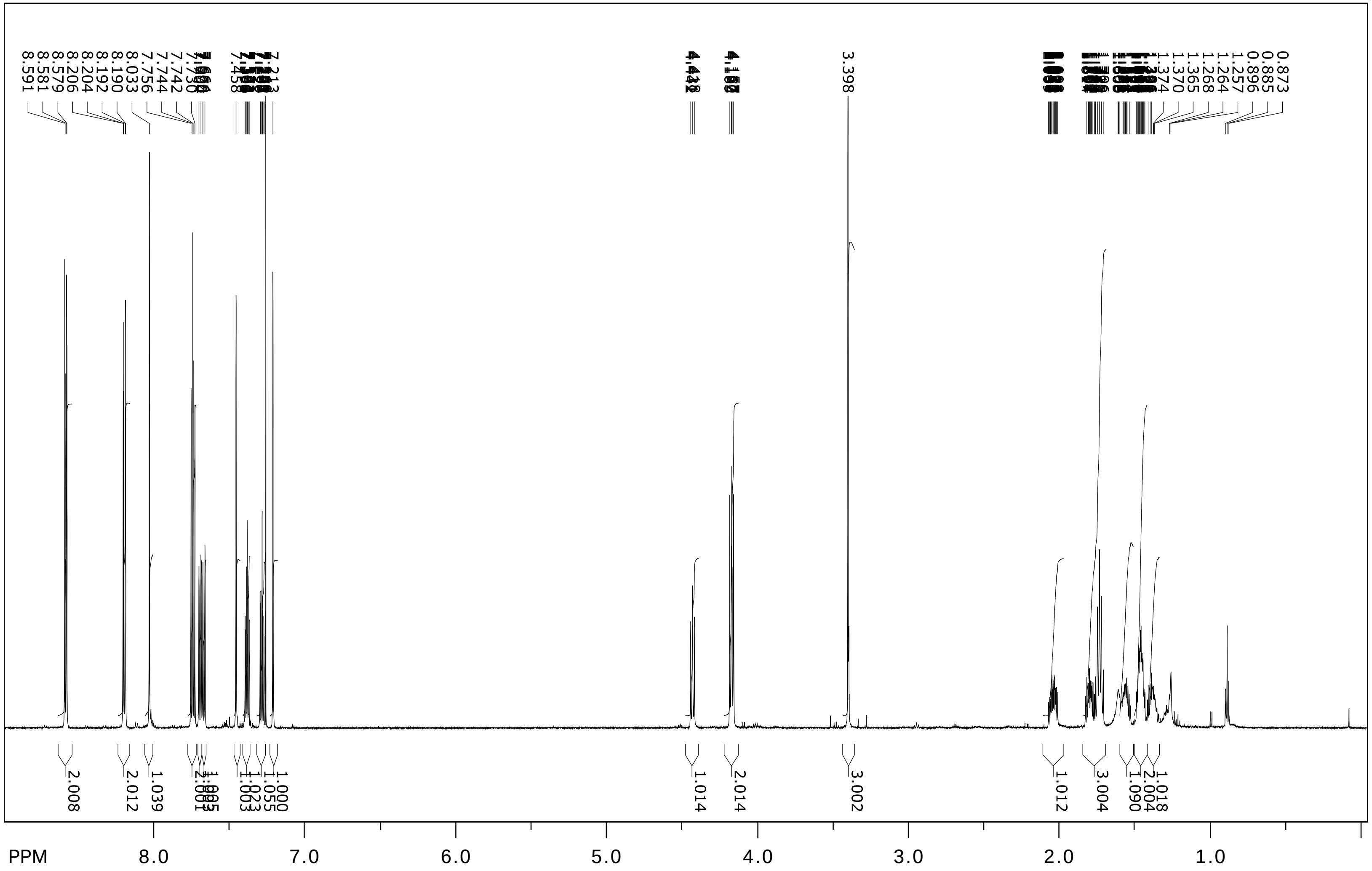

$^{13}C$ NMR ($CDCl_3$, 150 MHz) of **1c-OMe**

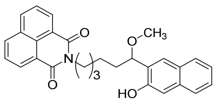


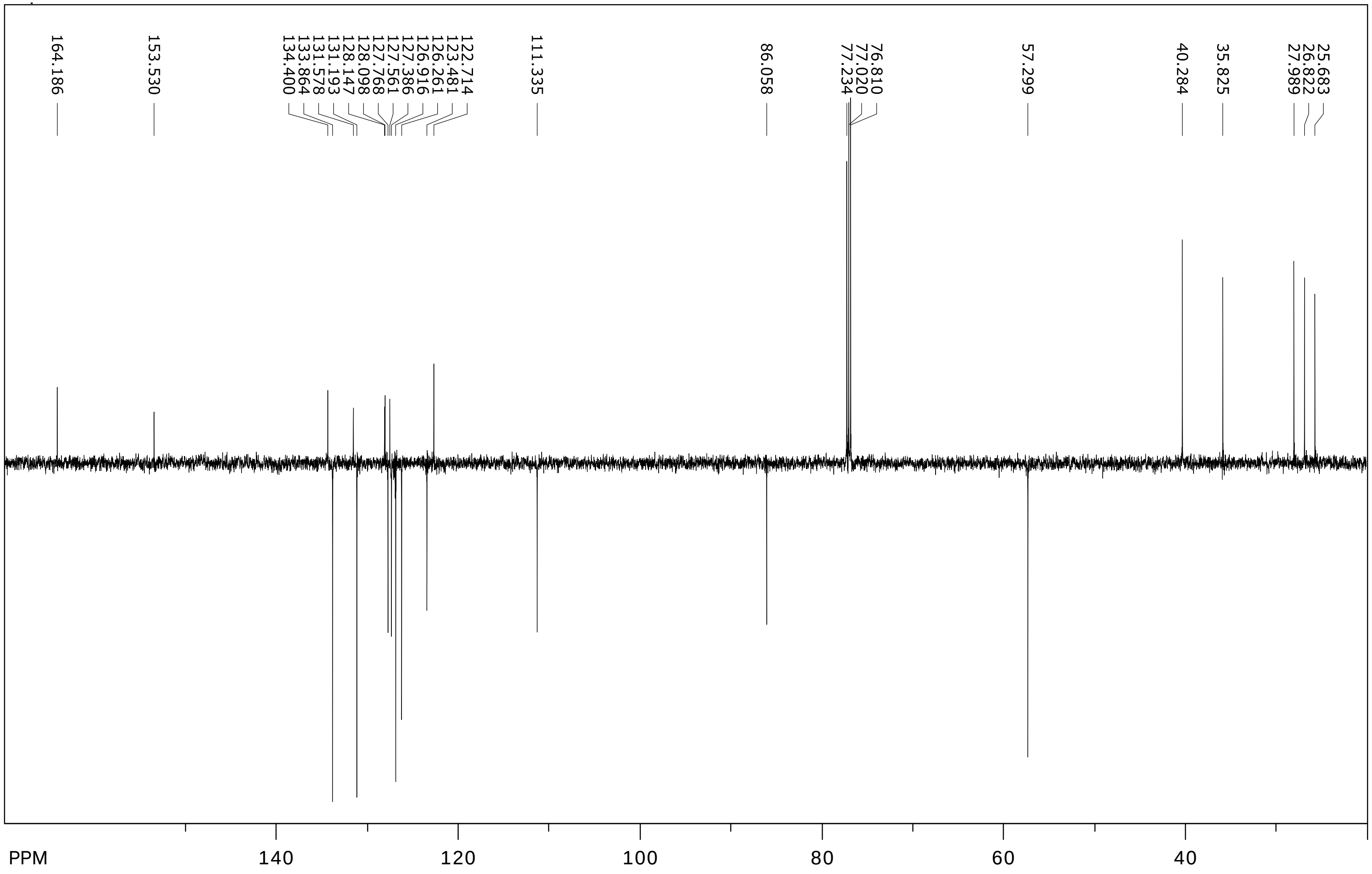

$^{1}$H NMR (CDCl3, 600 MHz) of **1d-OMe**

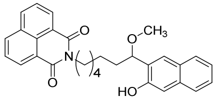

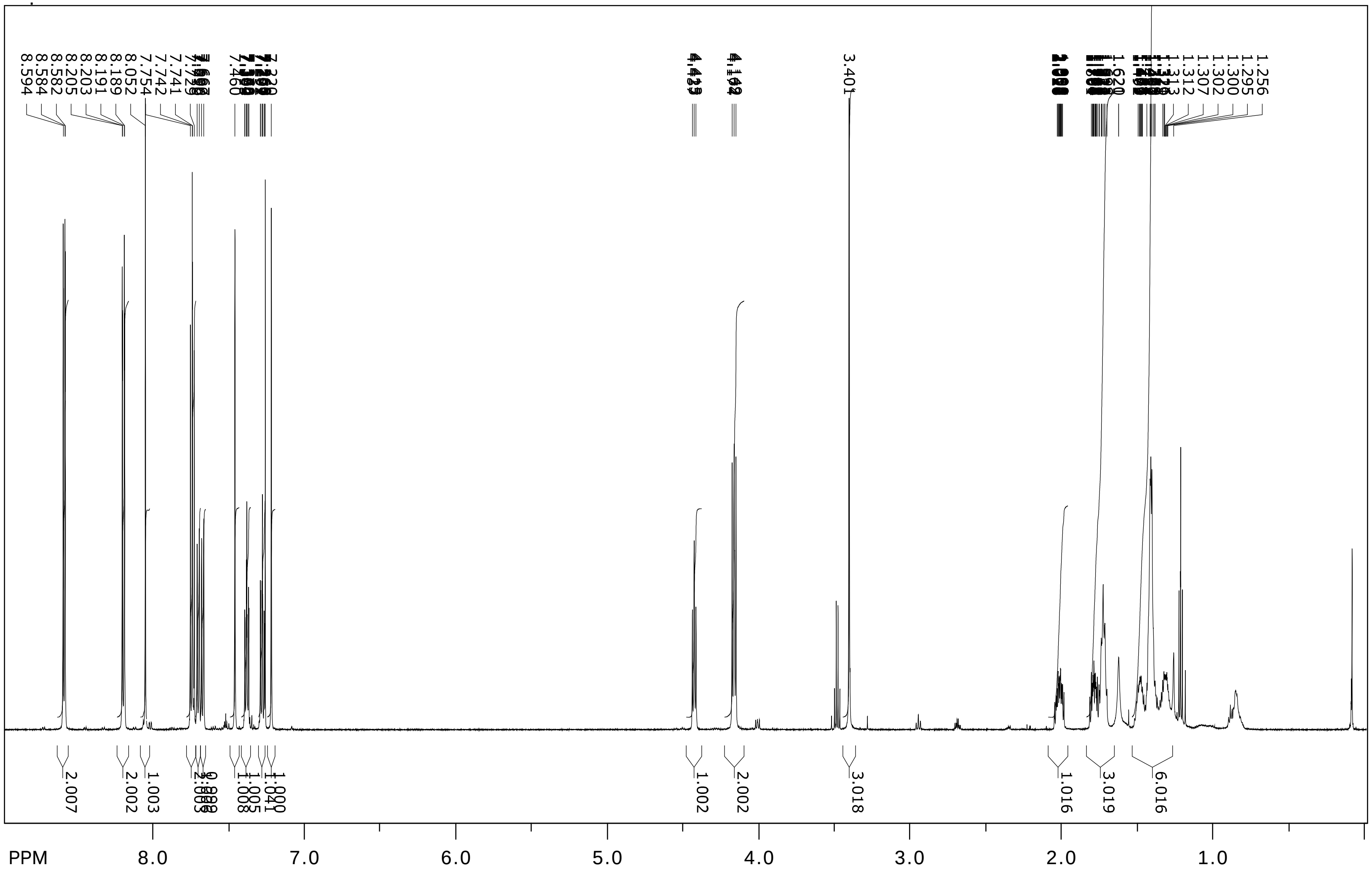

$^{13}$C NMR ($CDCl_3$, 150 MHz) of **1d-OMe** 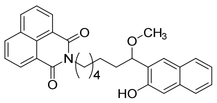

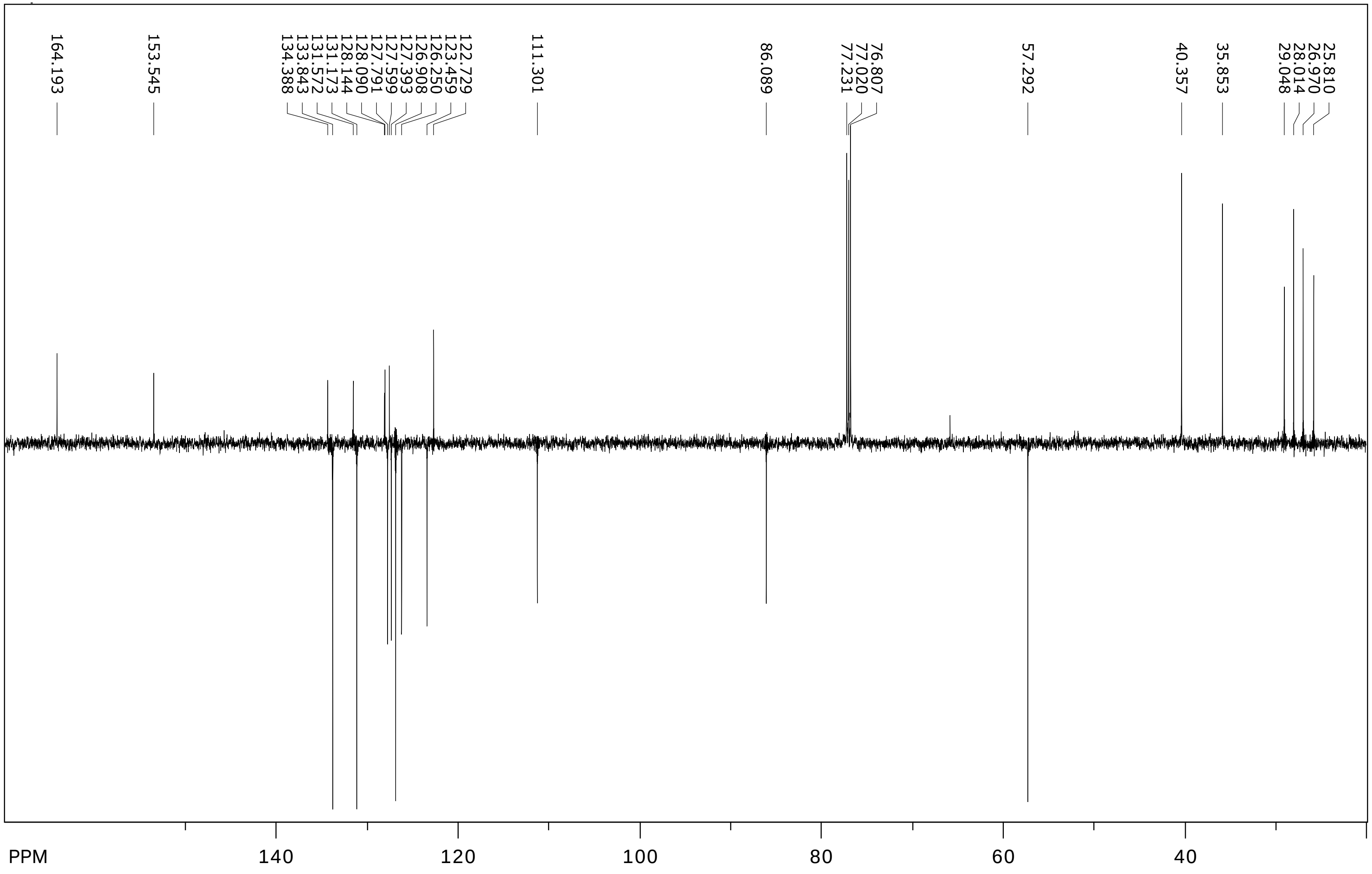

$^{1}$H NMR ($CDCl_3$, 600 MHz) of **1e-OMe**

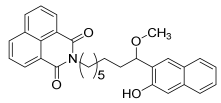

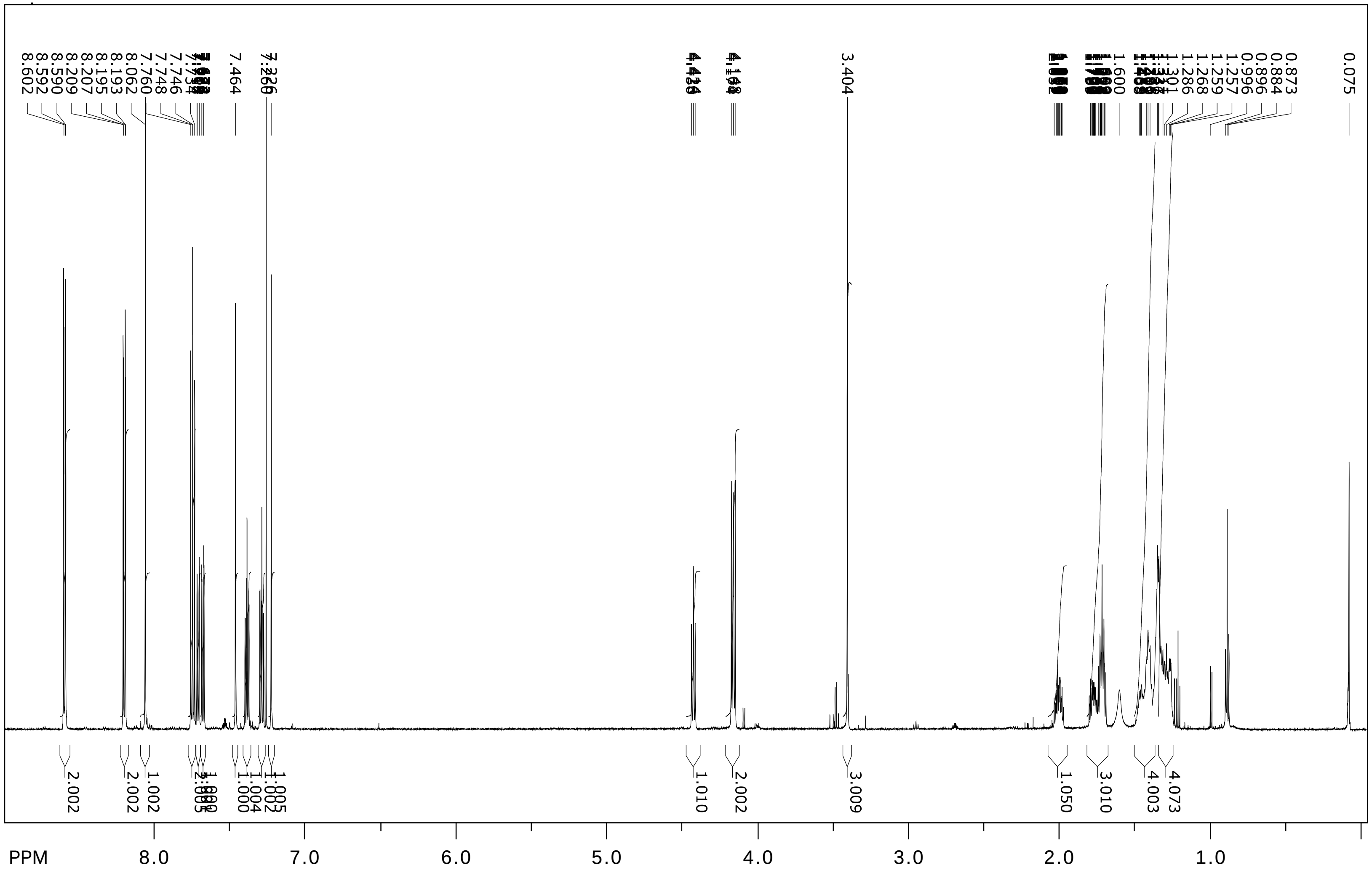

$^{13}$C NMR ($CDCl_3$, 150 MHz) of **1e-OMe**

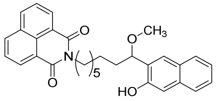


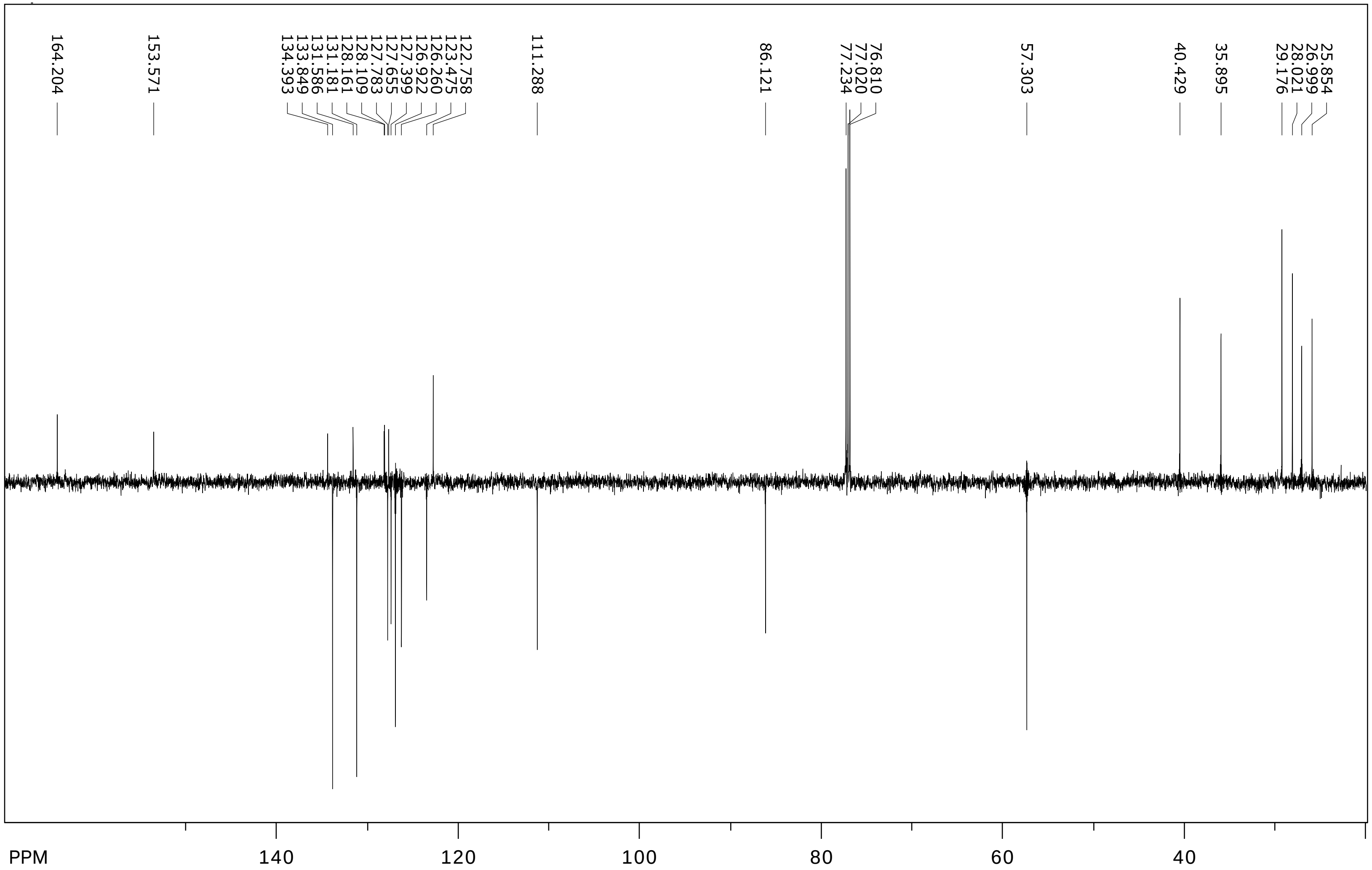

UPLC chromatogram and mass spectrum (method ②) of **1c** after the irradiation (14×8 W, 350 nm, 180 min) in $CH_3OH$-$H_2O$ (4:1 v/v). $t_R$ (**1c**) = 1,23 min, $m/z$ = 422.18 $[M–OH]^+$, found 422.26; $t_R$ (**1c-OMe**) = 1.41 min, $m/z$ = 422.18 $[M–OCH_3]^+$, found 422.32; $t_R$ (1,8-naphthalimide) = 0.72 min, $m/z$ = 198.05 $[M+H]^+$, found 198.05.

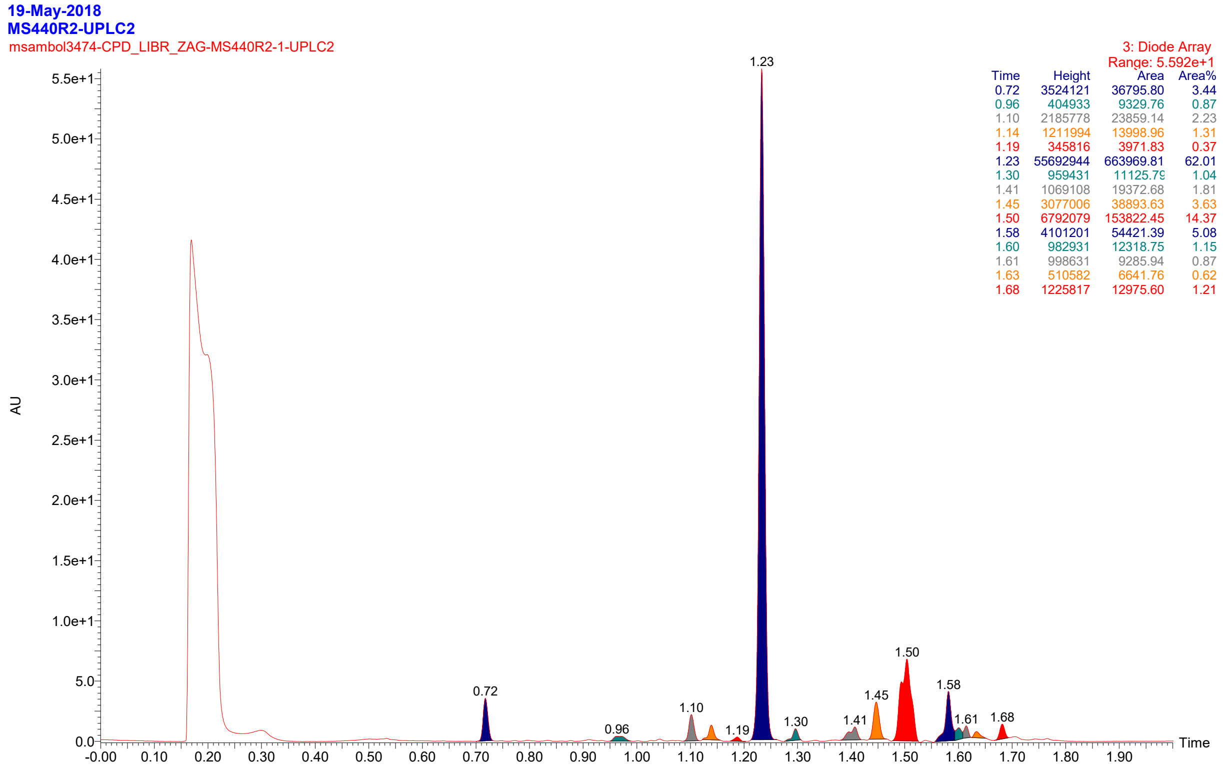


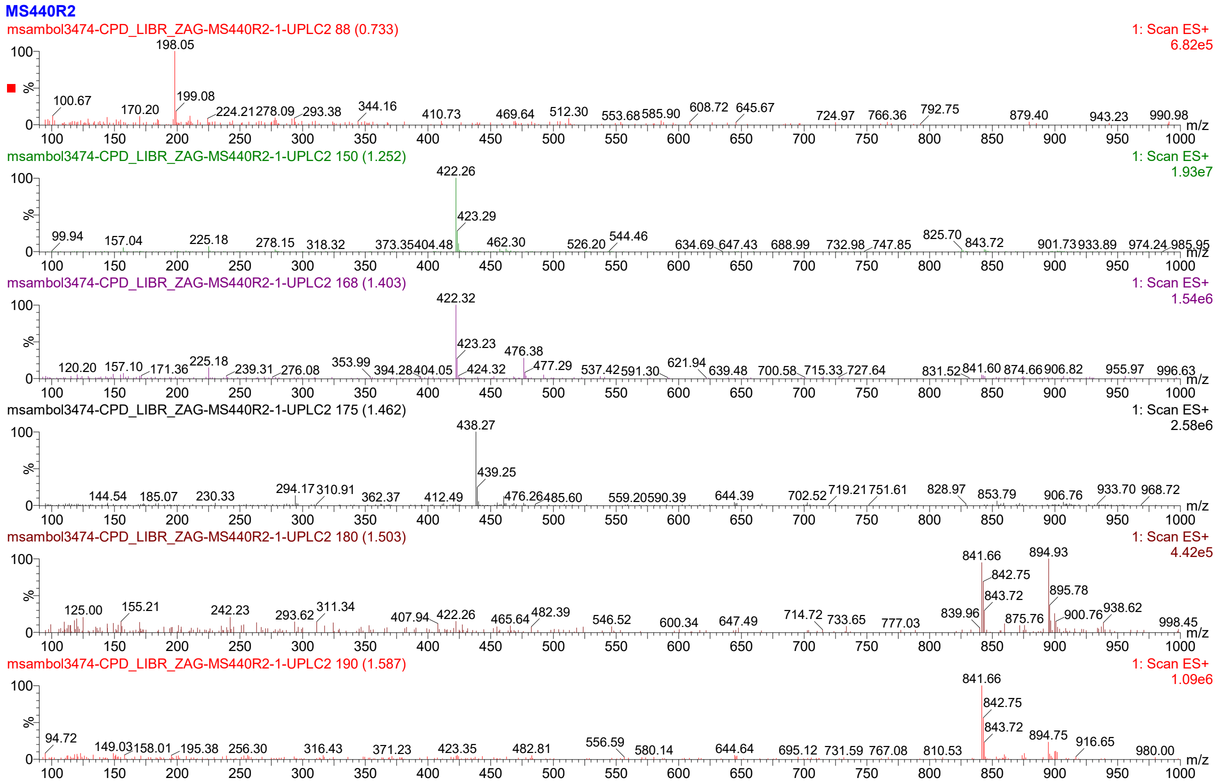

**References**

[1] Ramchander, J.; Rameshwar, N.; Reddy, T. S.; Raju, G.; Reddy, A. R. Synthesis and photophysical properties of 1,4-disubstituted naphthyloxymethyl-*N*-alkyl naphthimido-1,2,3-triazole, *J. Chem. Sci.* **2014**, *126*, 1063-1074.
[2] Hossain, S. U.; Sengupta, S.; Bhattacharya, S. Synthesis and evaluation of antioxidative properties of a series of organoselenium compounds, *Bioorg. Med. Chem.* **2005**, *13*, 5750-5758.
[3] Mo, S.; Meng, Q.; Wan, S.; Su, Z.; Yan, H.; Tang, B. Z.; Yin, M. Tunable Mechanoresponsive Self-Assembly of an Amide-Linked Dyad with Dual Sensitivity of Photochromism and Mechanochromism, *Adv. Funct. Mater.* **2017**, *27*, 1701210.
[4] Veljković, J.; Uzelac, L.; Molčanov, K.; Mlinarić-Majerski, K.; Kralj, M.; Wan, P.; Basarić, N. Sterically Congested Adamantylnaphthalene Quinone Methides, *J. Org. Chem*. **2012**, *77*, 4596-4610.
[5] Uzelac, L.; Škalamera, Đ.; Mlinarić-Majerski, K.; Basarić, N.; Kralj, M. Selective Photocytotoxicity of Anthrols on Cancer Stem-like Cells: The Effect of Quinone Methides or Reactive Oxygen Species. *Eur. J. Med. Chem*. **2017**, *137*, 558-574.
[6] Doria, F.; Richter, S. N.; Nadai, M.; Colloredo-Mels, S.; Mella, M.; Palumbo, M.; Freccero, M. Photogeneration and Reactivity of Naphthoquinone Methides as Purine Selective DNA Alkylating Agents. *J. Med. Chem*. **2007**, *50*, 6570-6579.
[7] Verga, D.; Nadai, M.; Doria, F.; Percivalle, C.; Di Antonio, M.; Palumbo, M.; Richter, S. N.; Freccero, M. Photogeneration and Reactivity of Naphthoquinone Methides as Purine Selective DNA Alkylating Agents. *J. Am. Chem. Soc*. **2010**, *132*, 14625-14637.
